# Supplementary figures and images for: TMEM25 is a Par3-binding protein that attenuates claudin assembly during tight junction development (part 1 of 3)
Source: EMBO Rep. 2023 Dec 18;25(1):13. doi: 10.1038/s44319-023-00018-0 (PMC10897455; doi:10.1038/s44319-023-00018-0)

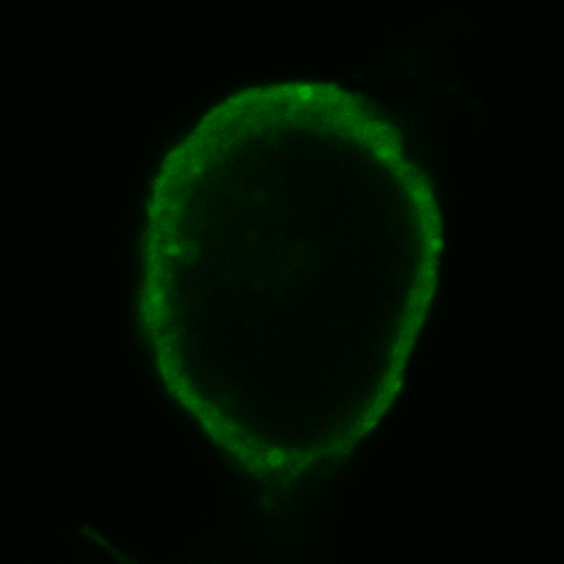

Supplement: Supplementary file 2 — Source Data Fig. 1 [file 44319_2023_18_MOESM2_ESM.zip › Figure EV2/EV2A/Right_HA.tif]

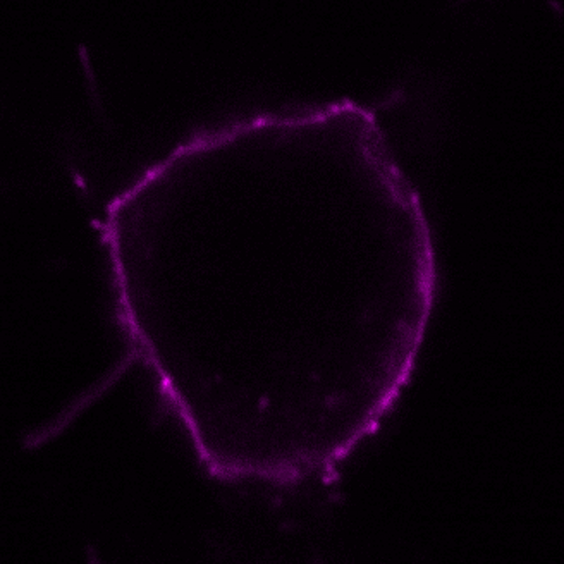

Supplement: Supplementary file 2 — Source Data Fig. 1 [file 44319_2023_18_MOESM2_ESM.zip › Figure EV2/EV2A/Left_FLAG.tif]

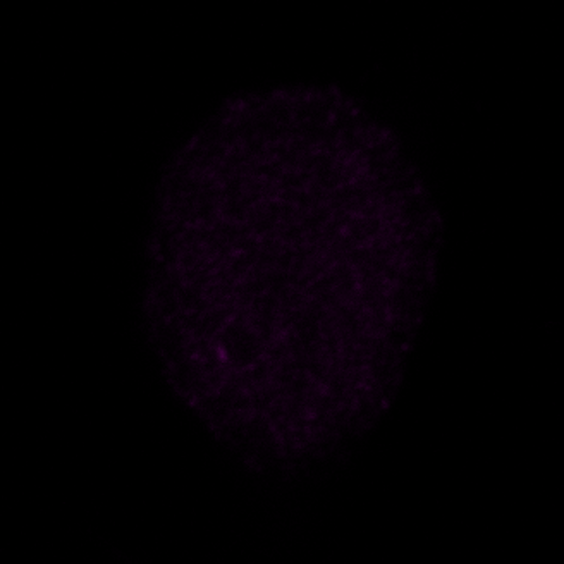

Supplement: Supplementary file 2 — Source Data Fig. 1 [file 44319_2023_18_MOESM2_ESM.zip › Figure EV2/EV2A/Right_FLAG.tif]

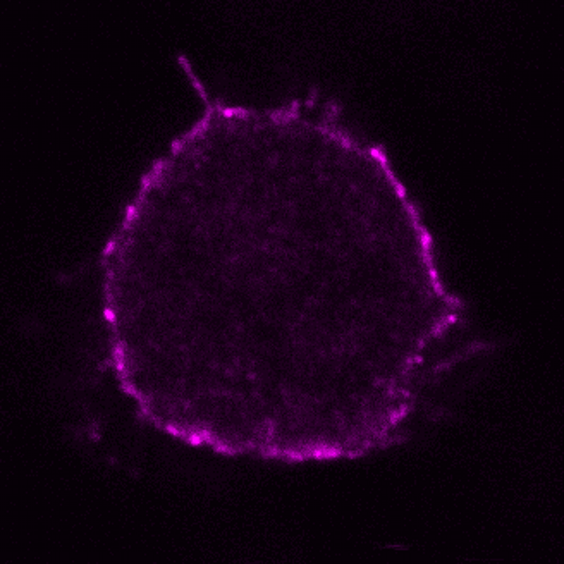

Supplement: Supplementary file 2 — Source Data Fig. 1 [file 44319_2023_18_MOESM2_ESM.zip › Figure EV2/EV2A/Middle_FLAG.tif]

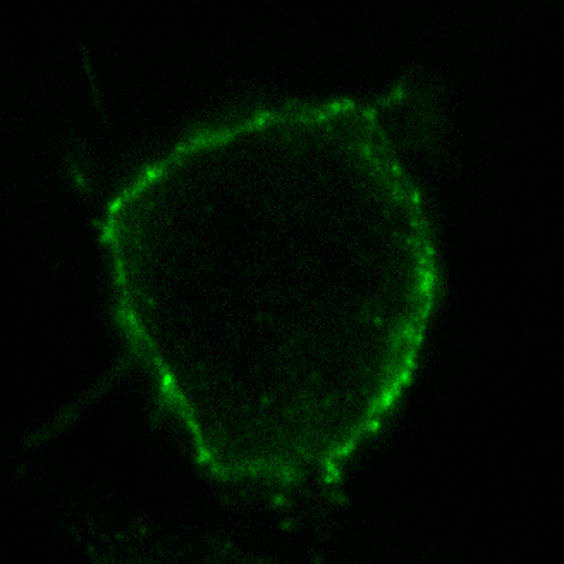

Supplement: Supplementary file 2 — Source Data Fig. 1 [file 44319_2023_18_MOESM2_ESM.zip › Figure EV2/EV2A/Left_HA.tif]

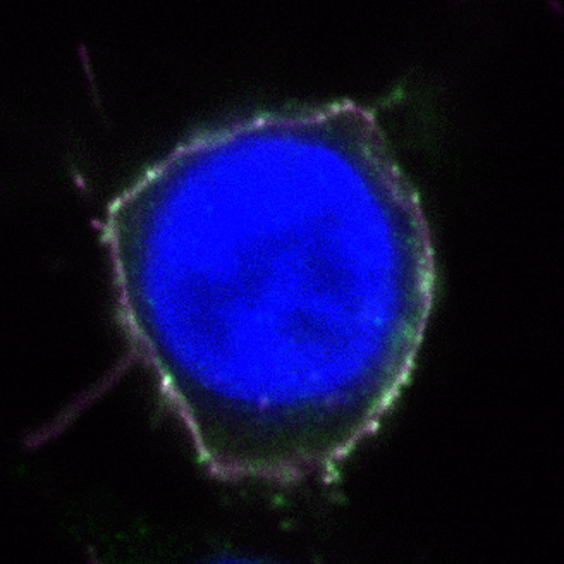

Supplement: Supplementary file 2 — Source Data Fig. 1 [file 44319_2023_18_MOESM2_ESM.zip › Figure EV2/EV2A/Left_merge.tif]

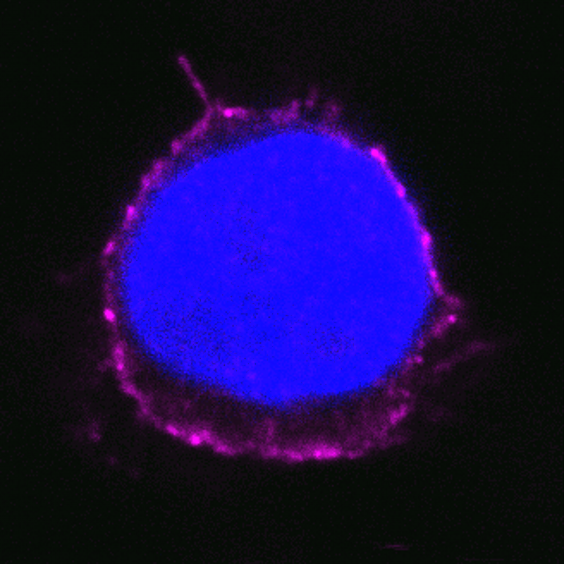

Supplement: Supplementary file 2 — Source Data Fig. 1 [file 44319_2023_18_MOESM2_ESM.zip › Figure EV2/EV2A/Middle_merge.tif]

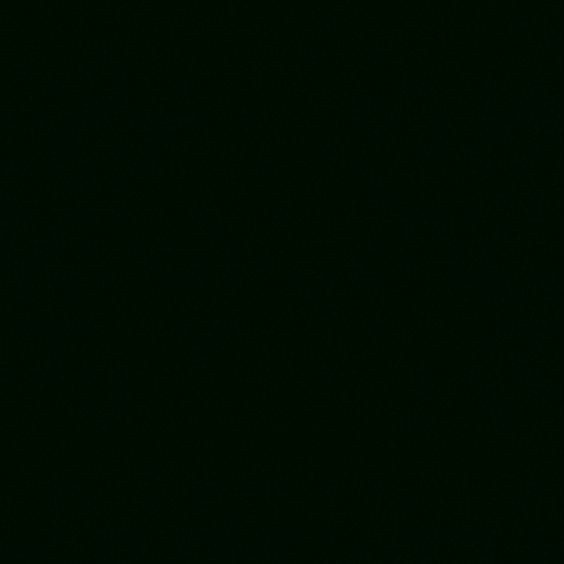

Supplement: Supplementary file 2 — Source Data Fig. 1 [file 44319_2023_18_MOESM2_ESM.zip › Figure EV2/EV2A/Middle_HA.tif]

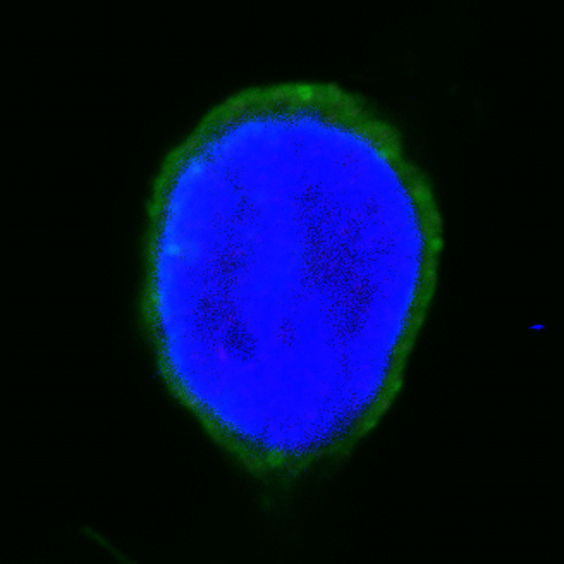

Supplement: Supplementary file 2 — Source Data Fig. 1 [file 44319_2023_18_MOESM2_ESM.zip › Figure EV2/EV2A/Right_merge.tif]

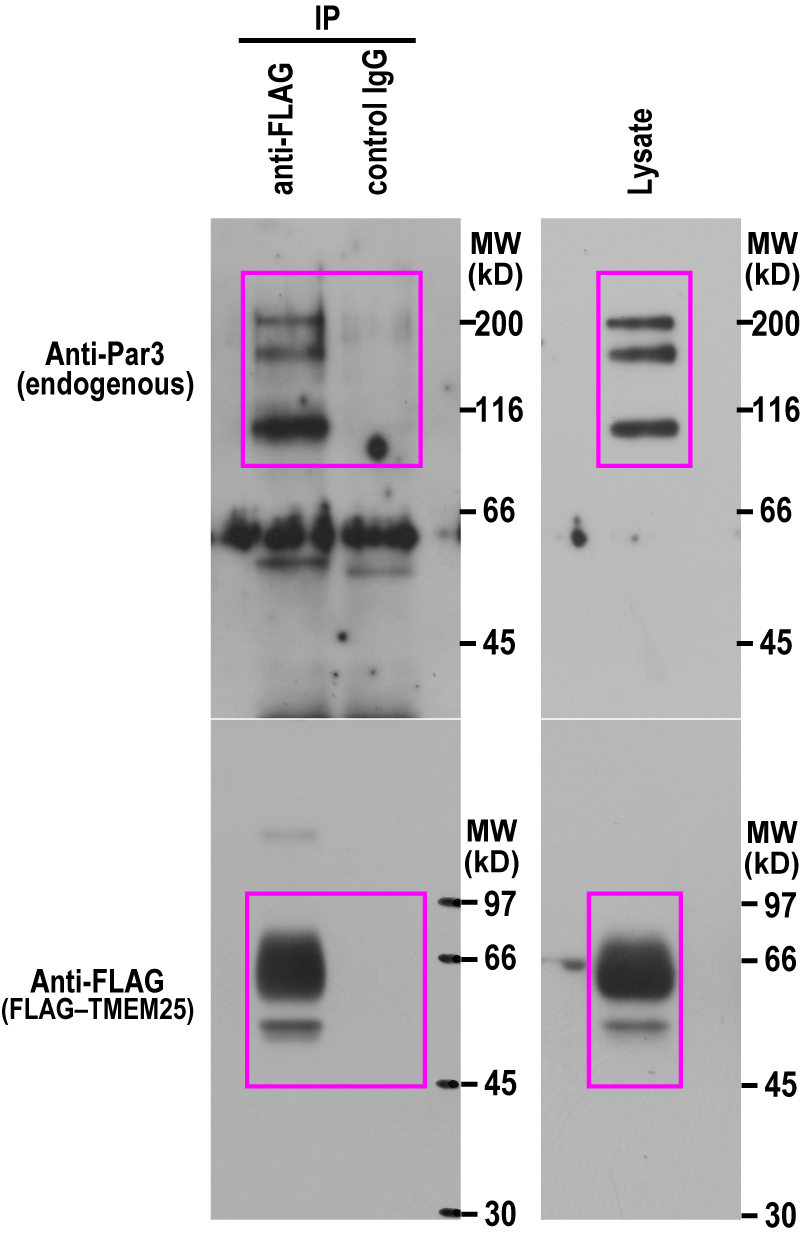

Supplement: Supplementary file 3 — Source Data Fig. 2 [file 44319_2023_18_MOESM3_ESM.zip › Figure_1/1H/1H.tif]

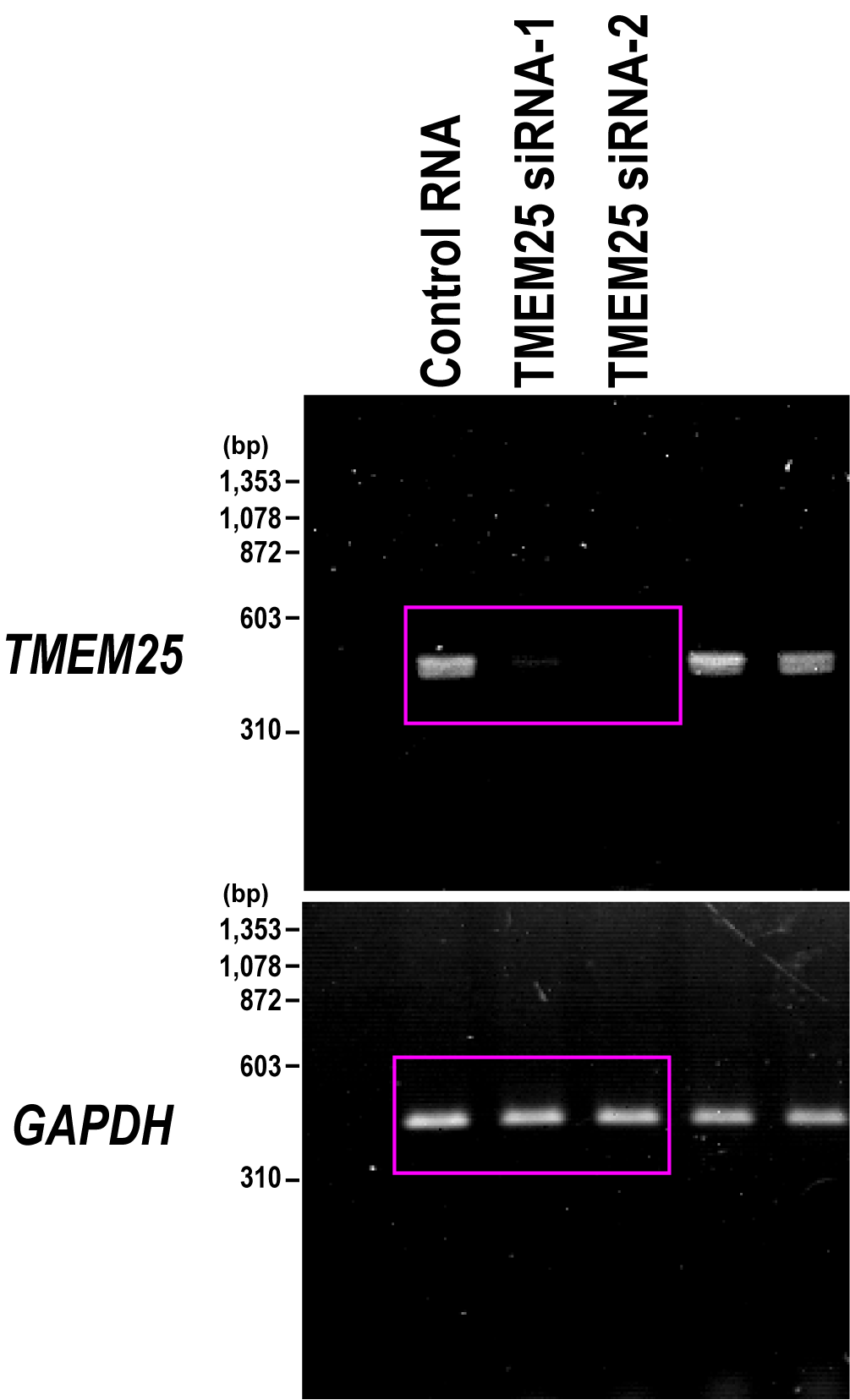

Supplement: Supplementary file 3 — Source Data Fig. 2 [file 44319_2023_18_MOESM3_ESM.zip › Figure_1/1F/1F RT-PCR.tif]

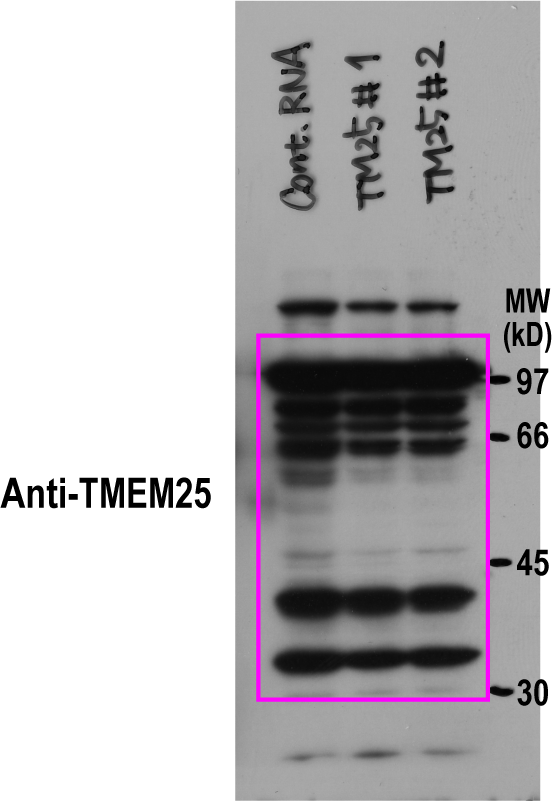

Supplement: Supplementary file 3 — Source Data Fig. 2 [file 44319_2023_18_MOESM3_ESM.zip › Figure_1/1F/1F immunoblot.tif]

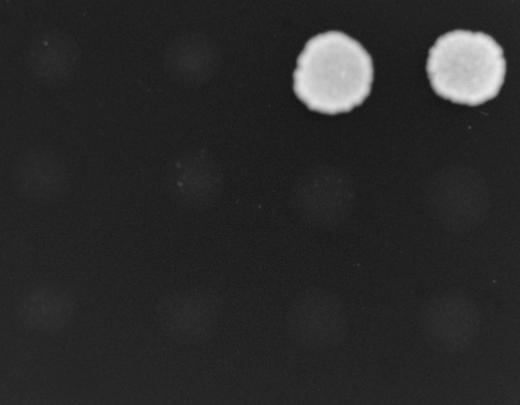

Supplement: Supplementary file 3 — Source Data Fig. 2 [file 44319_2023_18_MOESM3_ESM.zip › Figure_1/1B/His(-).tif]

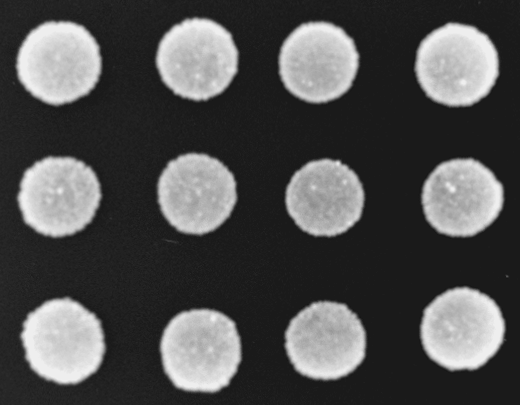

Supplement: Supplementary file 3 — Source Data Fig. 2 [file 44319_2023_18_MOESM3_ESM.zip › Figure_1/1B/His(+).tif]

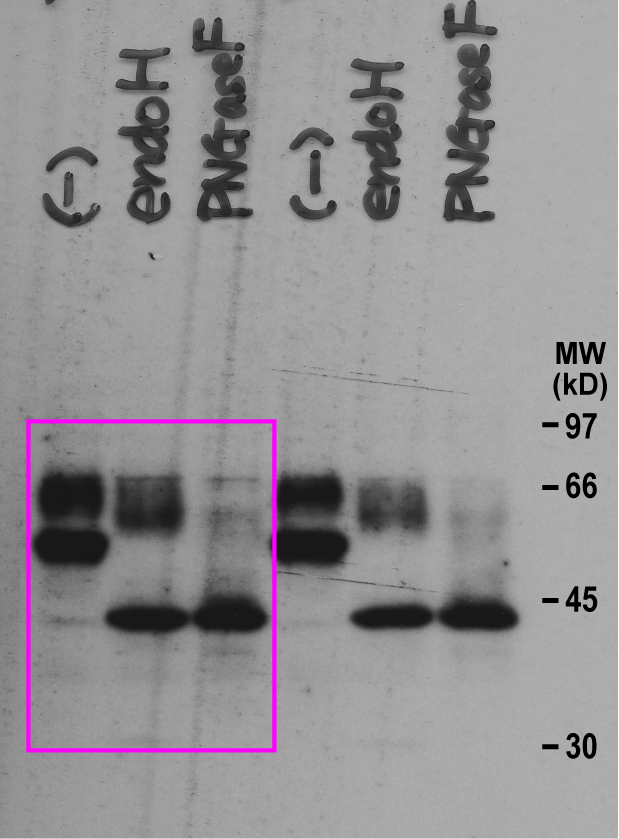

Supplement: Supplementary file 3 — Source Data Fig. 2 [file 44319_2023_18_MOESM3_ESM.zip › Figure_1/1E/1E.tif]

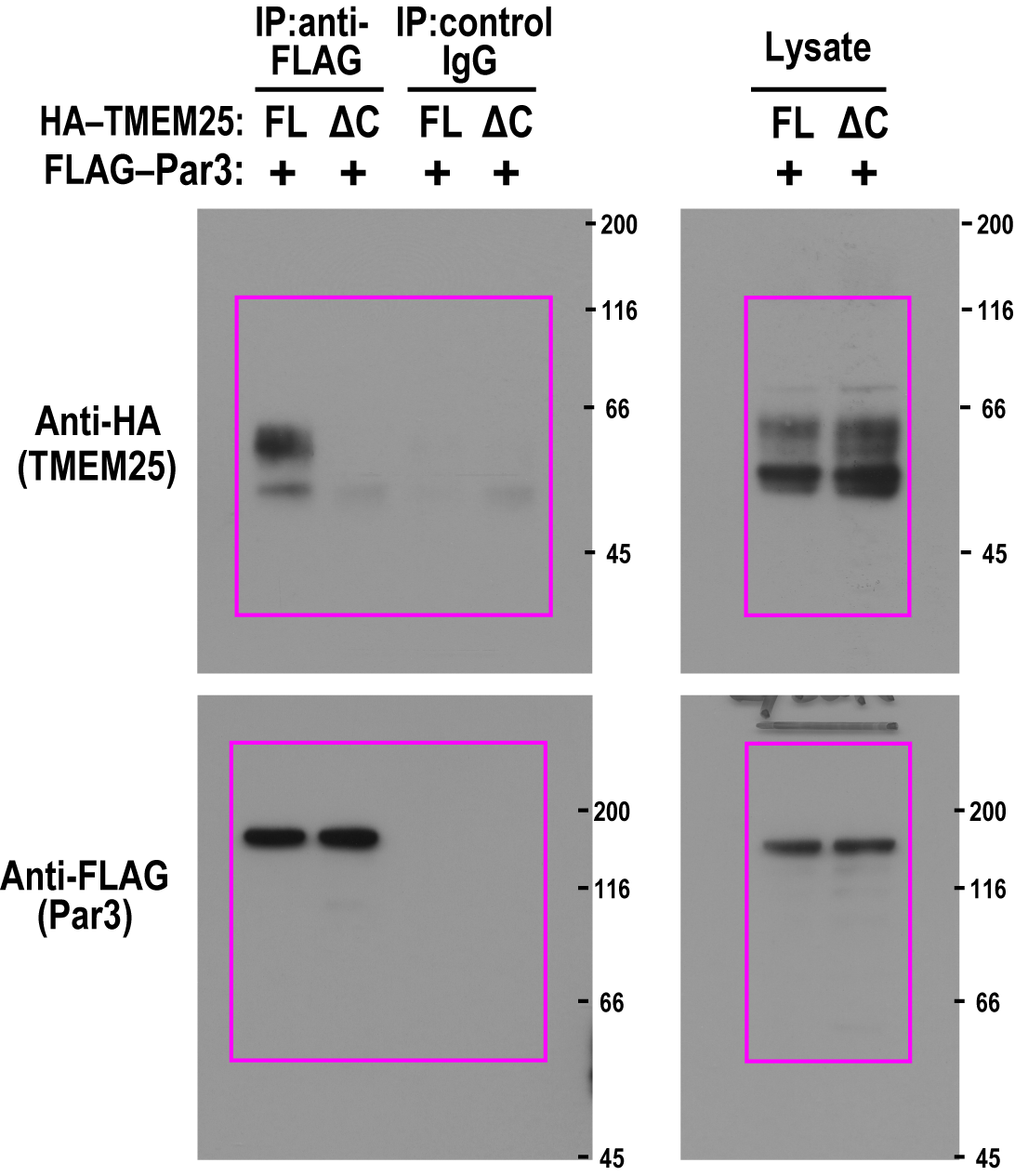

Supplement: Supplementary file 3 — Source Data Fig. 2 [file 44319_2023_18_MOESM3_ESM.zip › Figure_1/1D/1D.tif]

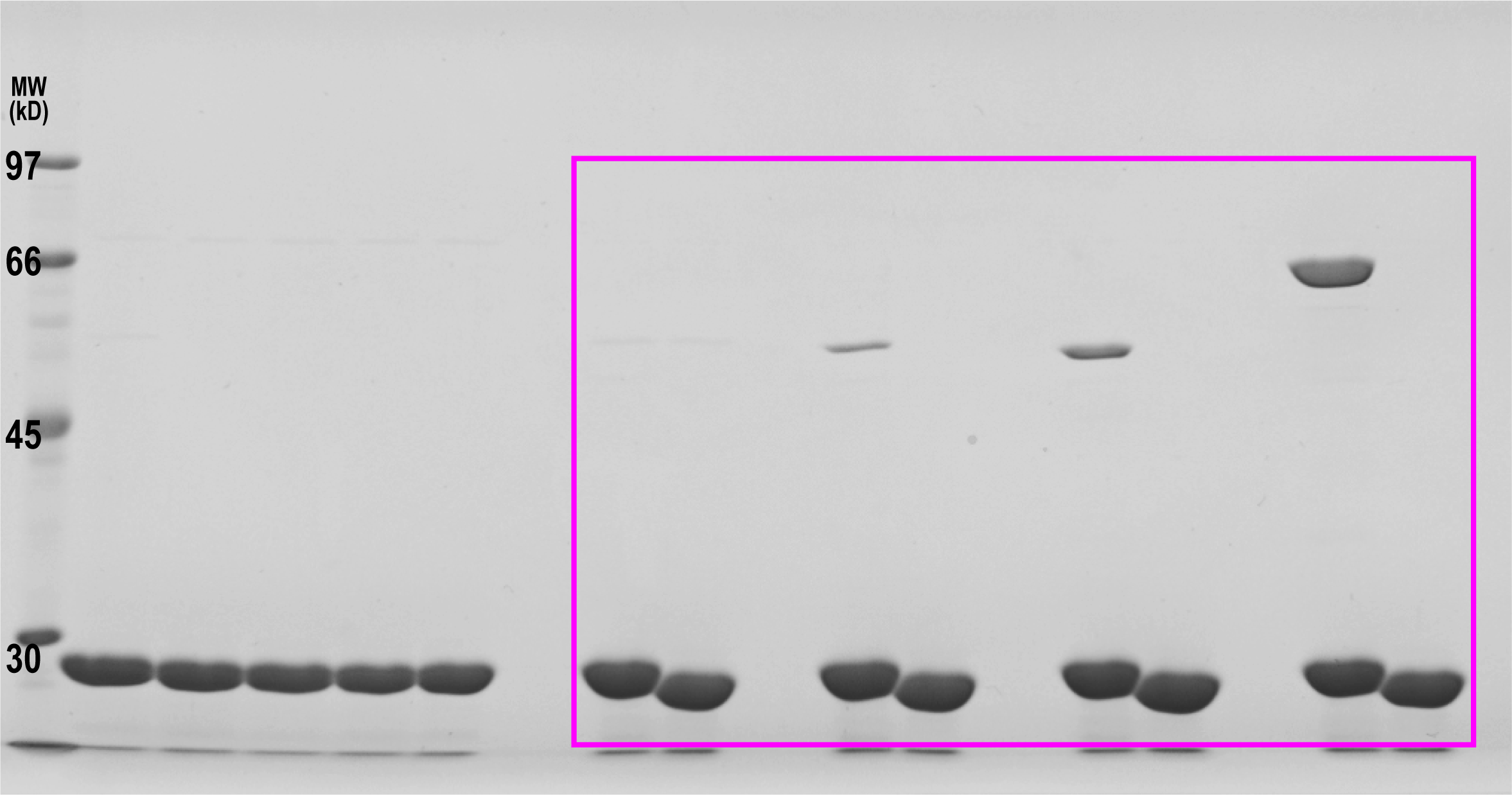

Supplement: Supplementary file 3 — Source Data Fig. 2 [file 44319_2023_18_MOESM3_ESM.zip › Figure_1/1C/pulldown.tif]

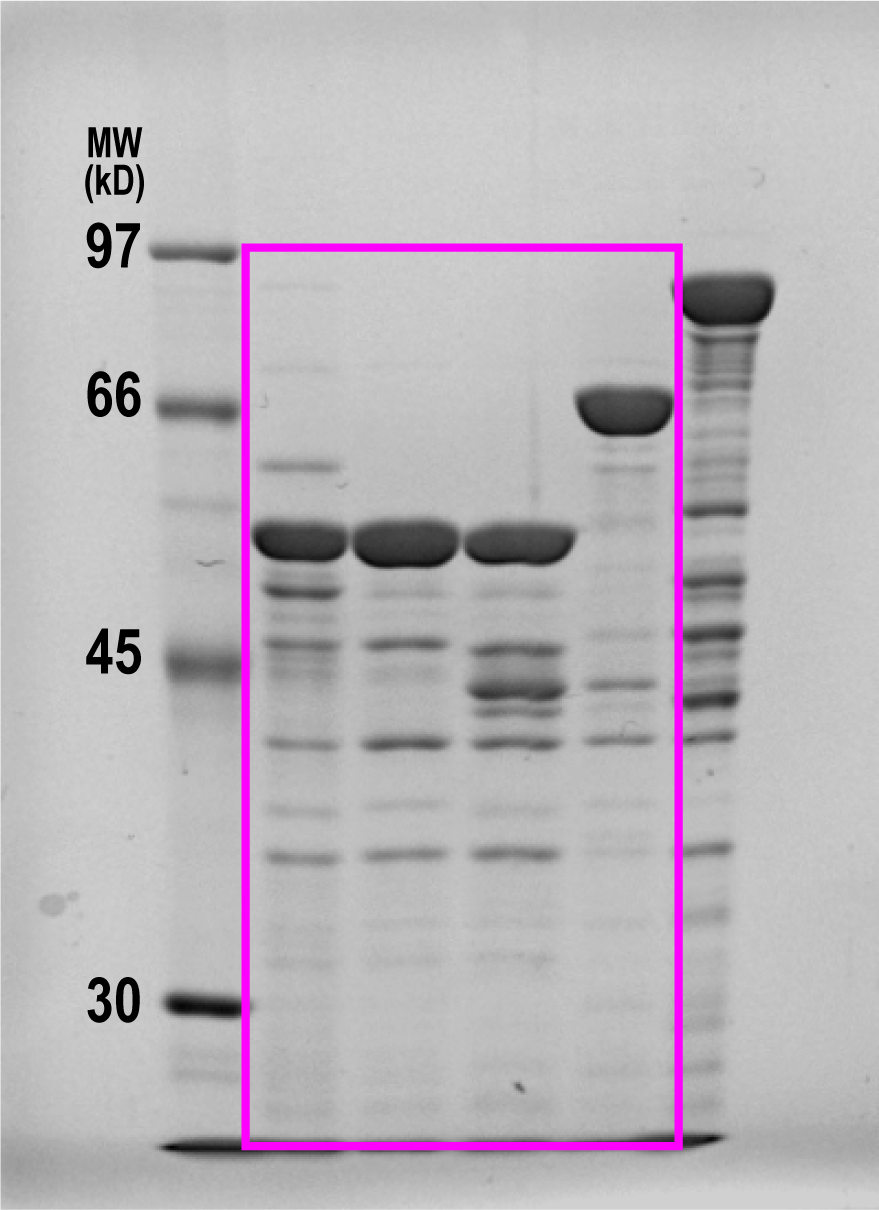

Supplement: Supplementary file 3 — Source Data Fig. 2 [file 44319_2023_18_MOESM3_ESM.zip › Figure_1/1C/Input.tif]

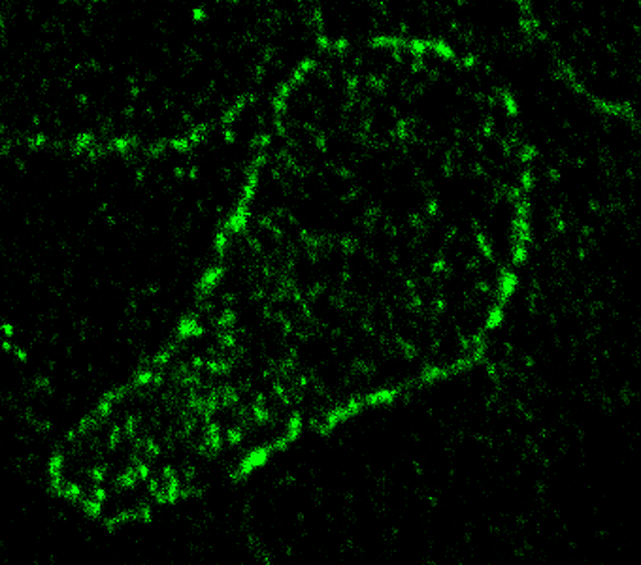

Supplement: Supplementary file 3 — Source Data Fig. 2 [file 44319_2023_18_MOESM3_ESM.zip › Figure_1/1G/upper panels/FLAG_xy.tif]

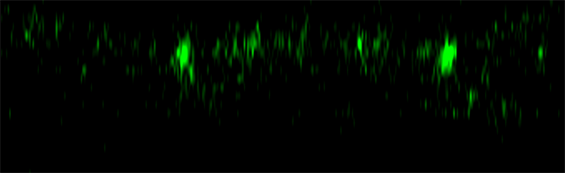

Supplement: Supplementary file 3 — Source Data Fig. 2 [file 44319_2023_18_MOESM3_ESM.zip › Figure_1/1G/upper panels/FLAG_xz.tif]

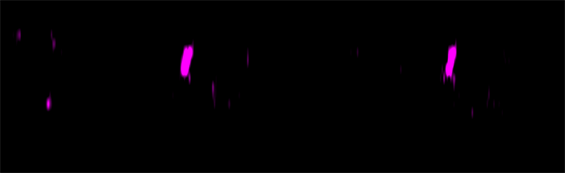

Supplement: Supplementary file 3 — Source Data Fig. 2 [file 44319_2023_18_MOESM3_ESM.zip › Figure_1/1G/upper panels/ZO1_xz.tif]

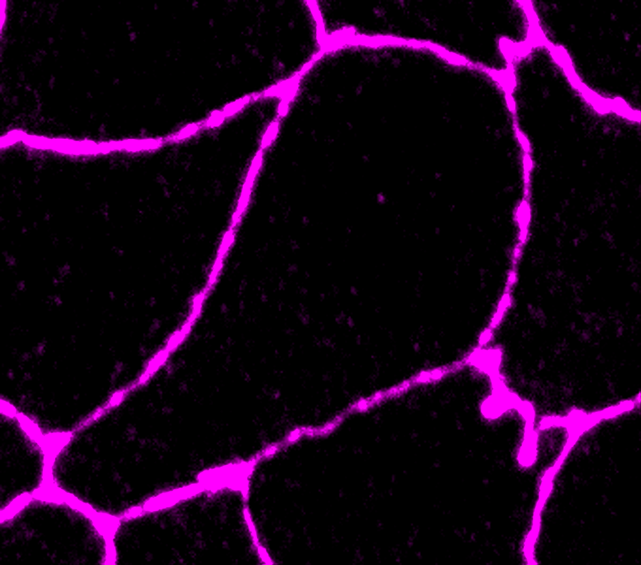

Supplement: Supplementary file 3 — Source Data Fig. 2 [file 44319_2023_18_MOESM3_ESM.zip › Figure_1/1G/upper panels/ZO1_xy.tif]

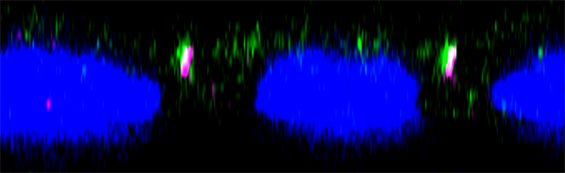

Supplement: Supplementary file 3 — Source Data Fig. 2 [file 44319_2023_18_MOESM3_ESM.zip › Figure_1/1G/upper panels/Merge_xz.tif]

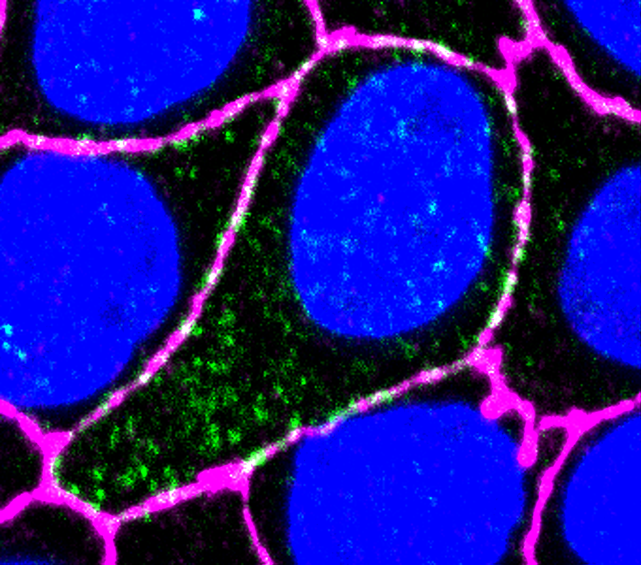

Supplement: Supplementary file 3 — Source Data Fig. 2 [file 44319_2023_18_MOESM3_ESM.zip › Figure_1/1G/upper panels/Merge_xy.tif]

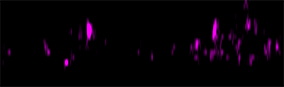

Supplement: Supplementary file 3 — Source Data Fig. 2 [file 44319_2023_18_MOESM3_ESM.zip › Figure_1/1G/lower panels/Par3_xz.tif]

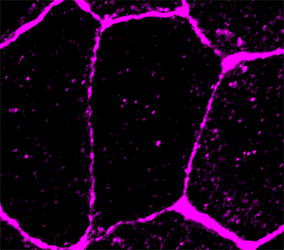

Supplement: Supplementary file 3 — Source Data Fig. 2 [file 44319_2023_18_MOESM3_ESM.zip › Figure_1/1G/lower panels/Par3_xy.tif]

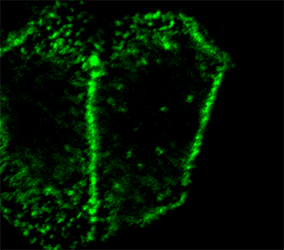

Supplement: Supplementary file 3 — Source Data Fig. 2 [file 44319_2023_18_MOESM3_ESM.zip › Figure_1/1G/lower panels/FLAG_xy.tif]

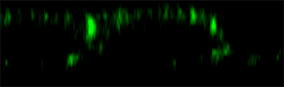

Supplement: Supplementary file 3 — Source Data Fig. 2 [file 44319_2023_18_MOESM3_ESM.zip › Figure_1/1G/lower panels/FLAG_xz.tif]

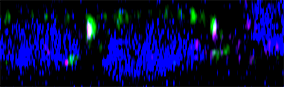

Supplement: Supplementary file 3 — Source Data Fig. 2 [file 44319_2023_18_MOESM3_ESM.zip › Figure_1/1G/lower panels/Merge_xz.tif]

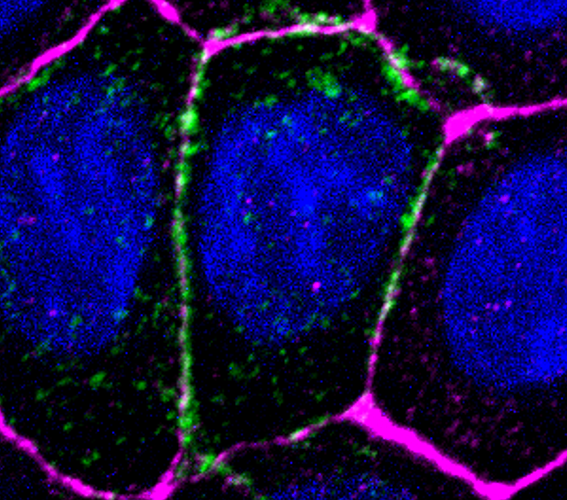

Supplement: Supplementary file 3 — Source Data Fig. 2 [file 44319_2023_18_MOESM3_ESM.zip › Figure_1/1G/lower panels/Merge_xy.tif]

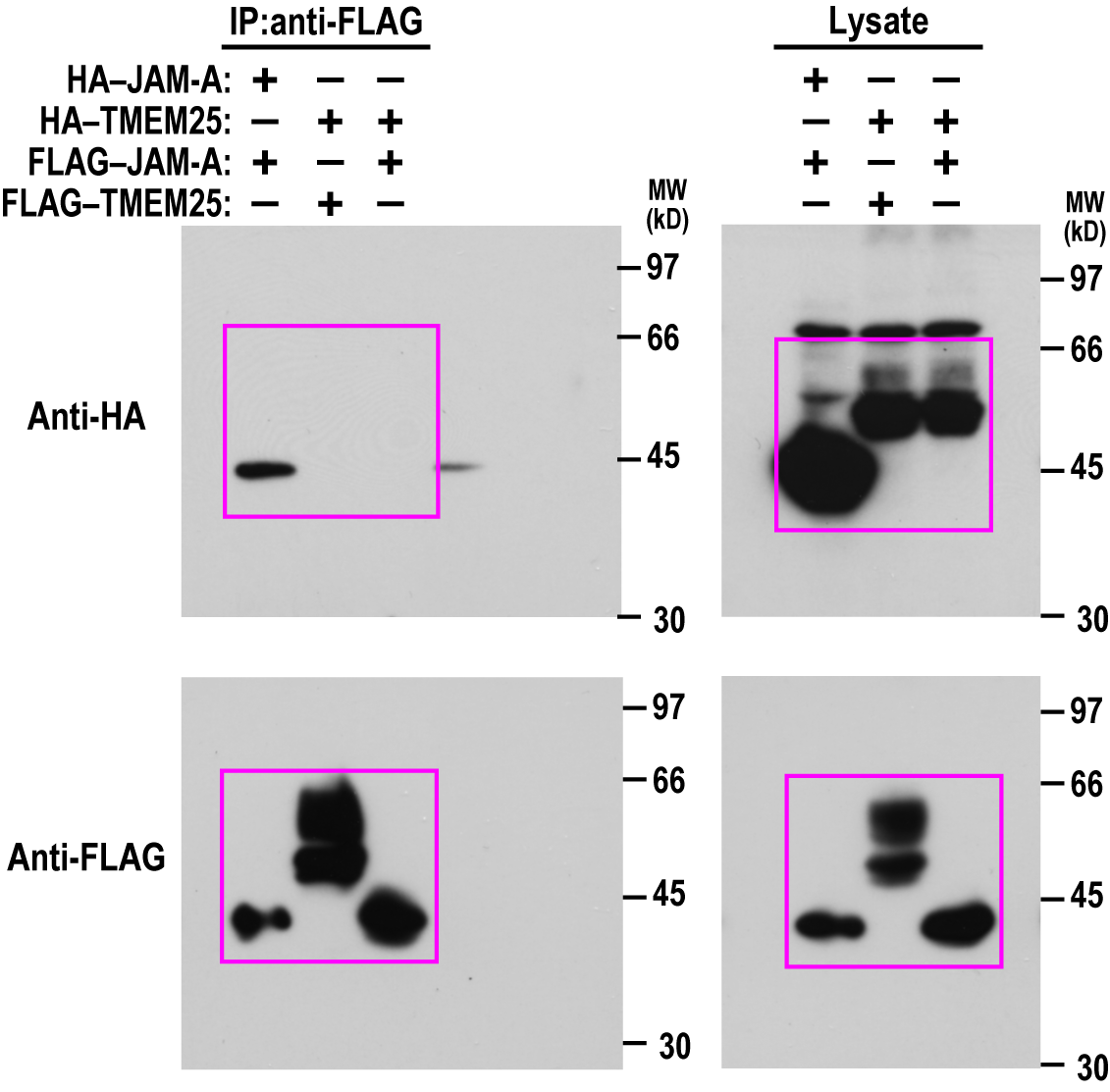

Supplement: Supplementary file 4 — Source Data Fig. 3 [file 44319_2023_18_MOESM4_ESM.zip › Figure_2/2G/2G.tif]

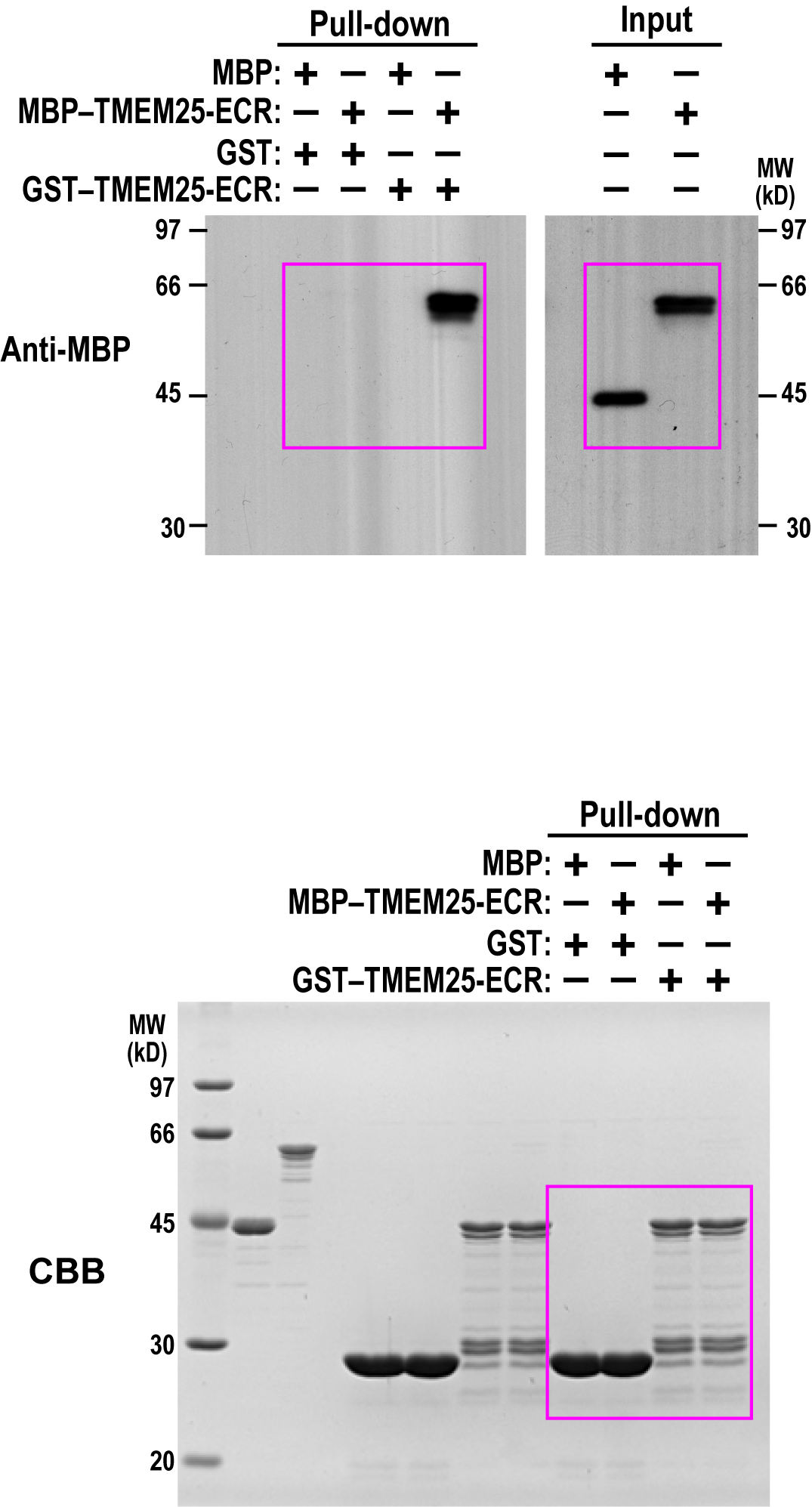

Supplement: Supplementary file 4 — Source Data Fig. 3 [file 44319_2023_18_MOESM4_ESM.zip › Figure_2/2A/2A.tif]

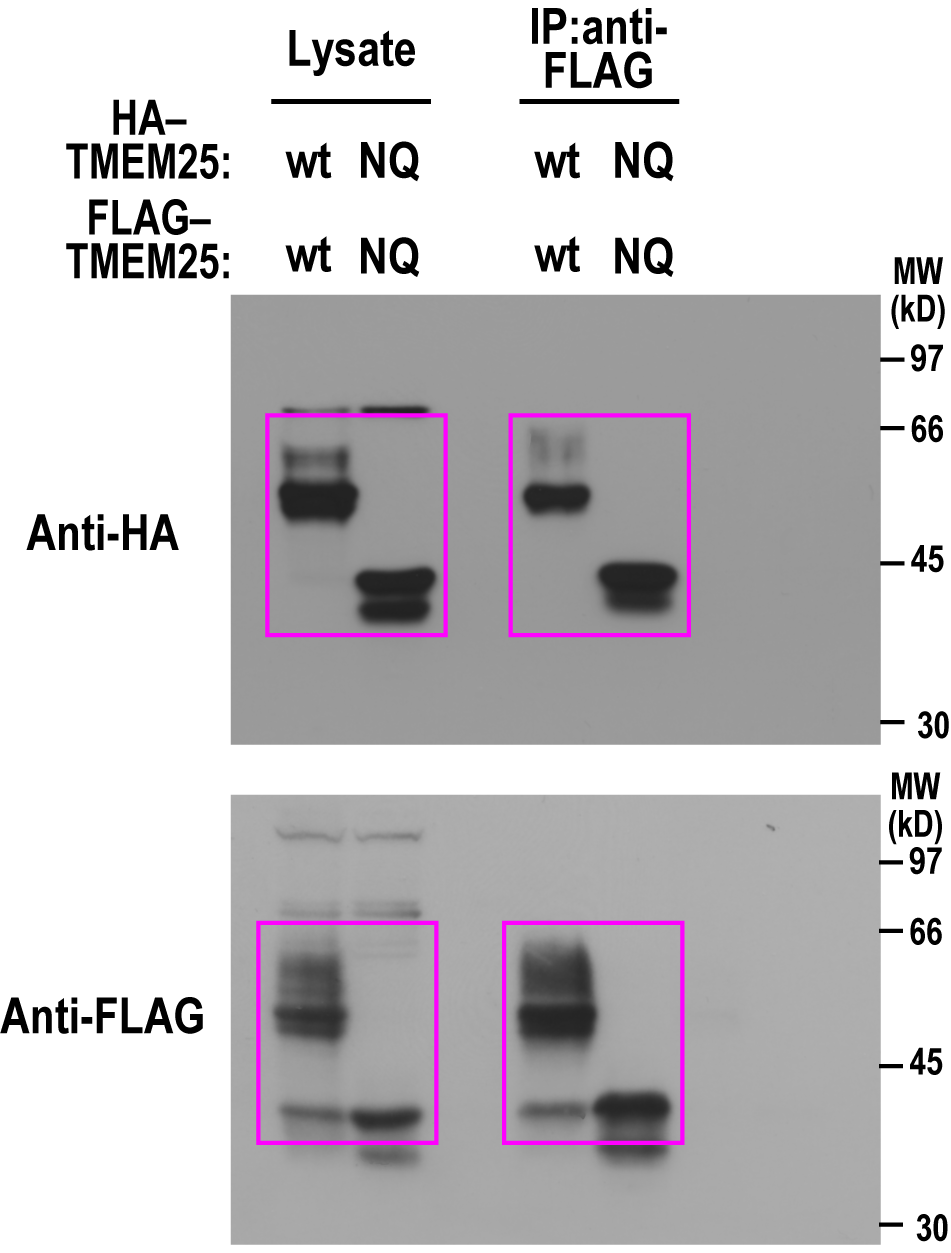

Supplement: Supplementary file 4 — Source Data Fig. 3 [file 44319_2023_18_MOESM4_ESM.zip › Figure_2/2F/2F.tif]

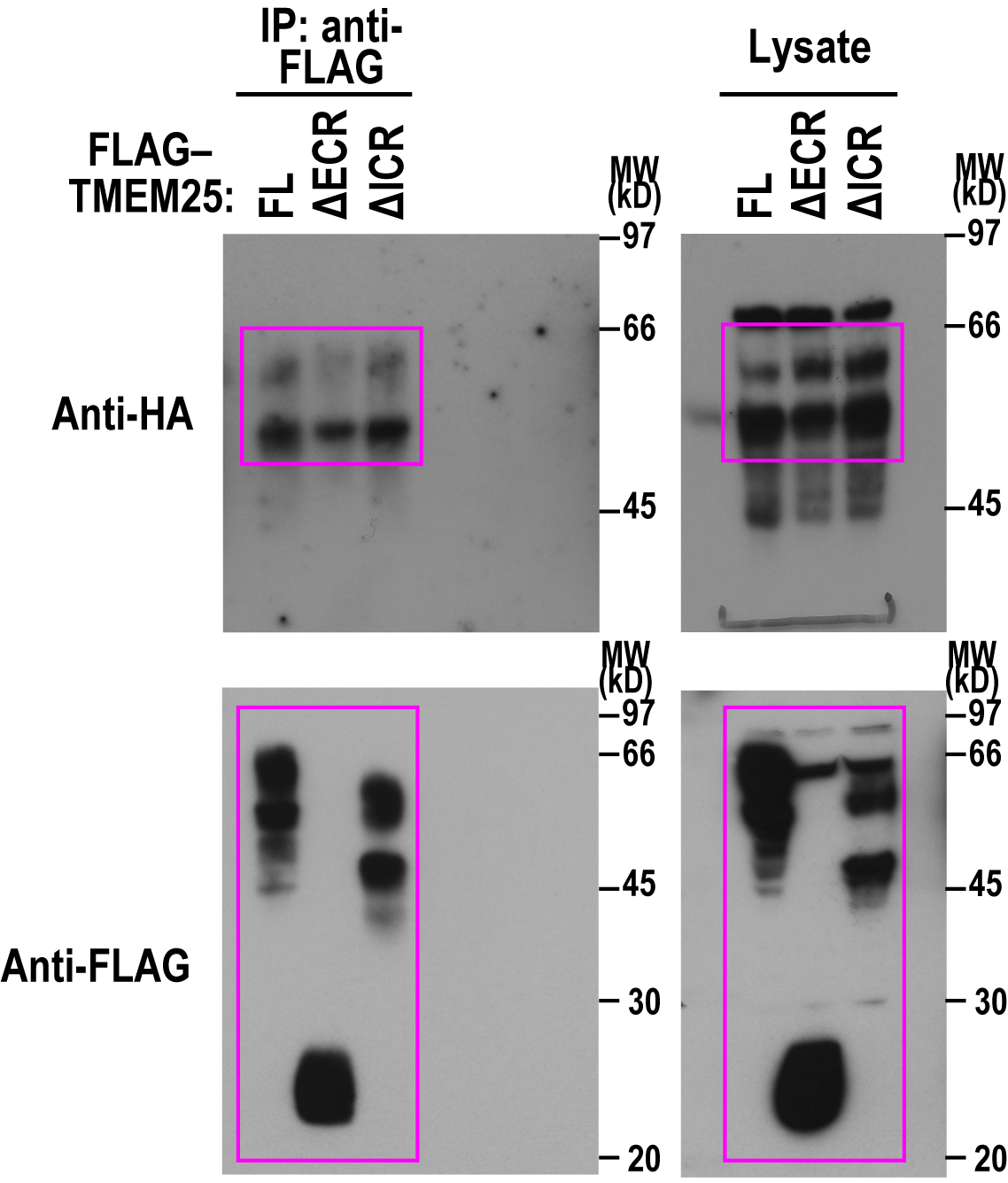

Supplement: Supplementary file 4 — Source Data Fig. 3 [file 44319_2023_18_MOESM4_ESM.zip › Figure_2/2D/2D.tif]

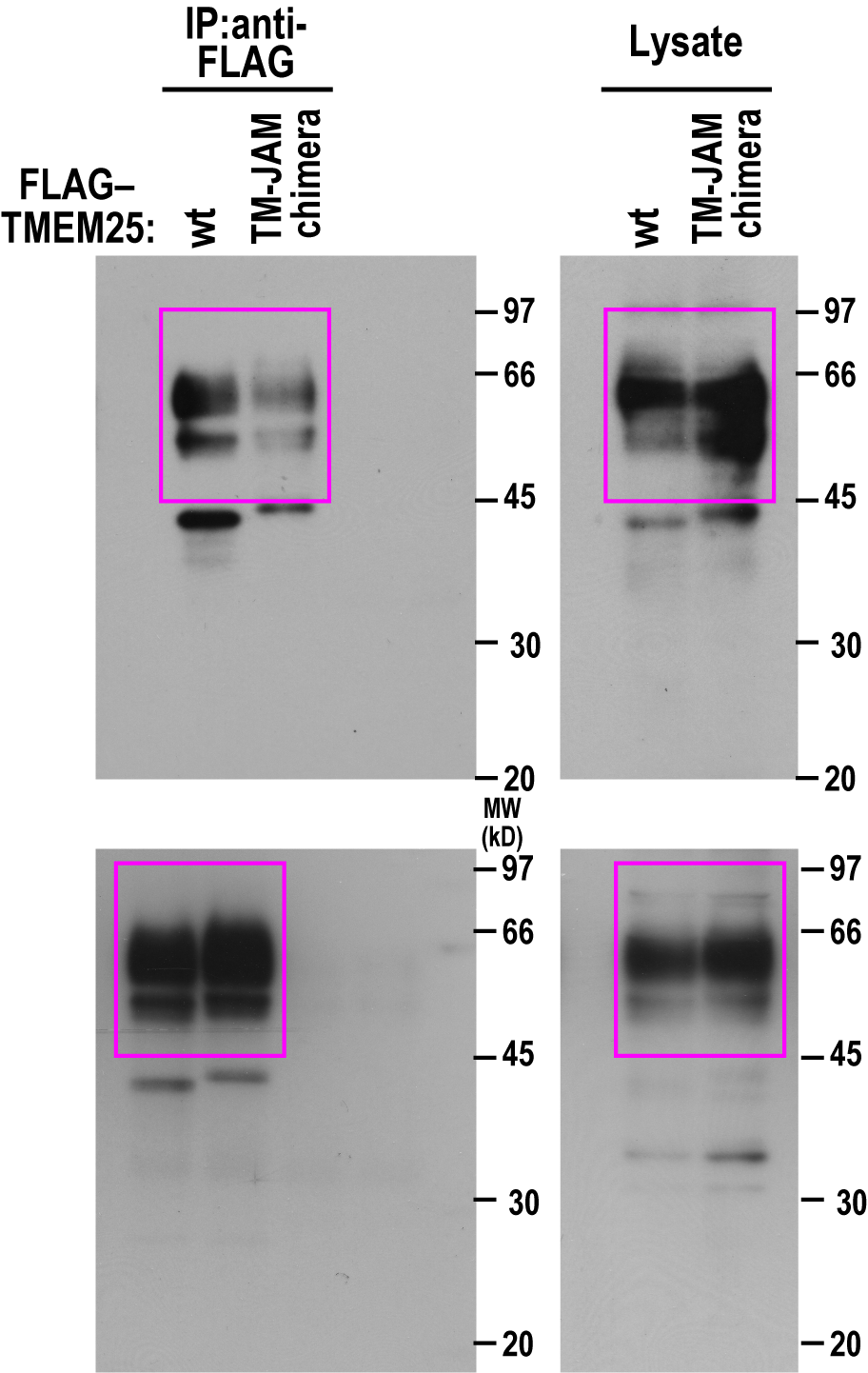

Supplement: Supplementary file 4 — Source Data Fig. 3 [file 44319_2023_18_MOESM4_ESM.zip › Figure_2/2E/2E.tif]

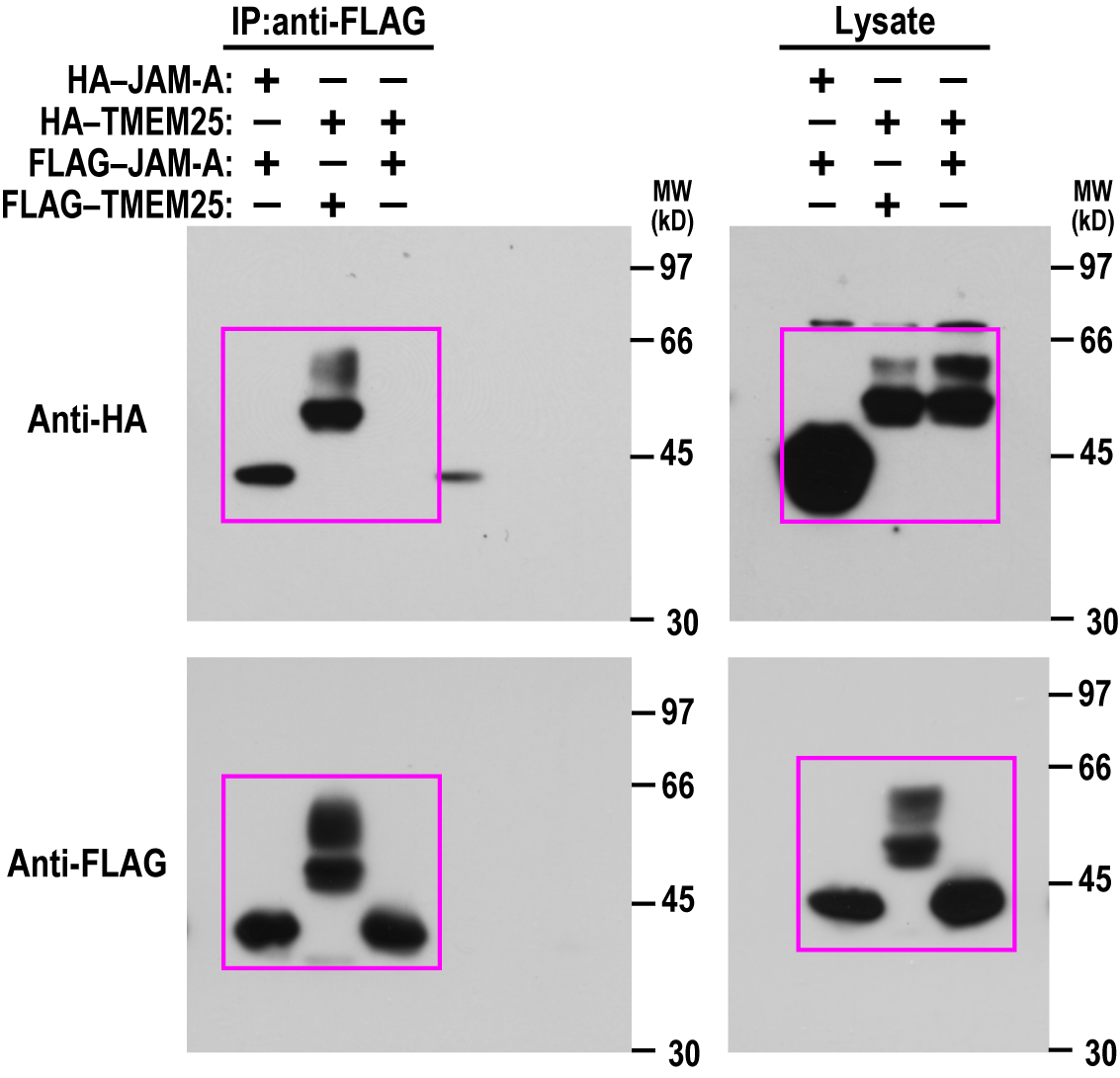

Supplement: Supplementary file 4 — Source Data Fig. 3 [file 44319_2023_18_MOESM4_ESM.zip › Figure_2/2B/2B.tif]

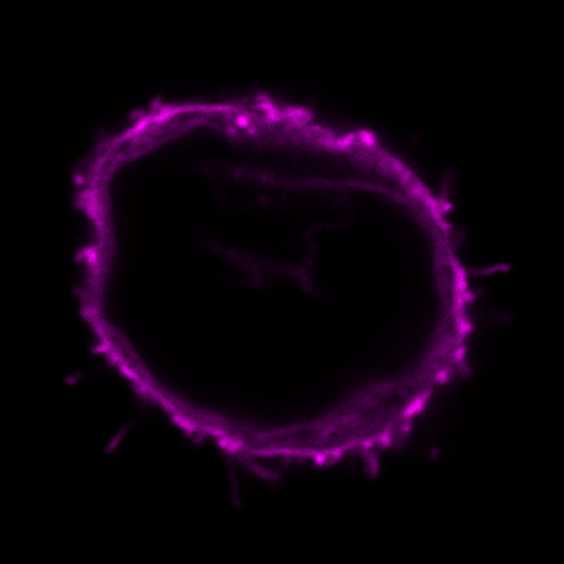

Supplement: Supplementary file 4 — Source Data Fig. 3 [file 44319_2023_18_MOESM4_ESM.zip › Figure_2/2C/2C image data/Right_HA.tif]

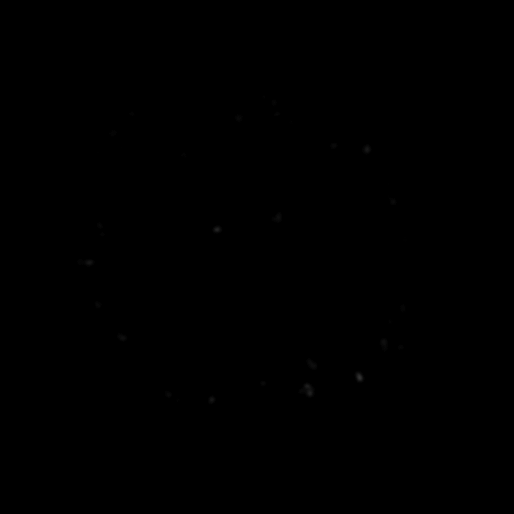

Supplement: Supplementary file 4 — Source Data Fig. 3 [file 44319_2023_18_MOESM4_ESM.zip › Figure_2/2C/2C image data/Right PLA signal.tif]

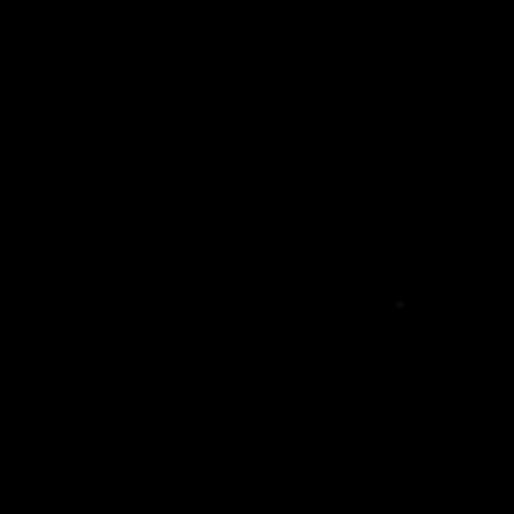

Supplement: Supplementary file 4 — Source Data Fig. 3 [file 44319_2023_18_MOESM4_ESM.zip › Figure_2/2C/2C image data/Left PLA signal.tif]

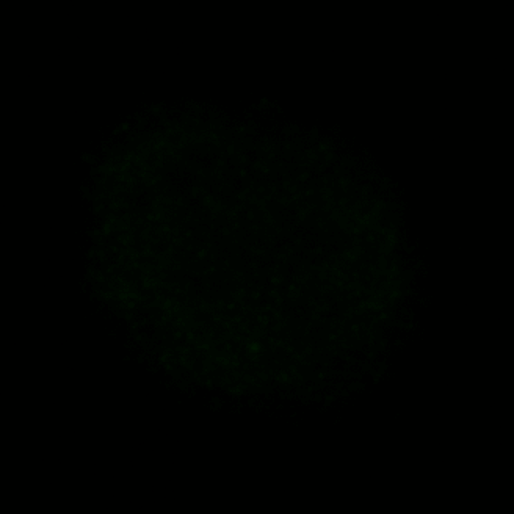

Supplement: Supplementary file 4 — Source Data Fig. 3 [file 44319_2023_18_MOESM4_ESM.zip › Figure_2/2C/2C image data/Right FLAG.tif]

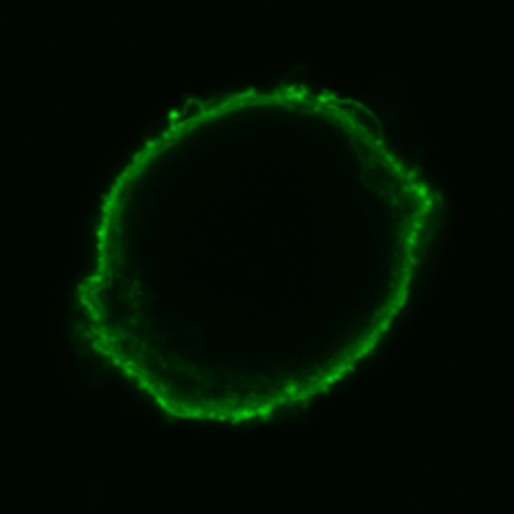

Supplement: Supplementary file 4 — Source Data Fig. 3 [file 44319_2023_18_MOESM4_ESM.zip › Figure_2/2C/2C image data/Left FLAG.tif]

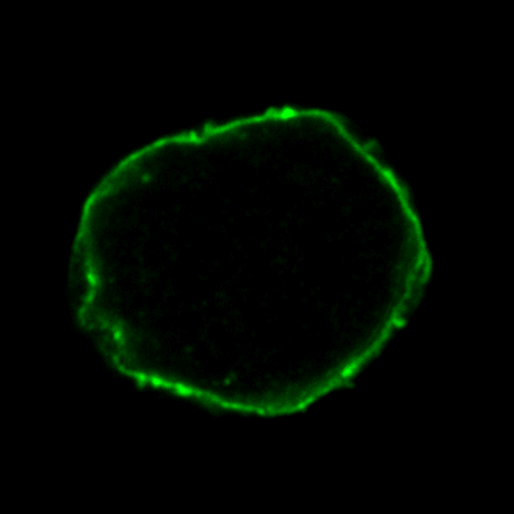

Supplement: Supplementary file 4 — Source Data Fig. 3 [file 44319_2023_18_MOESM4_ESM.zip › Figure_2/2C/2C image data/Middle FLAG.tif]

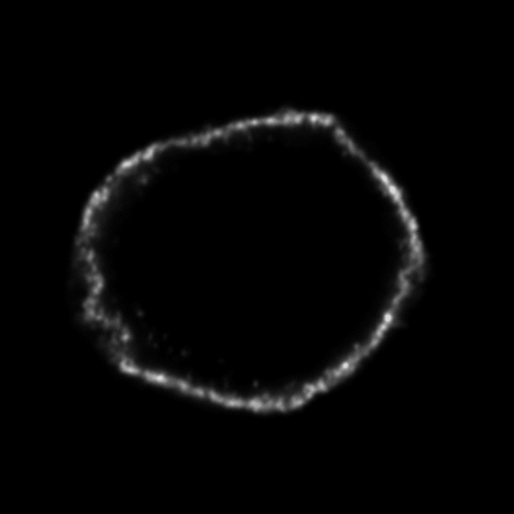

Supplement: Supplementary file 4 — Source Data Fig. 3 [file 44319_2023_18_MOESM4_ESM.zip › Figure_2/2C/2C image data/Middle PLA signal.tif]

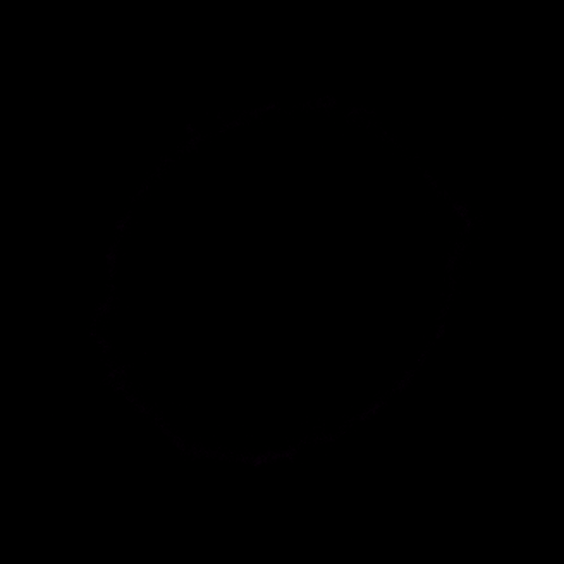

Supplement: Supplementary file 4 — Source Data Fig. 3 [file 44319_2023_18_MOESM4_ESM.zip › Figure_2/2C/2C image data/Left_HA.tif]

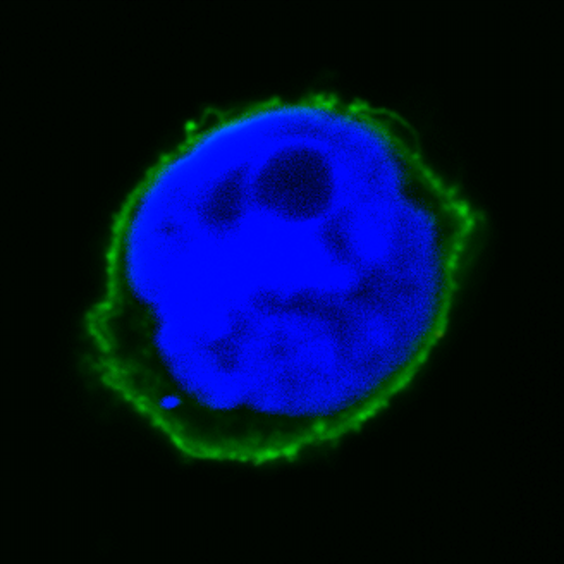

Supplement: Supplementary file 4 — Source Data Fig. 3 [file 44319_2023_18_MOESM4_ESM.zip › Figure_2/2C/2C image data/Left_Merge.tif]

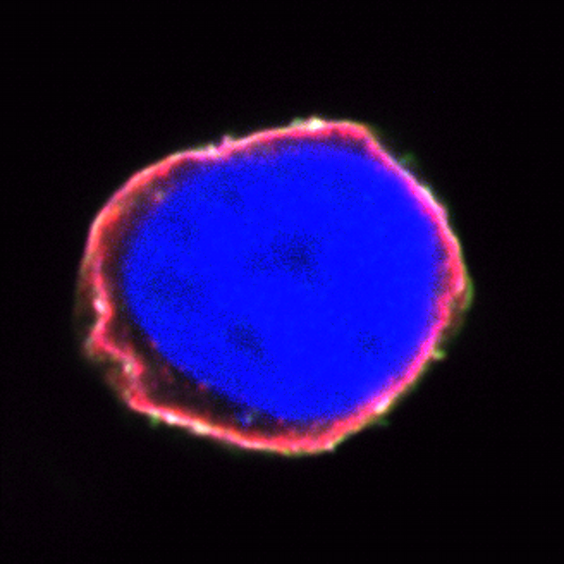

Supplement: Supplementary file 4 — Source Data Fig. 3 [file 44319_2023_18_MOESM4_ESM.zip › Figure_2/2C/2C image data/Middle_Merge.tif]

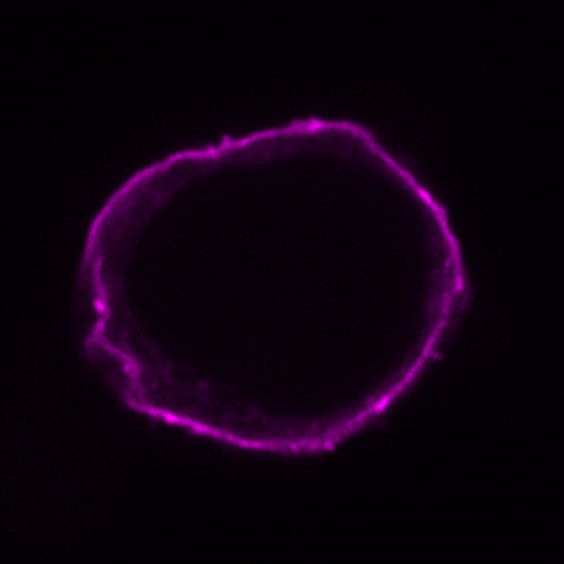

Supplement: Supplementary file 4 — Source Data Fig. 3 [file 44319_2023_18_MOESM4_ESM.zip › Figure_2/2C/2C image data/Middle_HA.tif]

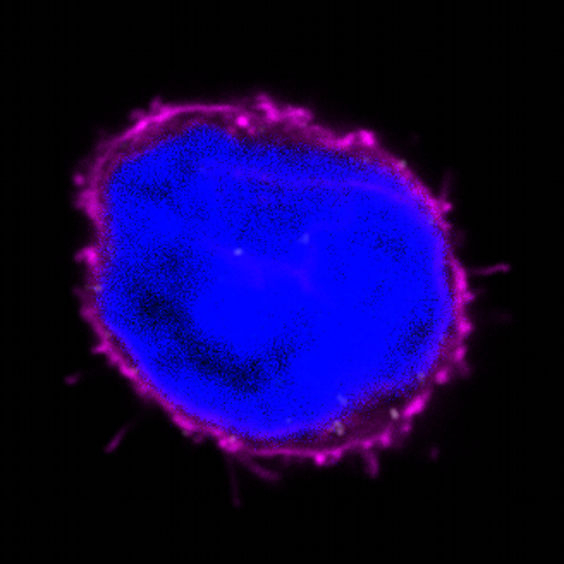

Supplement: Supplementary file 4 — Source Data Fig. 3 [file 44319_2023_18_MOESM4_ESM.zip › Figure_2/2C/2C image data/Right_Merge.tif]

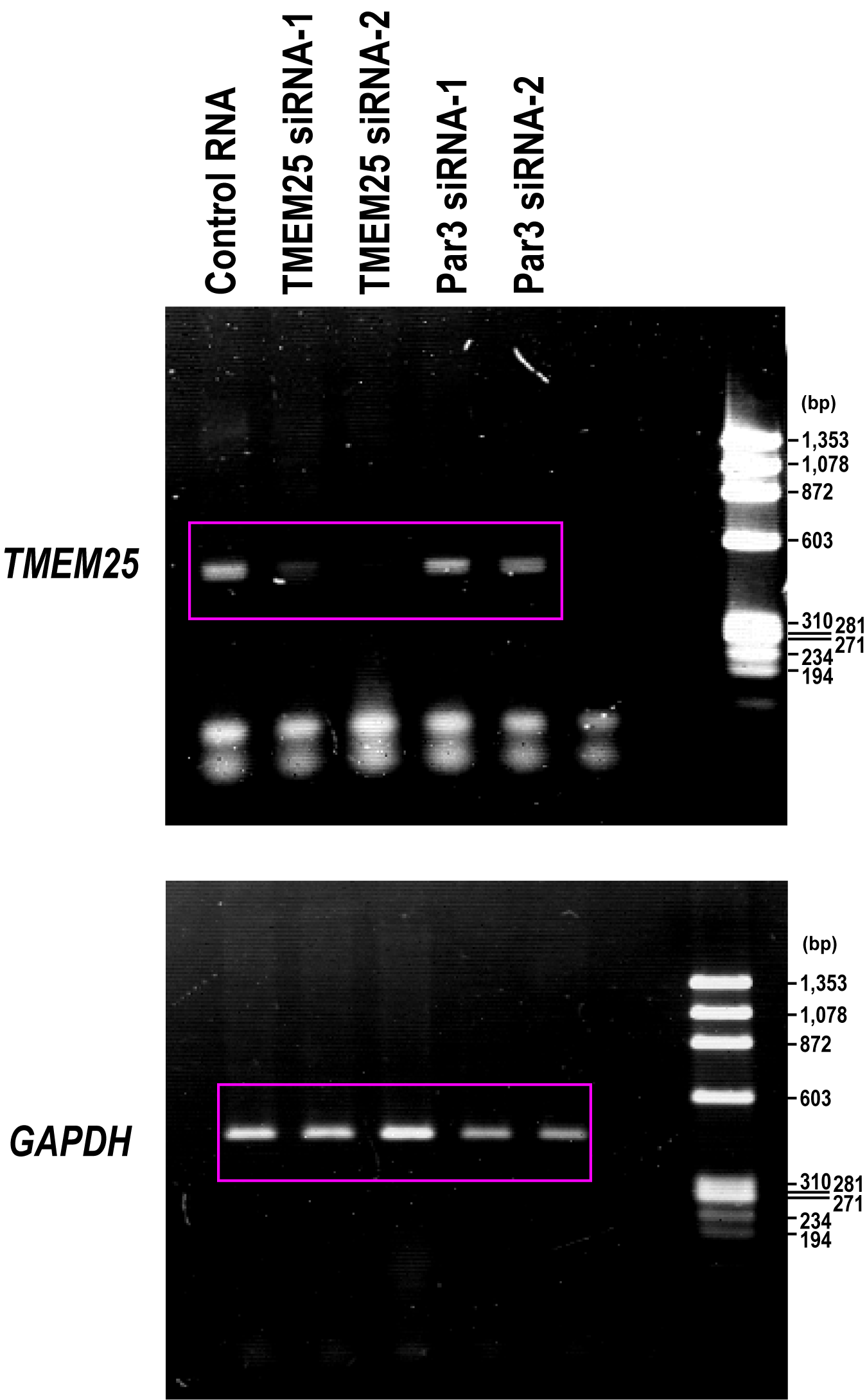

Supplement: Supplementary file 5 — Source Data Fig. 4 [file 44319_2023_18_MOESM5_ESM.zip › Figure_3/3A/3A_RT-PCR.tif]

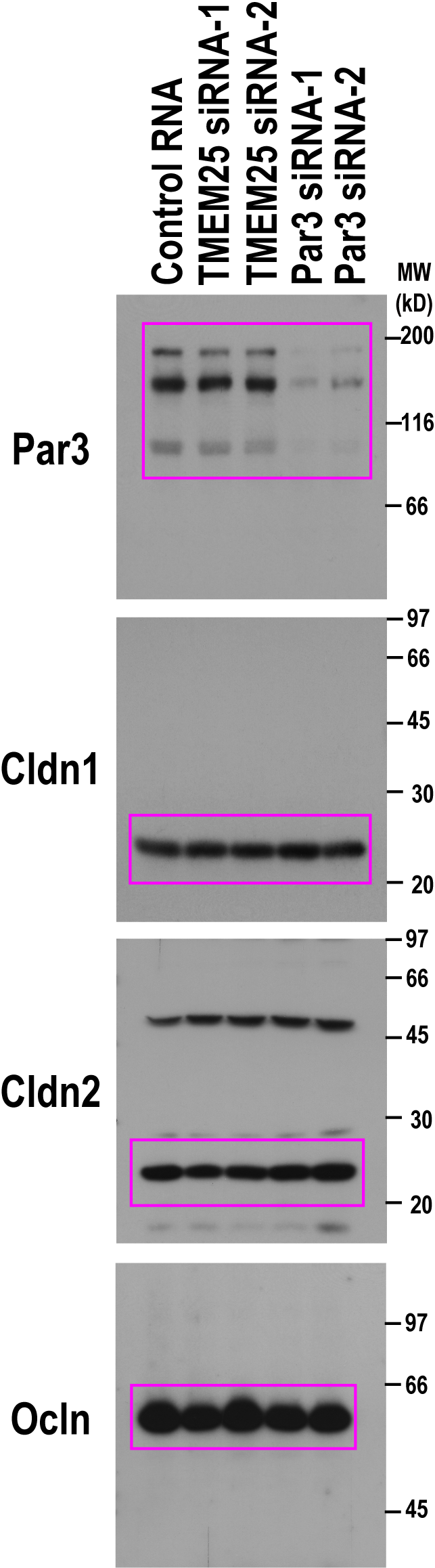

Supplement: Supplementary file 5 — Source Data Fig. 4 [file 44319_2023_18_MOESM5_ESM.zip › Figure_3/3A/3A_immunoblot.tif]

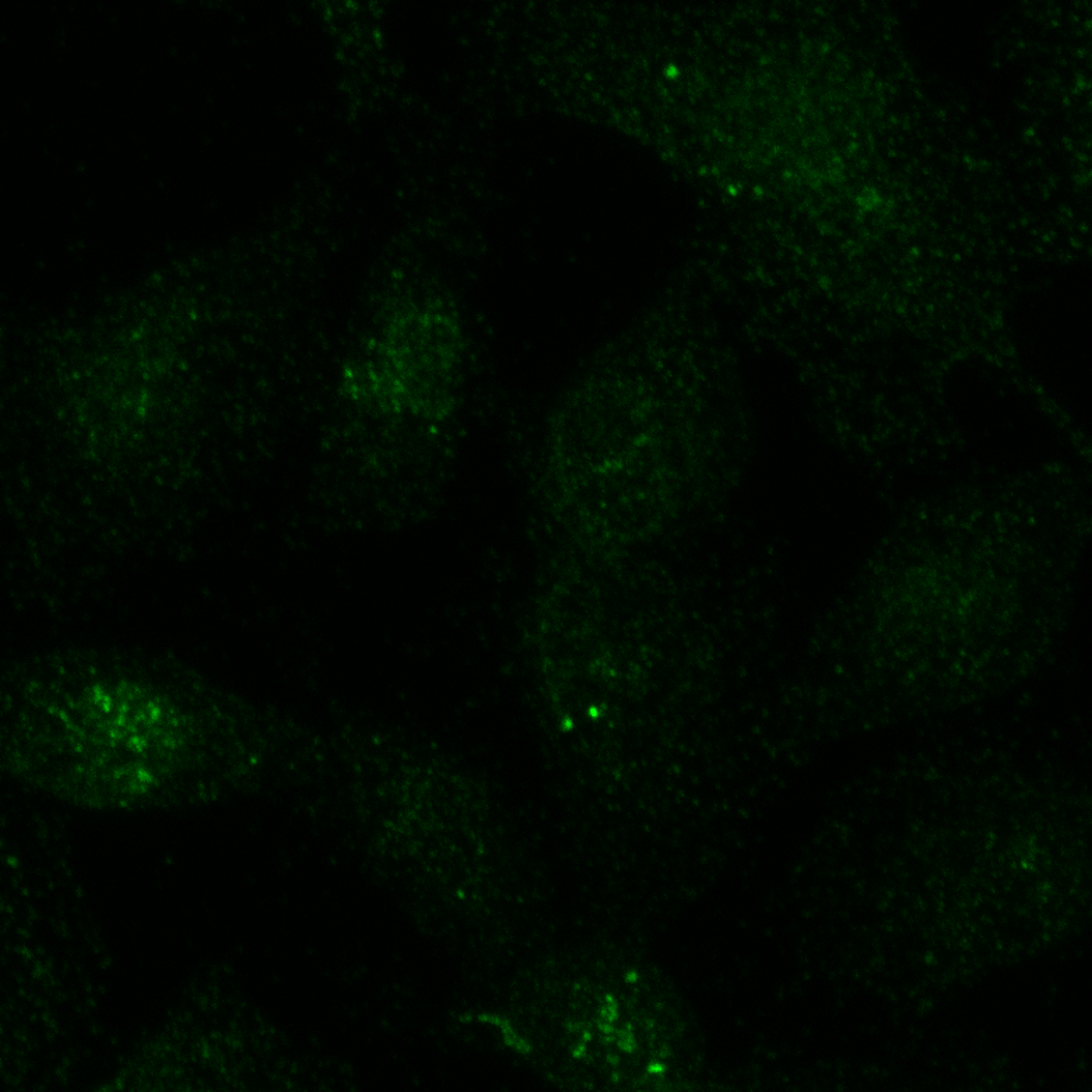

Supplement: Supplementary file 5 — Source Data Fig. 4 [file 44319_2023_18_MOESM5_ESM.zip › Figure_3/3E/3E image data/0h_right_Cldn2.tif]

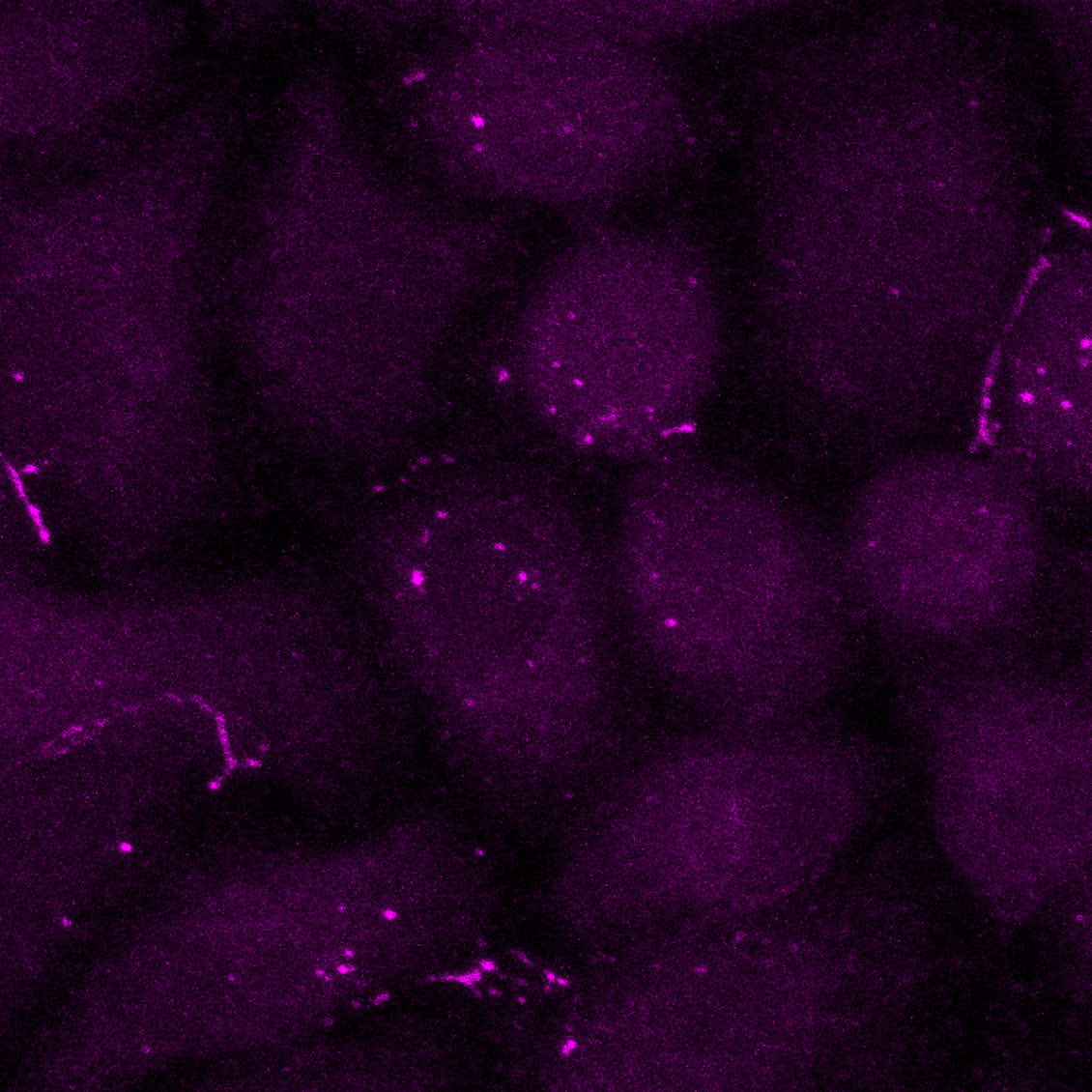

Supplement: Supplementary file 5 — Source Data Fig. 4 [file 44319_2023_18_MOESM5_ESM.zip › Figure_3/3E/3E image data/0h_left_Ocln.tif]

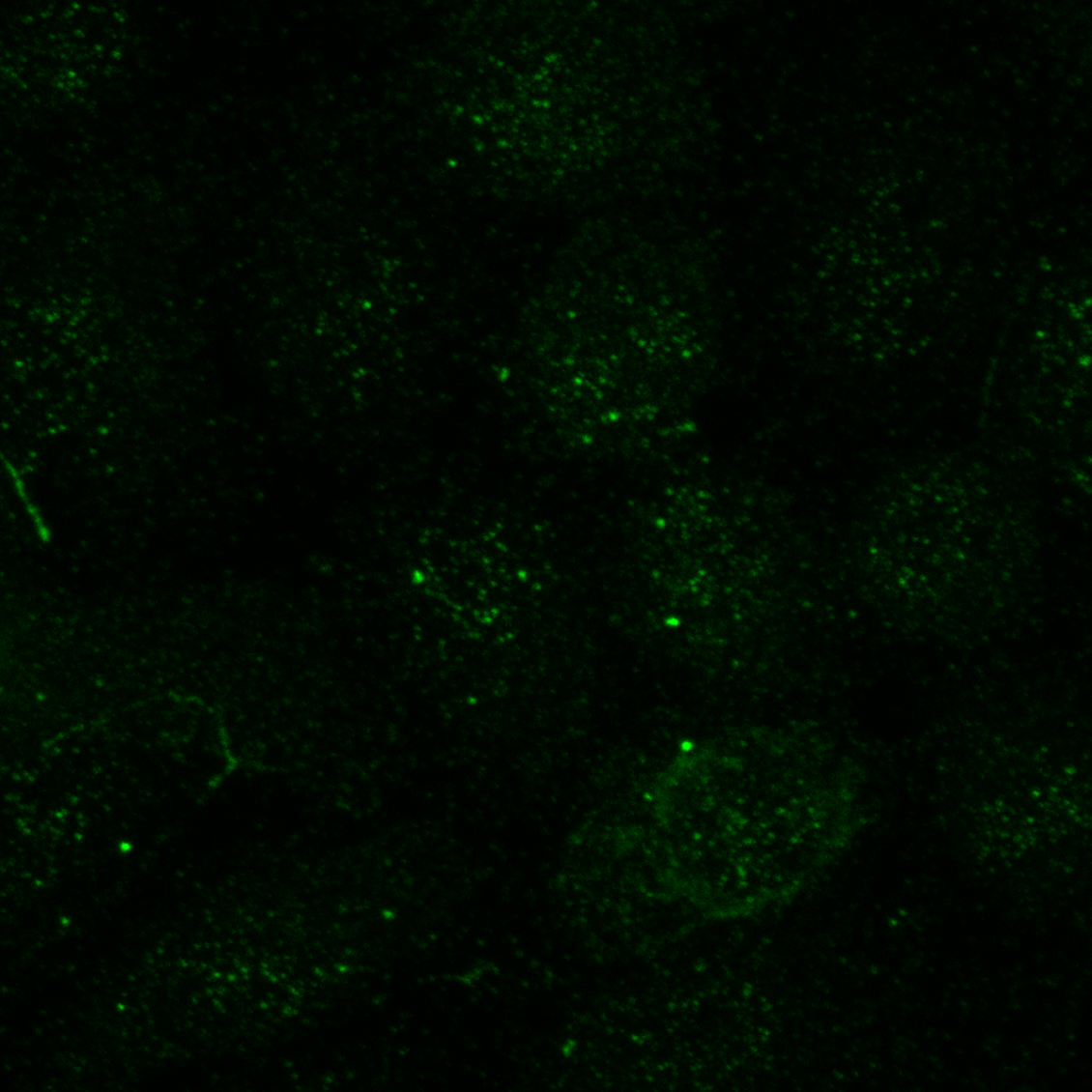

Supplement: Supplementary file 5 — Source Data Fig. 4 [file 44319_2023_18_MOESM5_ESM.zip › Figure_3/3E/3E image data/0h_left_Cldn2.tif]

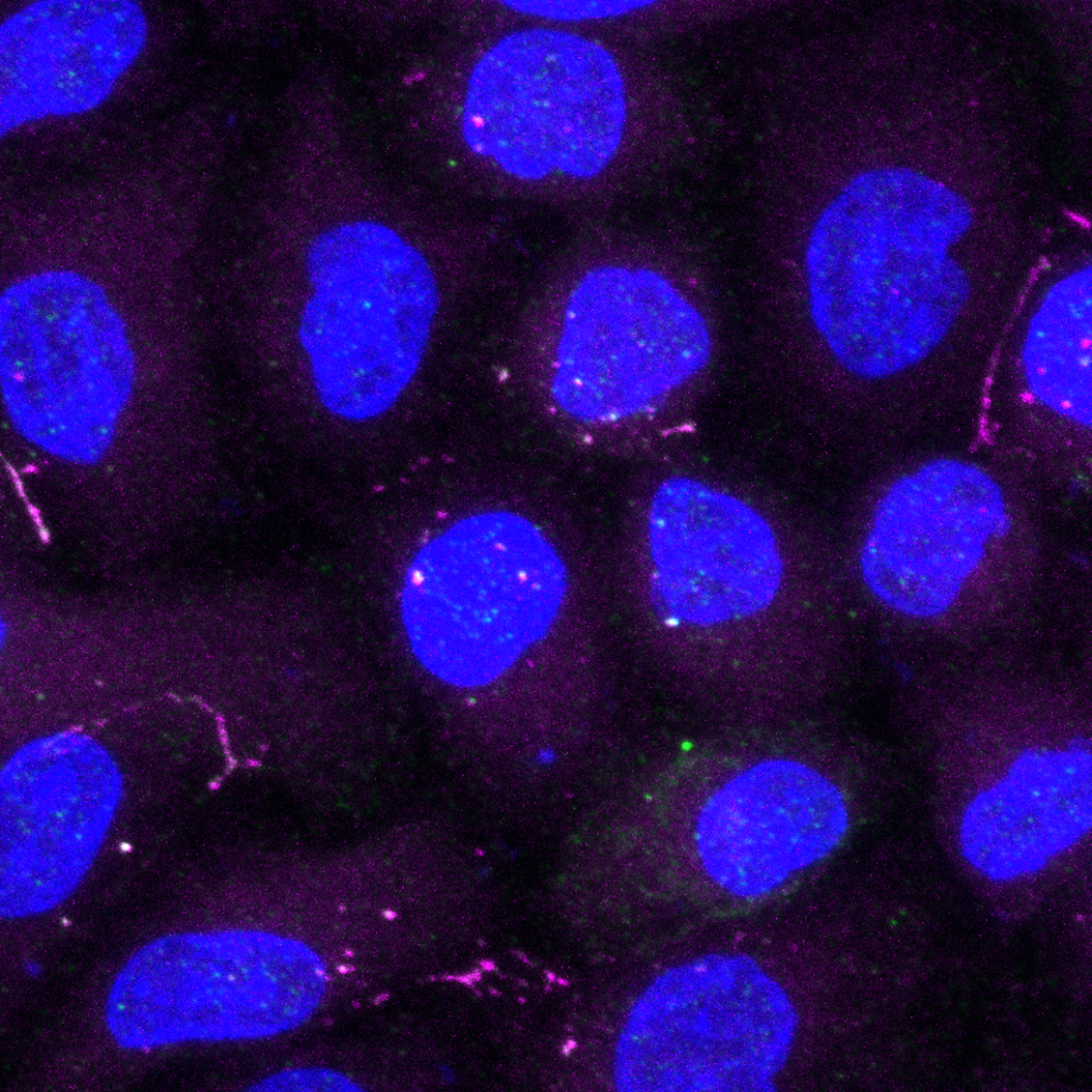

Supplement: Supplementary file 5 — Source Data Fig. 4 [file 44319_2023_18_MOESM5_ESM.zip › Figure_3/3E/3E image data/0h_left_merge.tif]

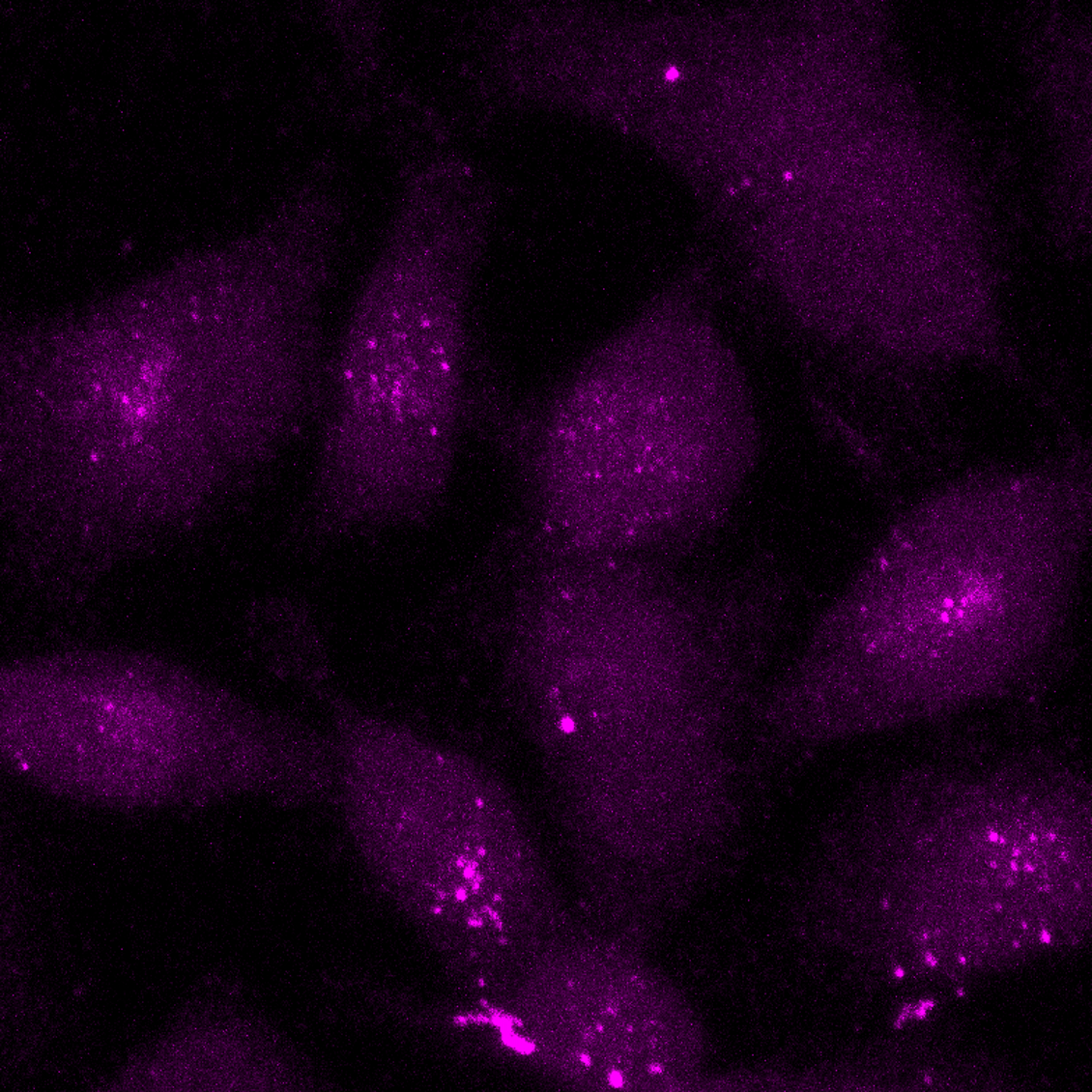

Supplement: Supplementary file 5 — Source Data Fig. 4 [file 44319_2023_18_MOESM5_ESM.zip › Figure_3/3E/3E image data/0h_right_Ocln.tif]

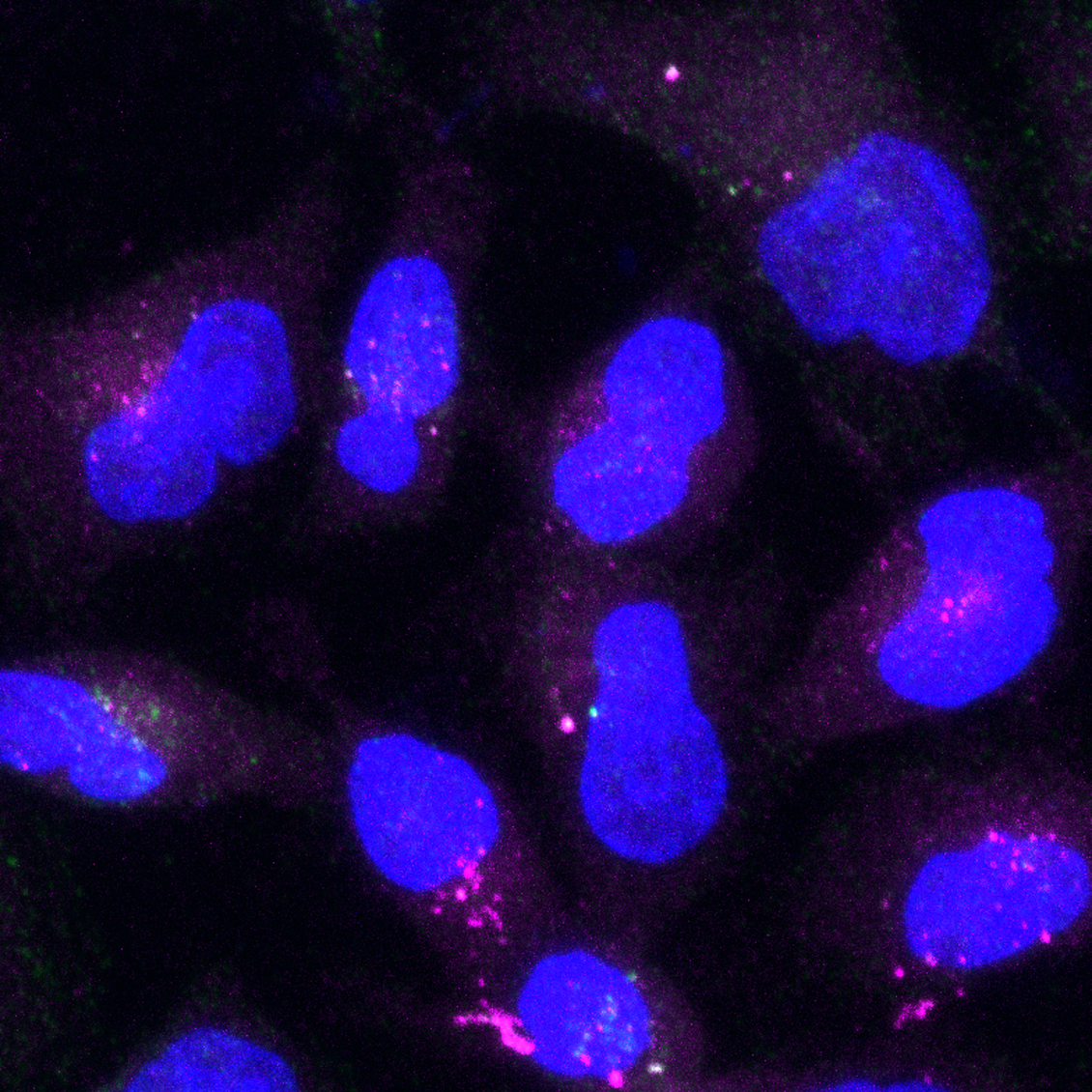

Supplement: Supplementary file 5 — Source Data Fig. 4 [file 44319_2023_18_MOESM5_ESM.zip › Figure_3/3E/3E image data/0h_right_merge.tif]

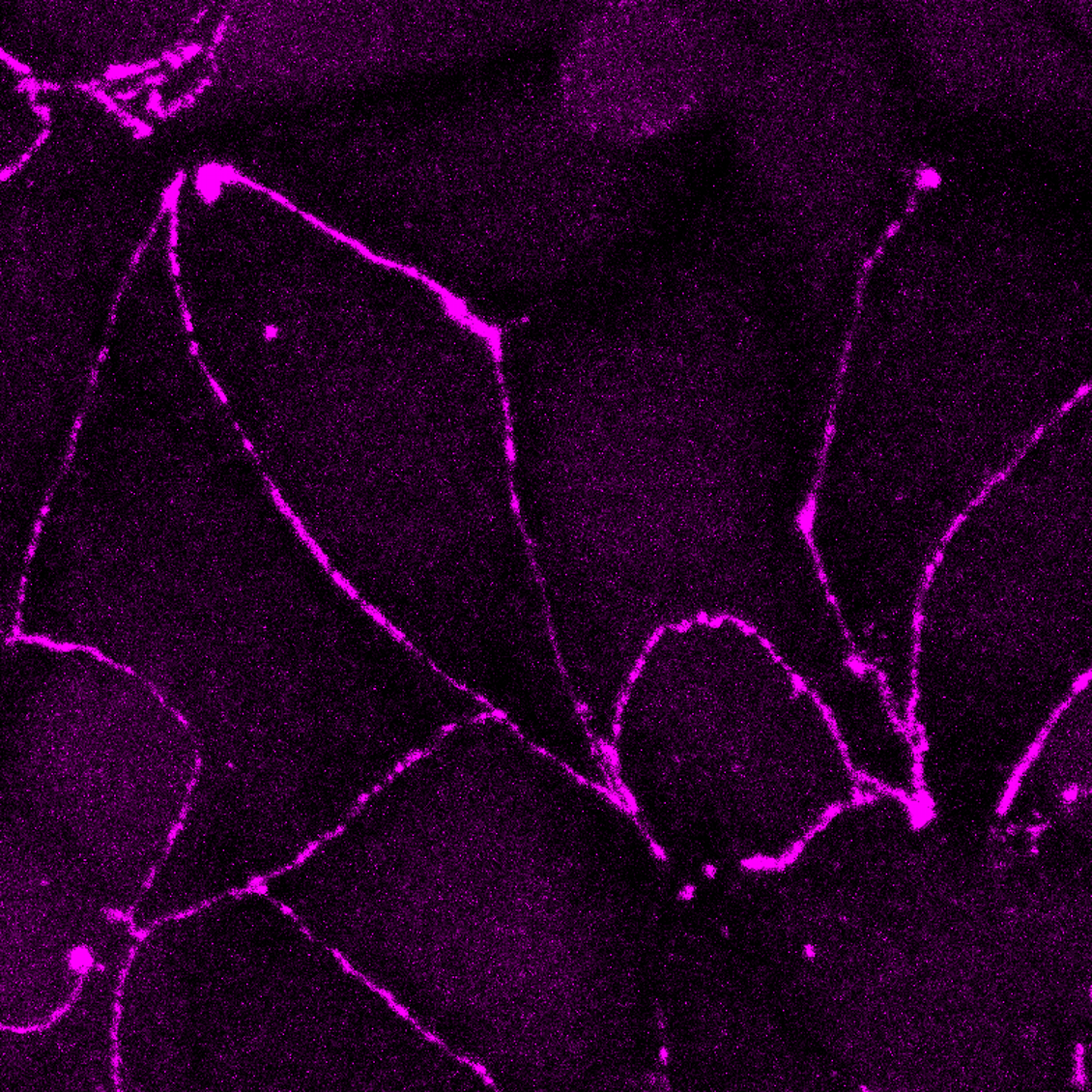

Supplement: Supplementary file 5 — Source Data Fig. 4 [file 44319_2023_18_MOESM5_ESM.zip › Figure_3/3E/3E image data/2h_left_Ocln.tif]

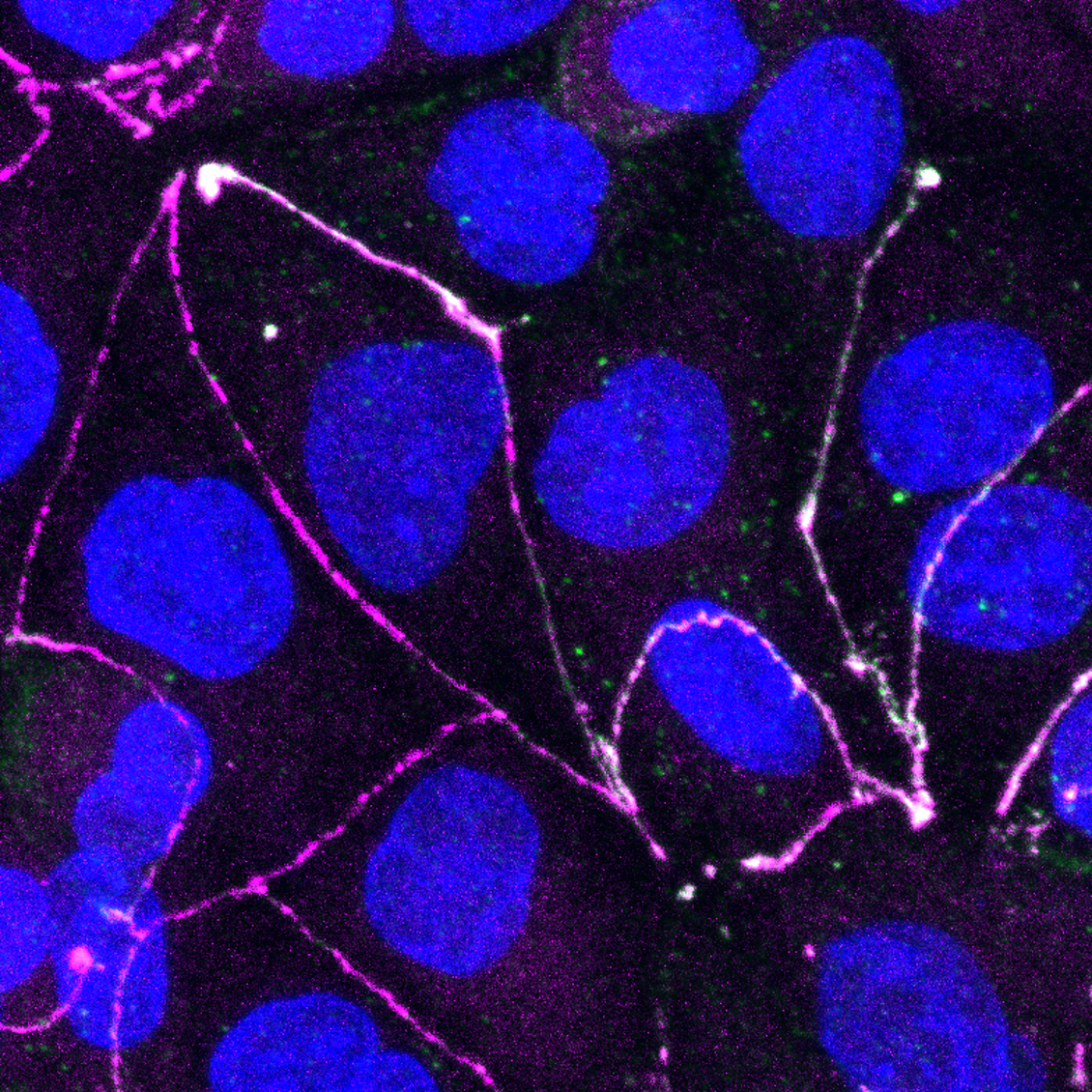

Supplement: Supplementary file 5 — Source Data Fig. 4 [file 44319_2023_18_MOESM5_ESM.zip › Figure_3/3E/3E image data/2h_left_merge.tif]

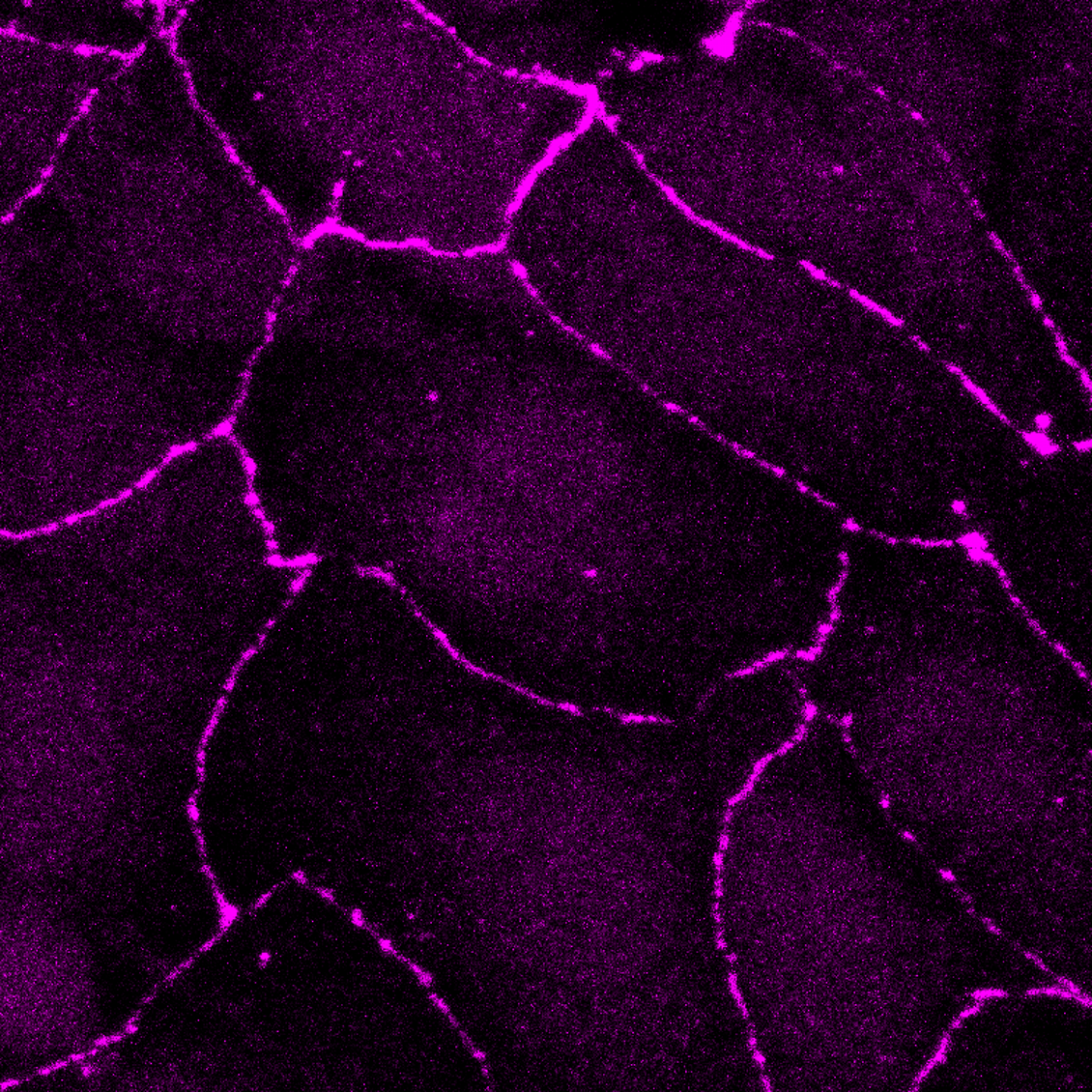

Supplement: Supplementary file 5 — Source Data Fig. 4 [file 44319_2023_18_MOESM5_ESM.zip › Figure_3/3E/3E image data/2h_right_Ocln.tif]

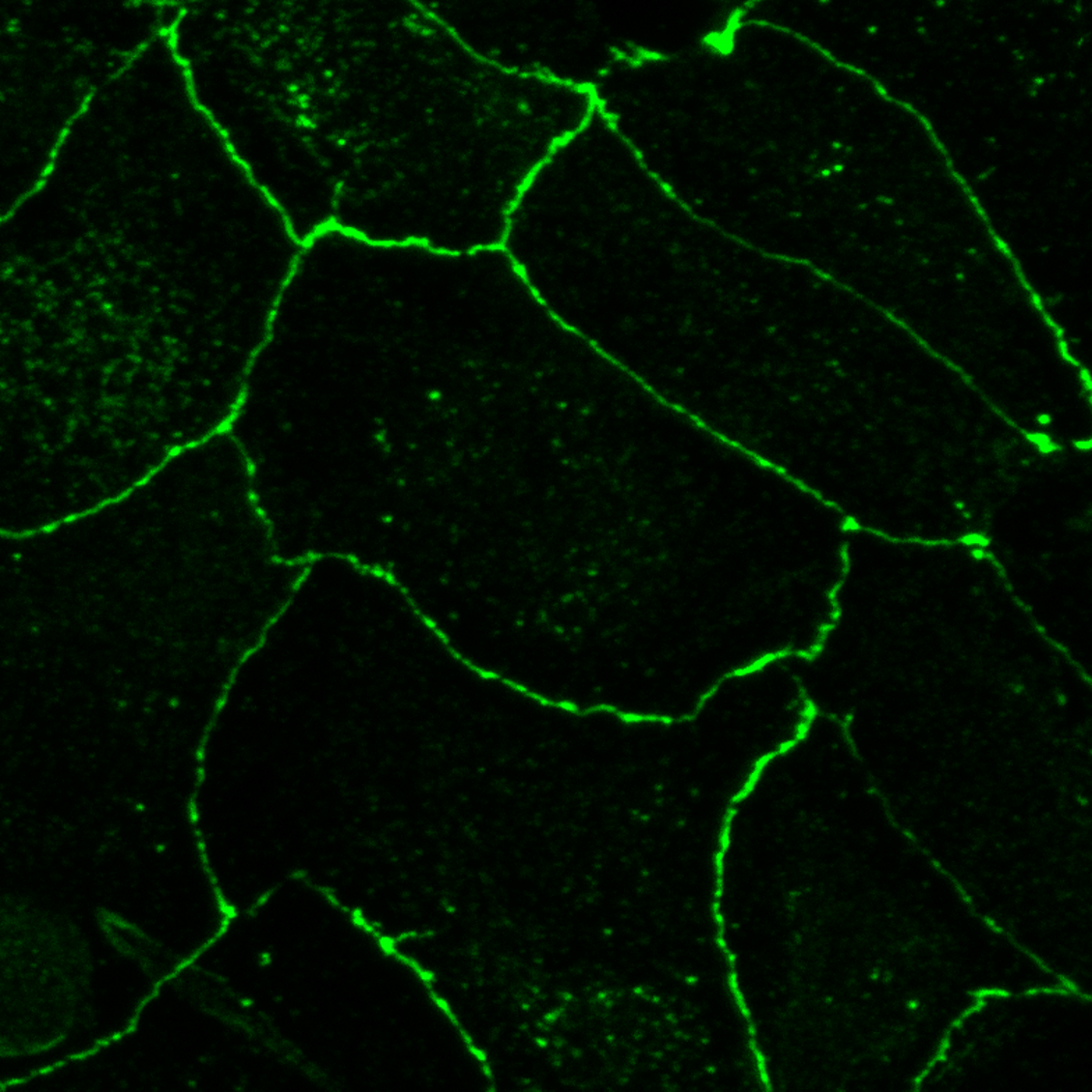

Supplement: Supplementary file 5 — Source Data Fig. 4 [file 44319_2023_18_MOESM5_ESM.zip › Figure_3/3E/3E image data/2h_right_Cldn2.tif]

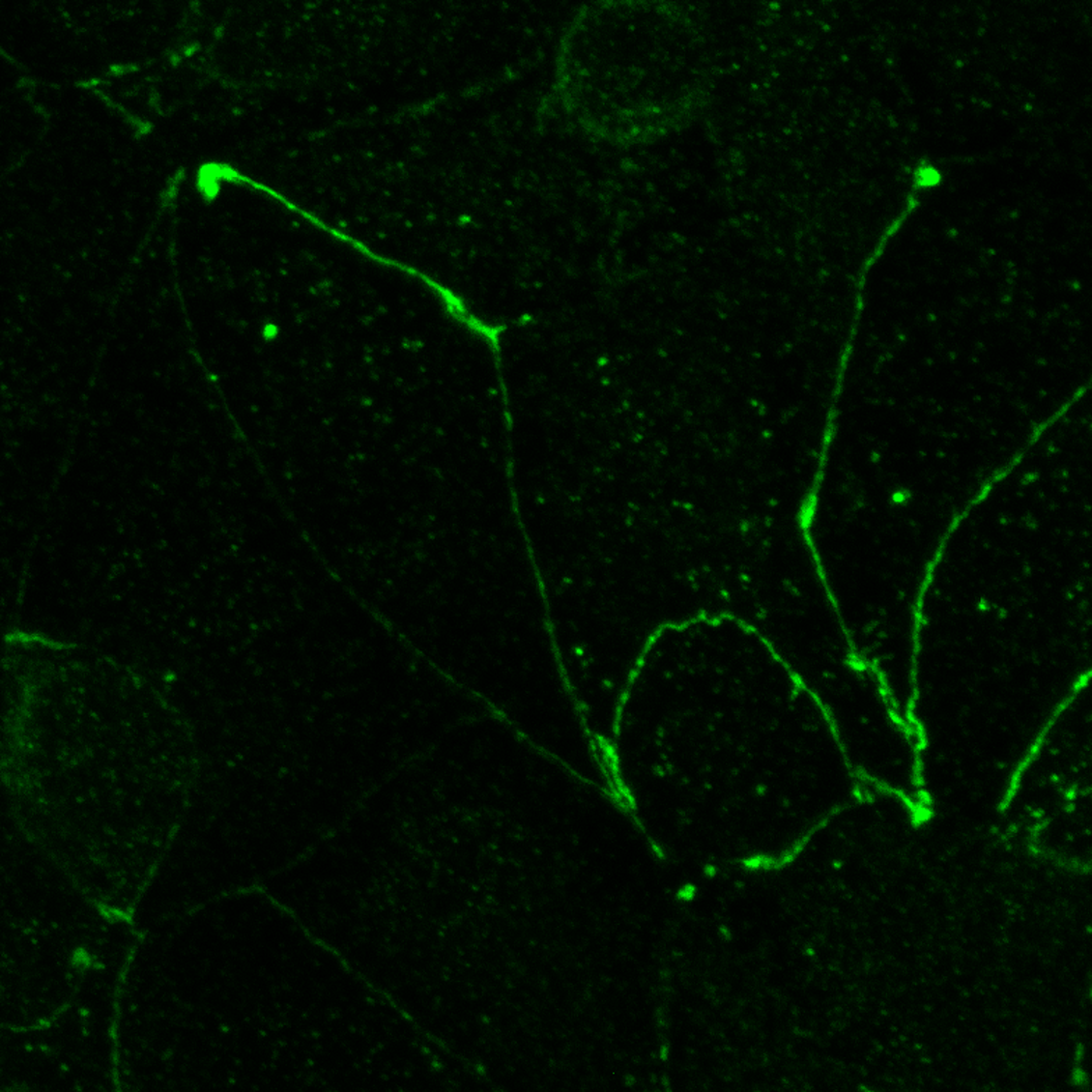

Supplement: Supplementary file 5 — Source Data Fig. 4 [file 44319_2023_18_MOESM5_ESM.zip › Figure_3/3E/3E image data/2h_left_Cldn2.tif]

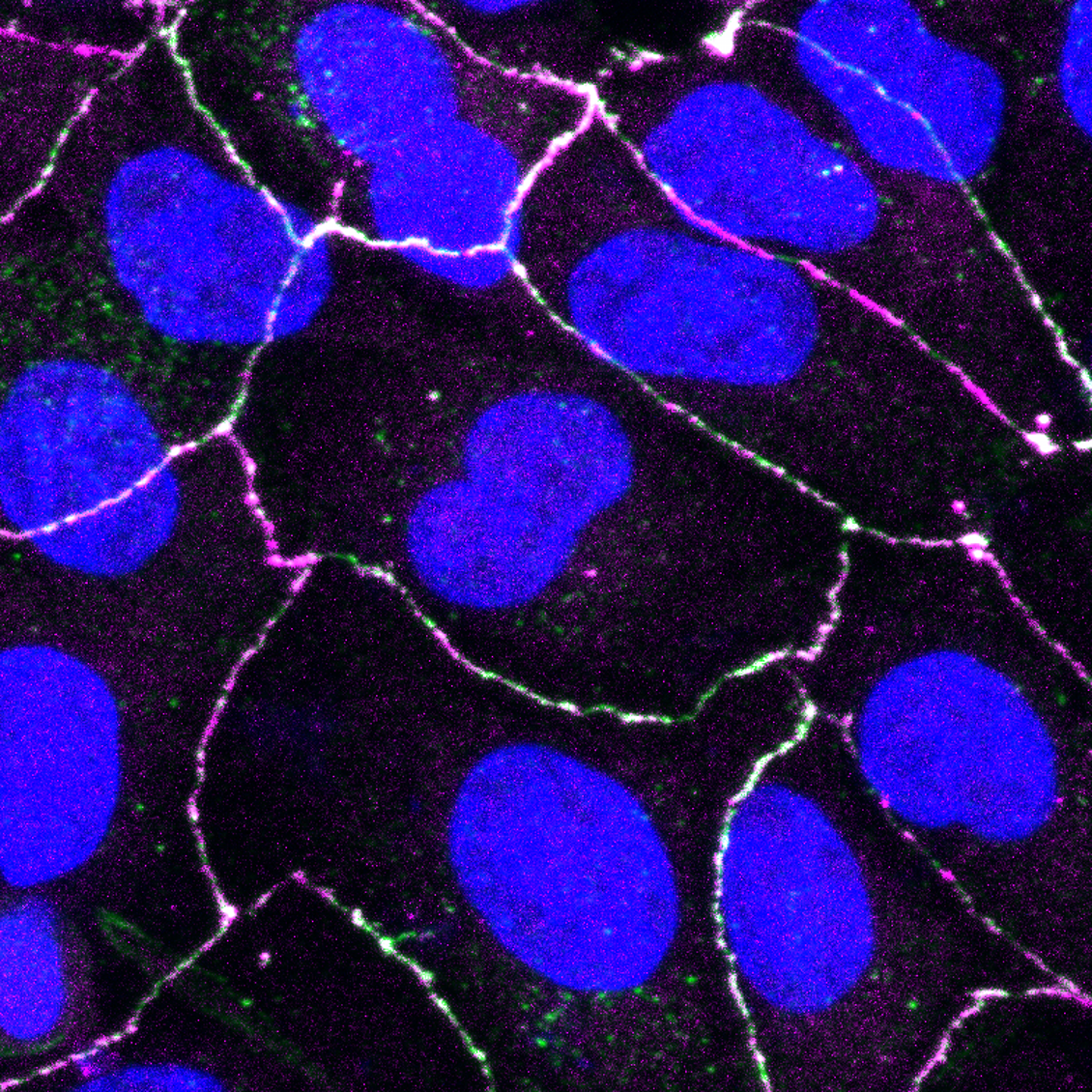

Supplement: Supplementary file 5 — Source Data Fig. 4 [file 44319_2023_18_MOESM5_ESM.zip › Figure_3/3E/3E image data/2h_right_merge.tif]

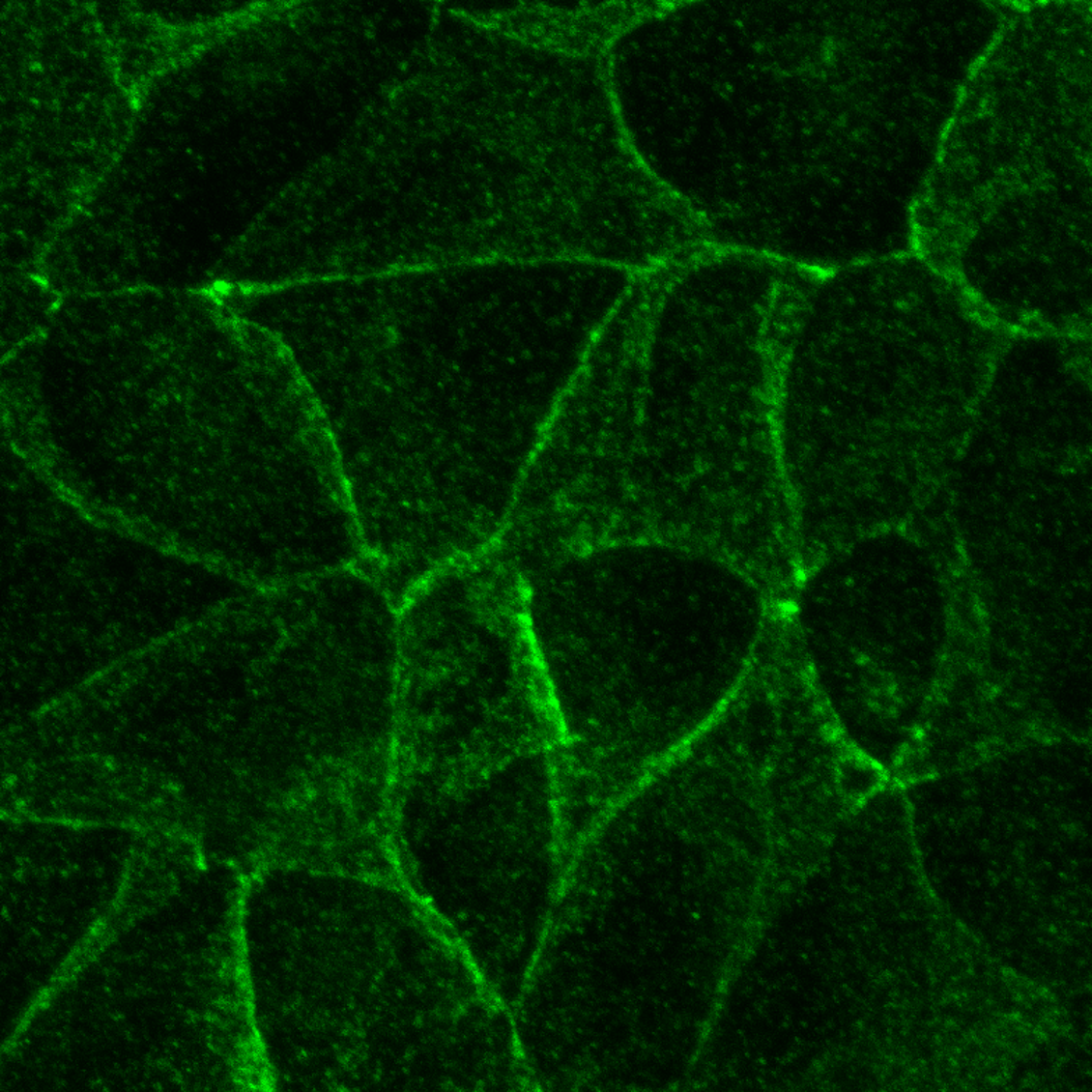

Supplement: Supplementary file 5 — Source Data Fig. 4 [file 44319_2023_18_MOESM5_ESM.zip › Figure_3/3C/3C xy images/2h_middle_Ecad.tif]

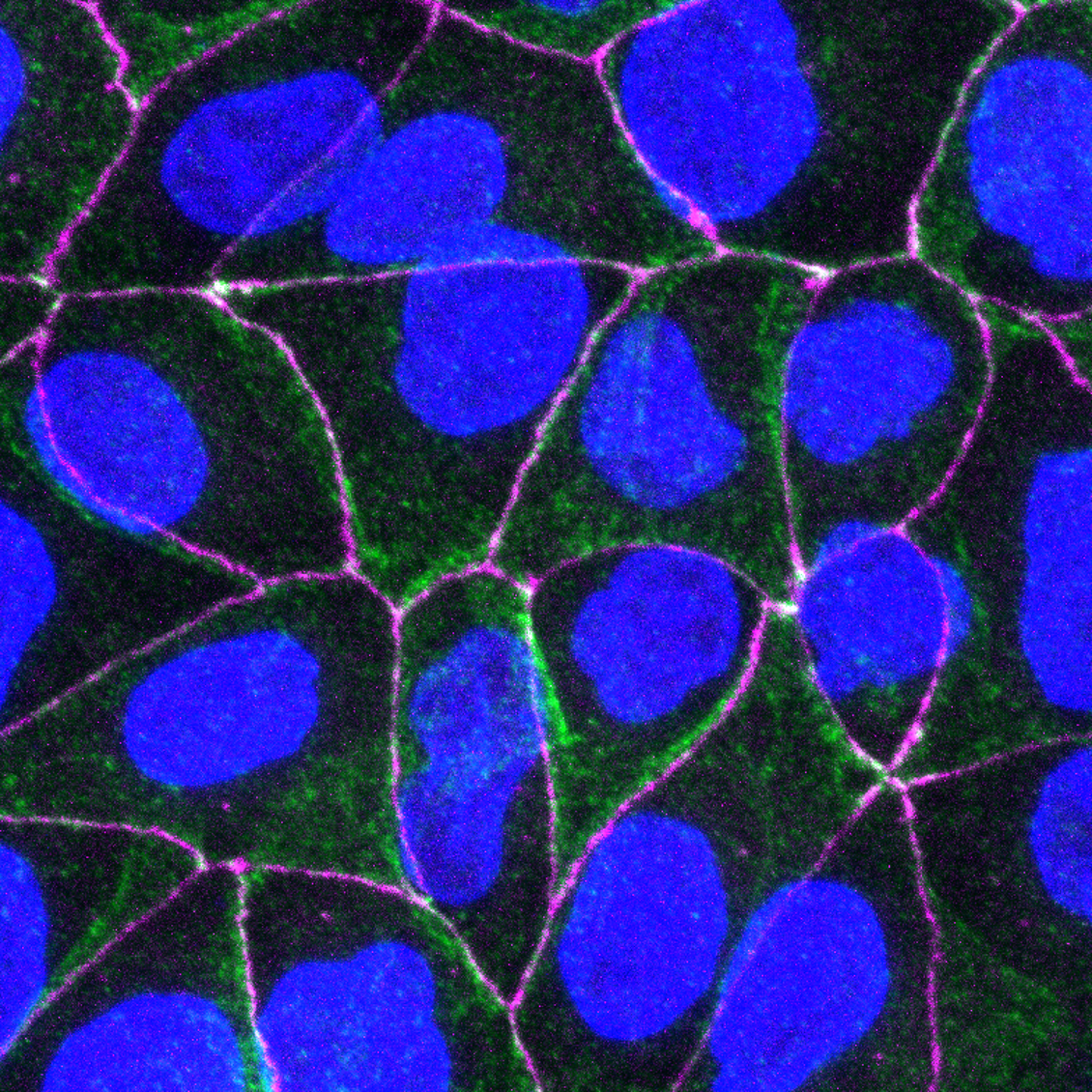

Supplement: Supplementary file 5 — Source Data Fig. 4 [file 44319_2023_18_MOESM5_ESM.zip › Figure_3/3C/3C xy images/2h_middle_merge.tif]

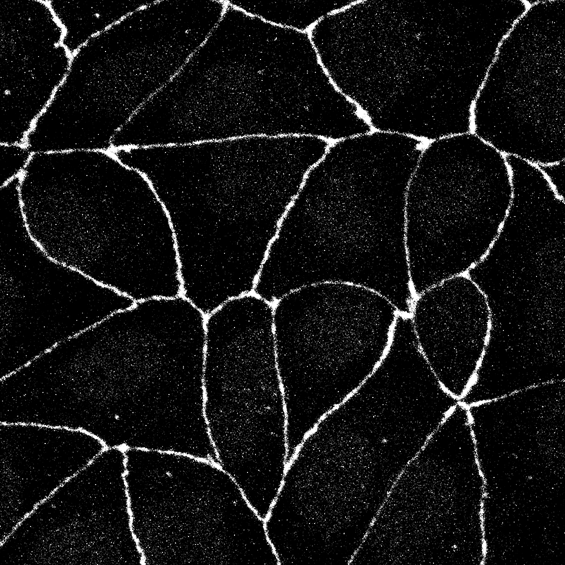

Supplement: Supplementary file 5 — Source Data Fig. 4 [file 44319_2023_18_MOESM5_ESM.zip › Figure_3/3C/3C xy images/2h_middle_ZO1.tif]

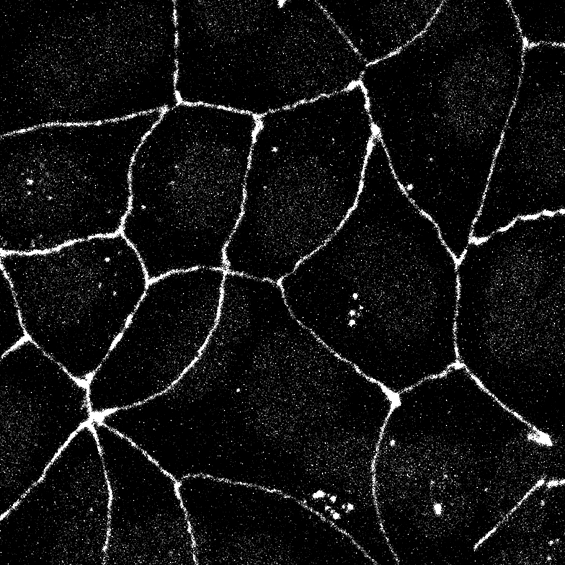

Supplement: Supplementary file 5 — Source Data Fig. 4 [file 44319_2023_18_MOESM5_ESM.zip › Figure_3/3C/3C xy images/2h_right_ZO1.tif]

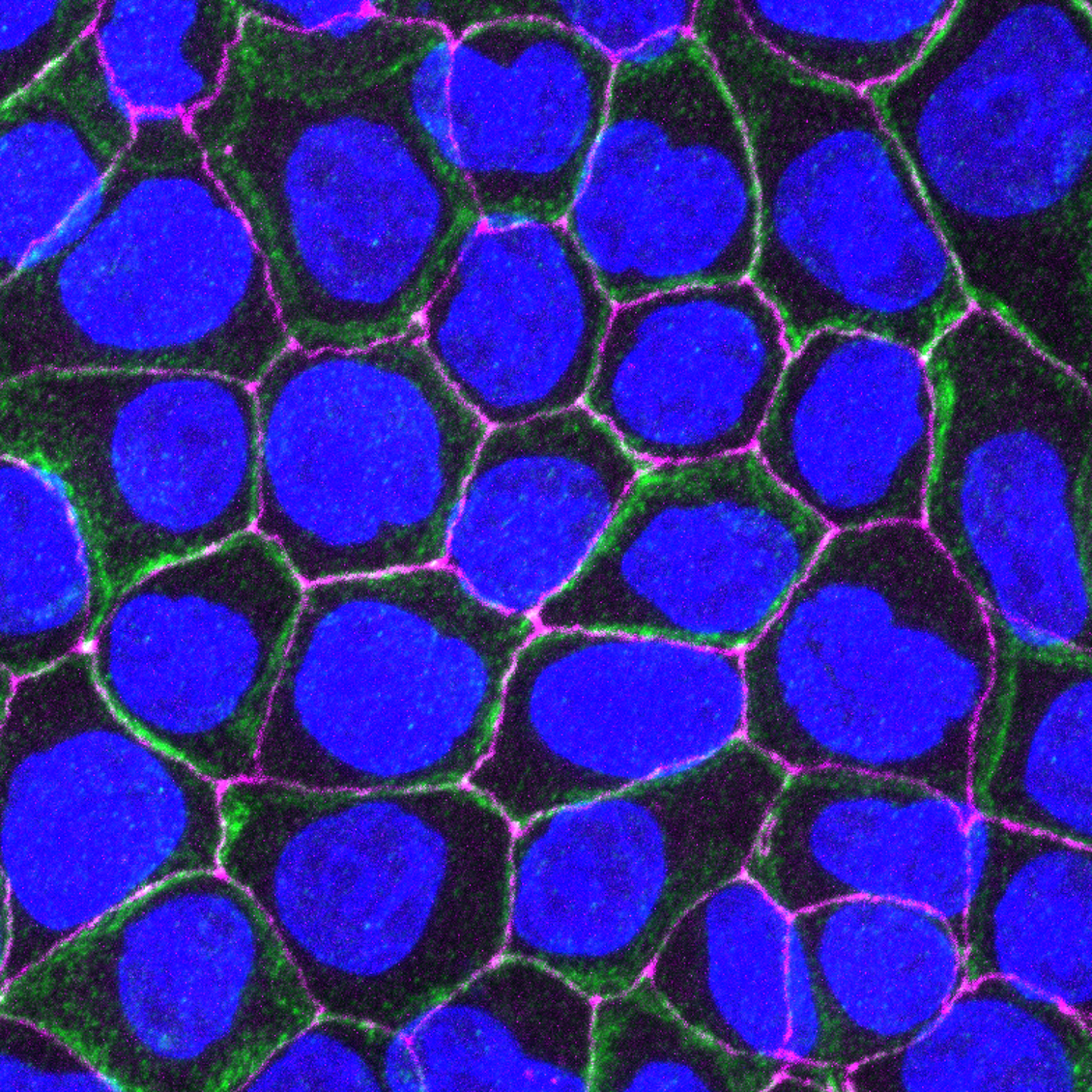

Supplement: Supplementary file 5 — Source Data Fig. 4 [file 44319_2023_18_MOESM5_ESM.zip › Figure_3/3C/3C xy images/6h_left_merge.tif]

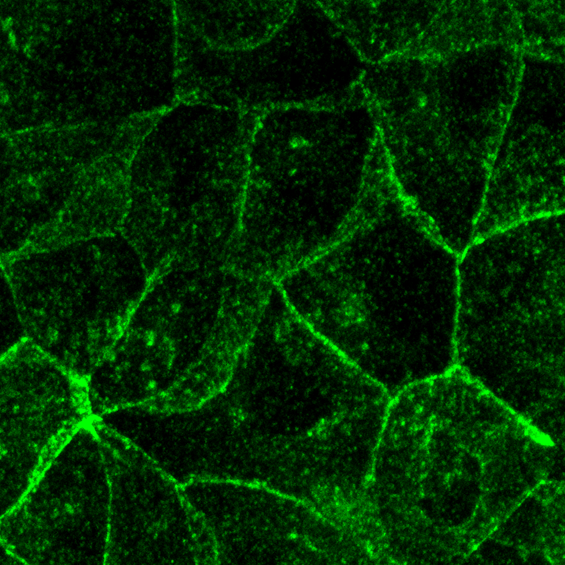

Supplement: Supplementary file 5 — Source Data Fig. 4 [file 44319_2023_18_MOESM5_ESM.zip › Figure_3/3C/3C xy images/2h_right_Ecad.tif]

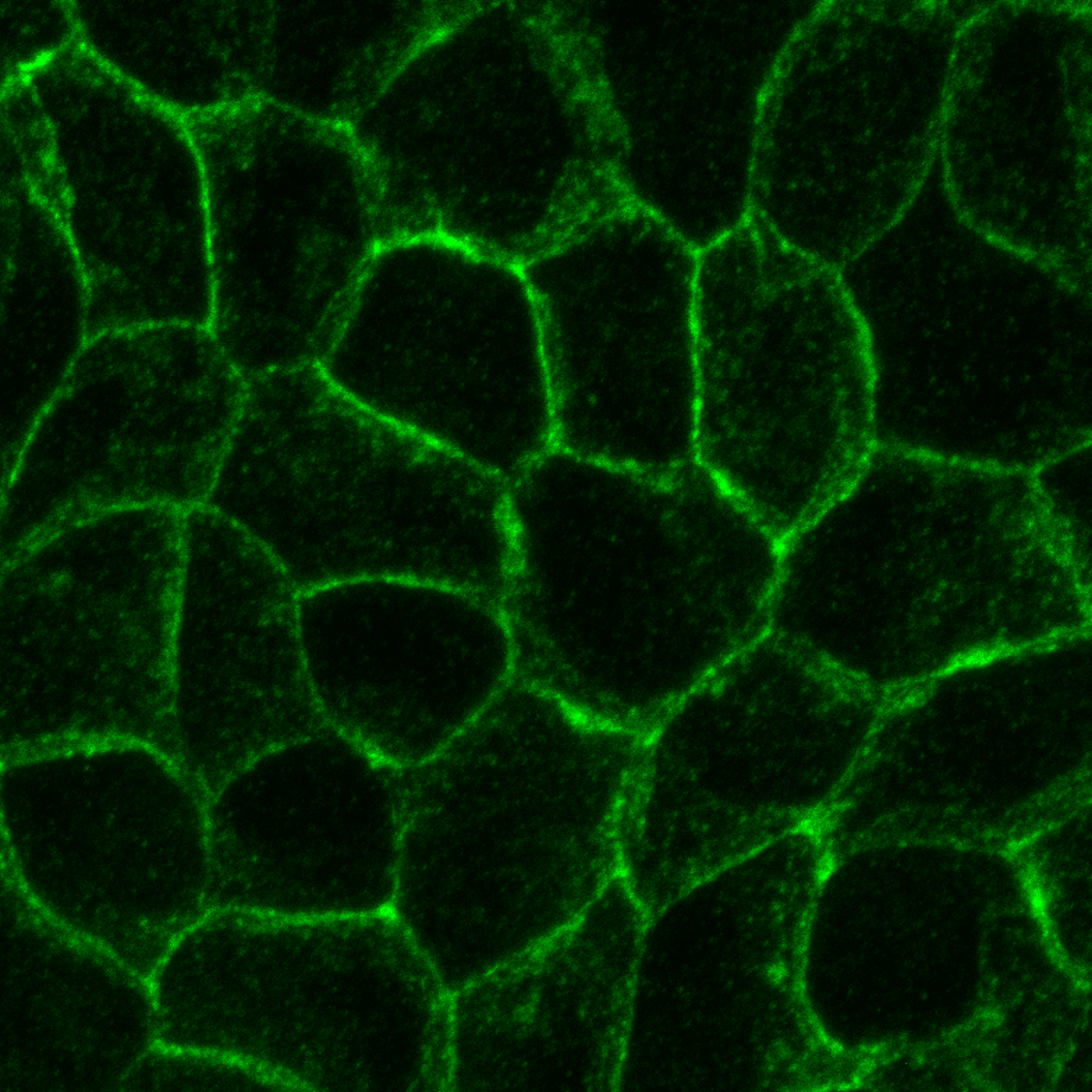

Supplement: Supplementary file 5 — Source Data Fig. 4 [file 44319_2023_18_MOESM5_ESM.zip › Figure_3/3C/3C xy images/6h_middle_Ecad.tif]

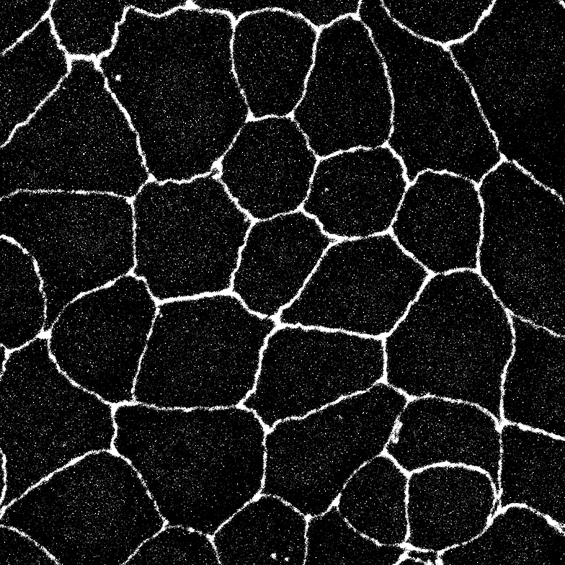

Supplement: Supplementary file 5 — Source Data Fig. 4 [file 44319_2023_18_MOESM5_ESM.zip › Figure_3/3C/3C xy images/6h_left_ZO1.tif]

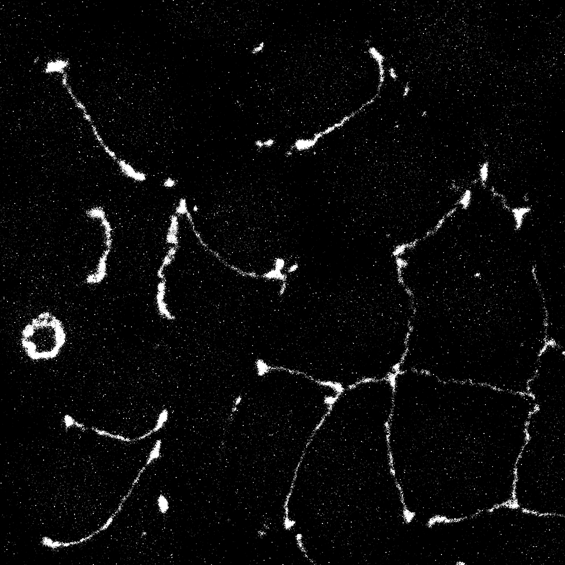

Supplement: Supplementary file 5 — Source Data Fig. 4 [file 44319_2023_18_MOESM5_ESM.zip › Figure_3/3C/3C xy images/2h_left_ZO1.tif]

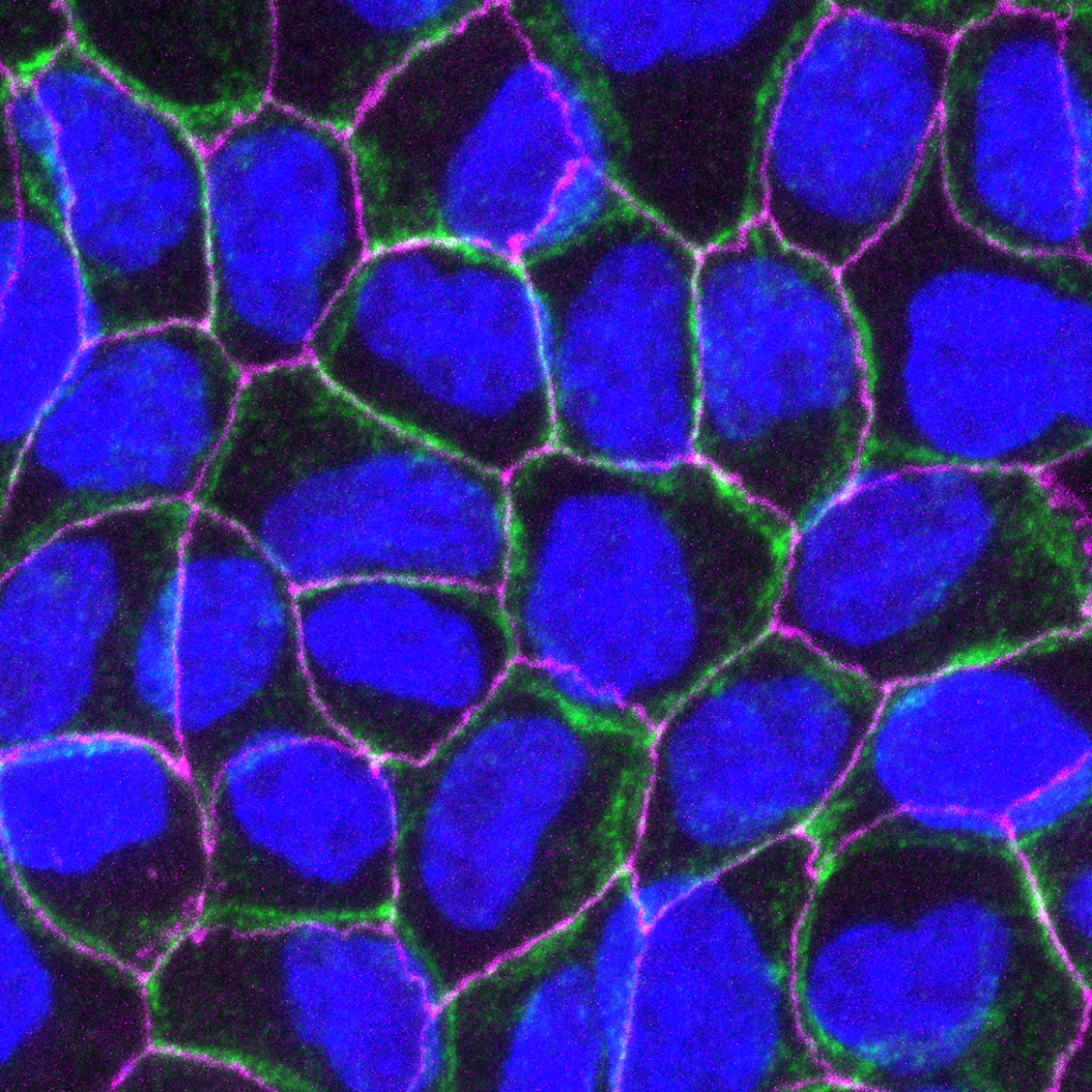

Supplement: Supplementary file 5 — Source Data Fig. 4 [file 44319_2023_18_MOESM5_ESM.zip › Figure_3/3C/3C xy images/6h_middle_merge.tif]

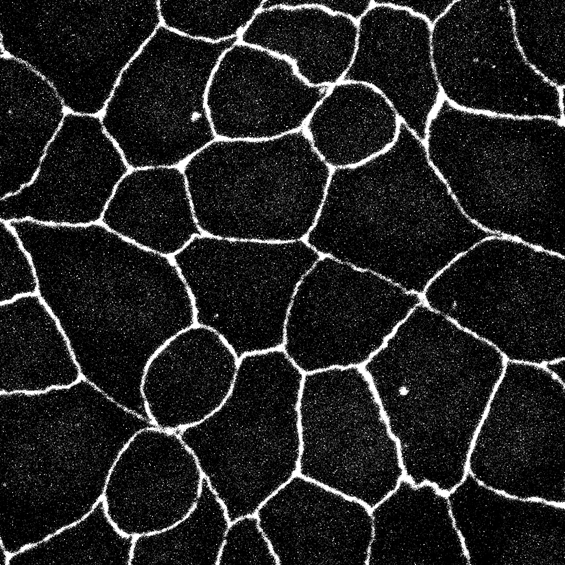

Supplement: Supplementary file 5 — Source Data Fig. 4 [file 44319_2023_18_MOESM5_ESM.zip › Figure_3/3C/3C xy images/6h_right_ZO1.tif]

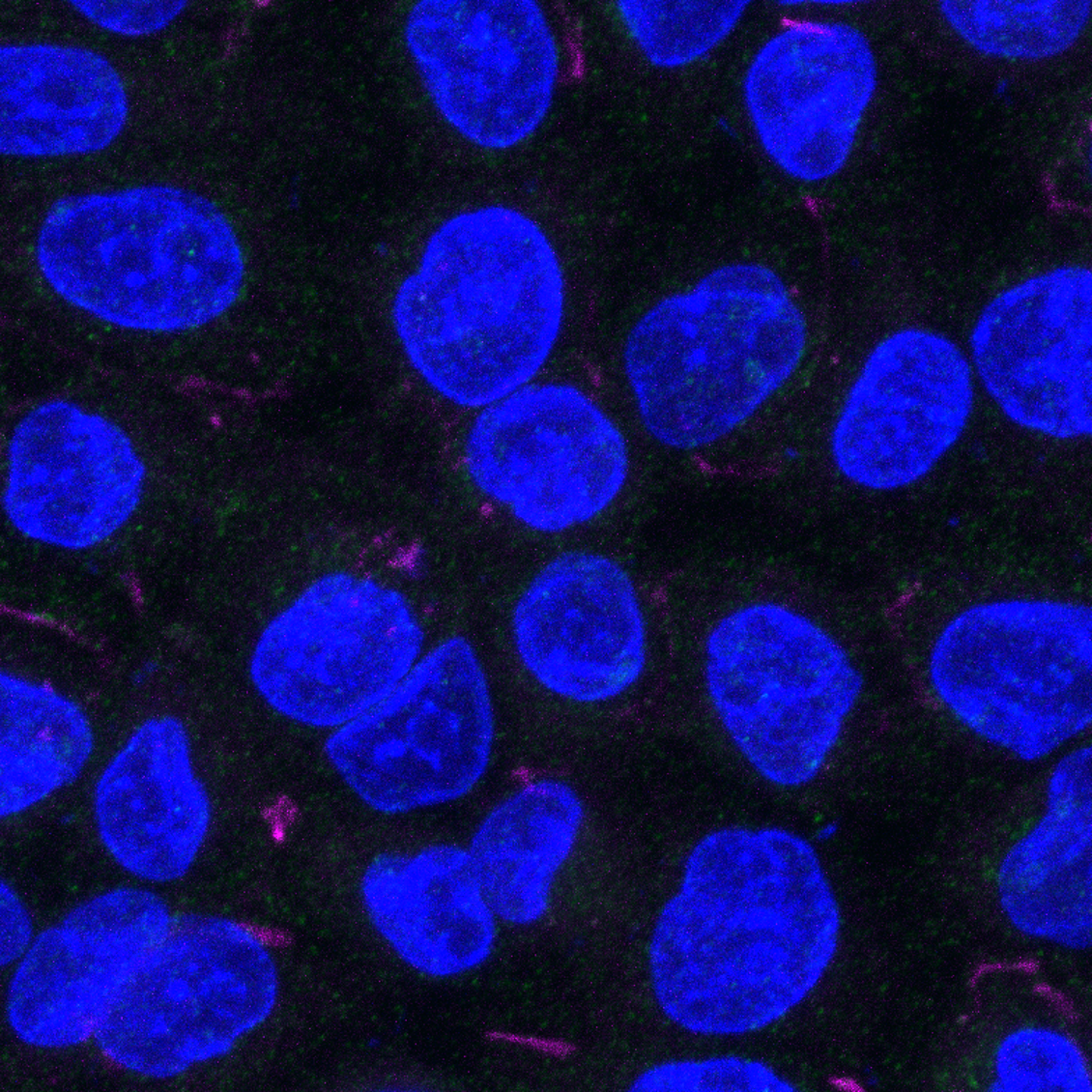

Supplement: Supplementary file 5 — Source Data Fig. 4 [file 44319_2023_18_MOESM5_ESM.zip › Figure_3/3C/3C xy images/0h_left_merge.tif]

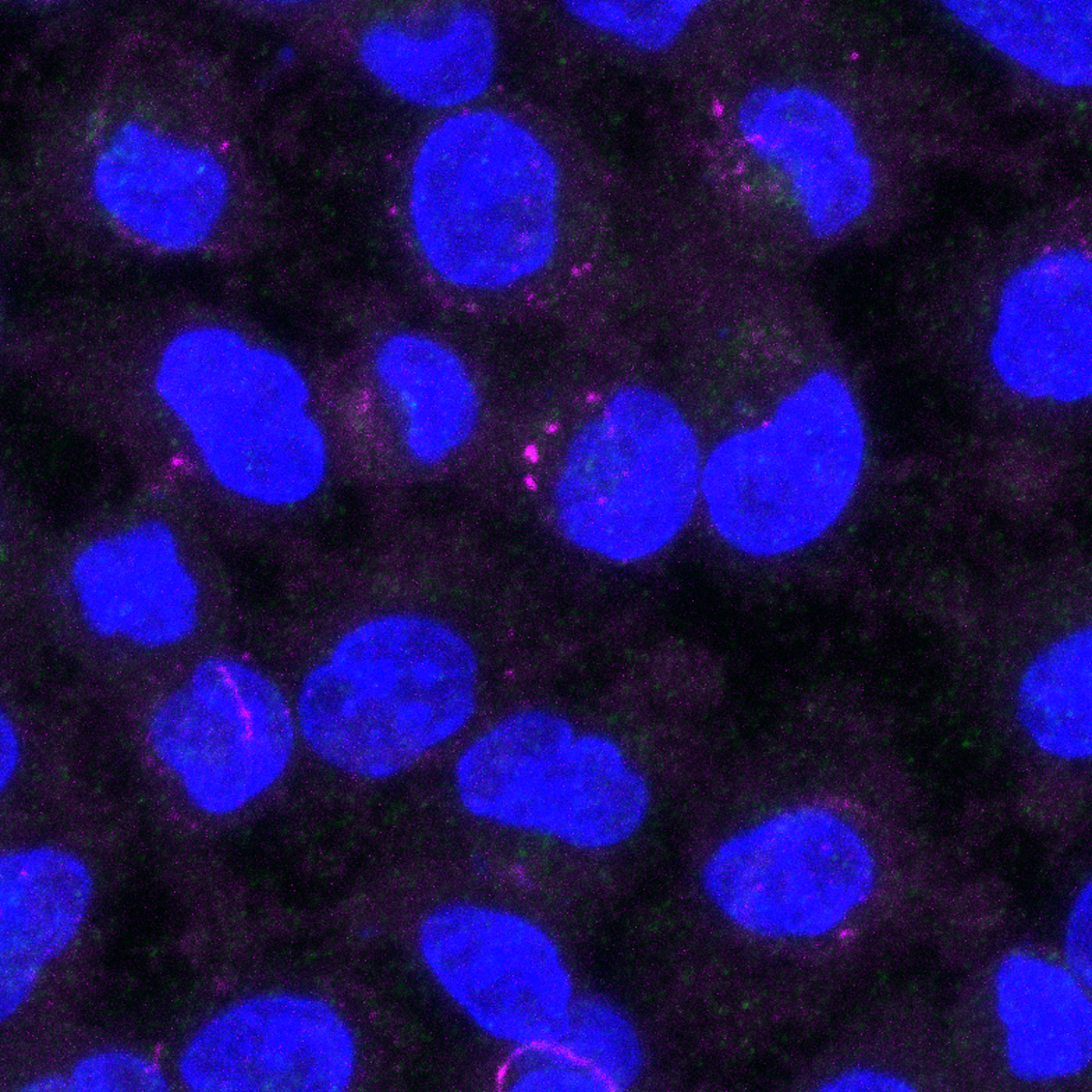

Supplement: Supplementary file 5 — Source Data Fig. 4 [file 44319_2023_18_MOESM5_ESM.zip › Figure_3/3C/3C xy images/0h_middle_merge.tif]

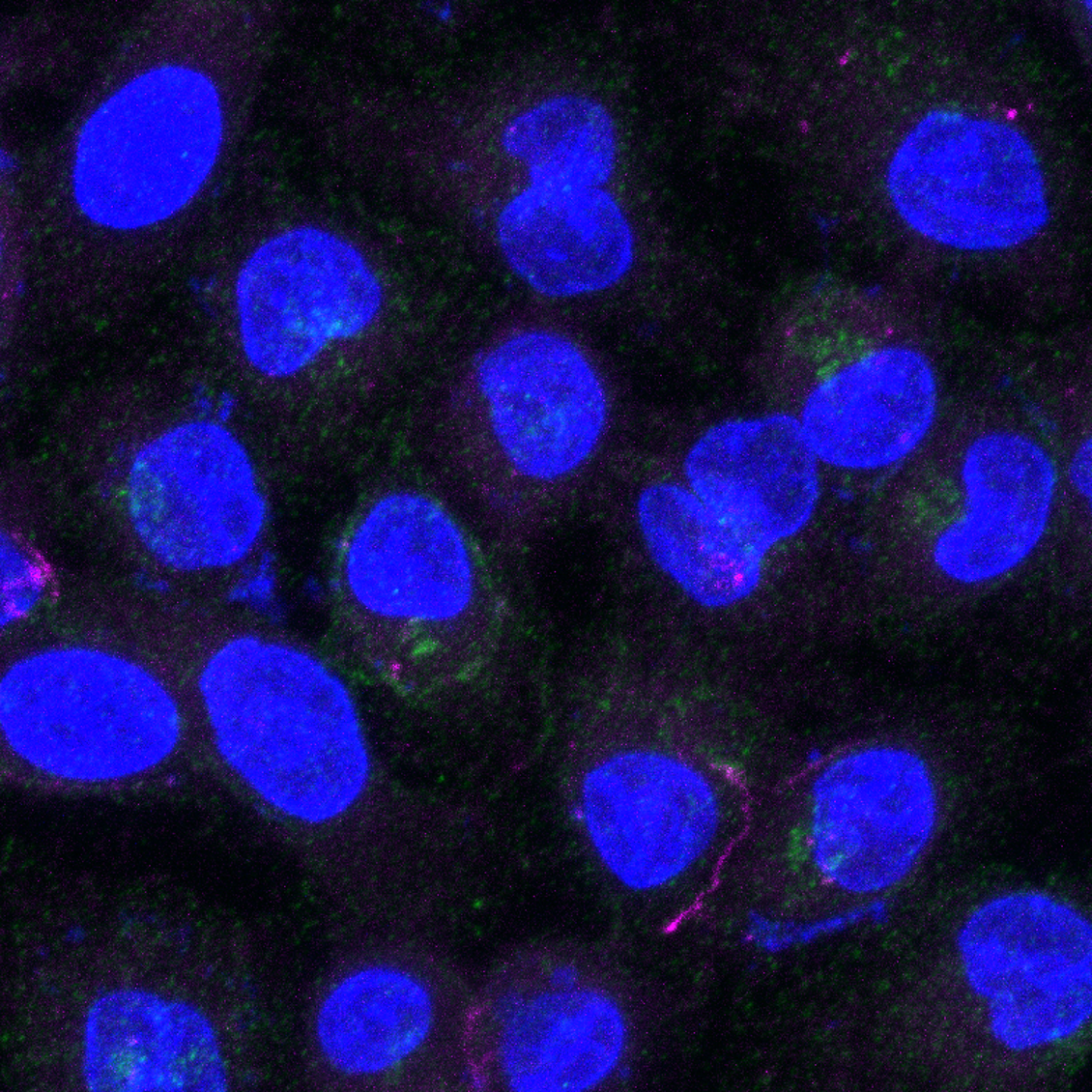

Supplement: Supplementary file 5 — Source Data Fig. 4 [file 44319_2023_18_MOESM5_ESM.zip › Figure_3/3C/3C xy images/0h_right_merge.tif]

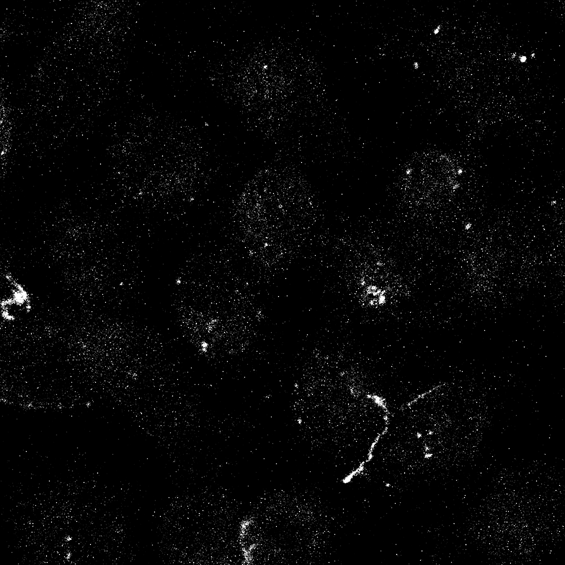

Supplement: Supplementary file 5 — Source Data Fig. 4 [file 44319_2023_18_MOESM5_ESM.zip › Figure_3/3C/3C xy images/0h_right_ZO1.tif]

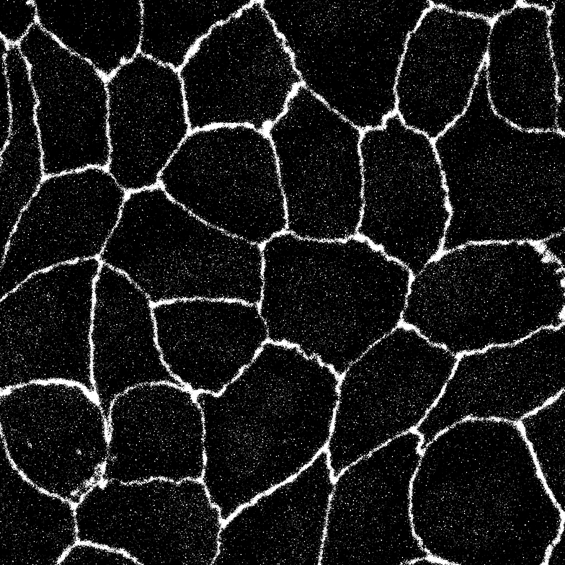

Supplement: Supplementary file 5 — Source Data Fig. 4 [file 44319_2023_18_MOESM5_ESM.zip › Figure_3/3C/3C xy images/6h_middle_ZO1.tif]

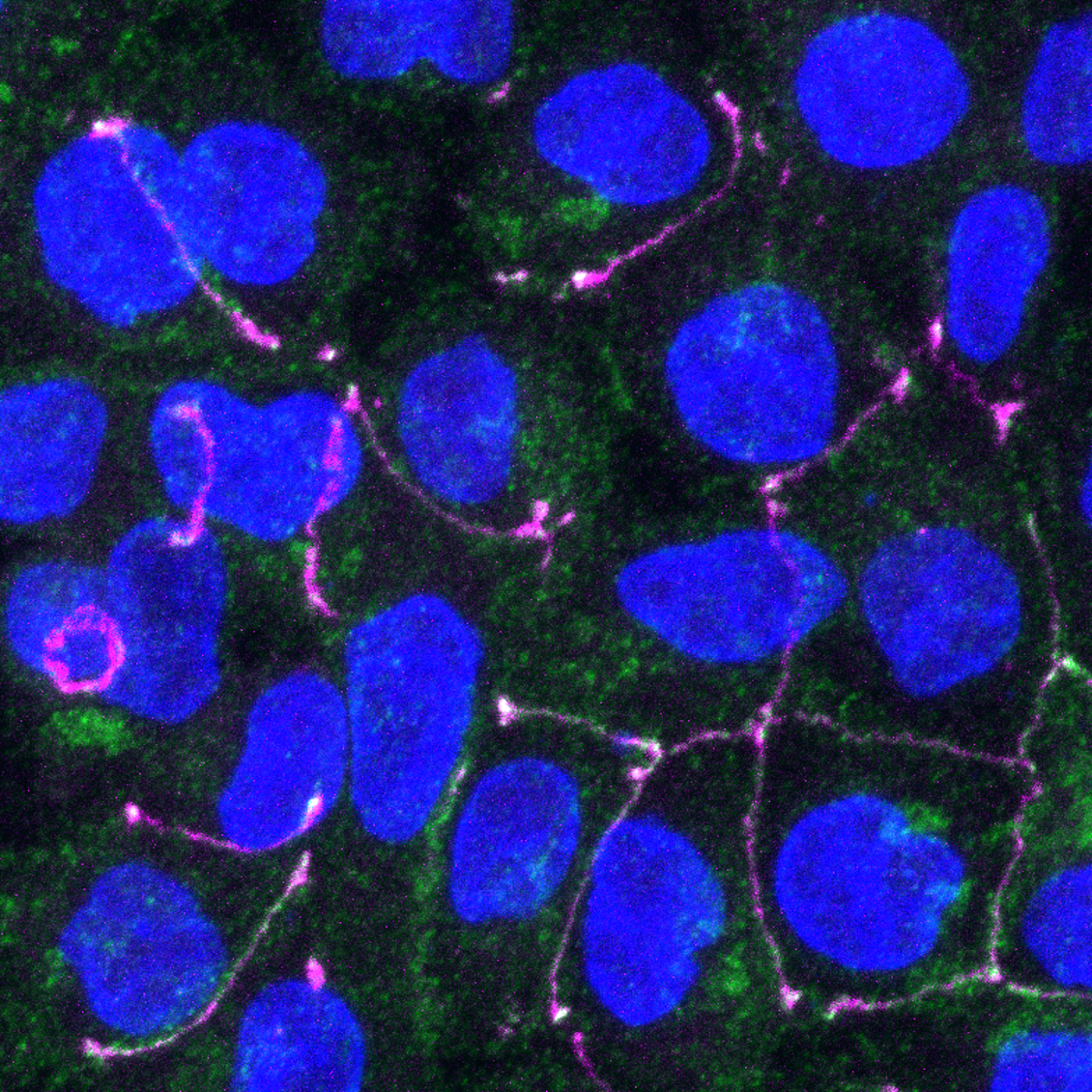

Supplement: Supplementary file 5 — Source Data Fig. 4 [file 44319_2023_18_MOESM5_ESM.zip › Figure_3/3C/3C xy images/2h_left_merge.tif]

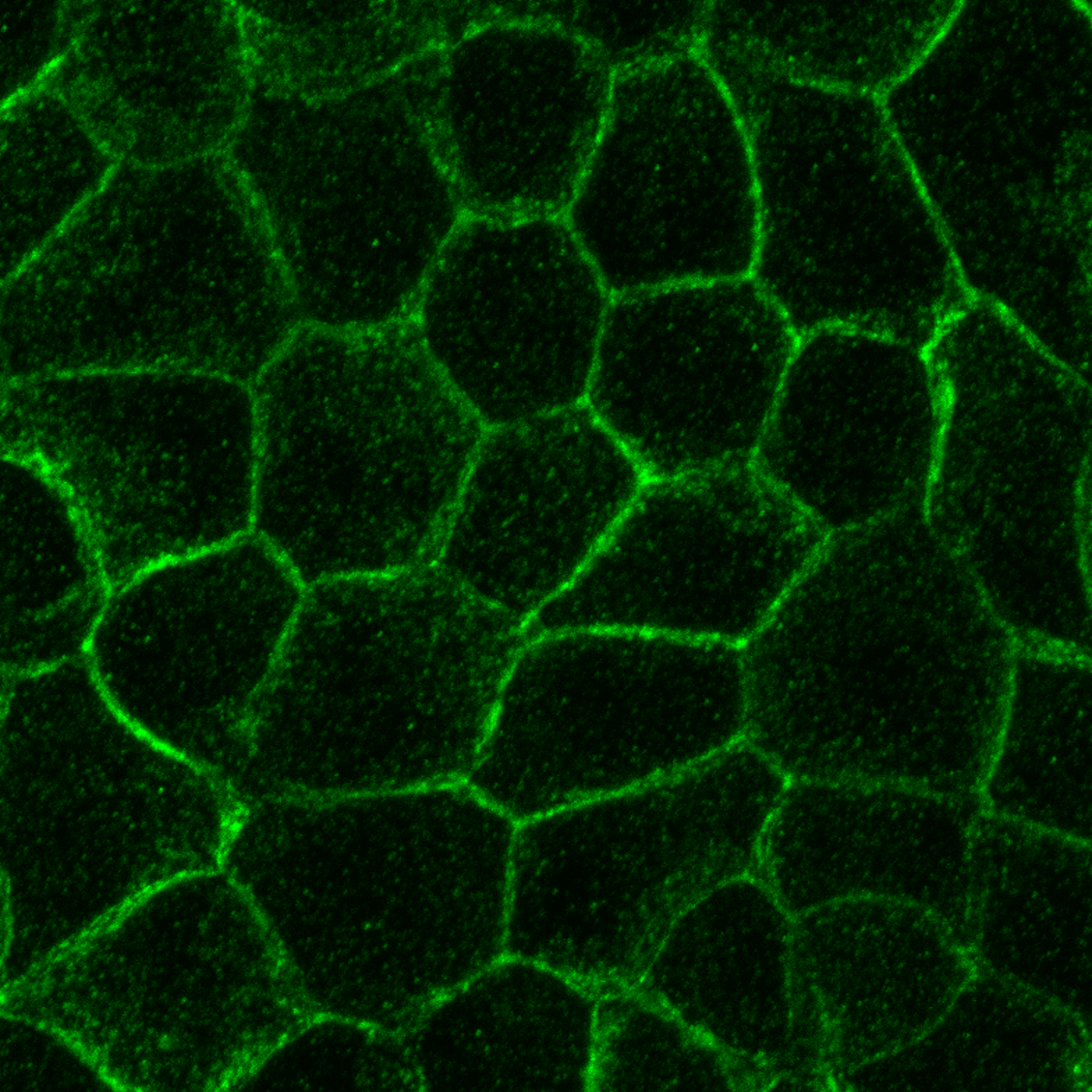

Supplement: Supplementary file 5 — Source Data Fig. 4 [file 44319_2023_18_MOESM5_ESM.zip › Figure_3/3C/3C xy images/6h_left_Ecad.tif]

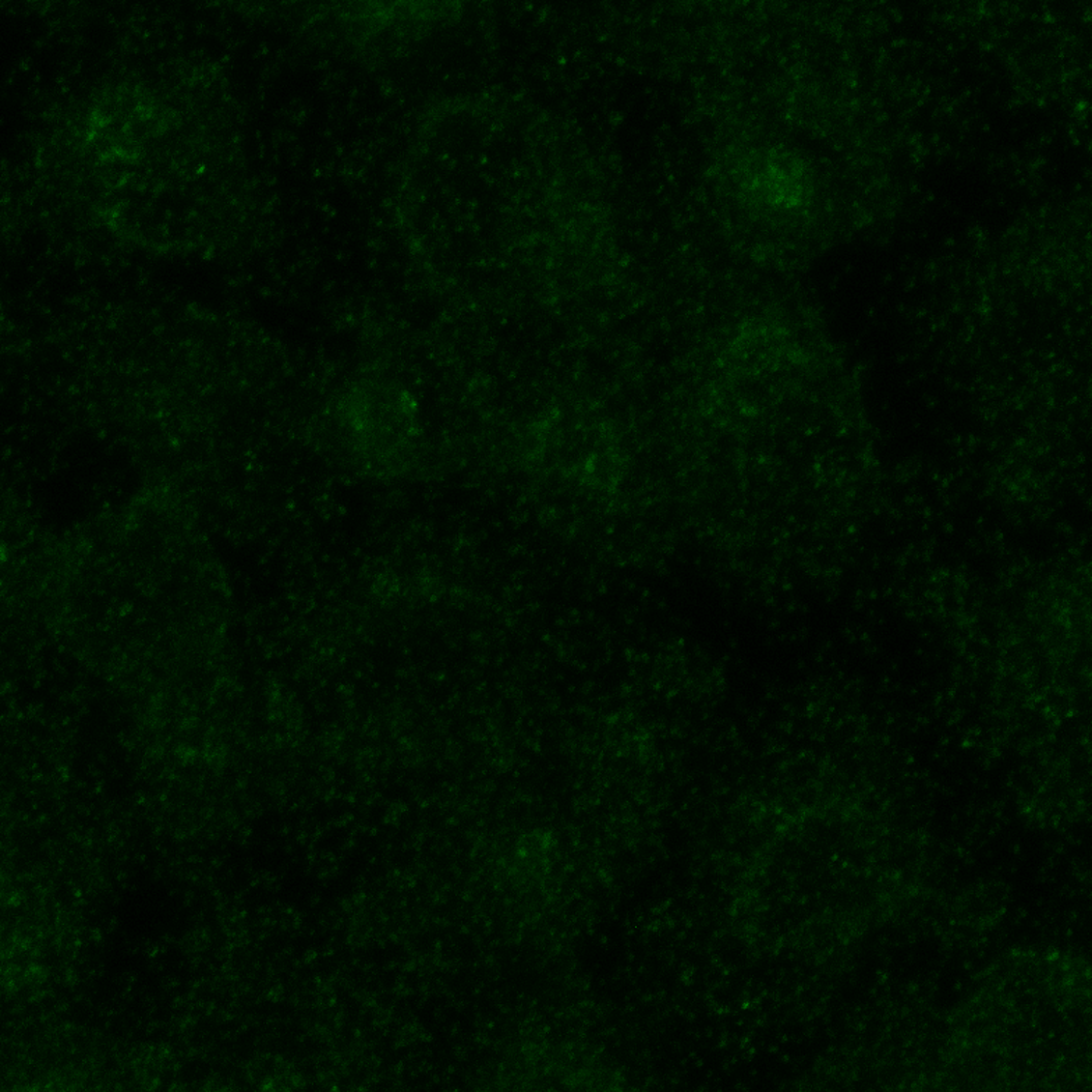

Supplement: Supplementary file 5 — Source Data Fig. 4 [file 44319_2023_18_MOESM5_ESM.zip › Figure_3/3C/3C xy images/0h_middle_Ecad.tif]

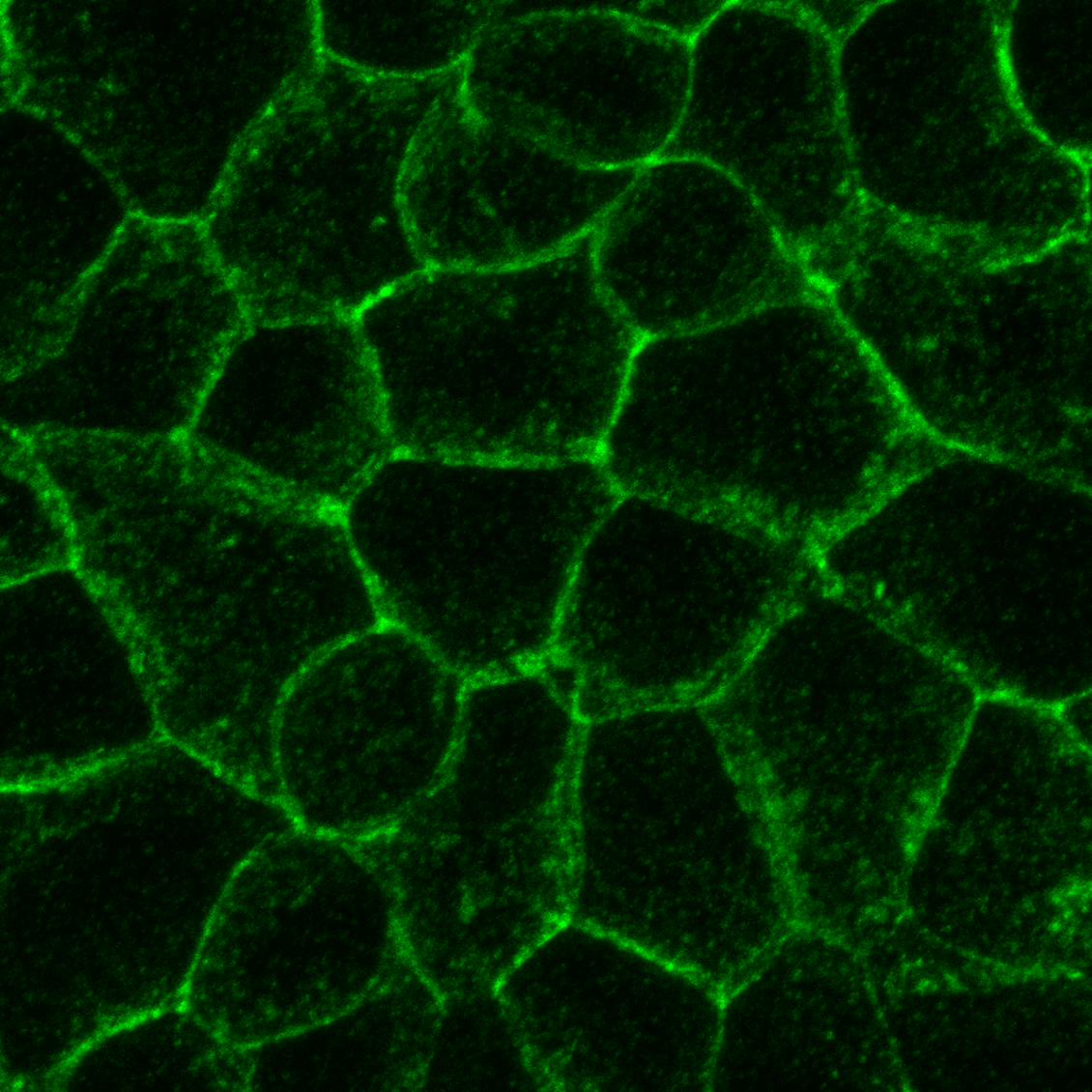

Supplement: Supplementary file 5 — Source Data Fig. 4 [file 44319_2023_18_MOESM5_ESM.zip › Figure_3/3C/3C xy images/6h_right_Ecad.tif]

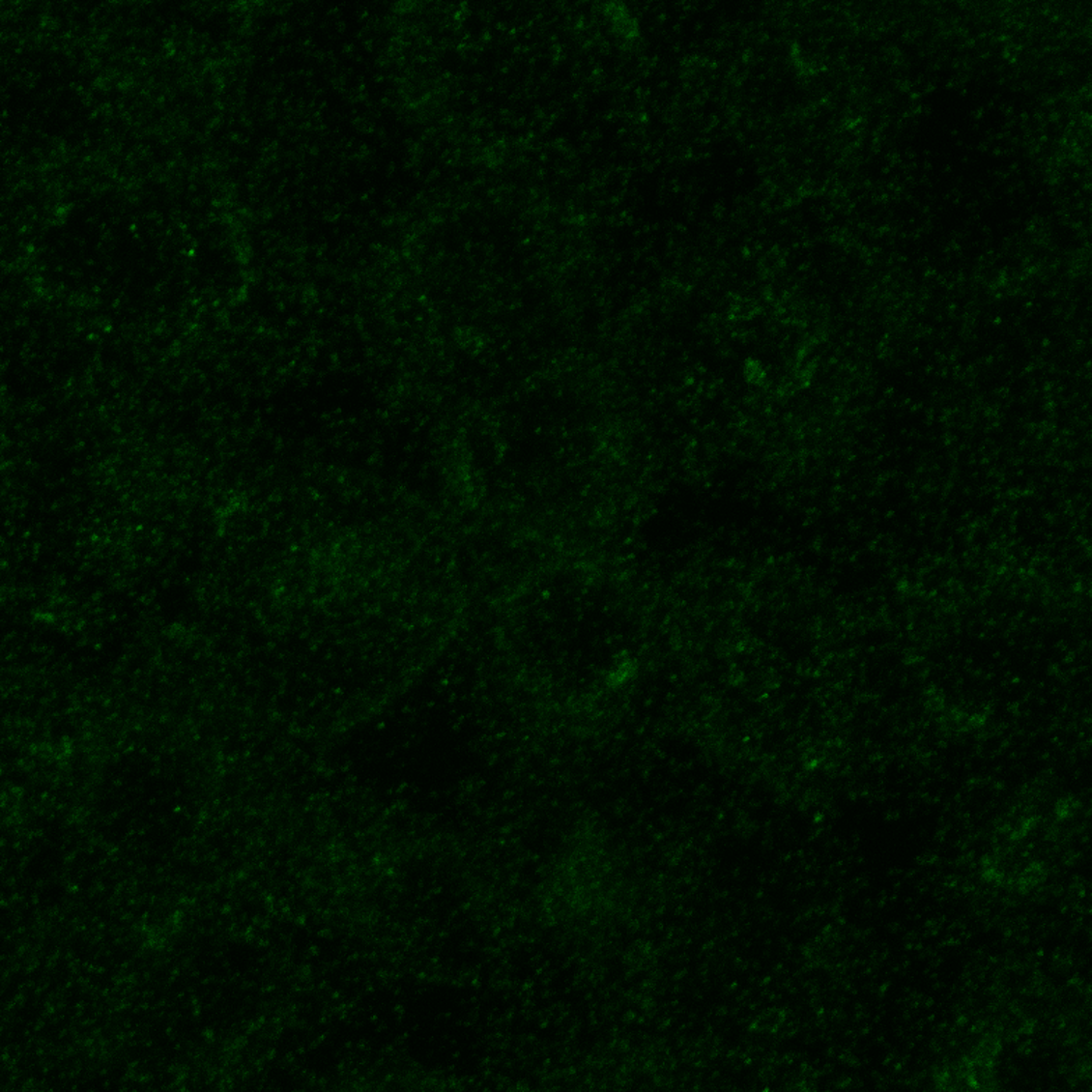

Supplement: Supplementary file 5 — Source Data Fig. 4 [file 44319_2023_18_MOESM5_ESM.zip › Figure_3/3C/3C xy images/0h_left_Ecad.tif]

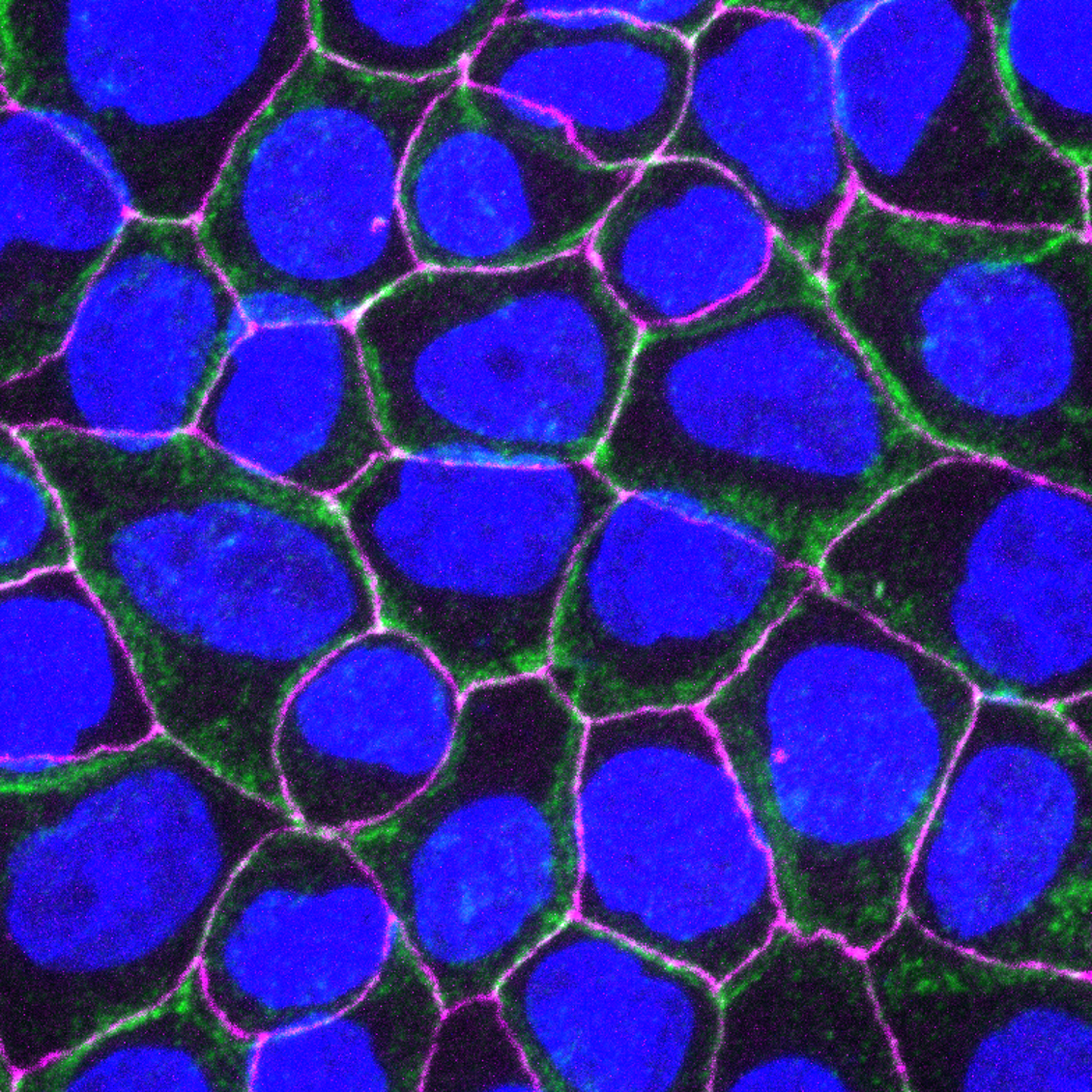

Supplement: Supplementary file 5 — Source Data Fig. 4 [file 44319_2023_18_MOESM5_ESM.zip › Figure_3/3C/3C xy images/6h_right_merge.tif]

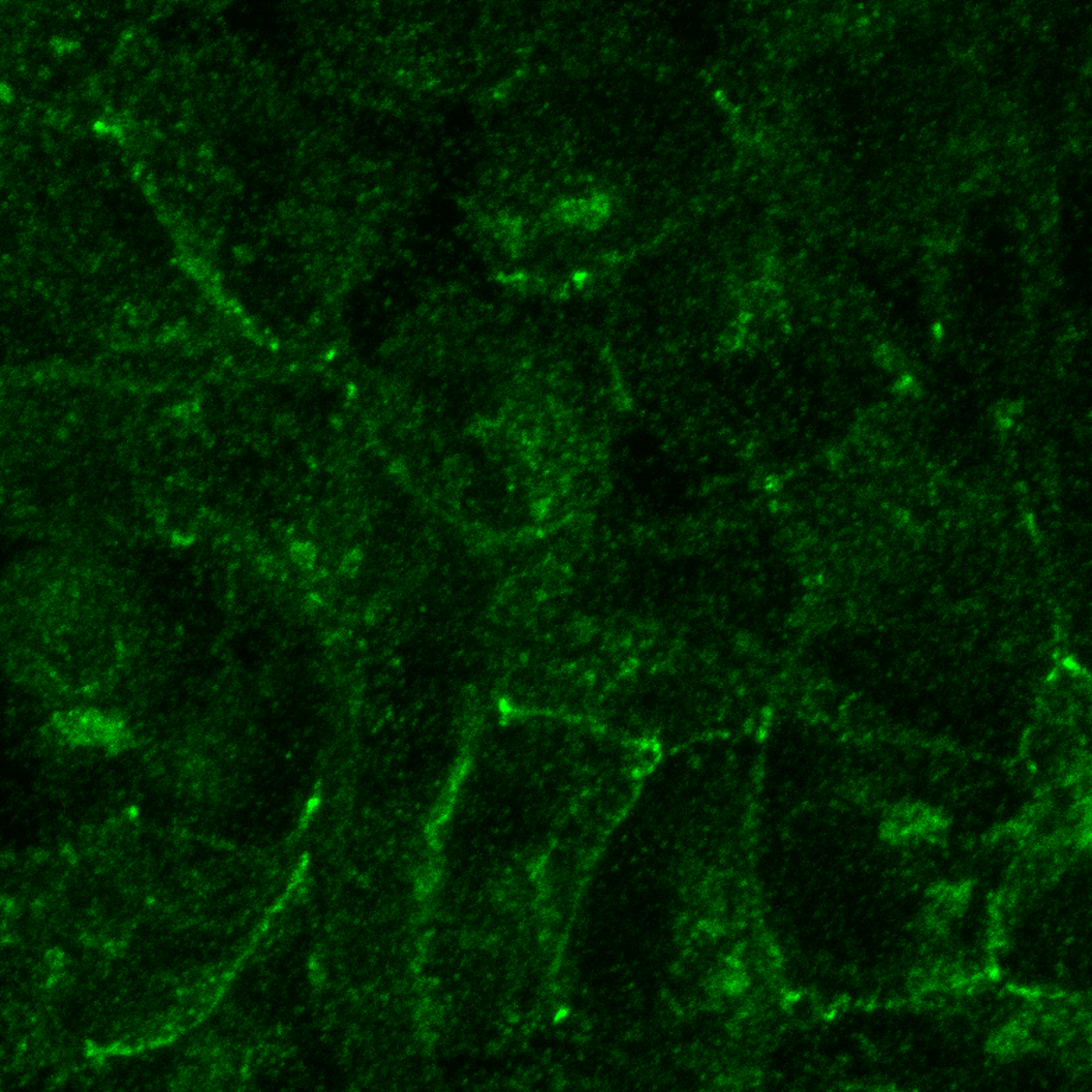

Supplement: Supplementary file 5 — Source Data Fig. 4 [file 44319_2023_18_MOESM5_ESM.zip › Figure_3/3C/3C xy images/2h_left_Ecad.tif]

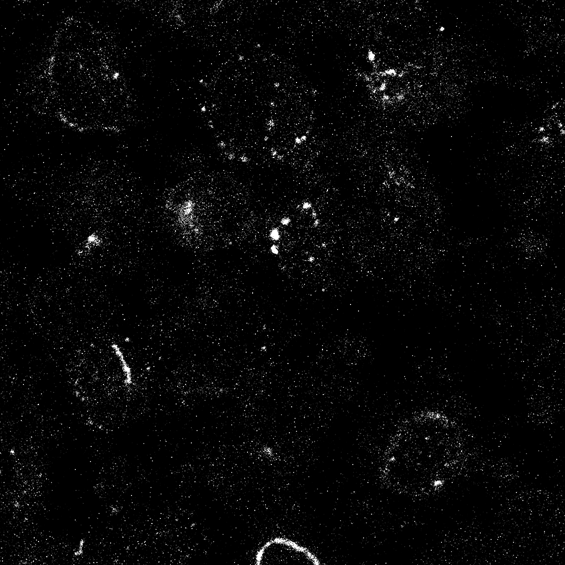

Supplement: Supplementary file 5 — Source Data Fig. 4 [file 44319_2023_18_MOESM5_ESM.zip › Figure_3/3C/3C xy images/0h_middle_ZO1.tif]

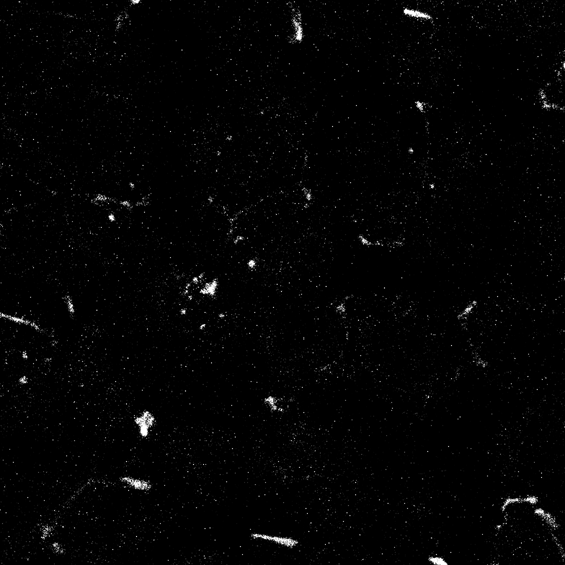

Supplement: Supplementary file 5 — Source Data Fig. 4 [file 44319_2023_18_MOESM5_ESM.zip › Figure_3/3C/3C xy images/0h_left_ZO1.tif]

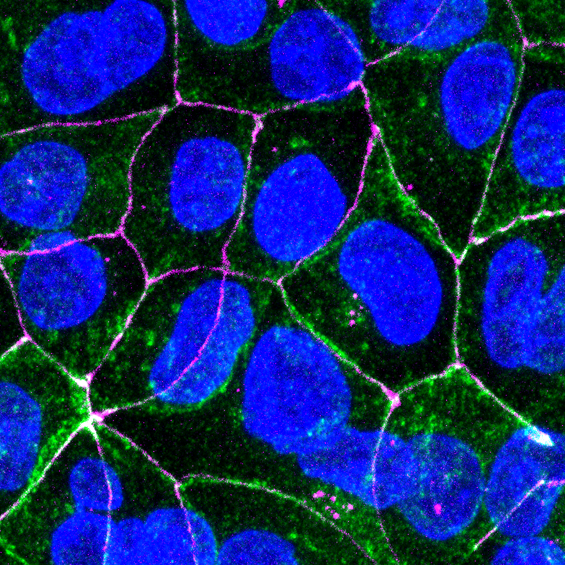

Supplement: Supplementary file 5 — Source Data Fig. 4 [file 44319_2023_18_MOESM5_ESM.zip › Figure_3/3C/3C xy images/2h_right_merge.tif]

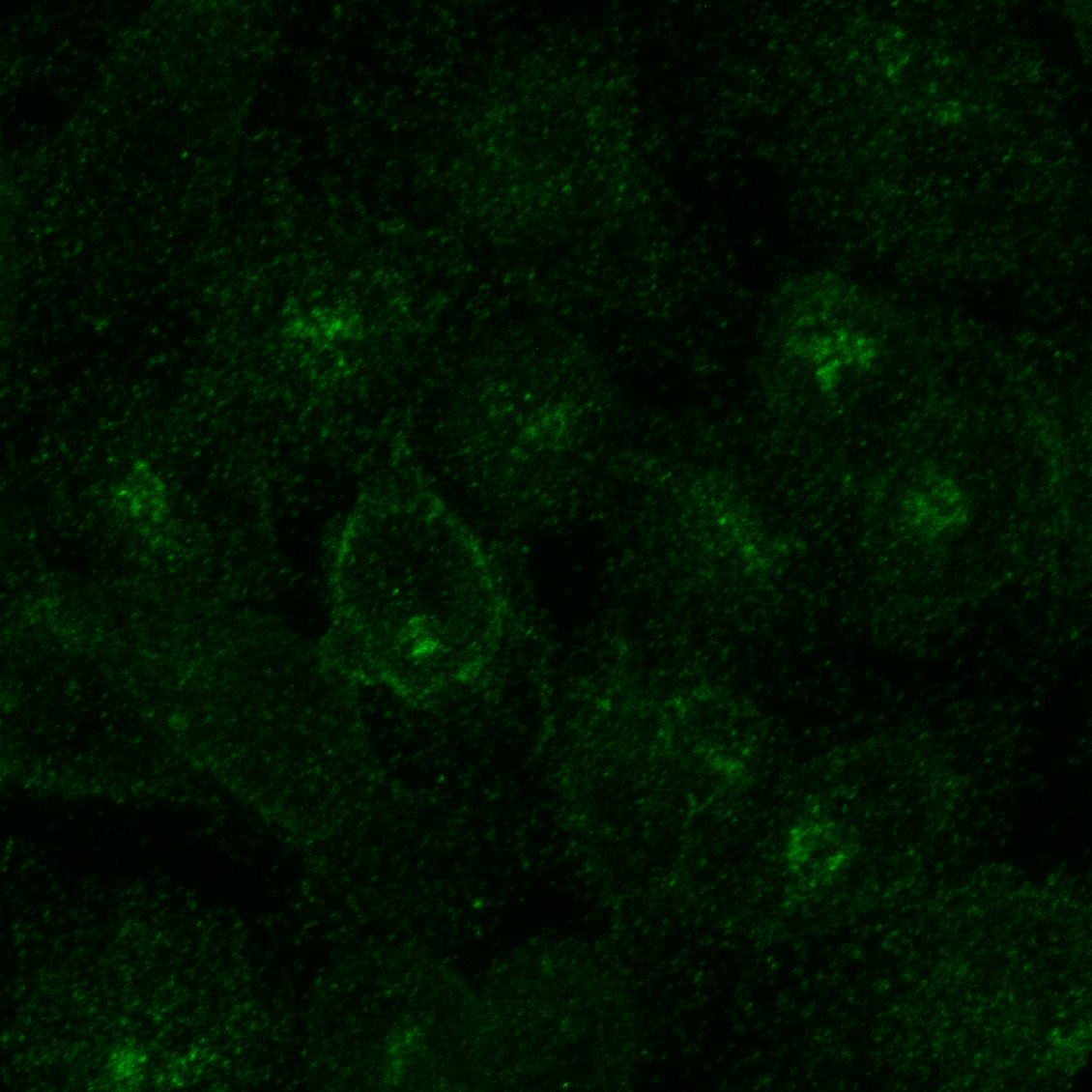

Supplement: Supplementary file 5 — Source Data Fig. 4 [file 44319_2023_18_MOESM5_ESM.zip › Figure_3/3C/3C xy images/0h_right_Ecad.tif]

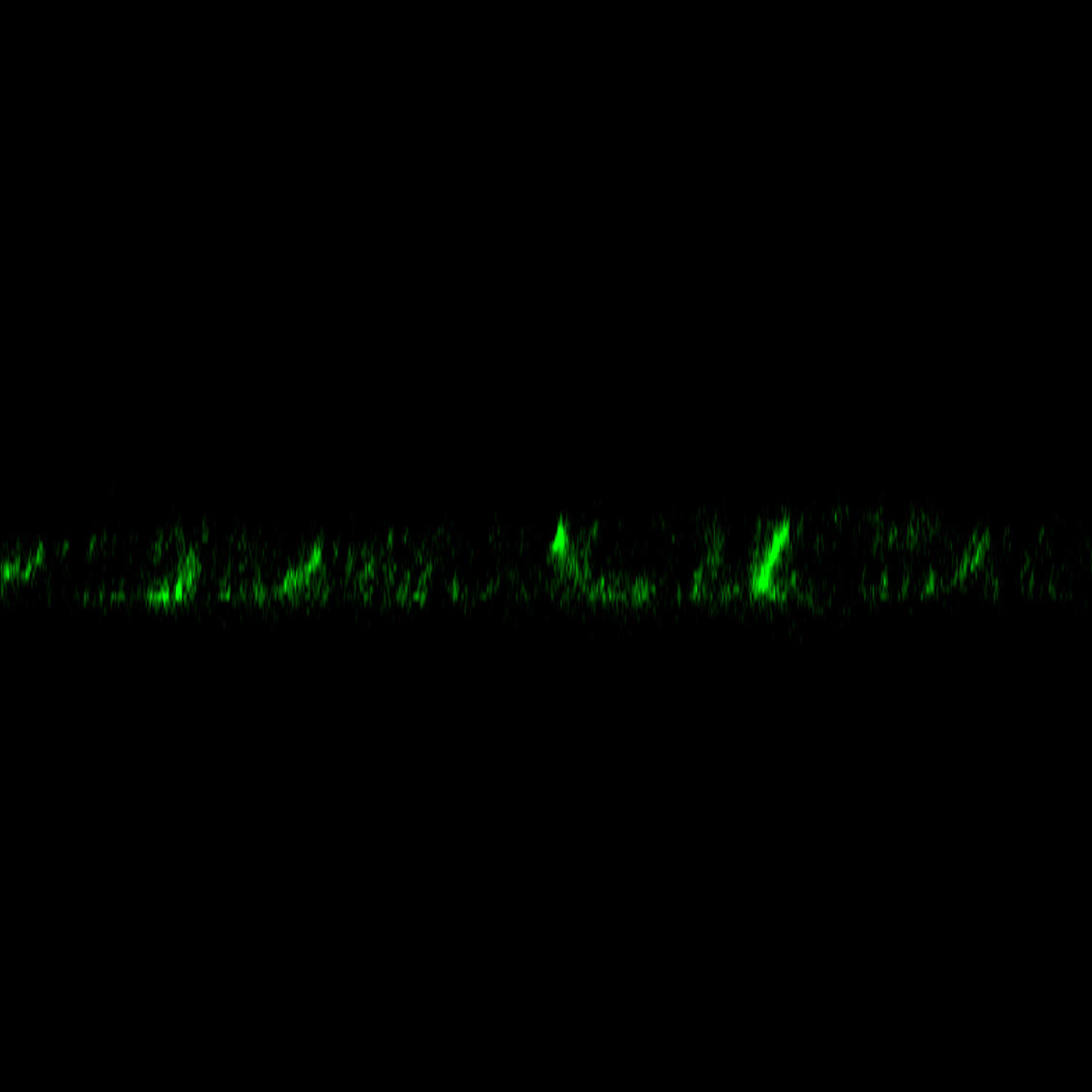

Supplement: Supplementary file 5 — Source Data Fig. 4 [file 44319_2023_18_MOESM5_ESM.zip › Figure_3/3C/3C xz images/2h_middle_Ecad.tif]

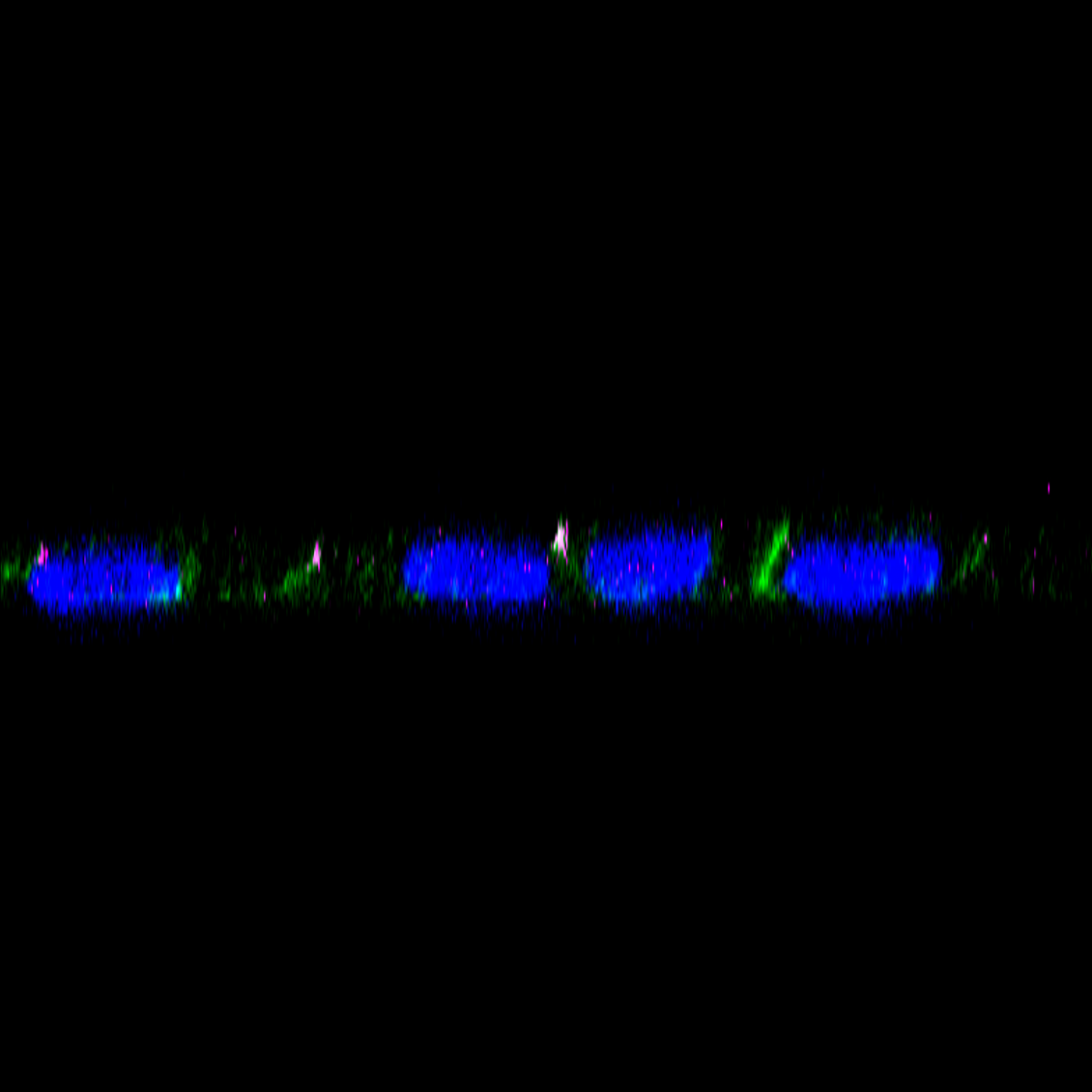

Supplement: Supplementary file 5 — Source Data Fig. 4 [file 44319_2023_18_MOESM5_ESM.zip › Figure_3/3C/3C xz images/2h_middle_merge.tif]

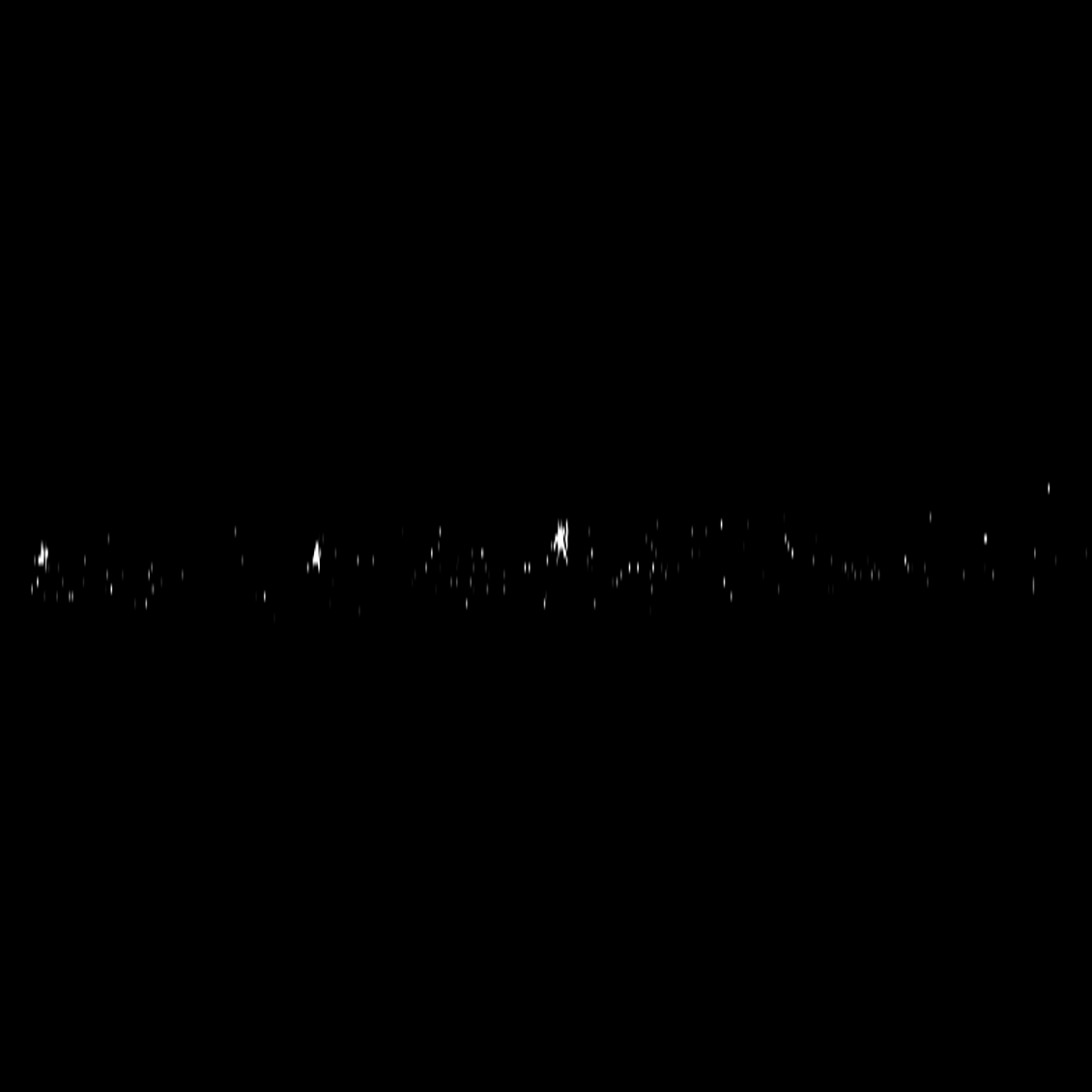

Supplement: Supplementary file 5 — Source Data Fig. 4 [file 44319_2023_18_MOESM5_ESM.zip › Figure_3/3C/3C xz images/2h_middle_ZO1.tif]

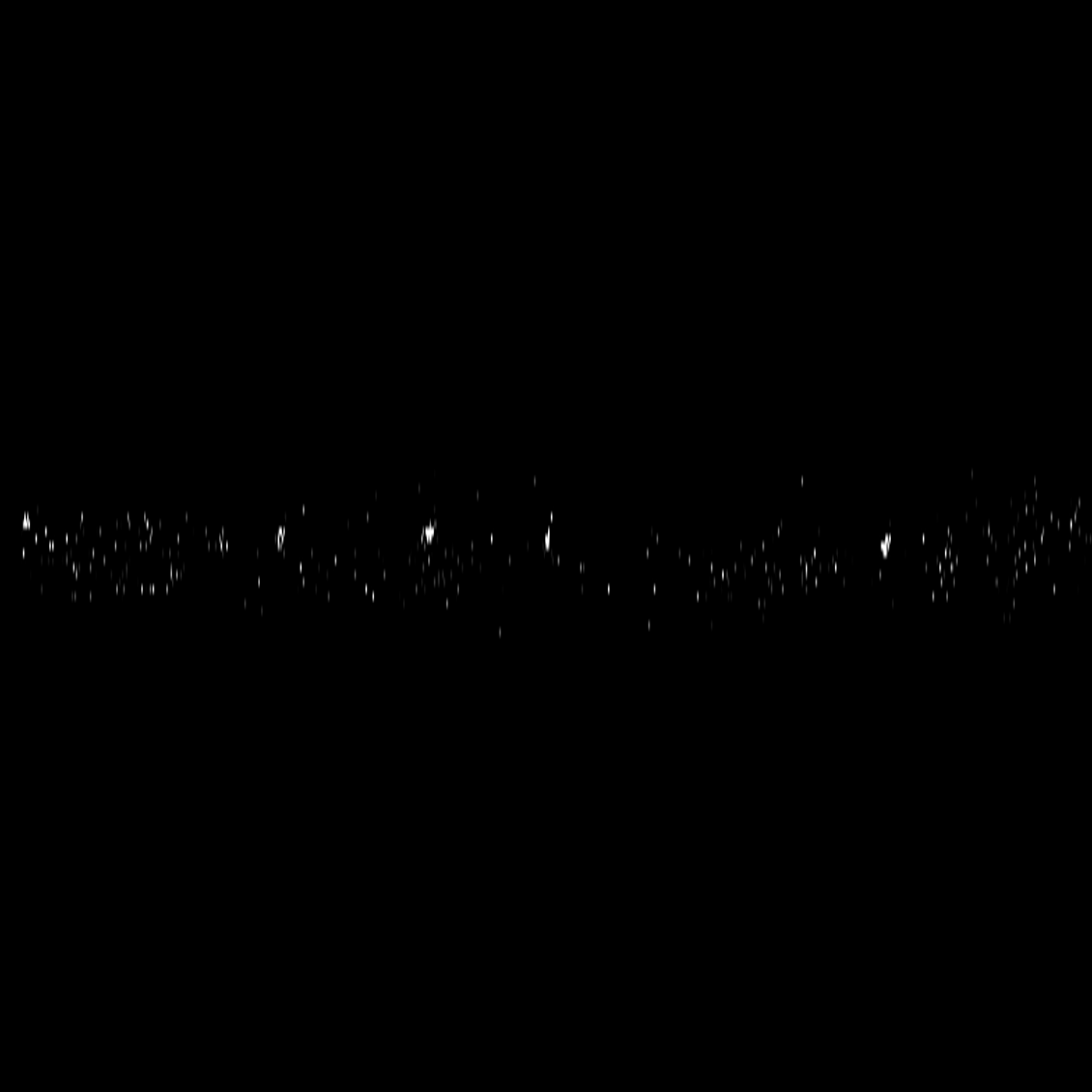

Supplement: Supplementary file 5 — Source Data Fig. 4 [file 44319_2023_18_MOESM5_ESM.zip › Figure_3/3C/3C xz images/2h_right_ZO1.tif]

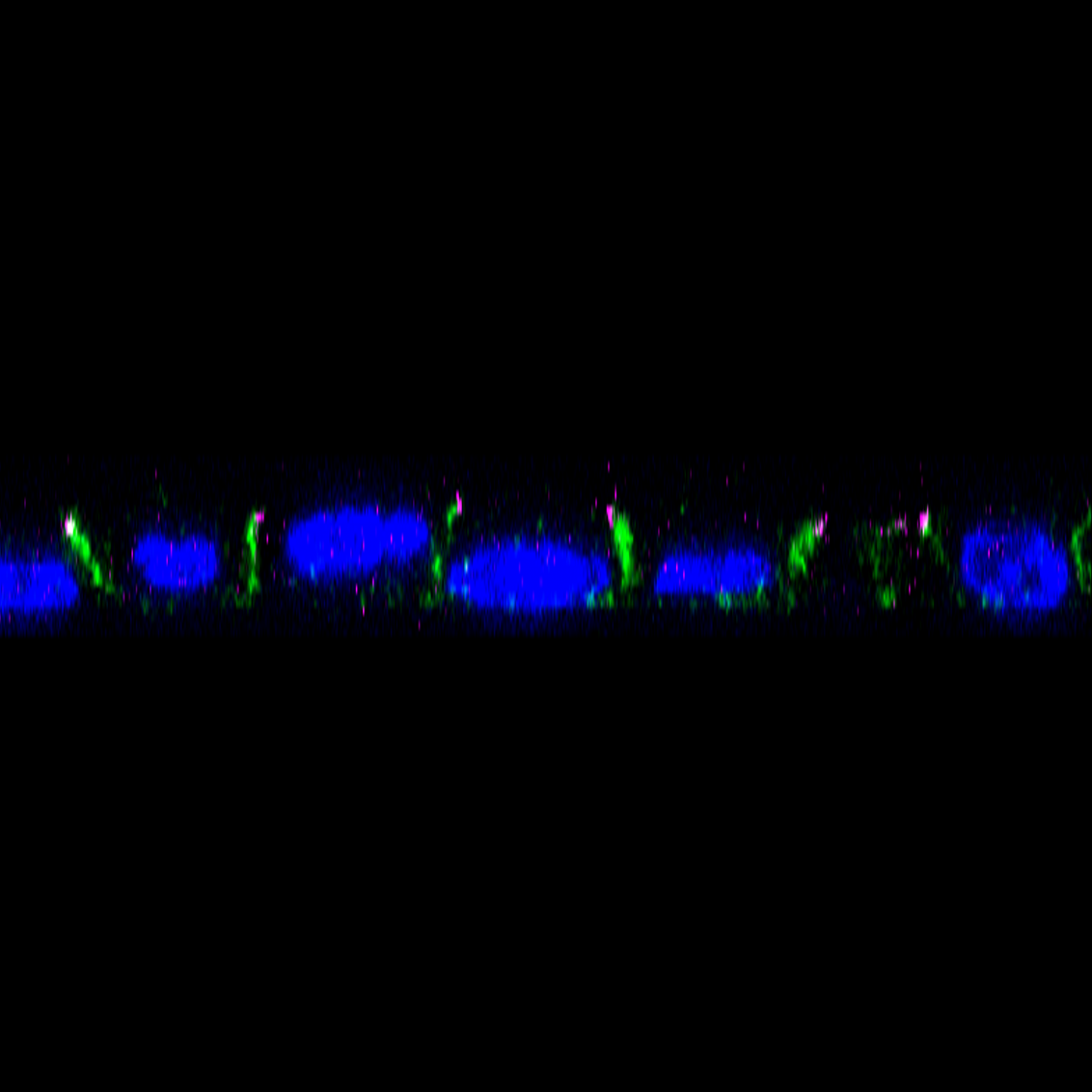

Supplement: Supplementary file 5 — Source Data Fig. 4 [file 44319_2023_18_MOESM5_ESM.zip › Figure_3/3C/3C xz images/6h_left_merge.tif]

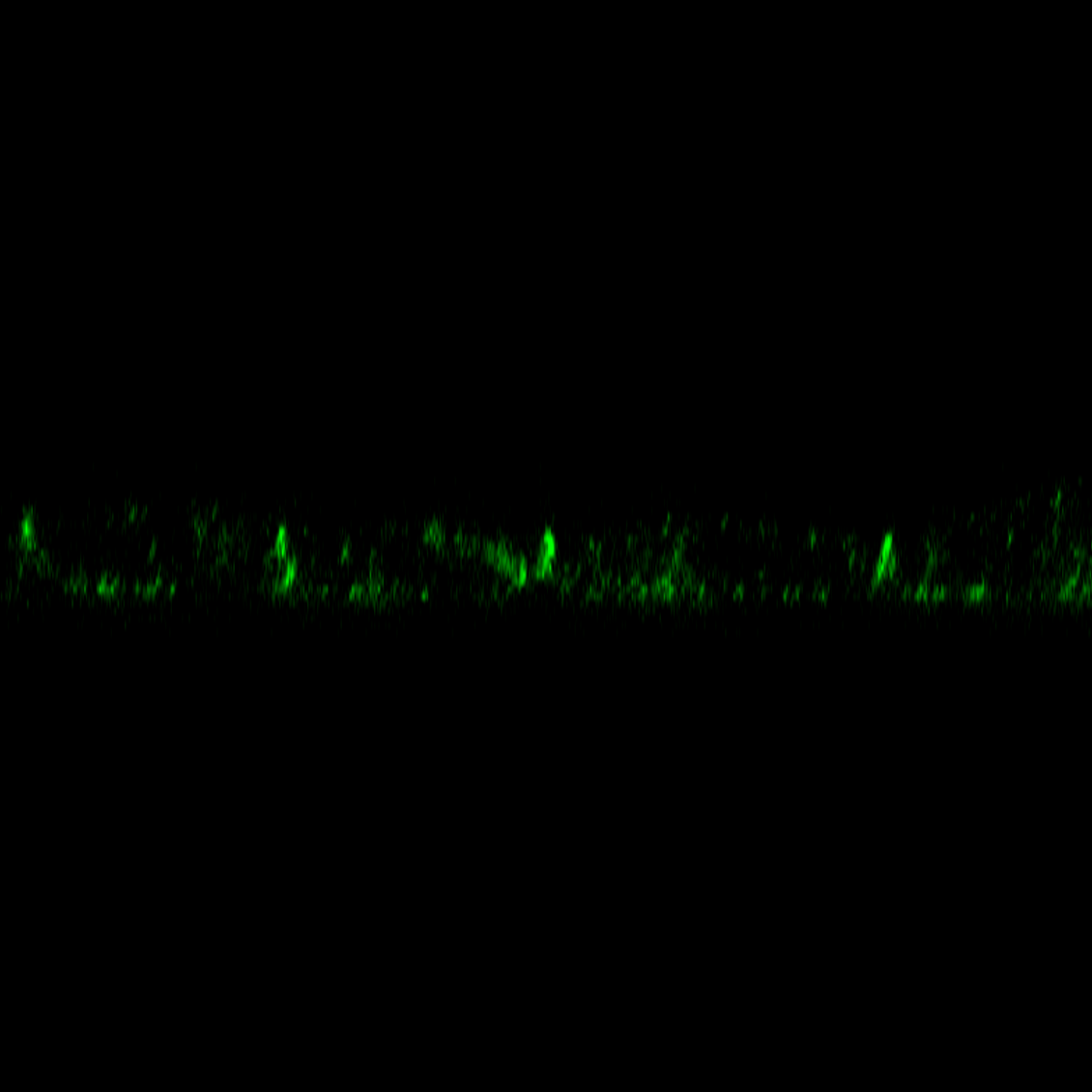

Supplement: Supplementary file 5 — Source Data Fig. 4 [file 44319_2023_18_MOESM5_ESM.zip › Figure_3/3C/3C xz images/2h_right_Ecad.tif]

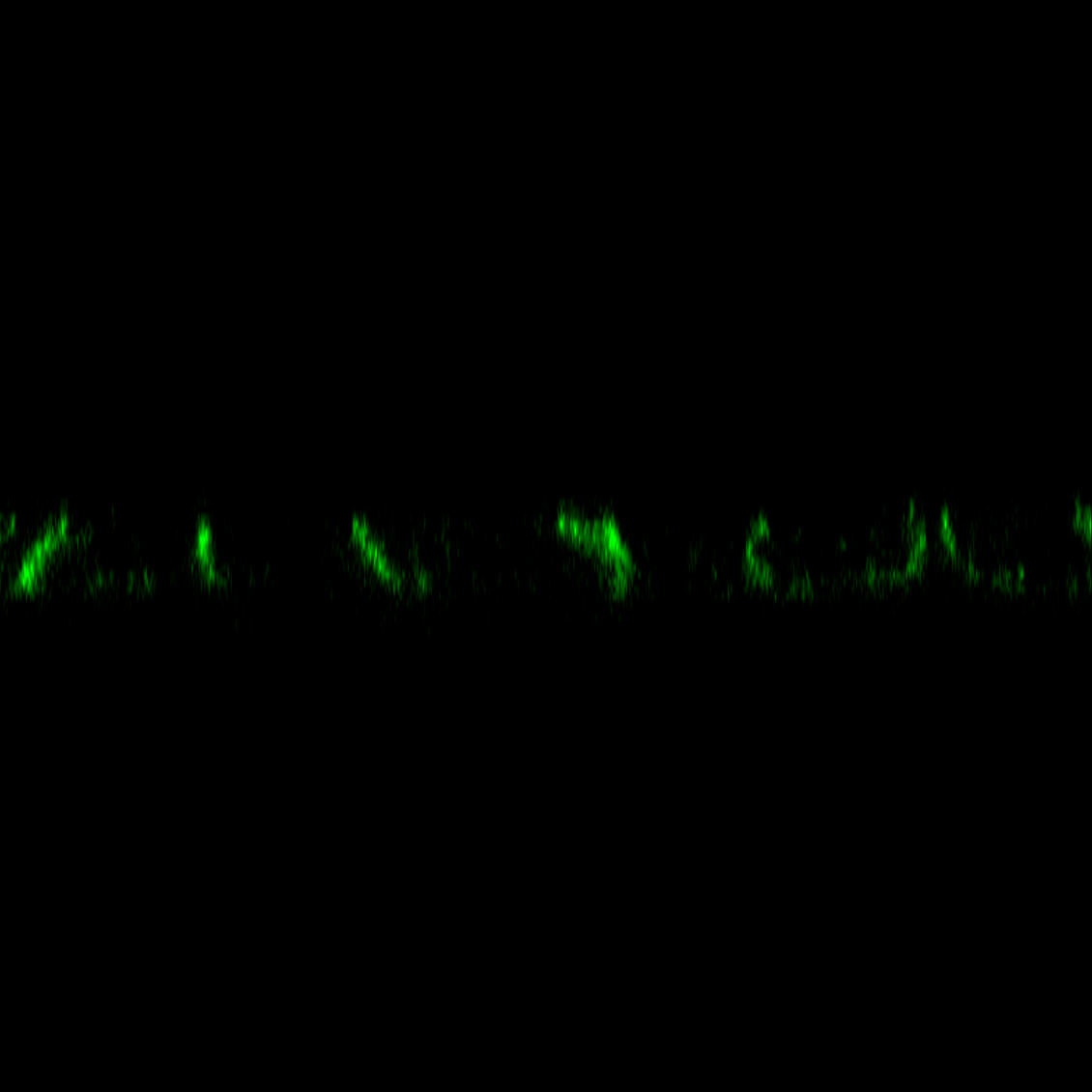

Supplement: Supplementary file 5 — Source Data Fig. 4 [file 44319_2023_18_MOESM5_ESM.zip › Figure_3/3C/3C xz images/6h_middle_Ecad.tif]

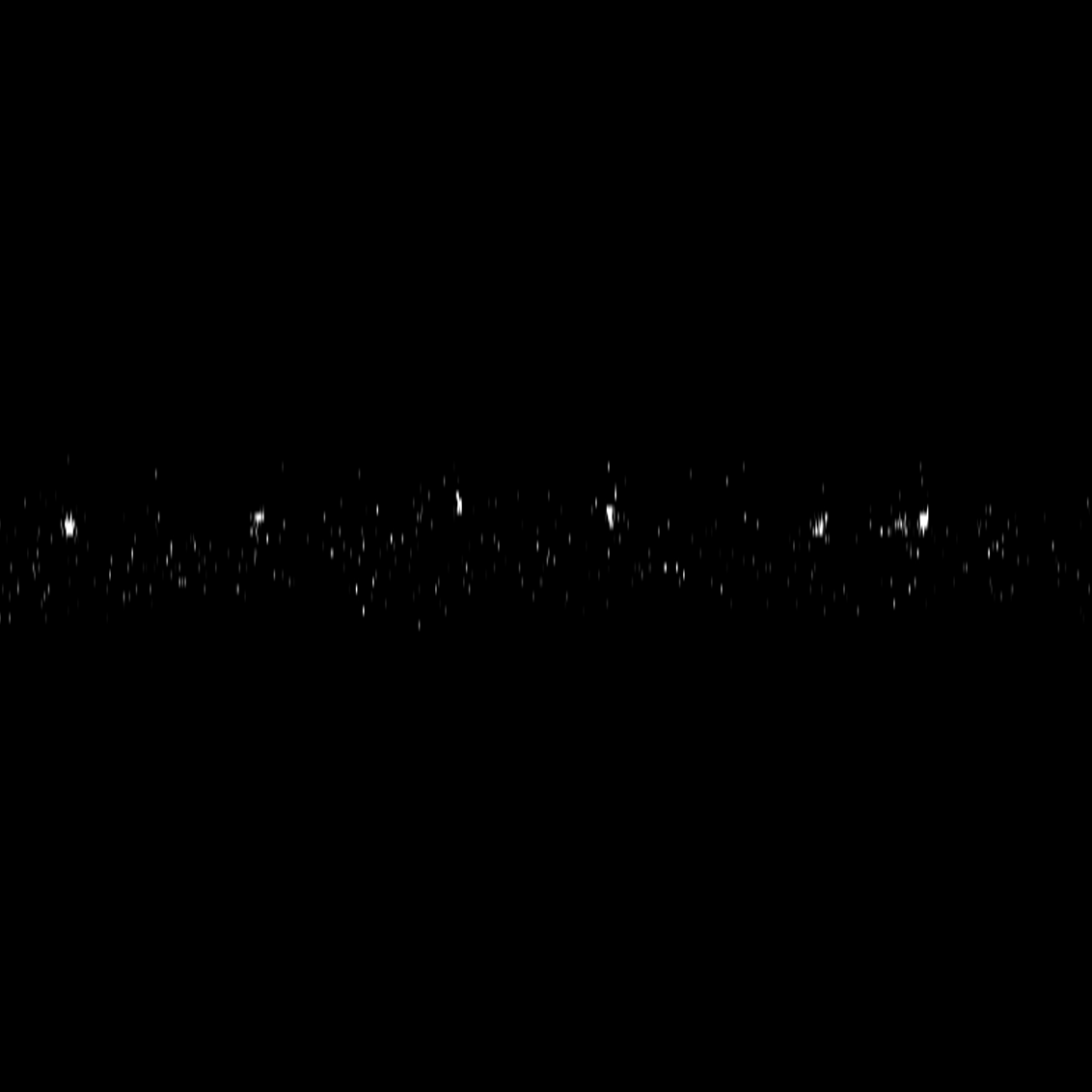

Supplement: Supplementary file 5 — Source Data Fig. 4 [file 44319_2023_18_MOESM5_ESM.zip › Figure_3/3C/3C xz images/6h_left_ZO1.tif]

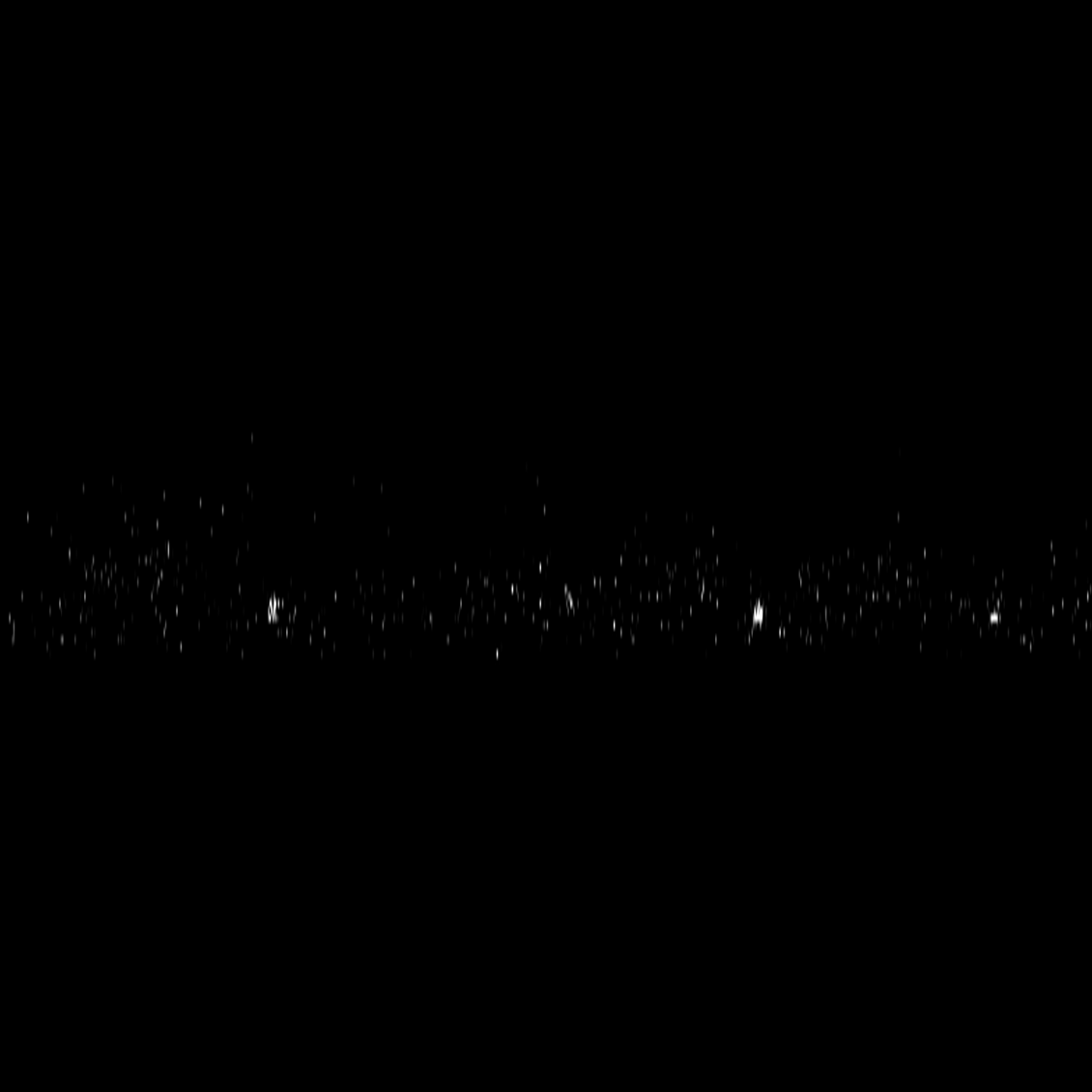

Supplement: Supplementary file 5 — Source Data Fig. 4 [file 44319_2023_18_MOESM5_ESM.zip › Figure_3/3C/3C xz images/2h_left_ZO1.tif]

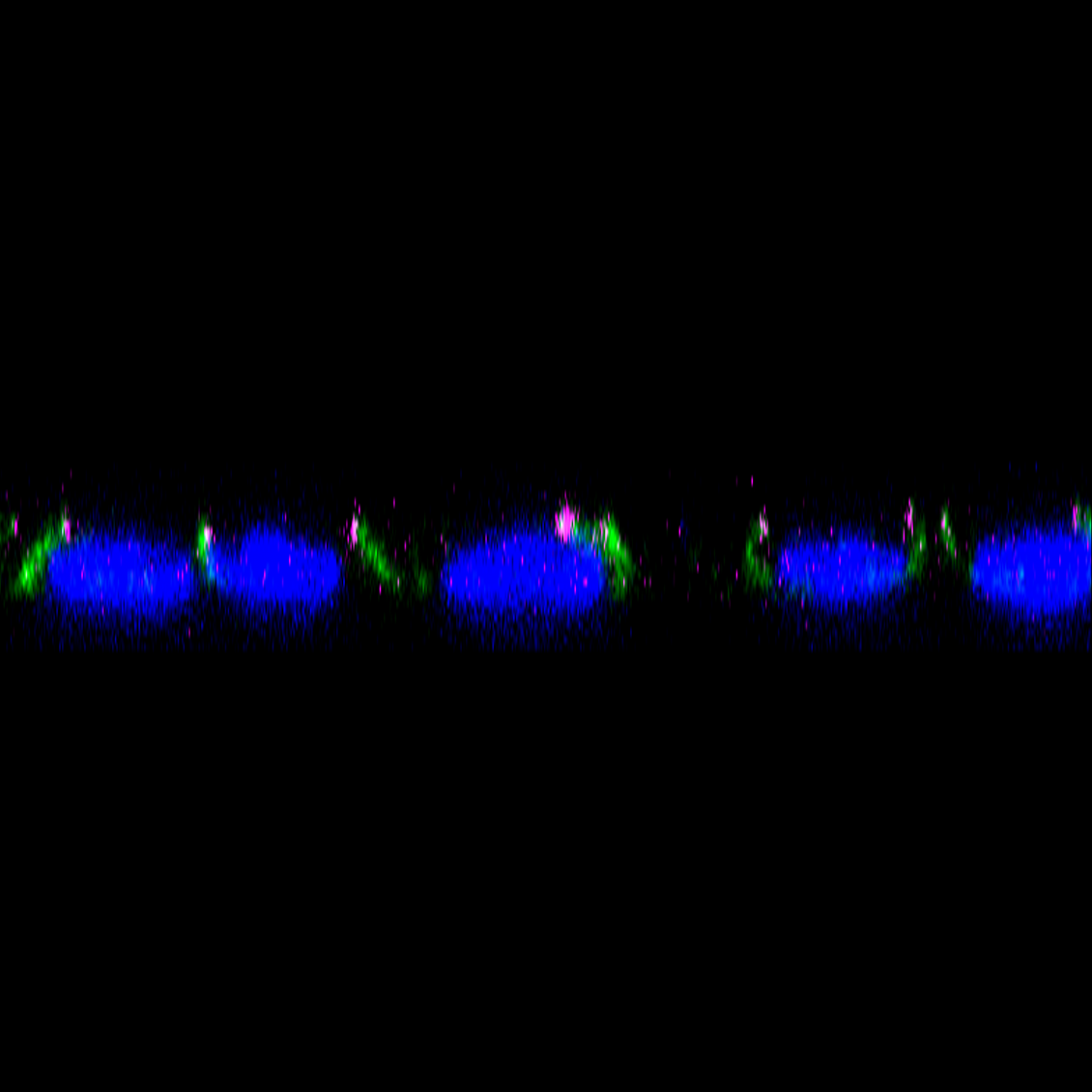

Supplement: Supplementary file 5 — Source Data Fig. 4 [file 44319_2023_18_MOESM5_ESM.zip › Figure_3/3C/3C xz images/6h_middle_merge.tif]

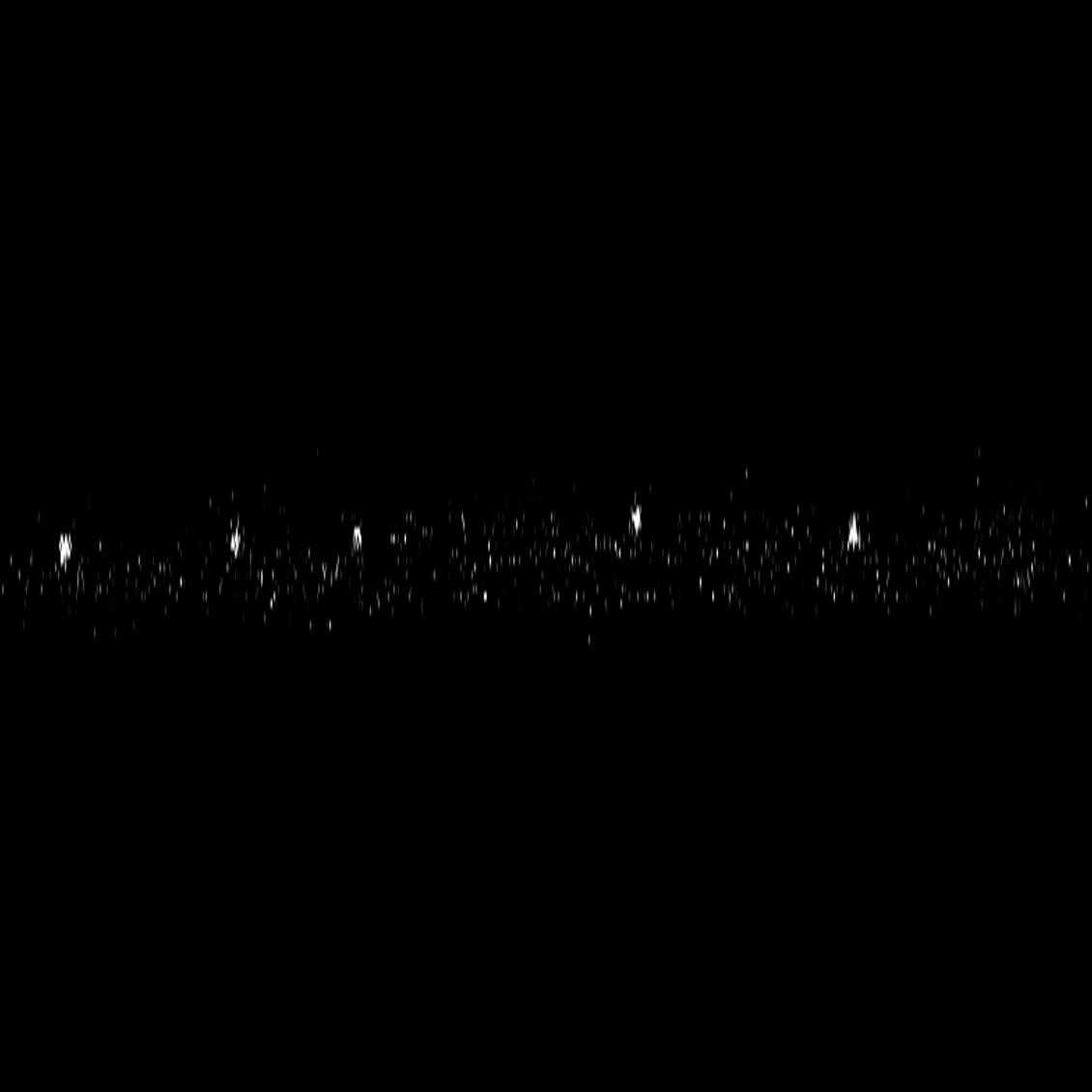

Supplement: Supplementary file 5 — Source Data Fig. 4 [file 44319_2023_18_MOESM5_ESM.zip › Figure_3/3C/3C xz images/6h_right_ZO1.tif]
