# Supplementary figures and images for: TMEM25 is a Par3-binding protein that attenuates claudin assembly during tight junction development (part 2 of 3)
Source: EMBO Rep. 2023 Dec 18;25(1):13. doi: 10.1038/s44319-023-00018-0 (PMC10897455; doi:10.1038/s44319-023-00018-0)

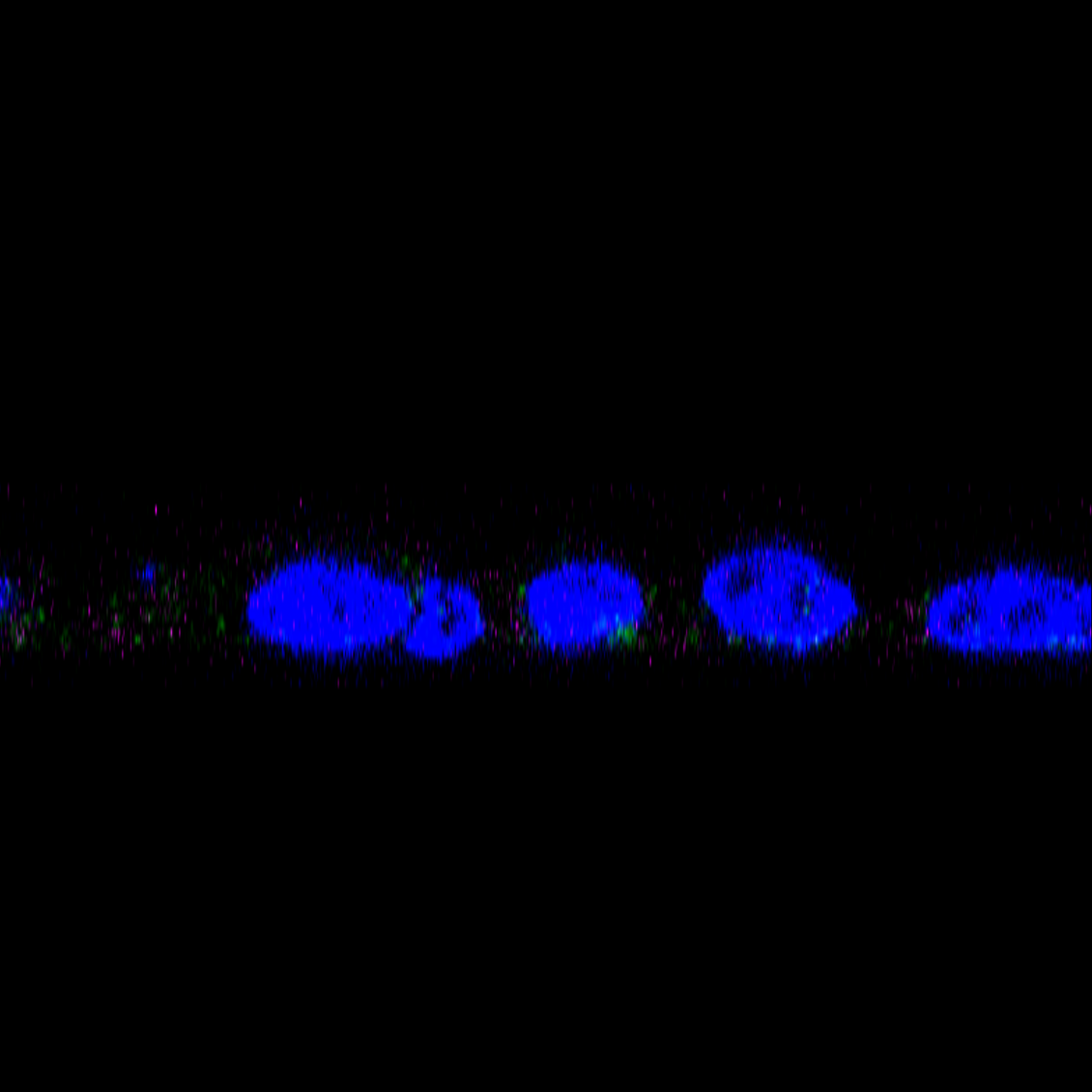

Supplement: Supplementary file 5 — Source Data Fig. 4 [file 44319_2023_18_MOESM5_ESM.zip › Figure_3/3C/3C xz images/0h_left_merge.tif]

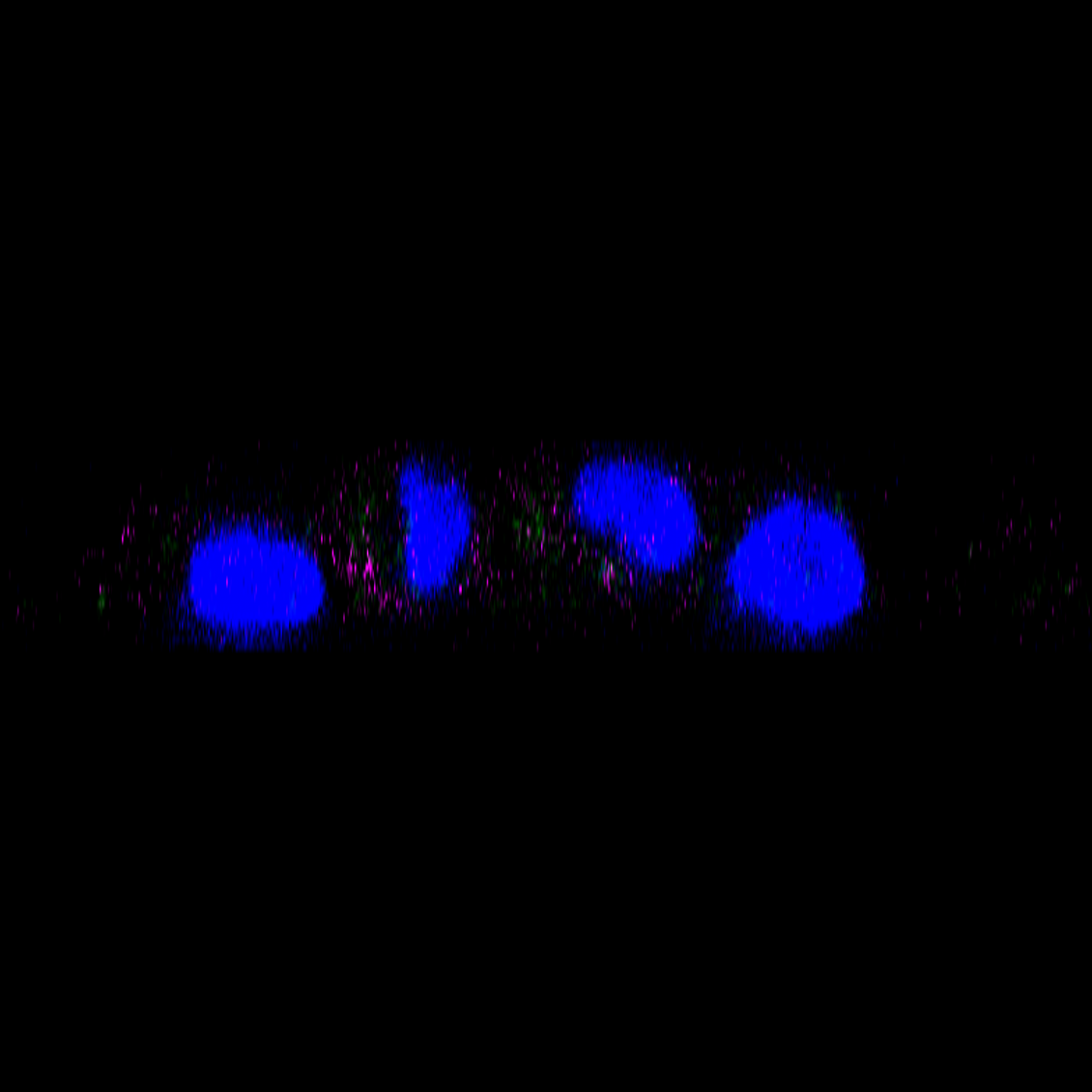

Supplement: Supplementary file 5 — Source Data Fig. 4 [file 44319_2023_18_MOESM5_ESM.zip › Figure_3/3C/3C xz images/0h_middle_merge.tif]

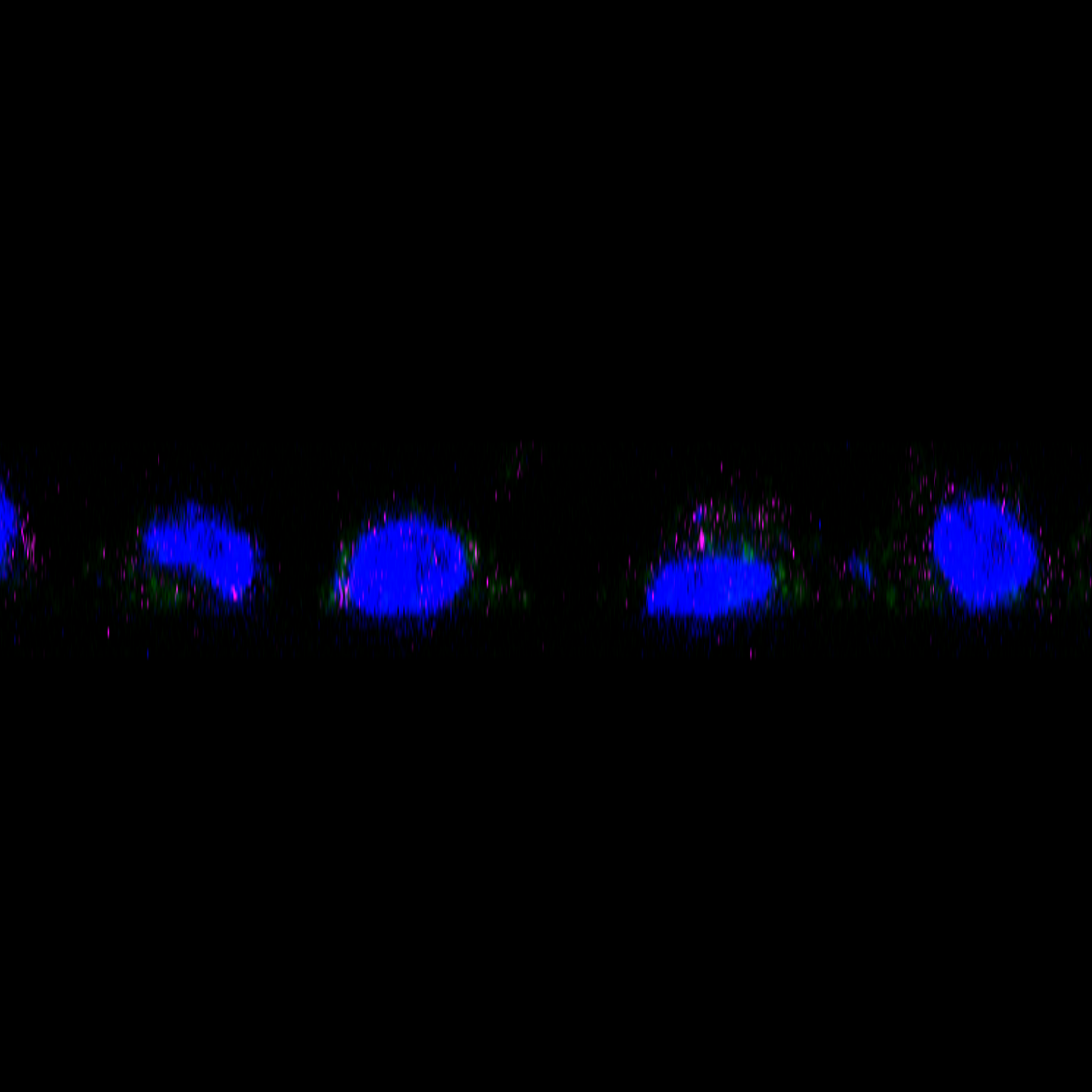

Supplement: Supplementary file 5 — Source Data Fig. 4 [file 44319_2023_18_MOESM5_ESM.zip › Figure_3/3C/3C xz images/0h_right_merge.tif]

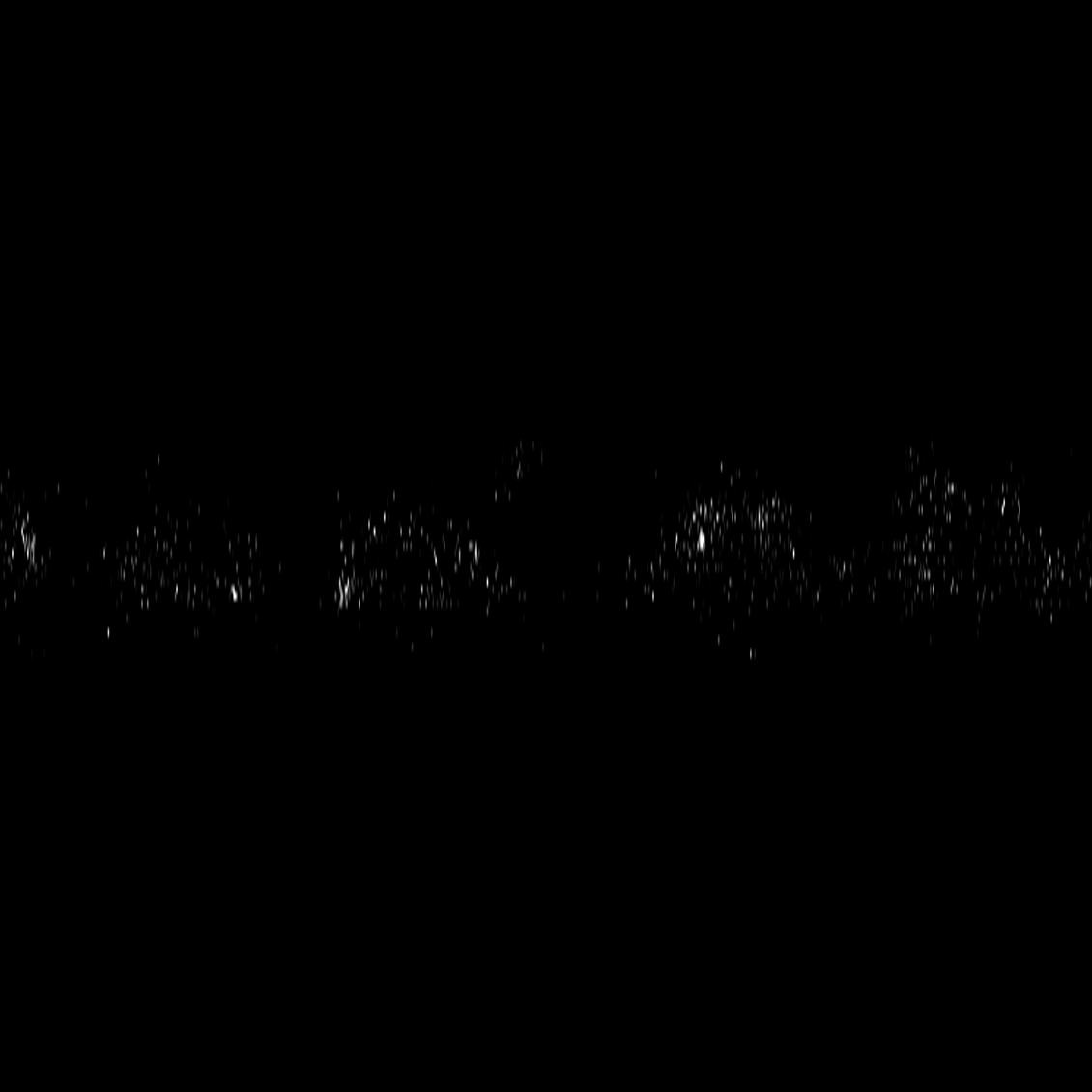

Supplement: Supplementary file 5 — Source Data Fig. 4 [file 44319_2023_18_MOESM5_ESM.zip › Figure_3/3C/3C xz images/0h_right_ZO1.tif]

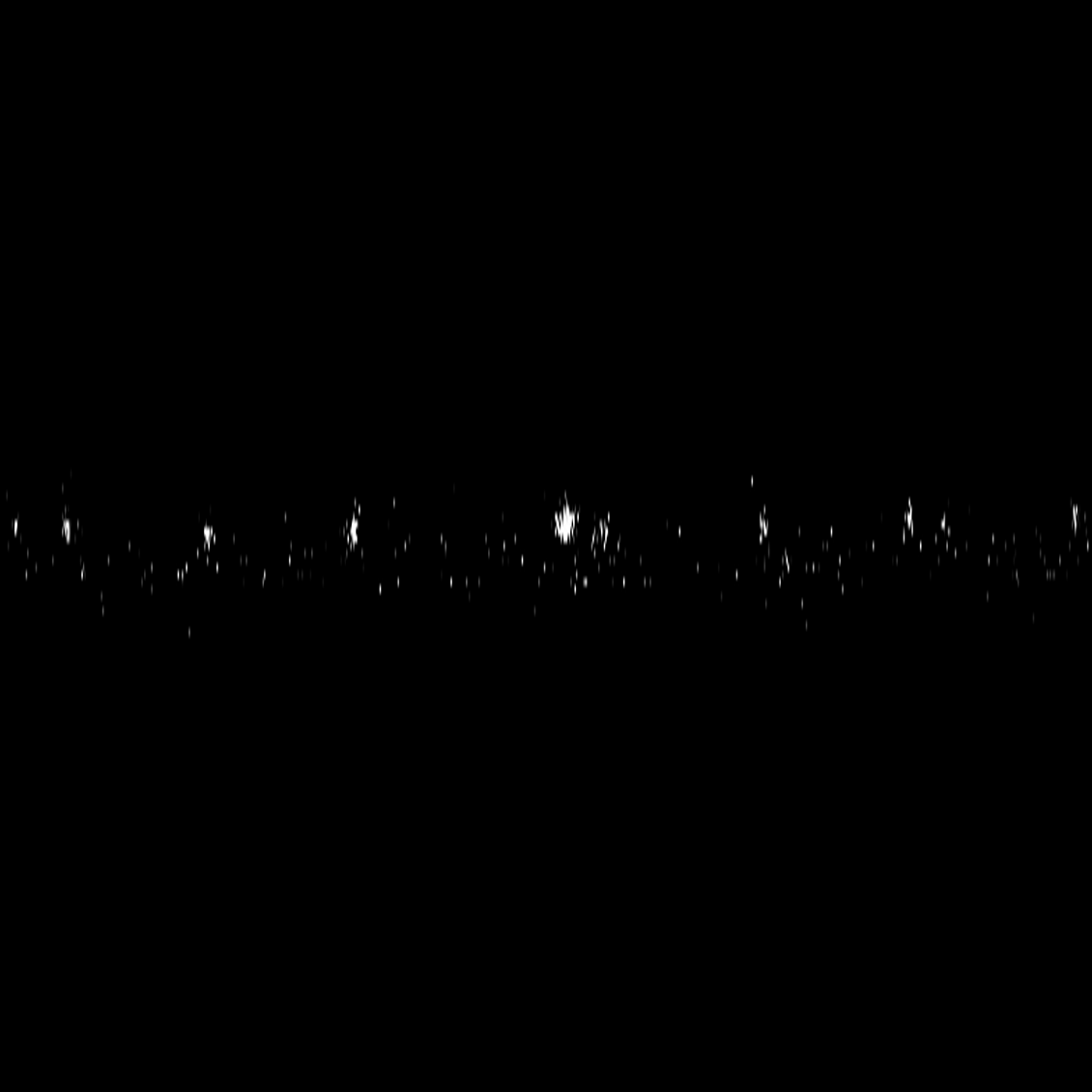

Supplement: Supplementary file 5 — Source Data Fig. 4 [file 44319_2023_18_MOESM5_ESM.zip › Figure_3/3C/3C xz images/6h_middle_ZO1.tif]

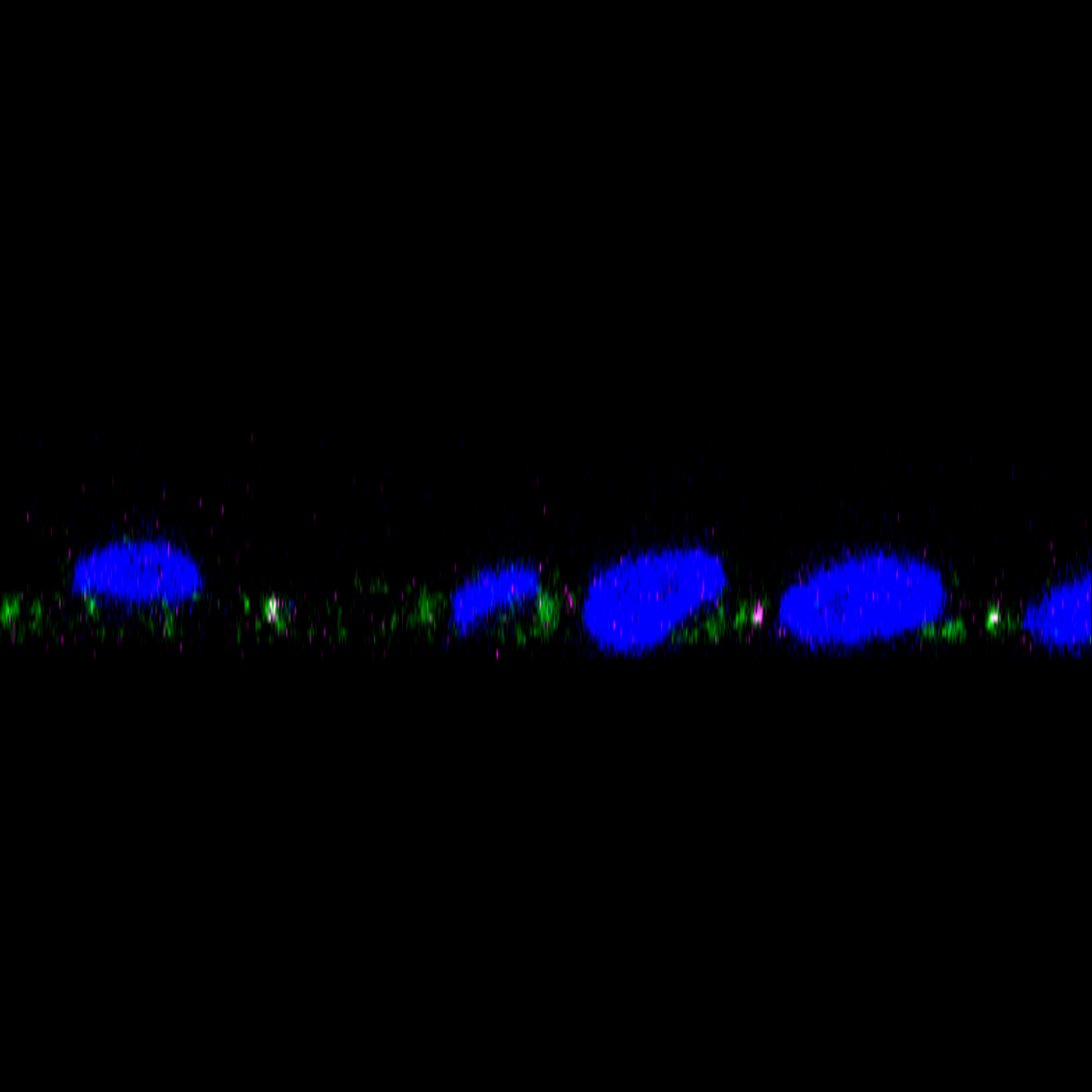

Supplement: Supplementary file 5 — Source Data Fig. 4 [file 44319_2023_18_MOESM5_ESM.zip › Figure_3/3C/3C xz images/2h_left_merge.tif]

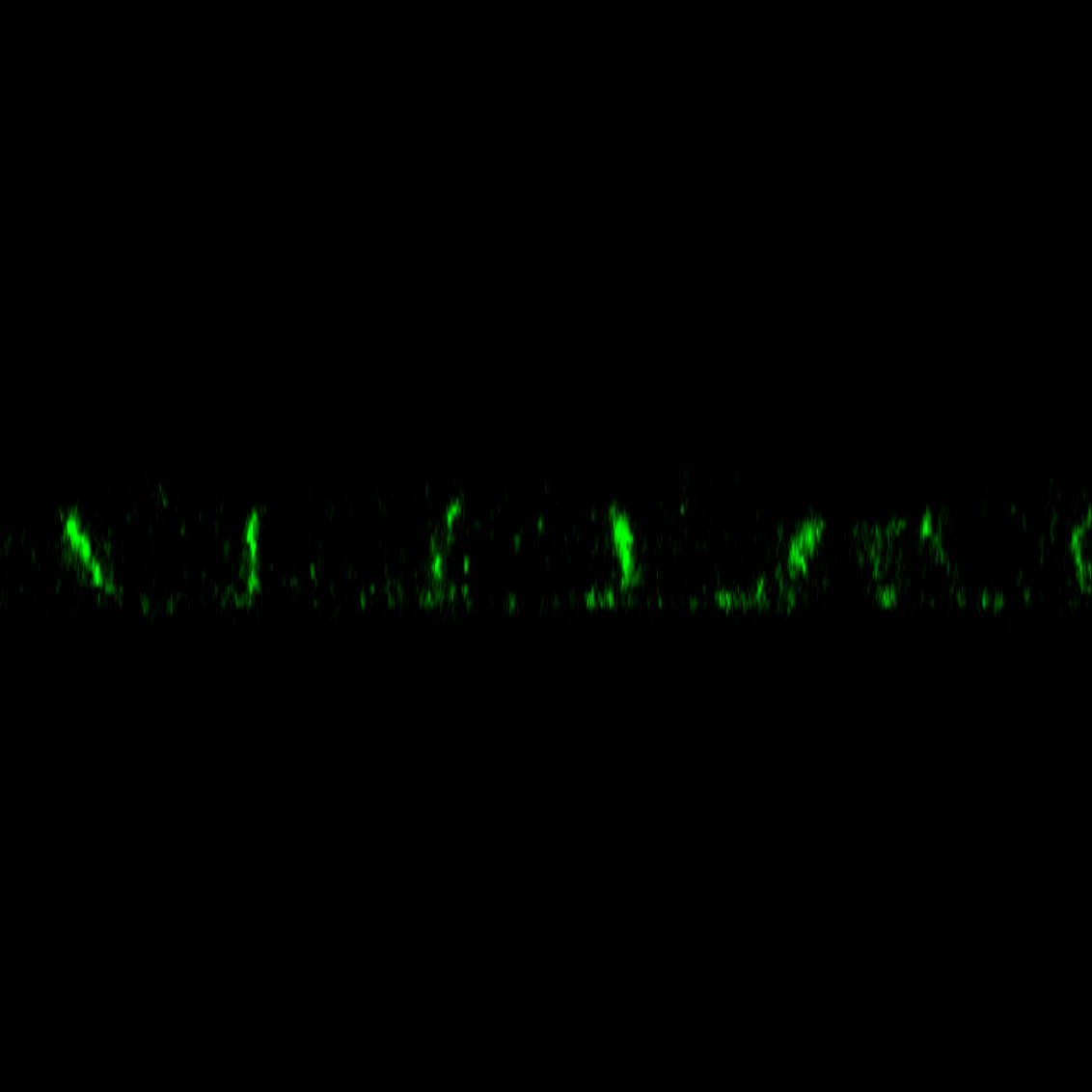

Supplement: Supplementary file 5 — Source Data Fig. 4 [file 44319_2023_18_MOESM5_ESM.zip › Figure_3/3C/3C xz images/6h_left_Ecad.tif]

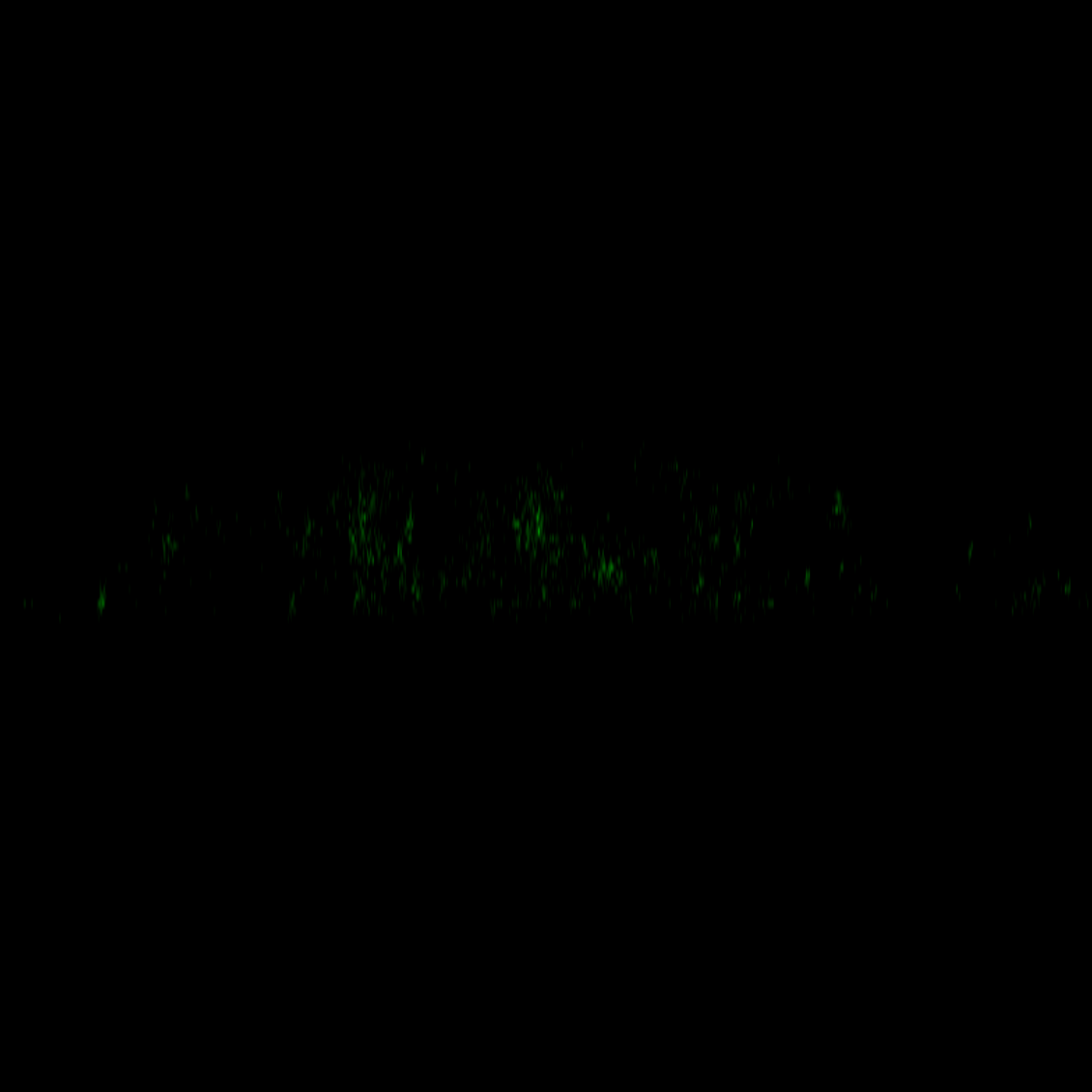

Supplement: Supplementary file 5 — Source Data Fig. 4 [file 44319_2023_18_MOESM5_ESM.zip › Figure_3/3C/3C xz images/0h_middle_Ecad.tif]

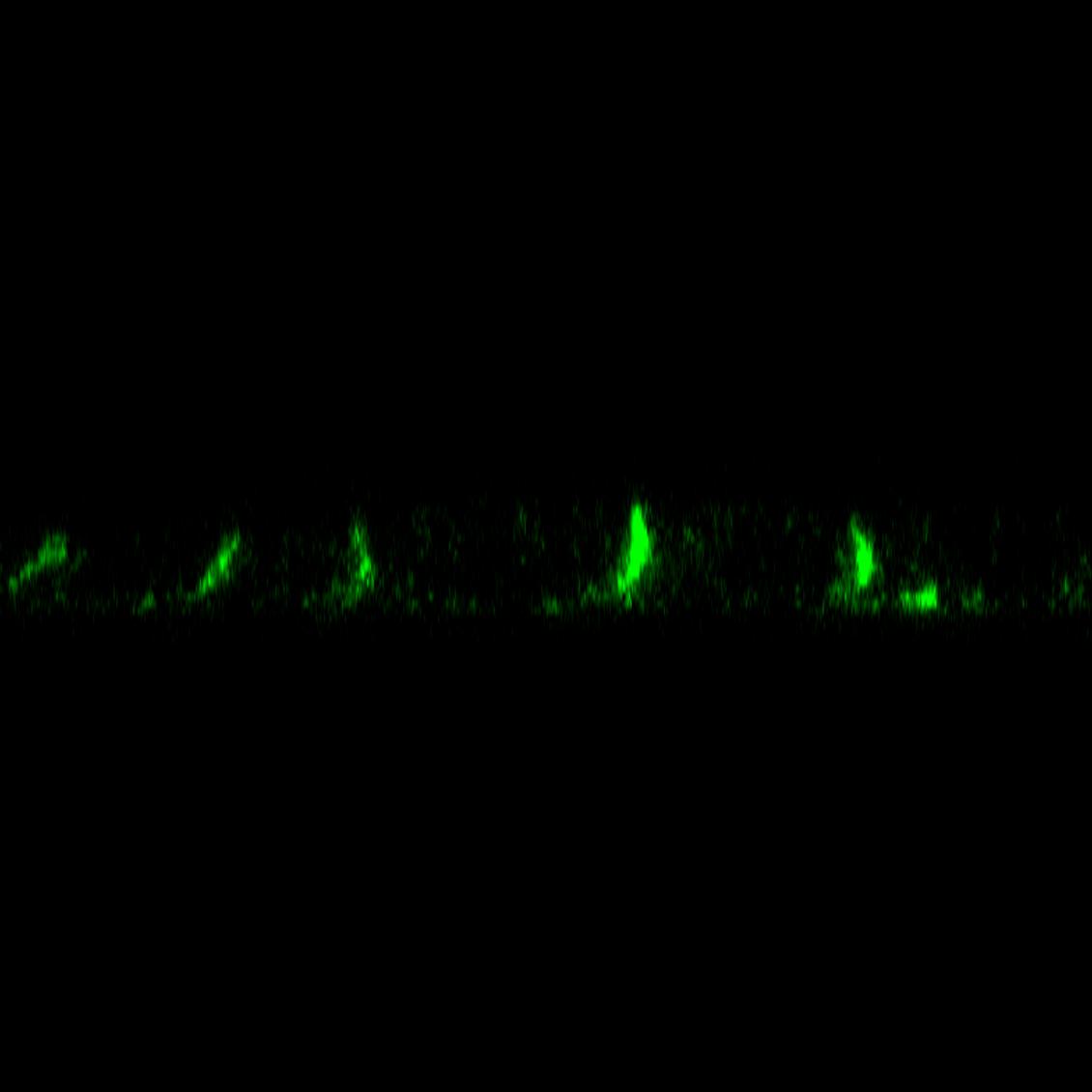

Supplement: Supplementary file 5 — Source Data Fig. 4 [file 44319_2023_18_MOESM5_ESM.zip › Figure_3/3C/3C xz images/6h_right_Ecad.tif]

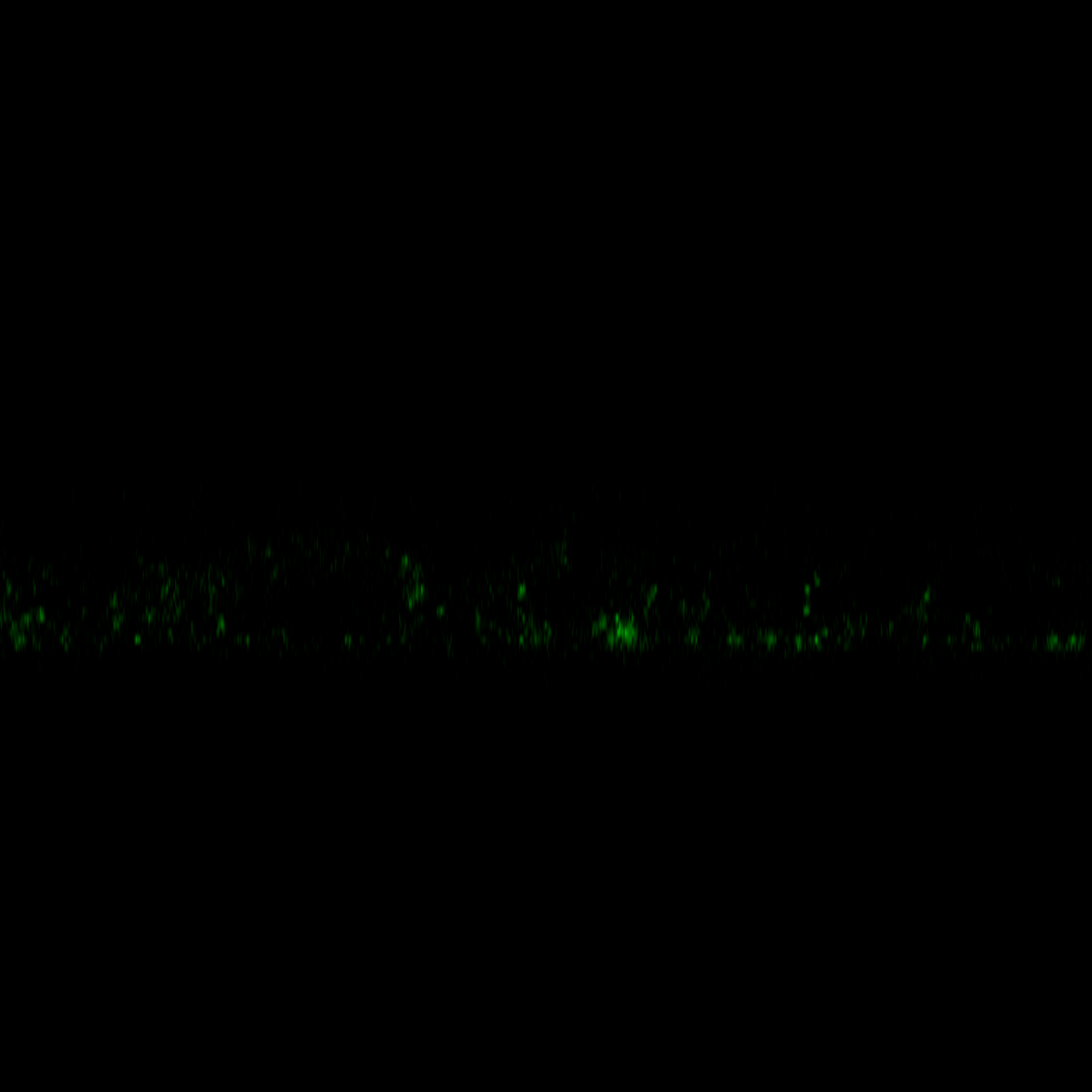

Supplement: Supplementary file 5 — Source Data Fig. 4 [file 44319_2023_18_MOESM5_ESM.zip › Figure_3/3C/3C xz images/0h_left_Ecad.tif]

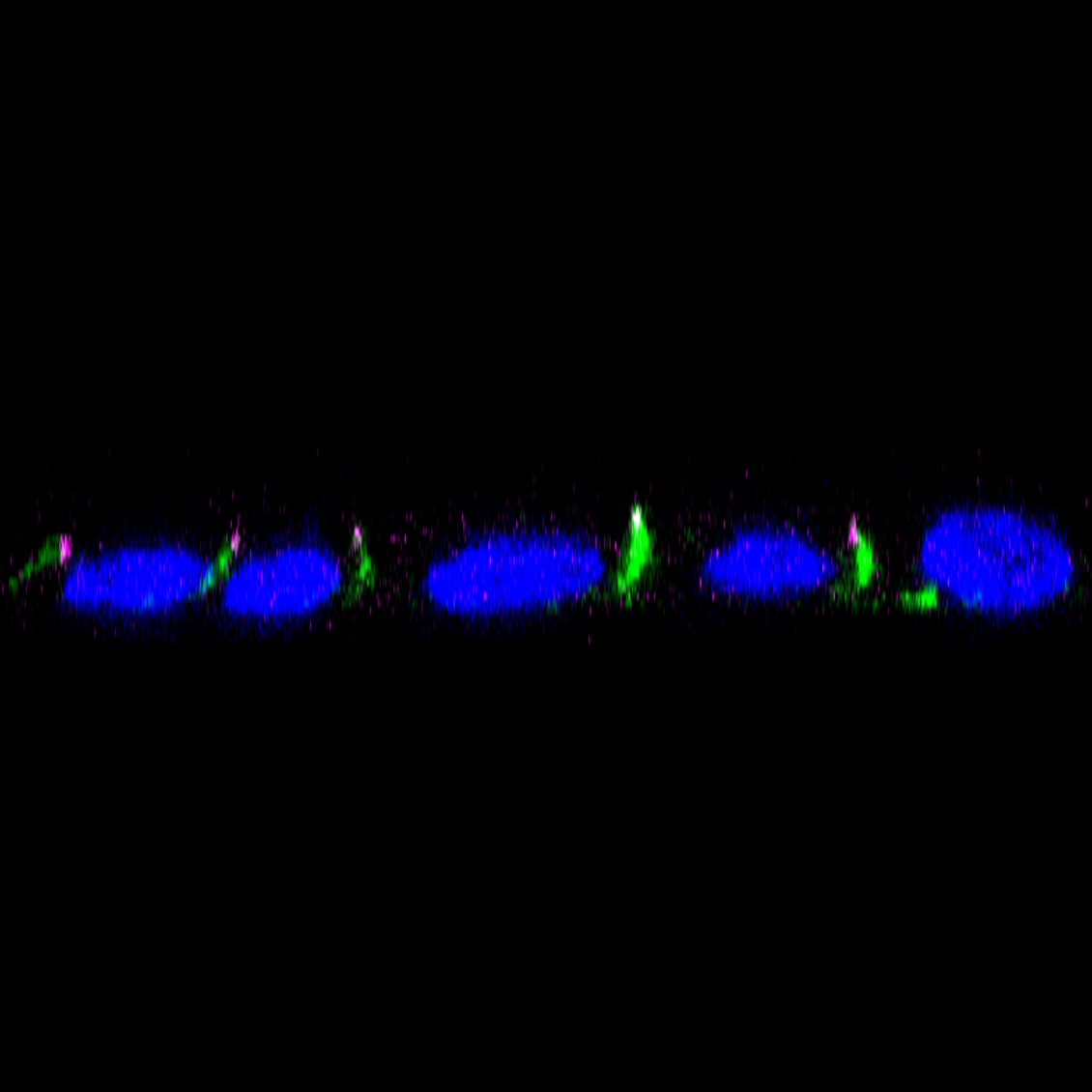

Supplement: Supplementary file 5 — Source Data Fig. 4 [file 44319_2023_18_MOESM5_ESM.zip › Figure_3/3C/3C xz images/6h_right_merge.tif]

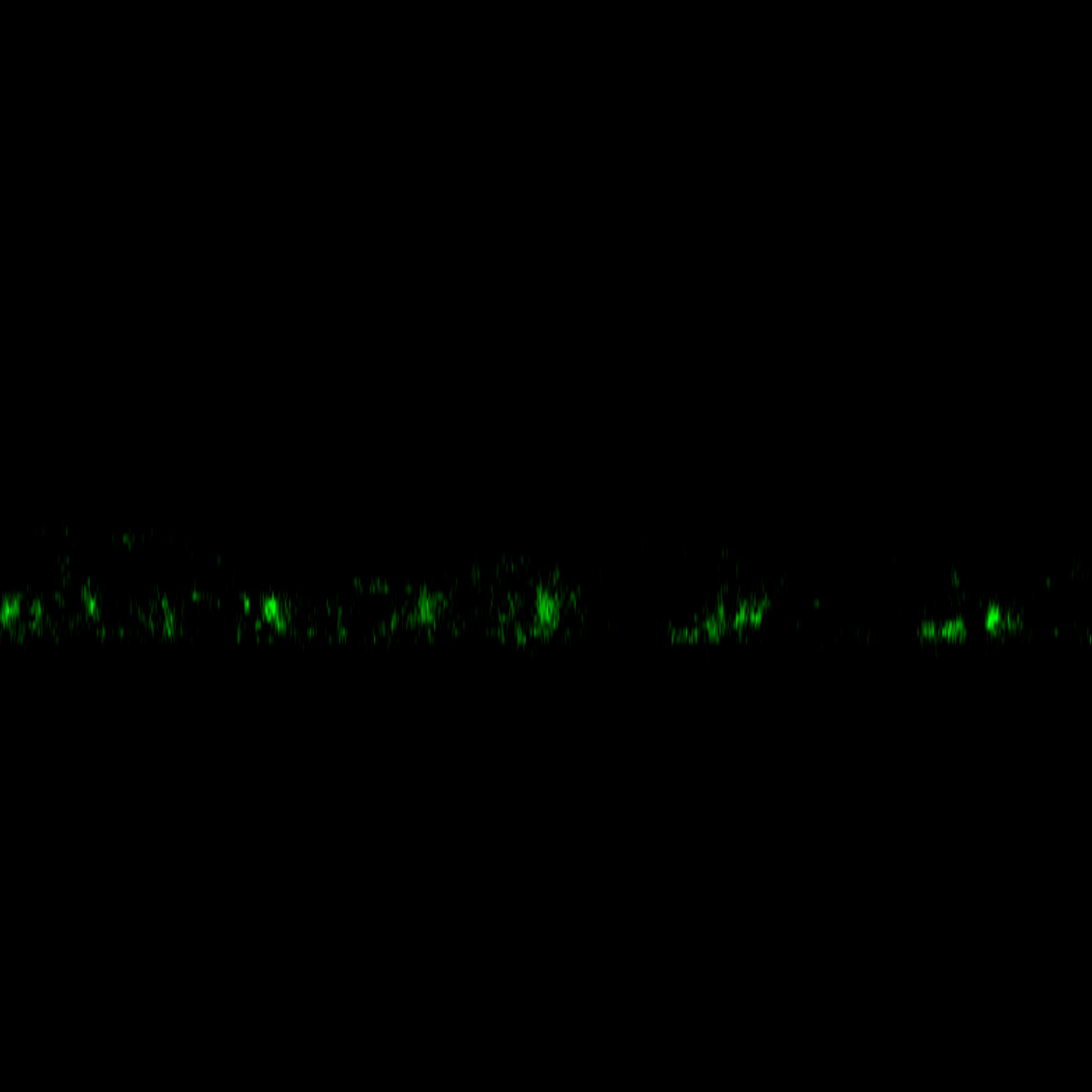

Supplement: Supplementary file 5 — Source Data Fig. 4 [file 44319_2023_18_MOESM5_ESM.zip › Figure_3/3C/3C xz images/2h_left_Ecad.tif]

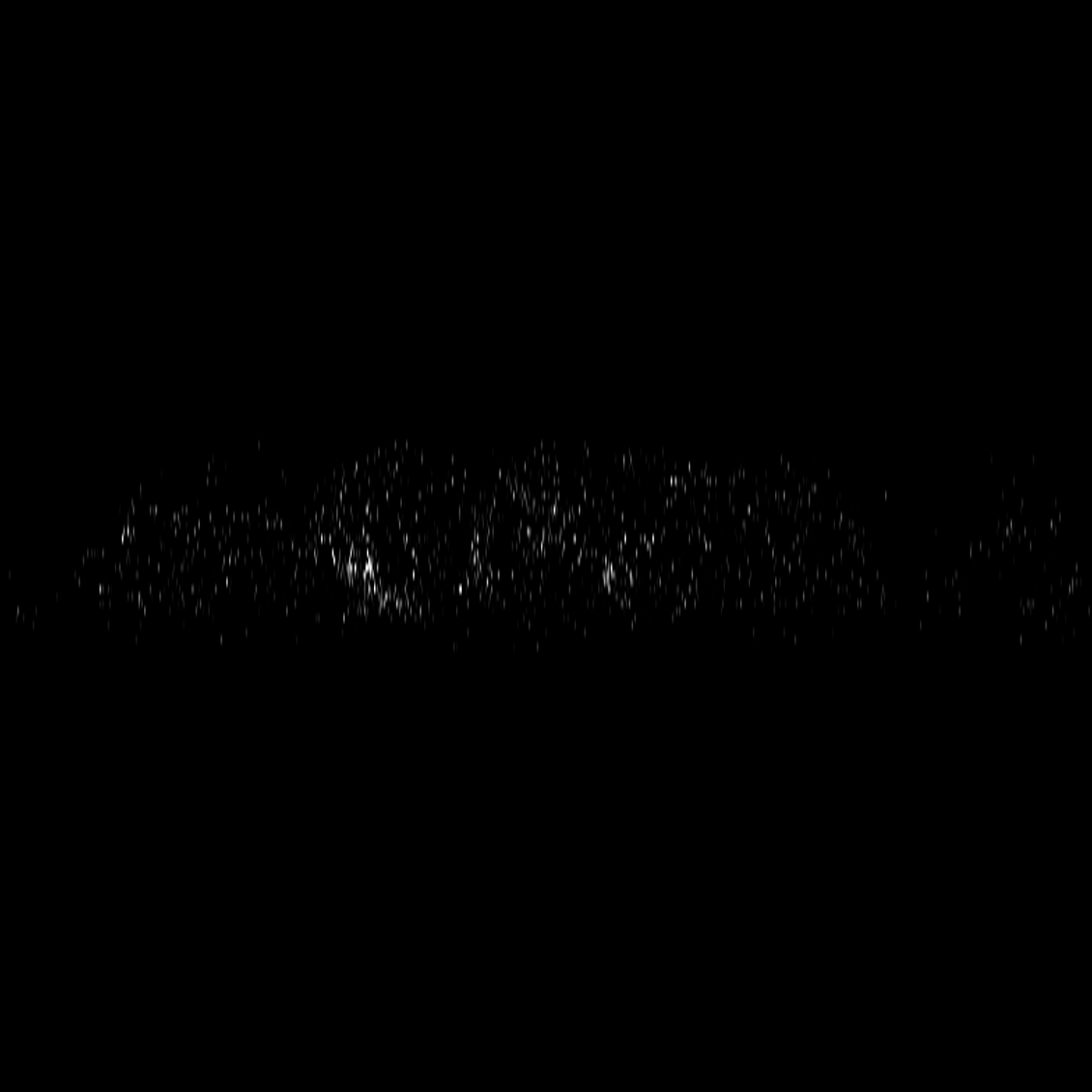

Supplement: Supplementary file 5 — Source Data Fig. 4 [file 44319_2023_18_MOESM5_ESM.zip › Figure_3/3C/3C xz images/0h_middle_ZO1.tif]

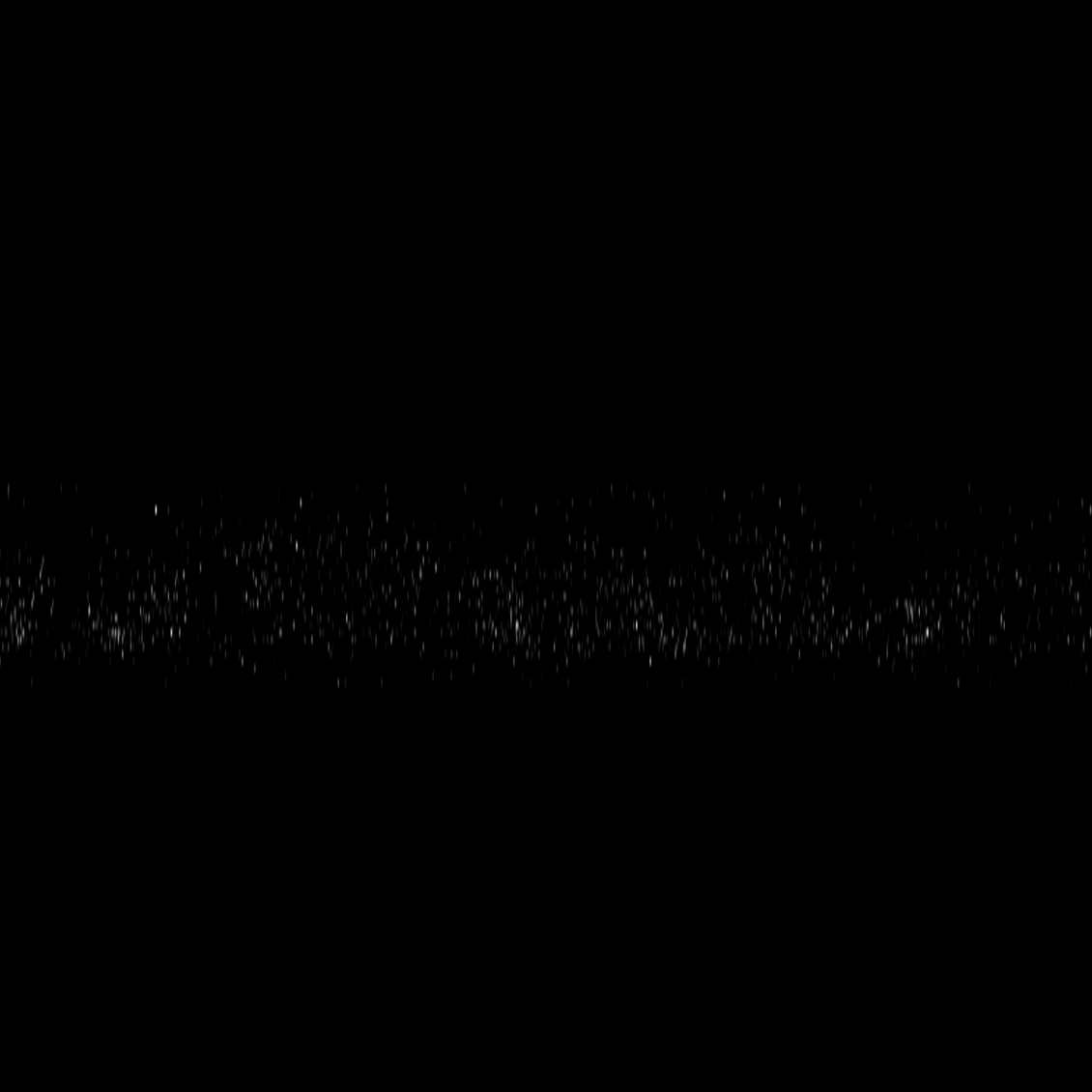

Supplement: Supplementary file 5 — Source Data Fig. 4 [file 44319_2023_18_MOESM5_ESM.zip › Figure_3/3C/3C xz images/0h_left_ZO1.tif]

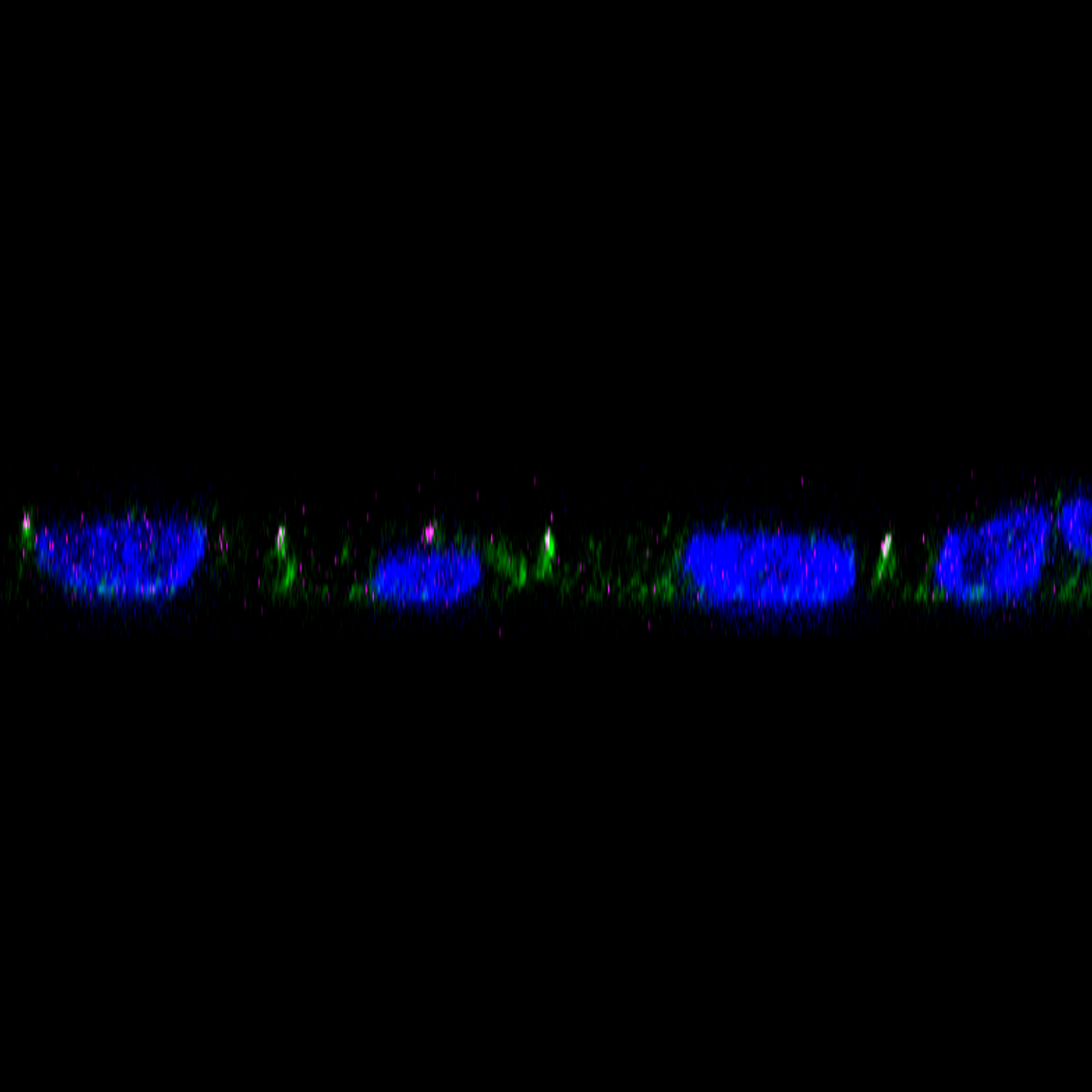

Supplement: Supplementary file 5 — Source Data Fig. 4 [file 44319_2023_18_MOESM5_ESM.zip › Figure_3/3C/3C xz images/2h_right_merge.tif]

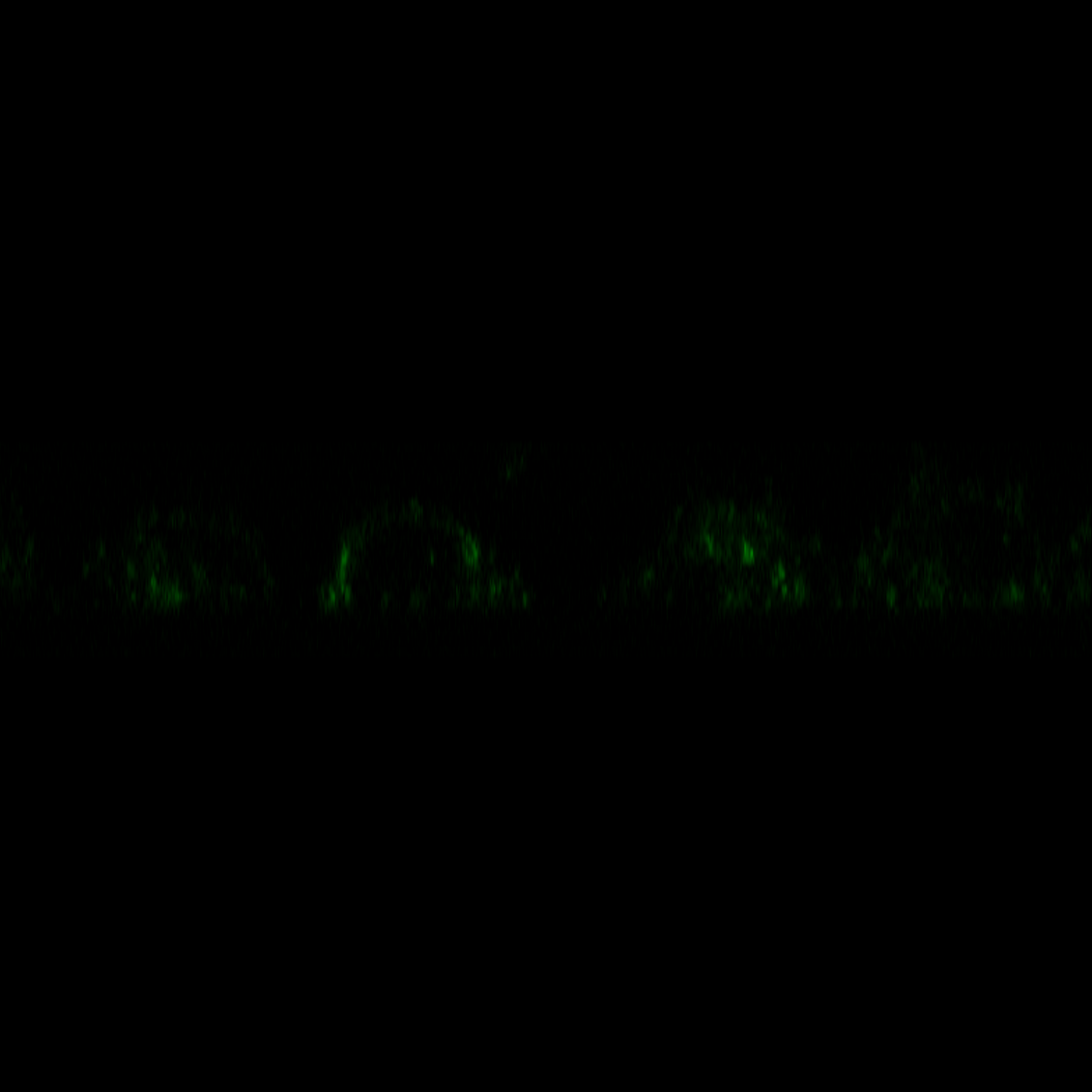

Supplement: Supplementary file 5 — Source Data Fig. 4 [file 44319_2023_18_MOESM5_ESM.zip › Figure_3/3C/3C xz images/0h_right_Ecad.tif]

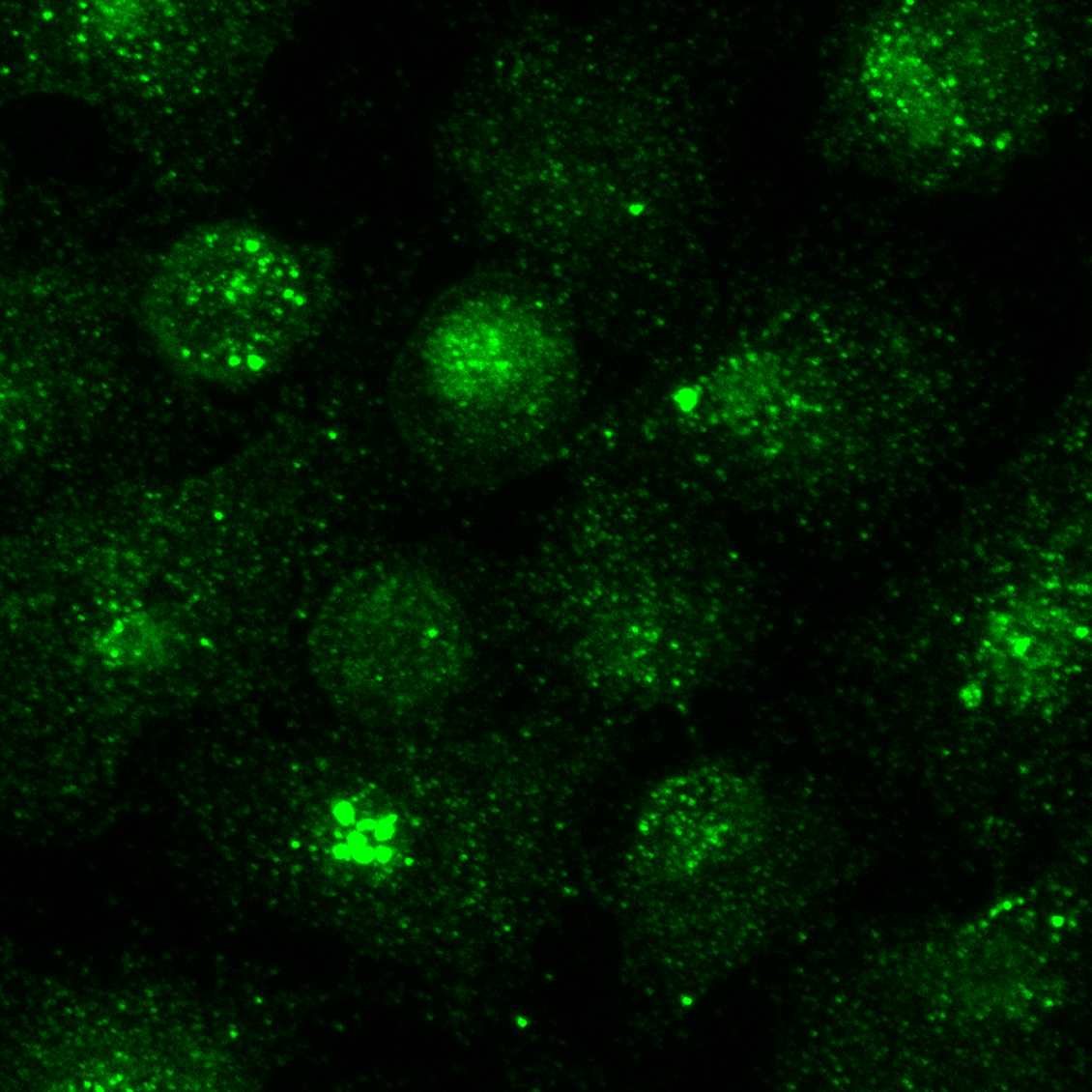

Supplement: Supplementary file 5 — Source Data Fig. 4 [file 44319_2023_18_MOESM5_ESM.zip › Figure_3/3D/3D image data/0h_right_Cldn1.tif]

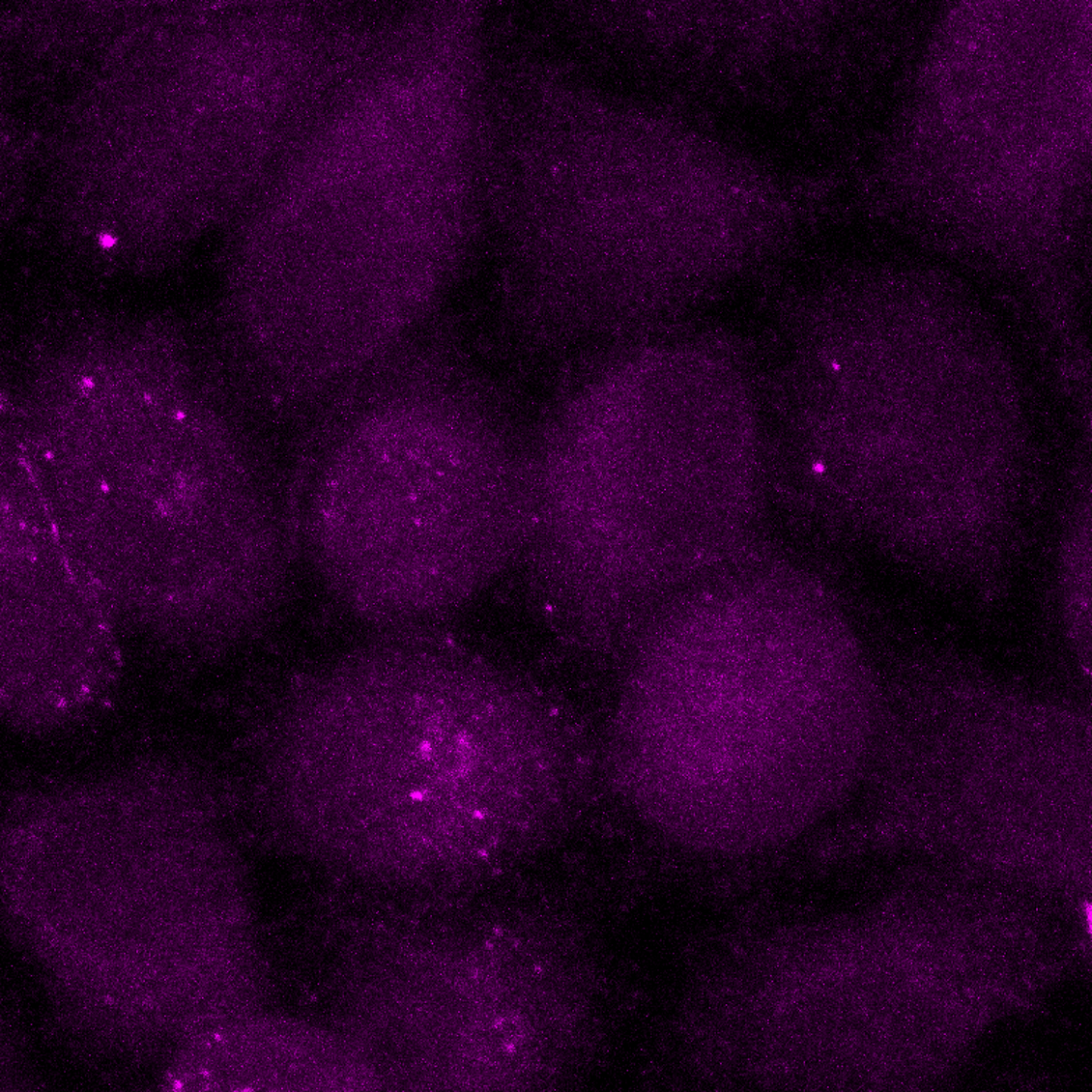

Supplement: Supplementary file 5 — Source Data Fig. 4 [file 44319_2023_18_MOESM5_ESM.zip › Figure_3/3D/3D image data/0h_left_Ocln.tif]

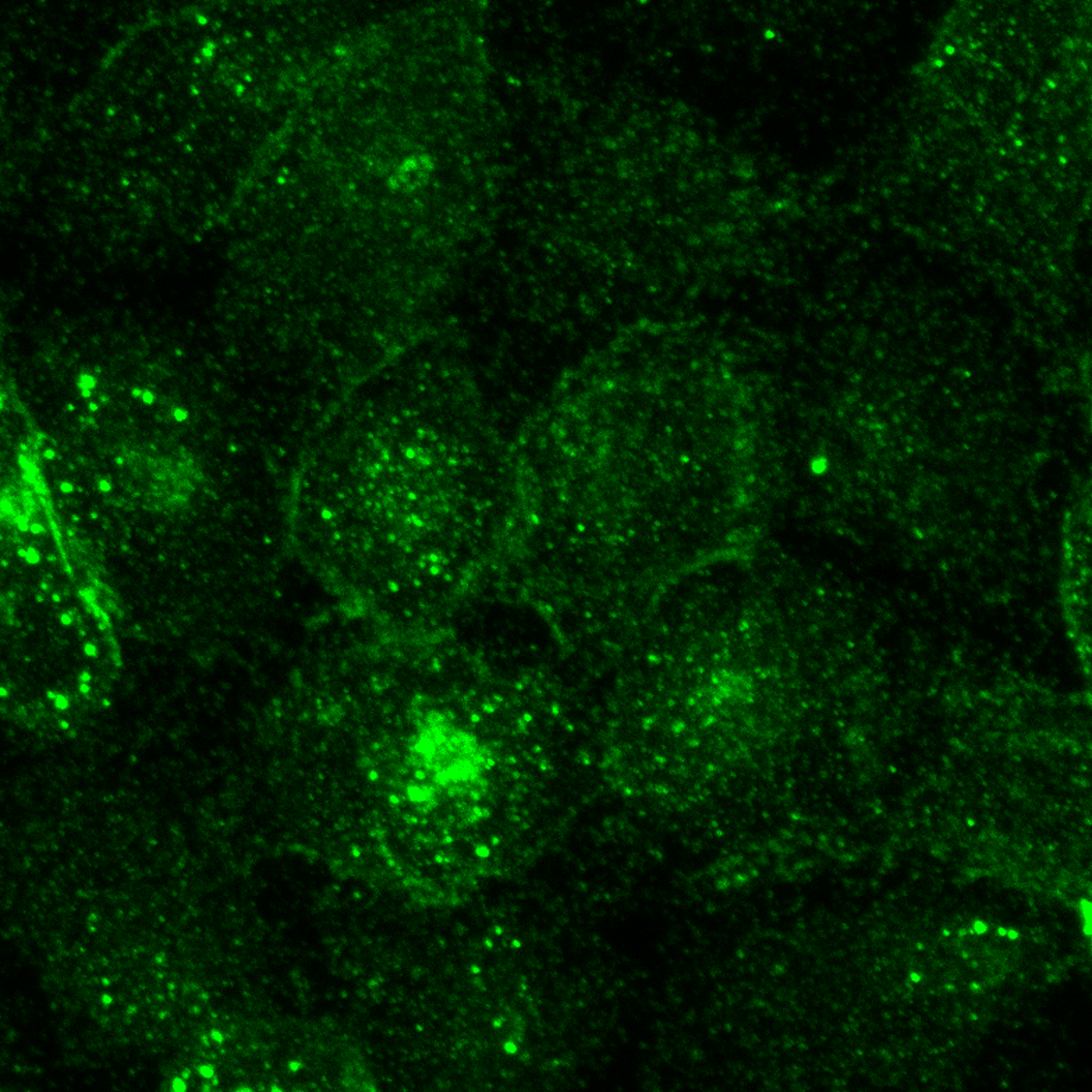

Supplement: Supplementary file 5 — Source Data Fig. 4 [file 44319_2023_18_MOESM5_ESM.zip › Figure_3/3D/3D image data/0h_left_Cldn1.tif]

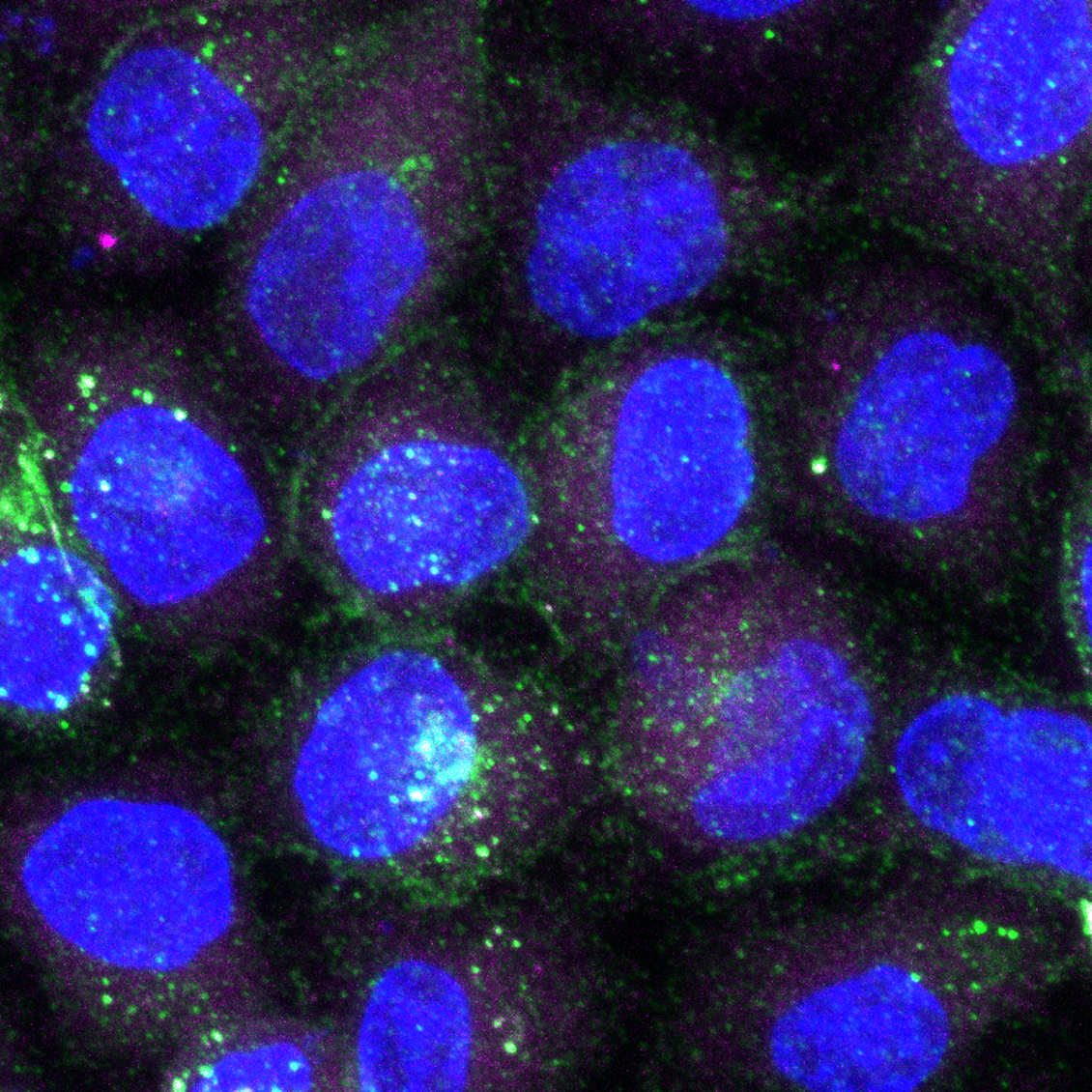

Supplement: Supplementary file 5 — Source Data Fig. 4 [file 44319_2023_18_MOESM5_ESM.zip › Figure_3/3D/3D image data/0h_left_merge.tif]

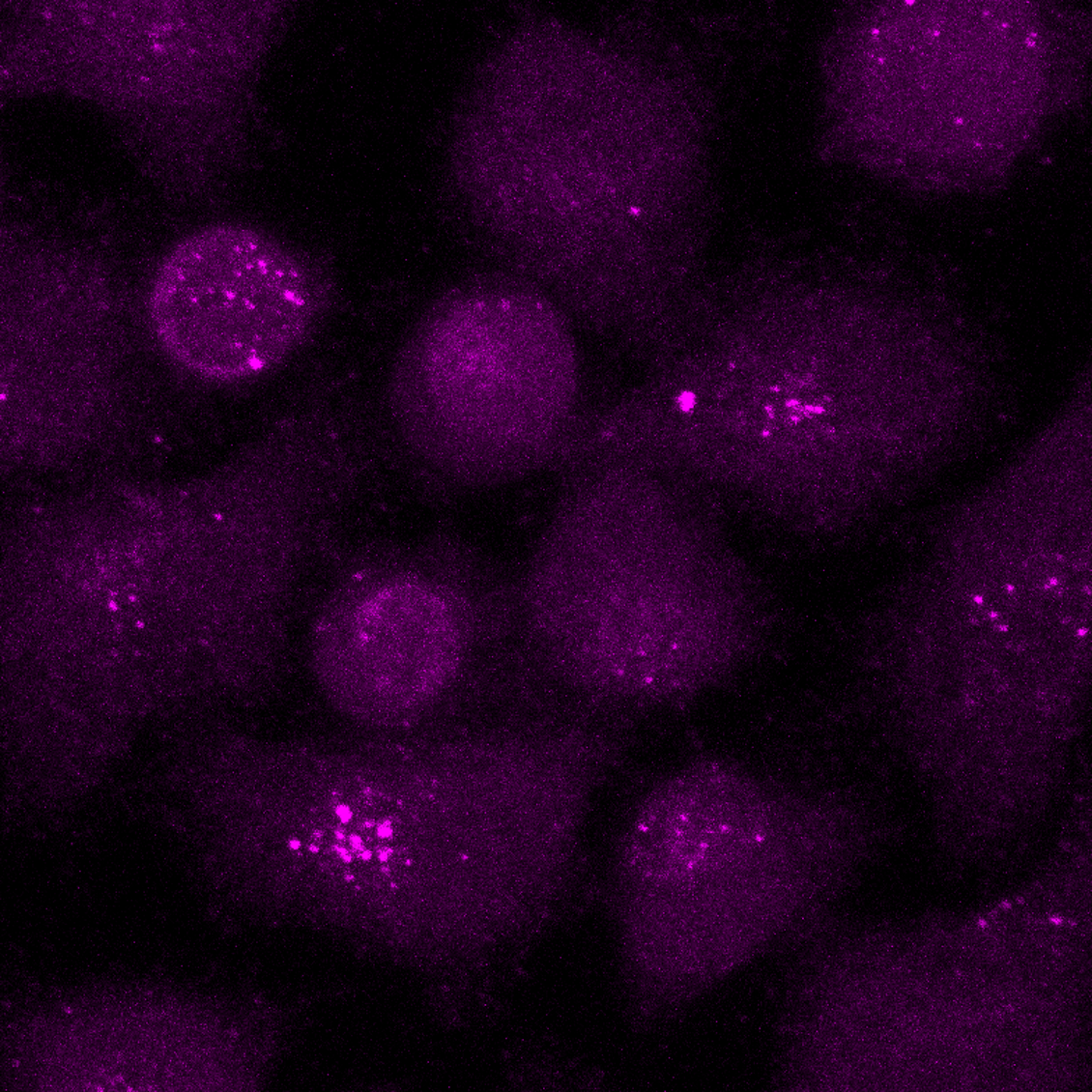

Supplement: Supplementary file 5 — Source Data Fig. 4 [file 44319_2023_18_MOESM5_ESM.zip › Figure_3/3D/3D image data/0h_right_Ocln.tif]

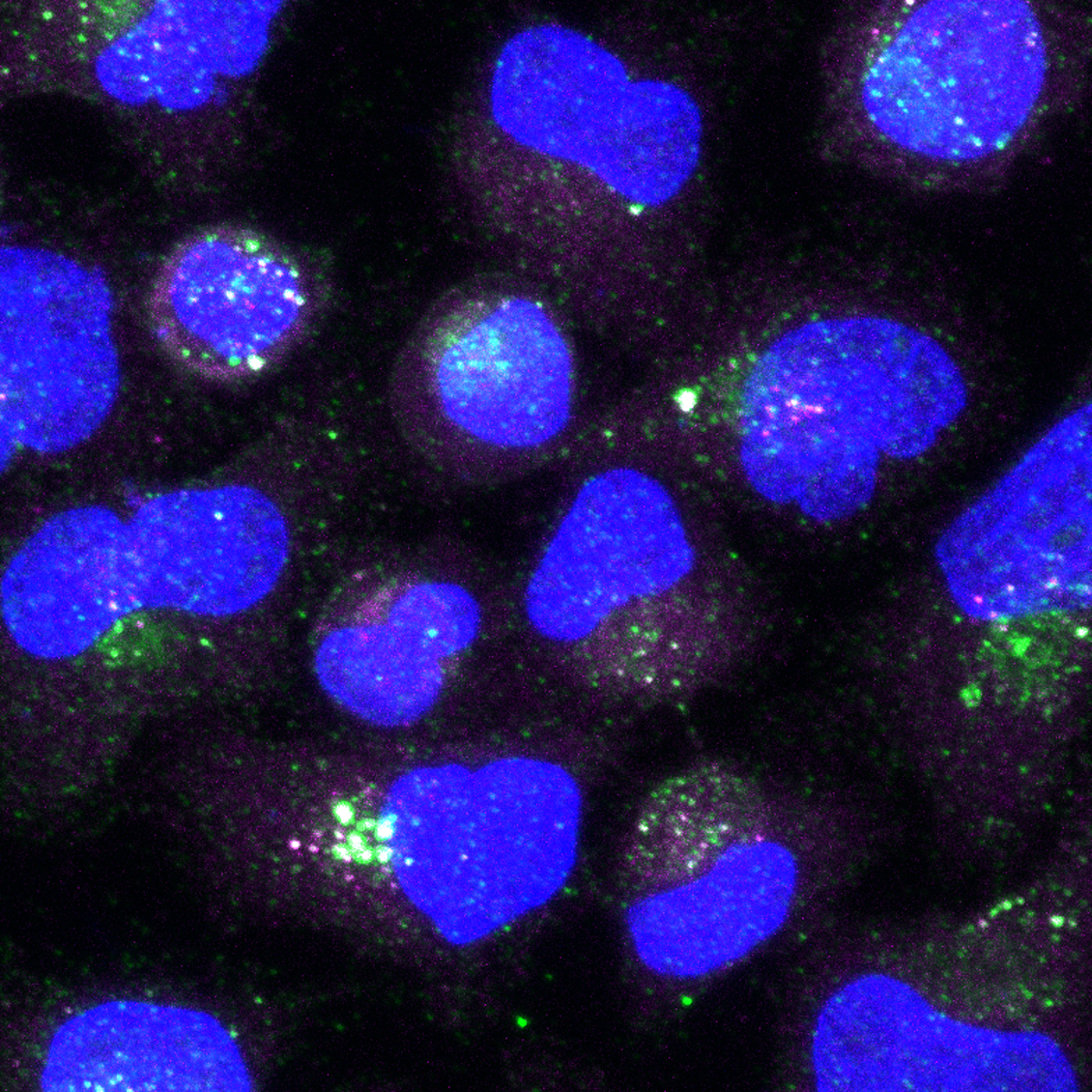

Supplement: Supplementary file 5 — Source Data Fig. 4 [file 44319_2023_18_MOESM5_ESM.zip › Figure_3/3D/3D image data/0h_right_merge.tif]

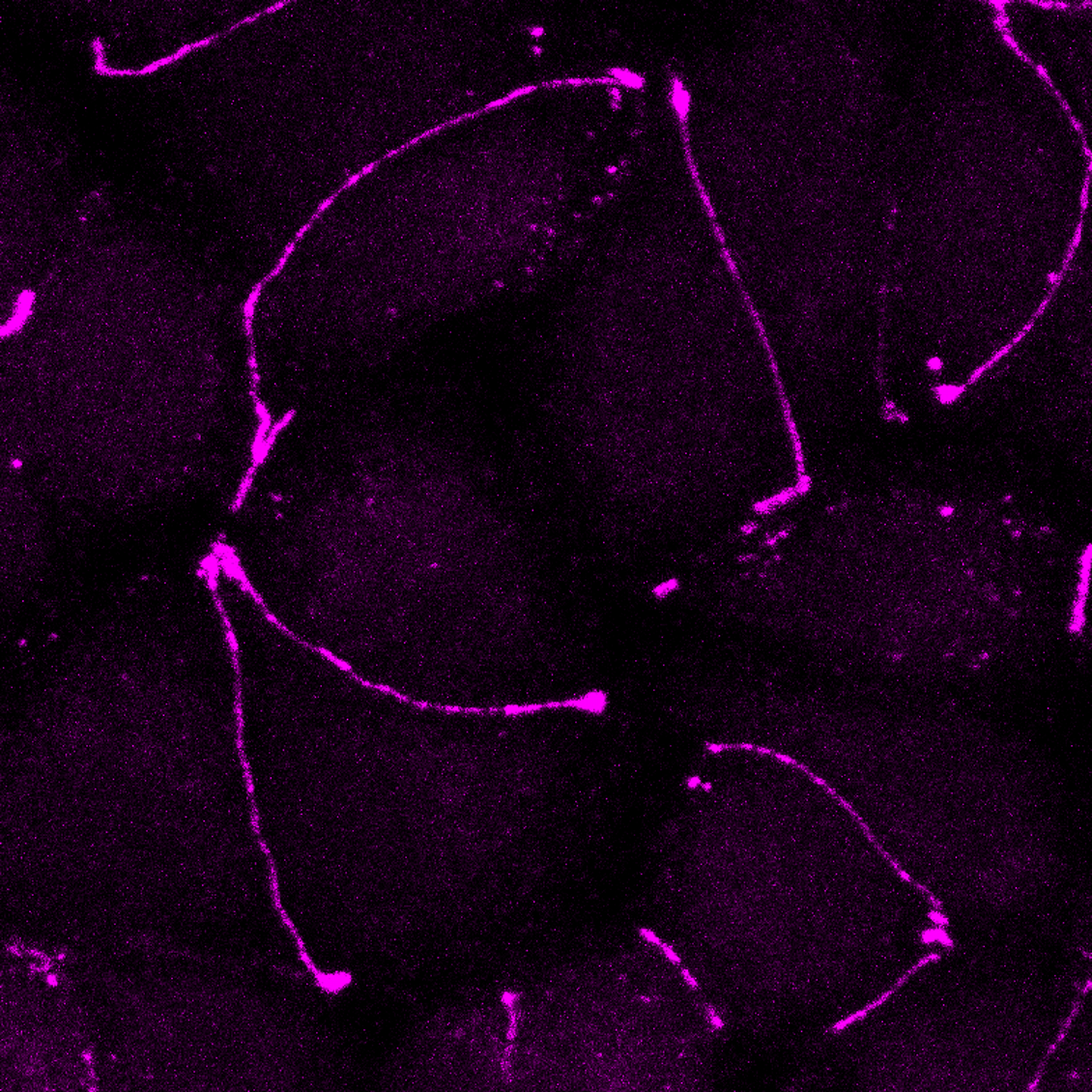

Supplement: Supplementary file 5 — Source Data Fig. 4 [file 44319_2023_18_MOESM5_ESM.zip › Figure_3/3D/3D image data/2h_left_Ocln.tif]

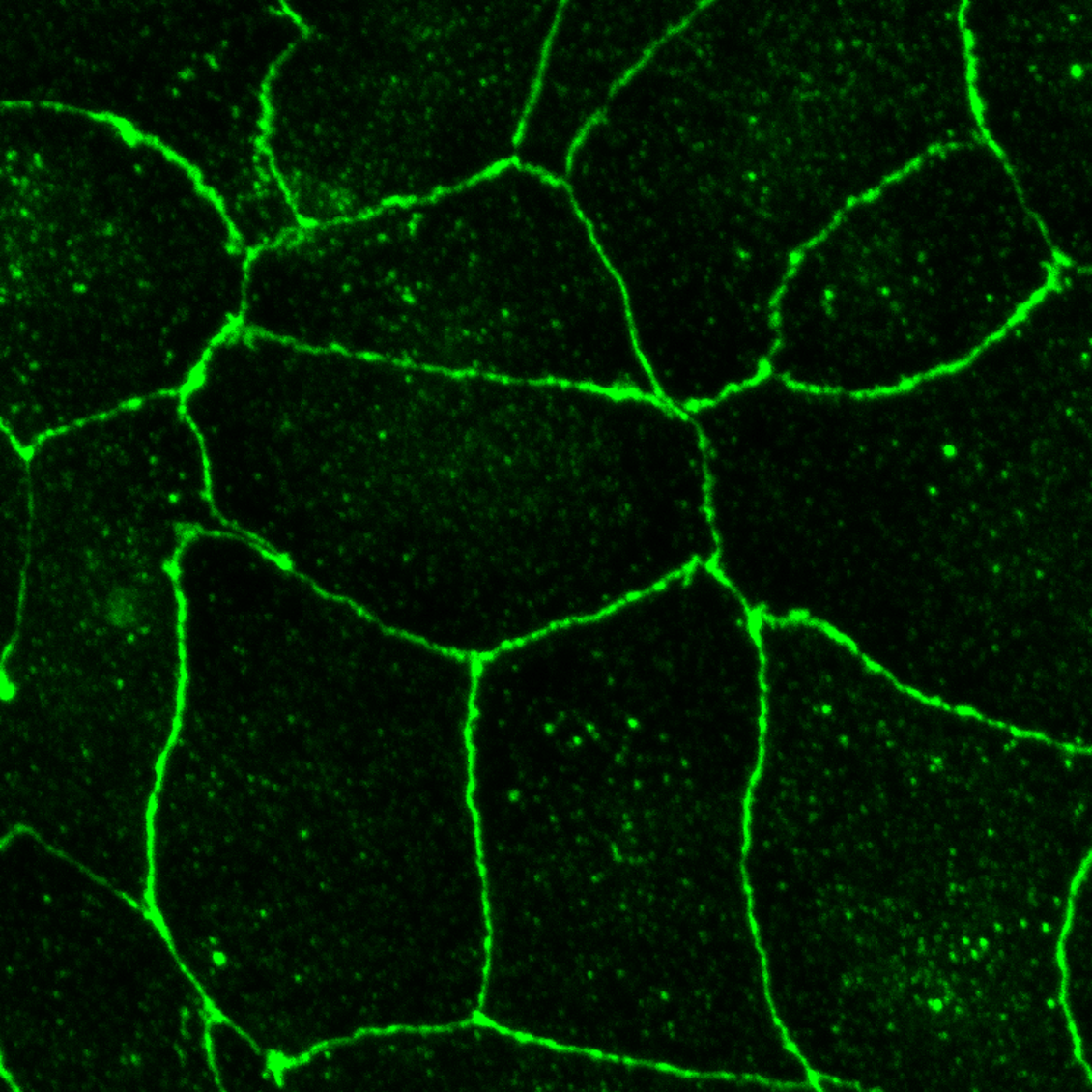

Supplement: Supplementary file 5 — Source Data Fig. 4 [file 44319_2023_18_MOESM5_ESM.zip › Figure_3/3D/3D image data/2h_right_Cldn1.tif]

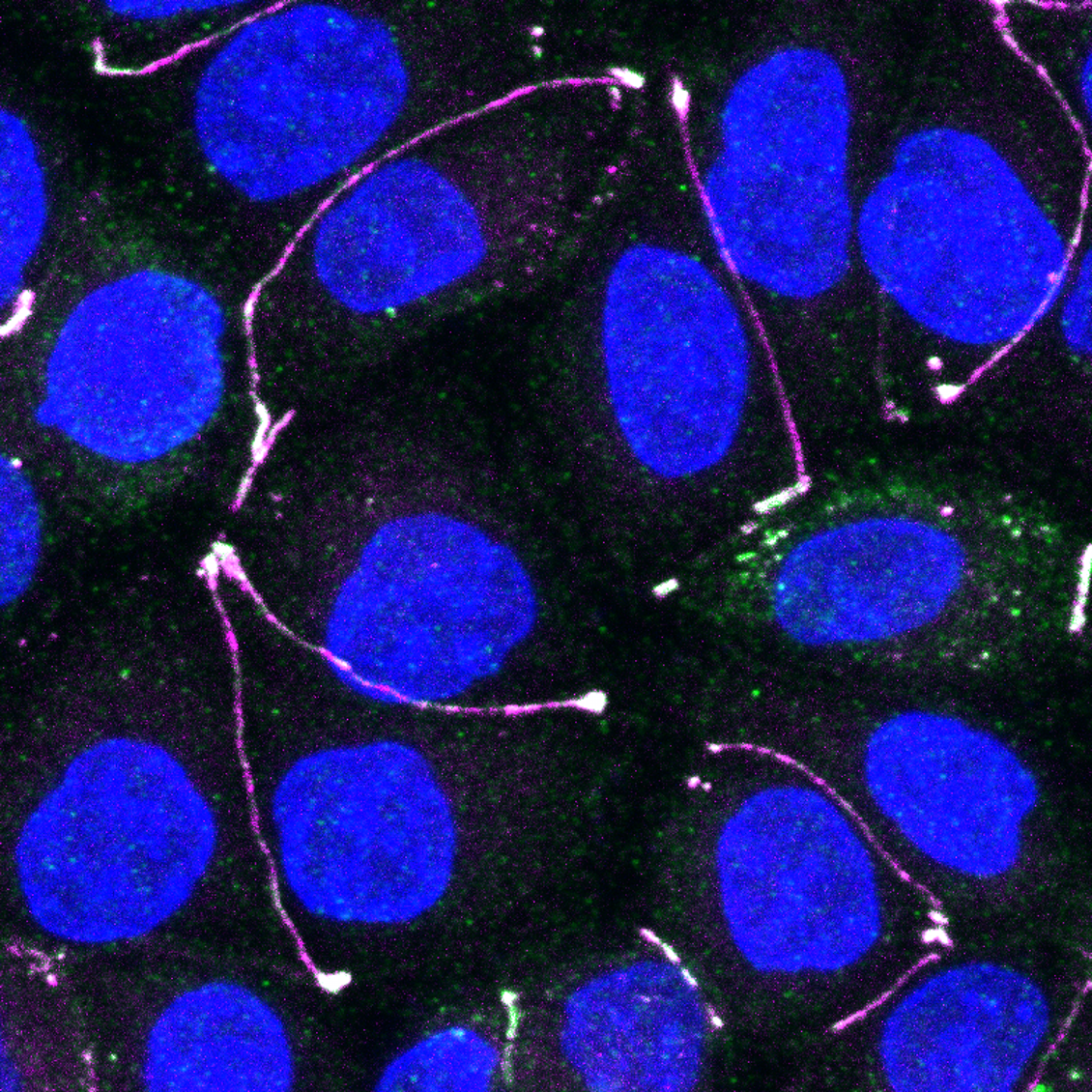

Supplement: Supplementary file 5 — Source Data Fig. 4 [file 44319_2023_18_MOESM5_ESM.zip › Figure_3/3D/3D image data/2h_left_merge.tif]

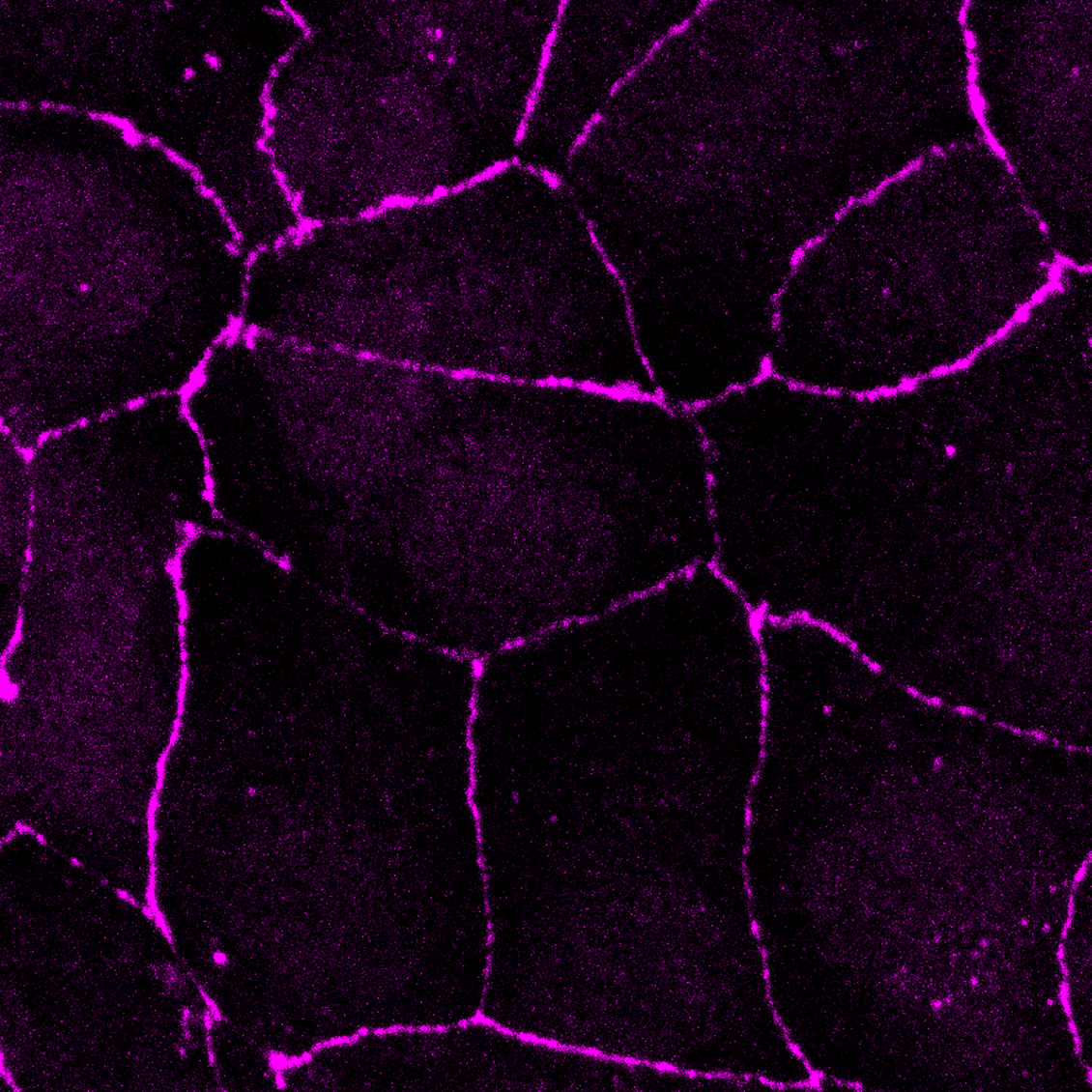

Supplement: Supplementary file 5 — Source Data Fig. 4 [file 44319_2023_18_MOESM5_ESM.zip › Figure_3/3D/3D image data/2h_right_Ocln.tif]

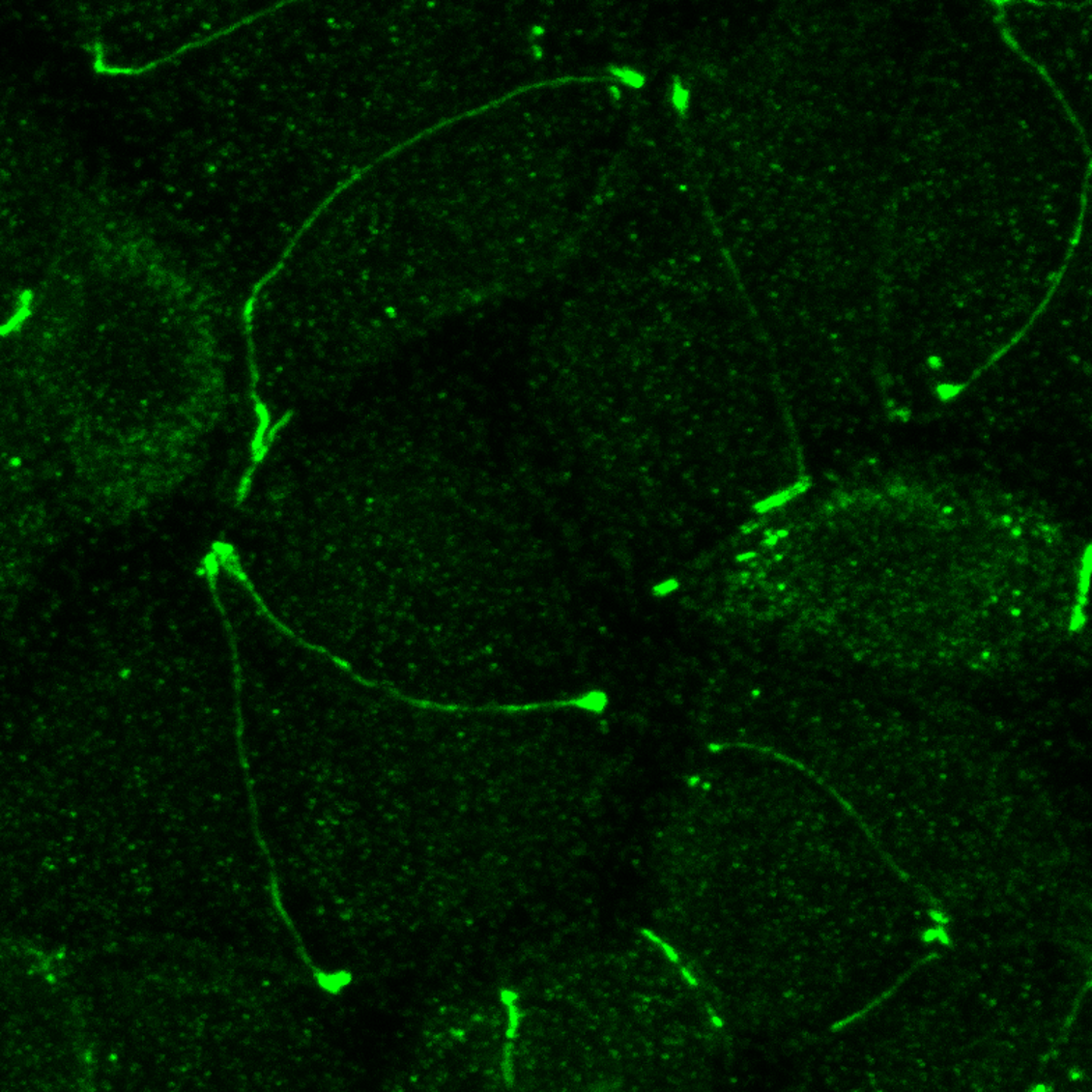

Supplement: Supplementary file 5 — Source Data Fig. 4 [file 44319_2023_18_MOESM5_ESM.zip › Figure_3/3D/3D image data/2h_left_Cldn1.tif]

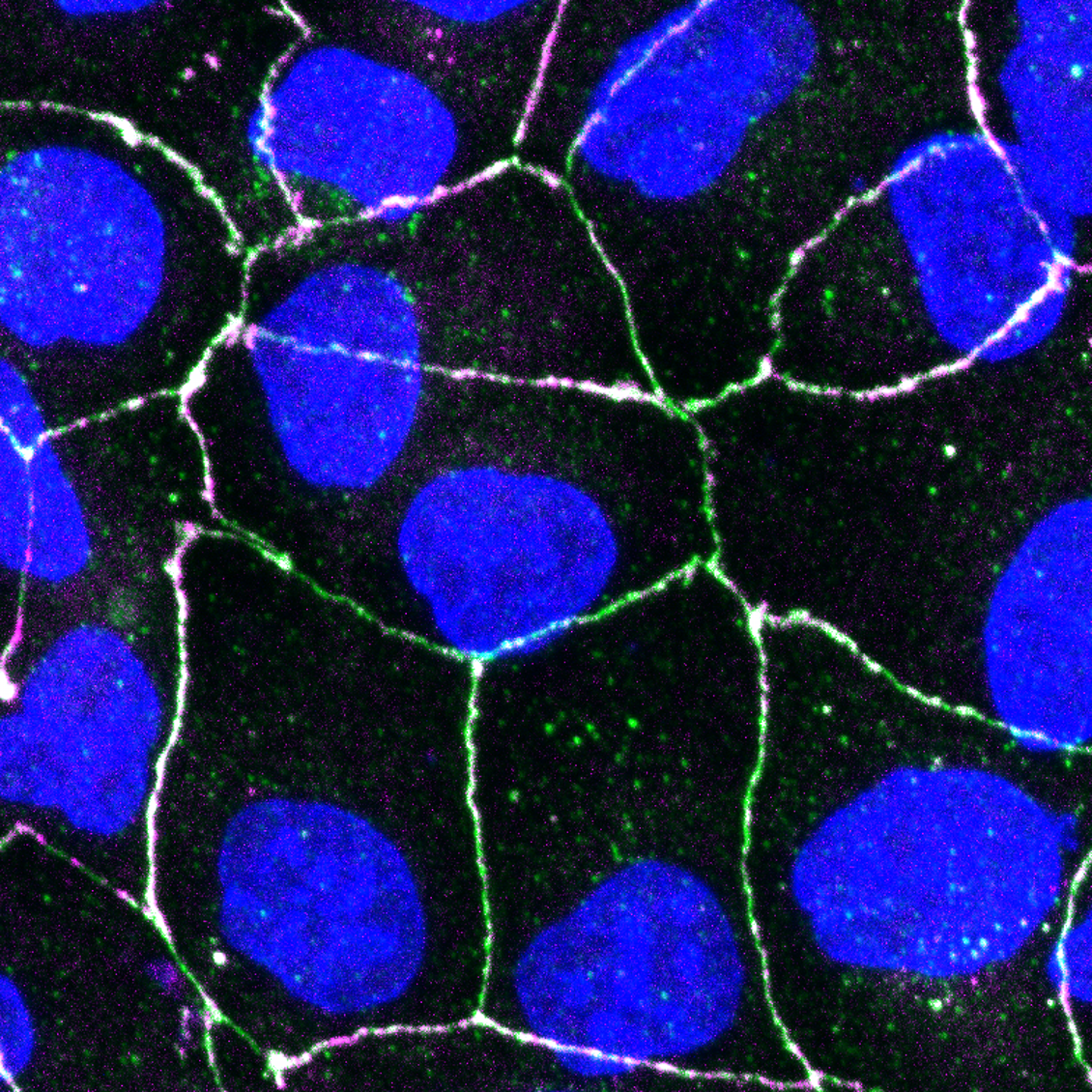

Supplement: Supplementary file 5 — Source Data Fig. 4 [file 44319_2023_18_MOESM5_ESM.zip › Figure_3/3D/3D image data/2h_right_merge.tif]

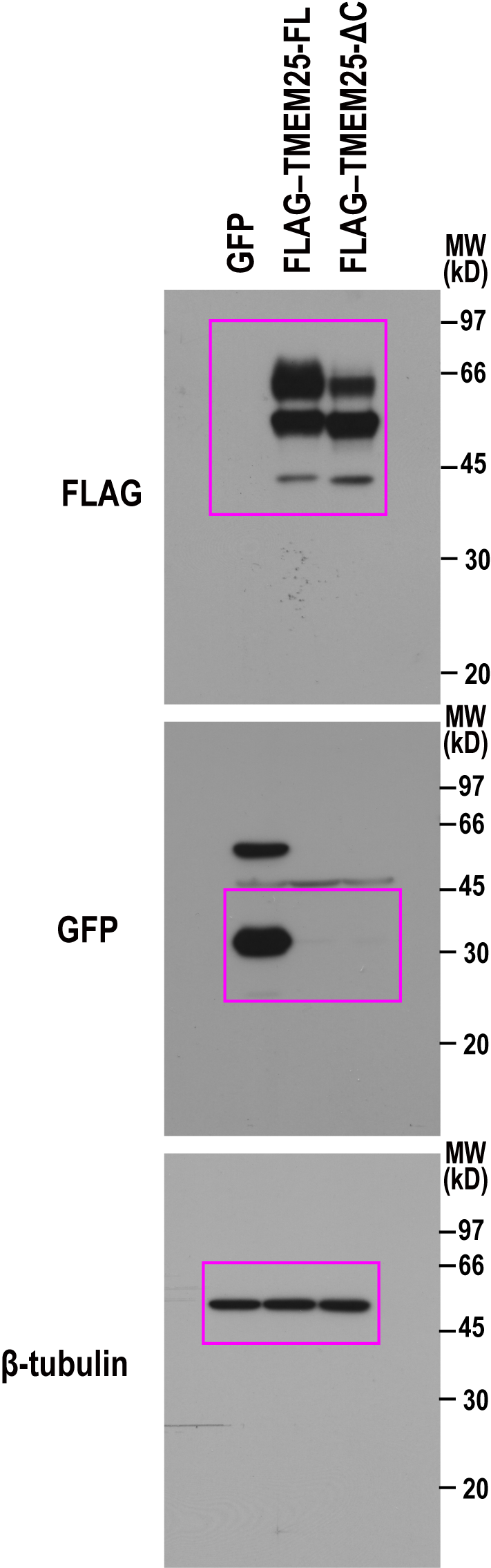

Supplement: Supplementary file 6 — Source Data Fig. 5 [file 44319_2023_18_MOESM6_ESM.zip › Figure_4/4B/4B immunoblot.tif]

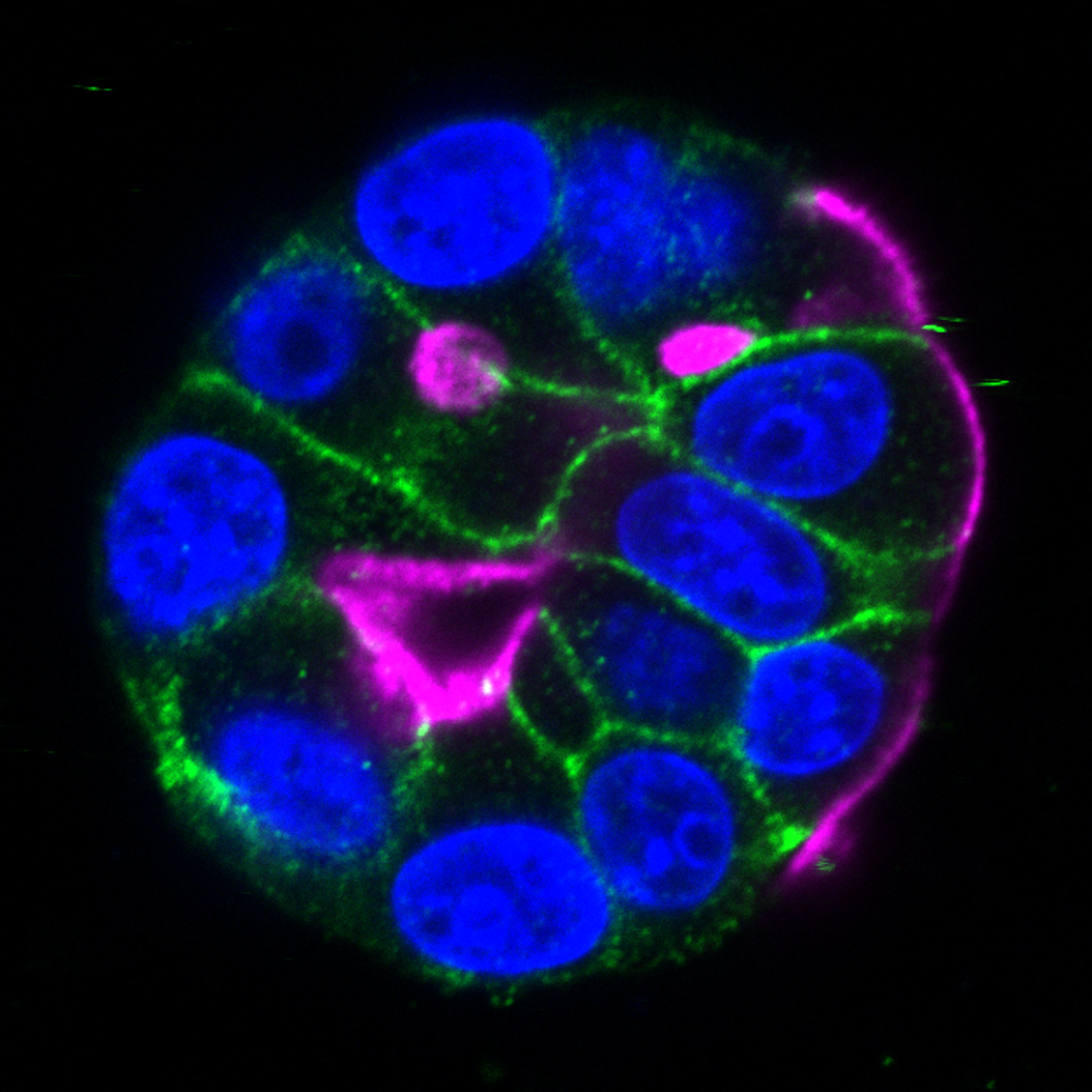

Supplement: Supplementary file 6 — Source Data Fig. 5 [file 44319_2023_18_MOESM6_ESM.zip › Figure_4/4C/Image data/72h_TMEM25_siRNA-2.tif]

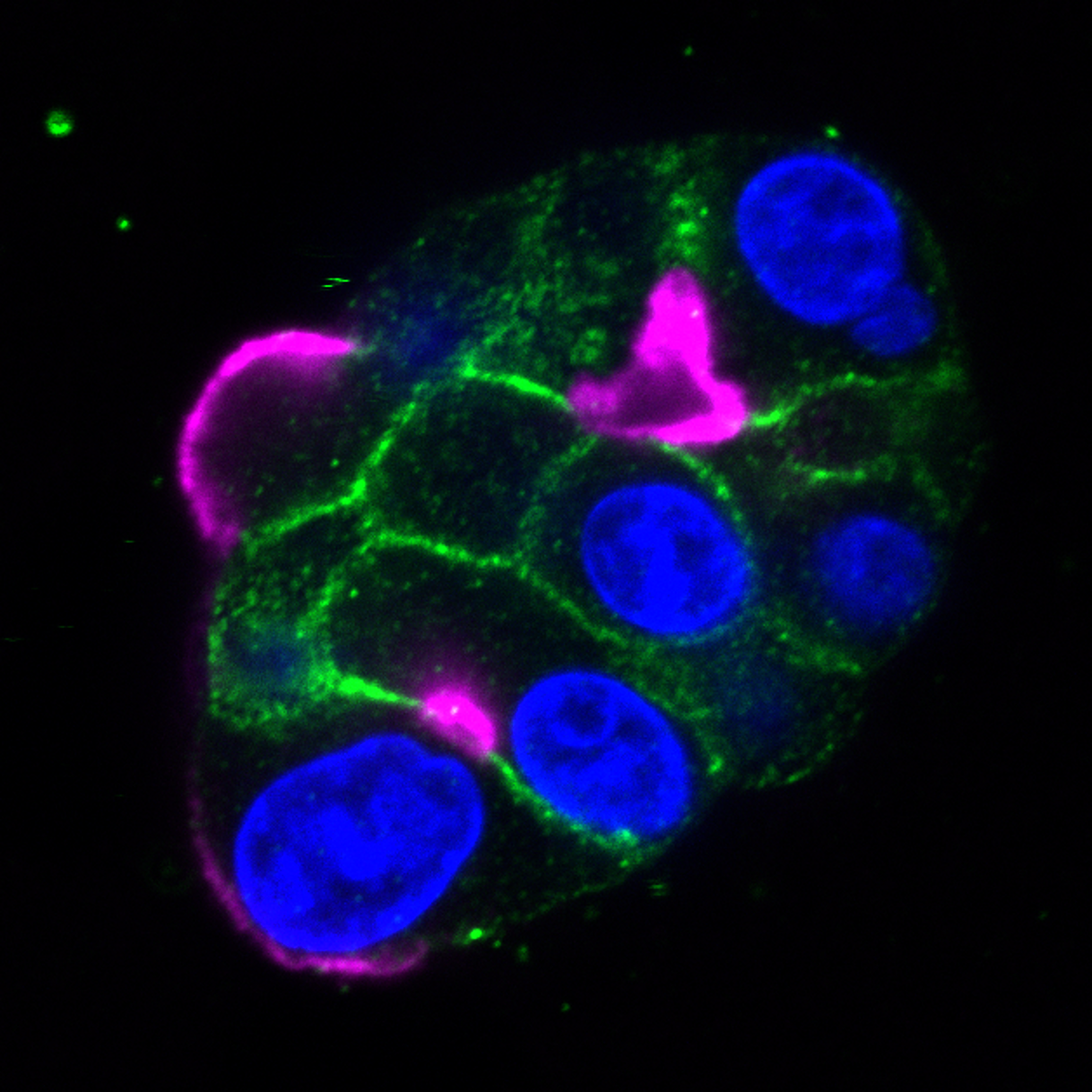

Supplement: Supplementary file 6 — Source Data Fig. 5 [file 44319_2023_18_MOESM6_ESM.zip › Figure_4/4C/Image data/72h_TMEM25_siRNA-1.tif]

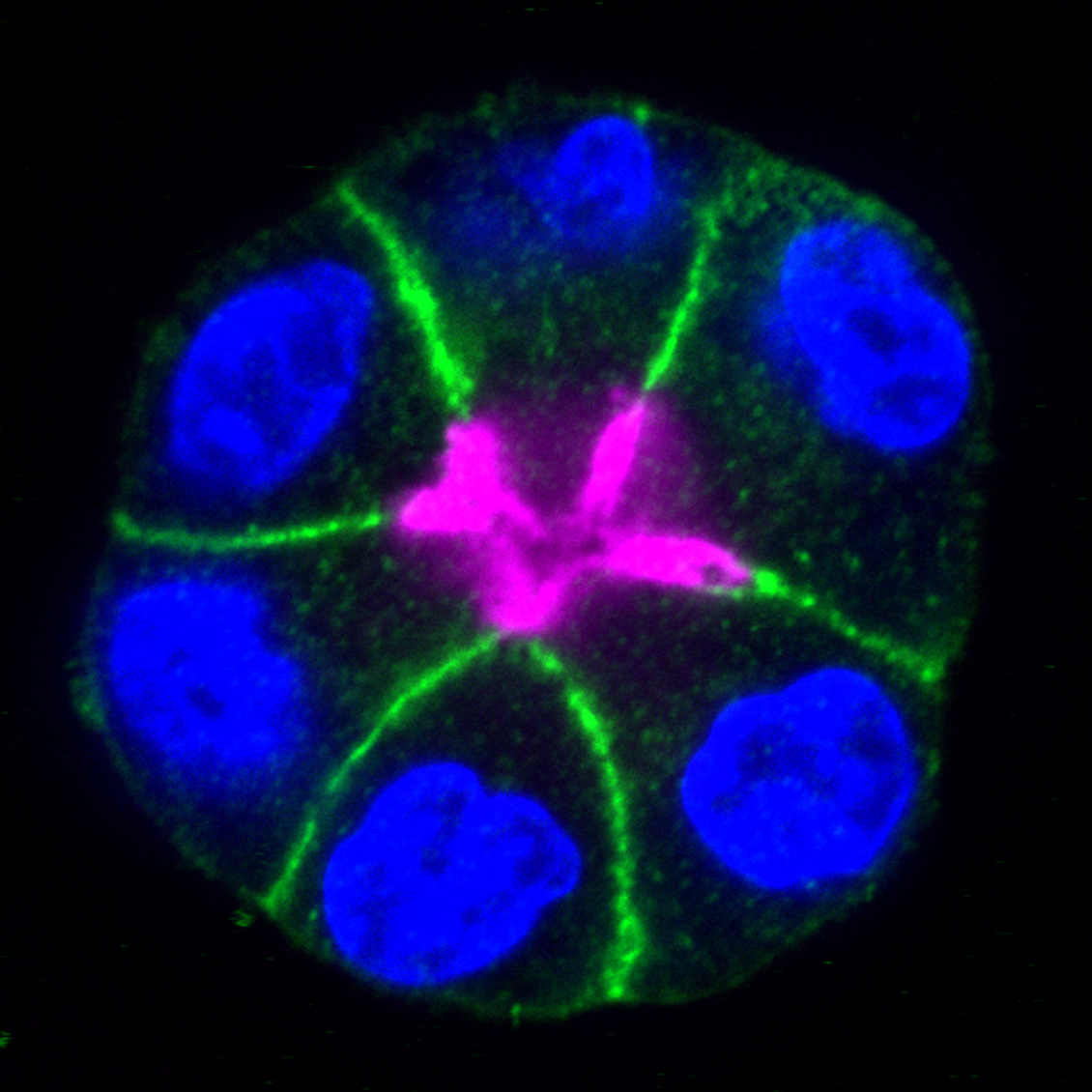

Supplement: Supplementary file 6 — Source Data Fig. 5 [file 44319_2023_18_MOESM6_ESM.zip › Figure_4/4C/Image data/48h_Control_RNA.tif]

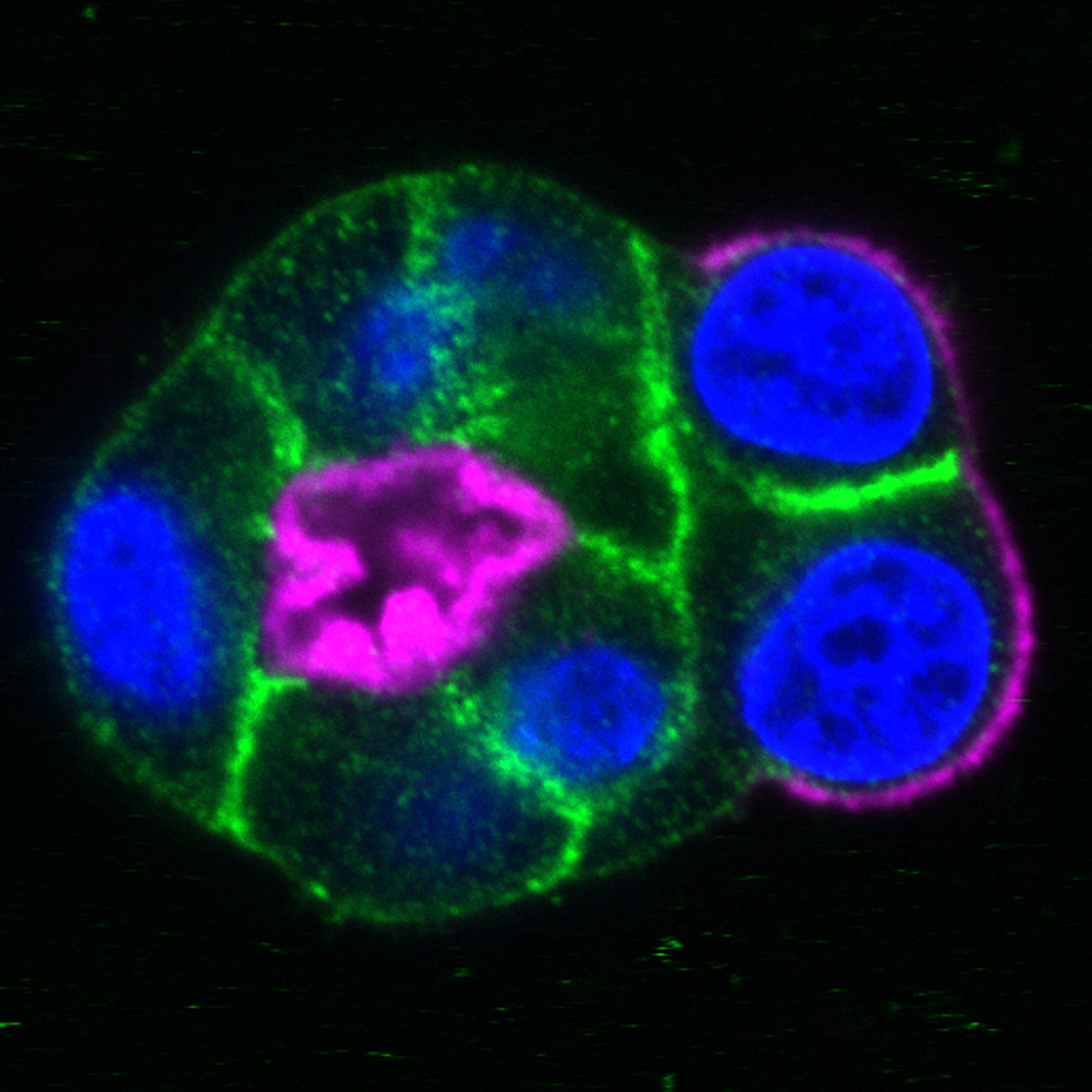

Supplement: Supplementary file 6 — Source Data Fig. 5 [file 44319_2023_18_MOESM6_ESM.zip › Figure_4/4C/Image data/48h_TMEM25_siRNA-1.tif]

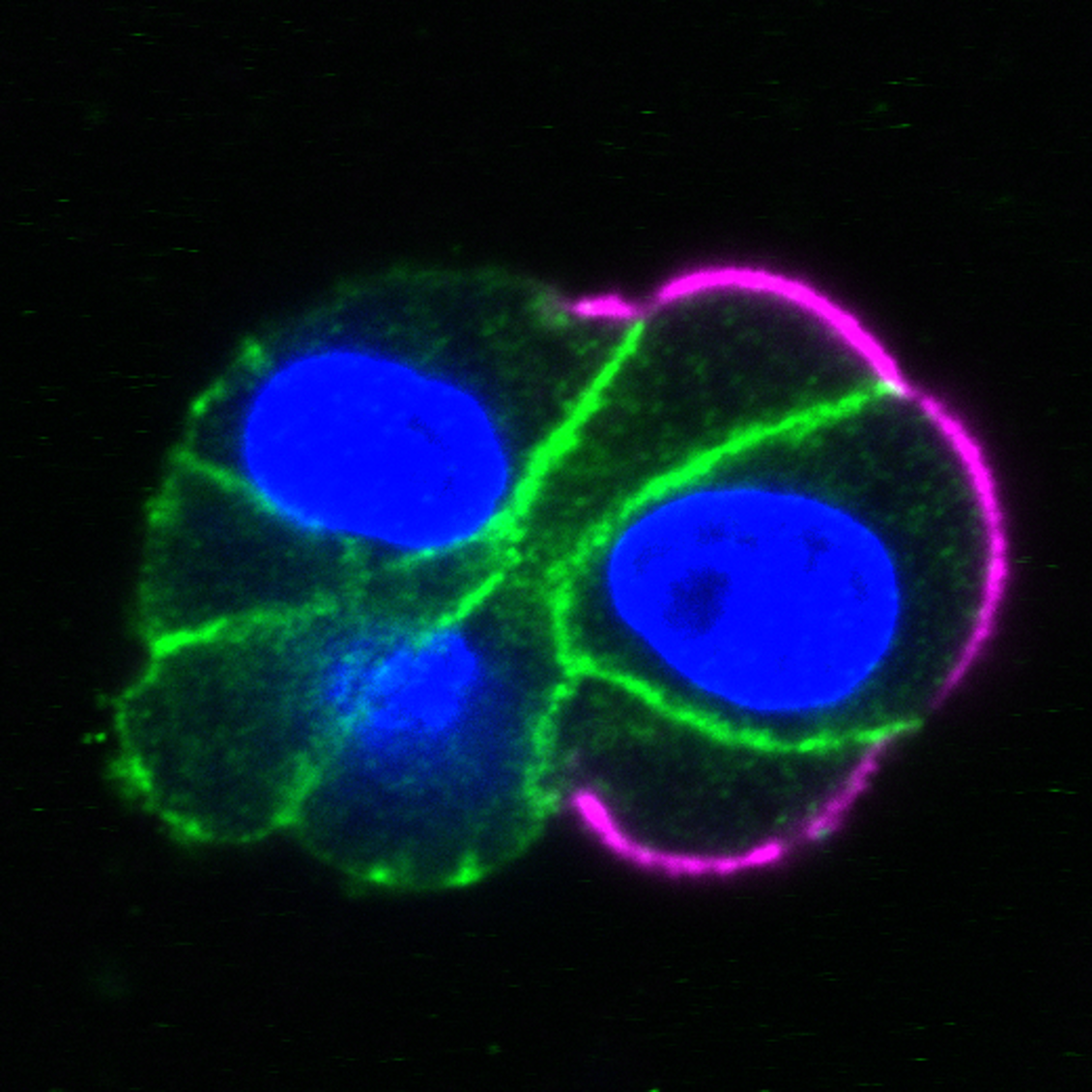

Supplement: Supplementary file 6 — Source Data Fig. 5 [file 44319_2023_18_MOESM6_ESM.zip › Figure_4/4C/Image data/48h_TMEM25_siRNA-2.tif]

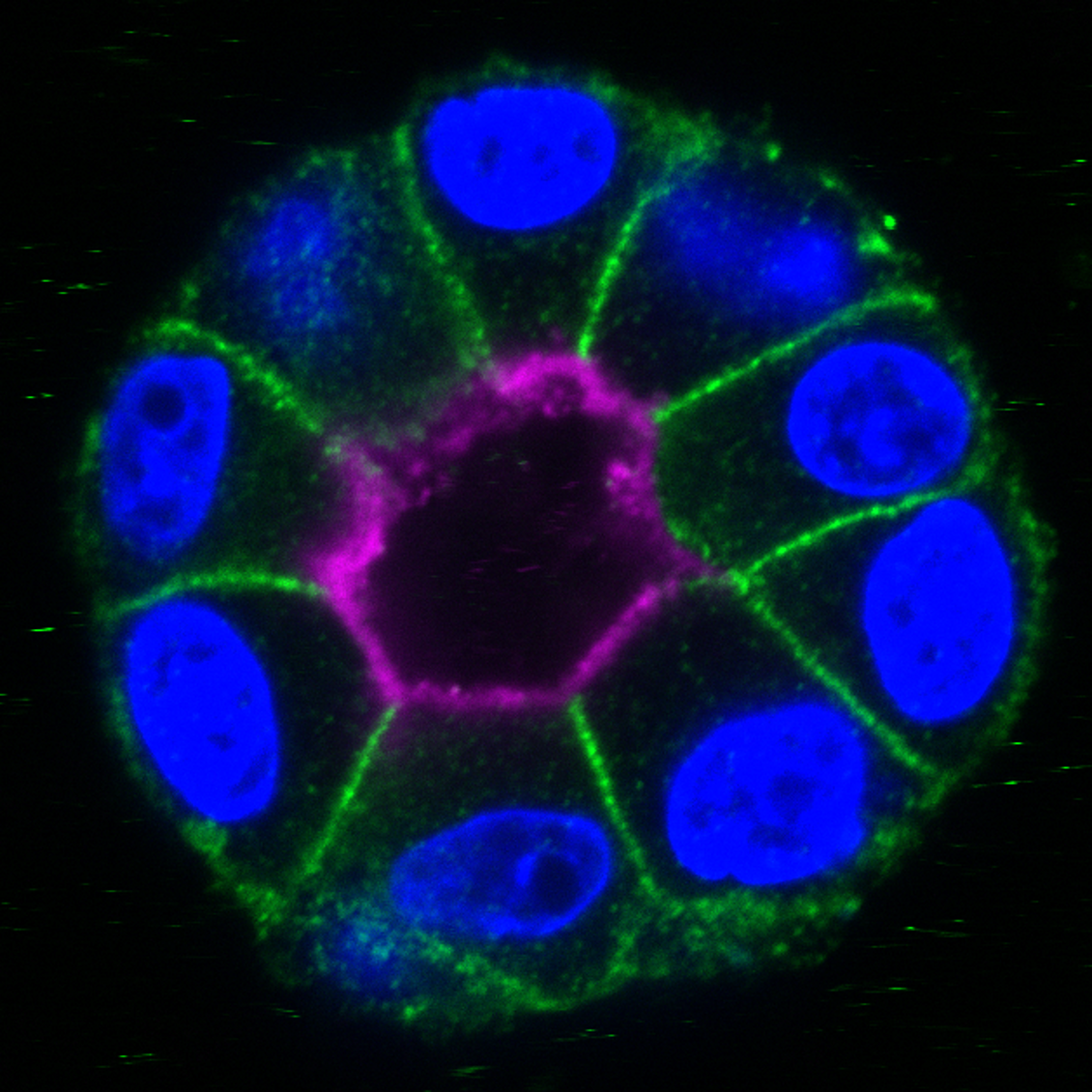

Supplement: Supplementary file 6 — Source Data Fig. 5 [file 44319_2023_18_MOESM6_ESM.zip › Figure_4/4C/Image data/72h_Control_RNA.tif]

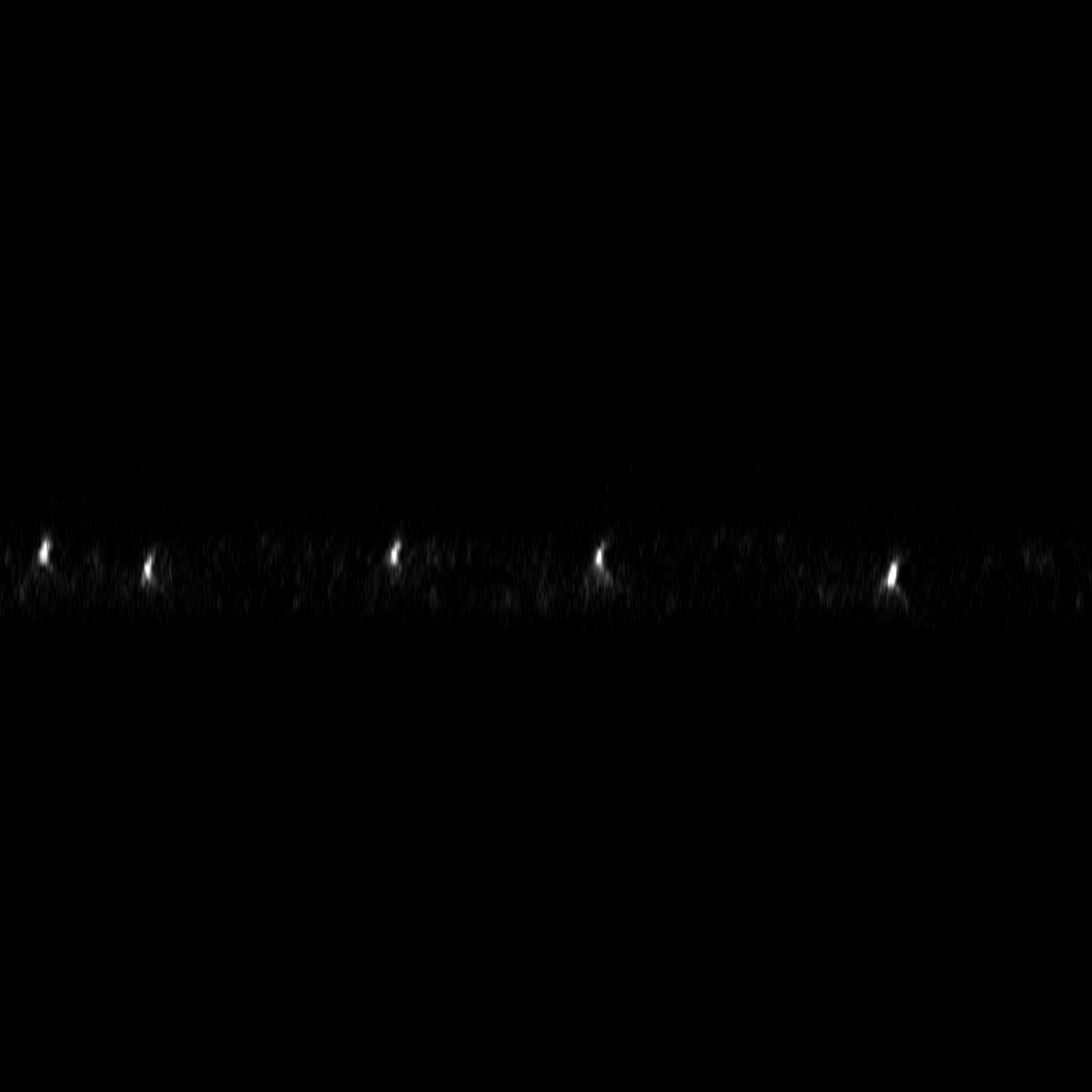

Supplement: Supplementary file 6 — Source Data Fig. 5 [file 44319_2023_18_MOESM6_ESM.zip › Figure_4/4A/4A xz images/12h_middle_ZO1.tif]

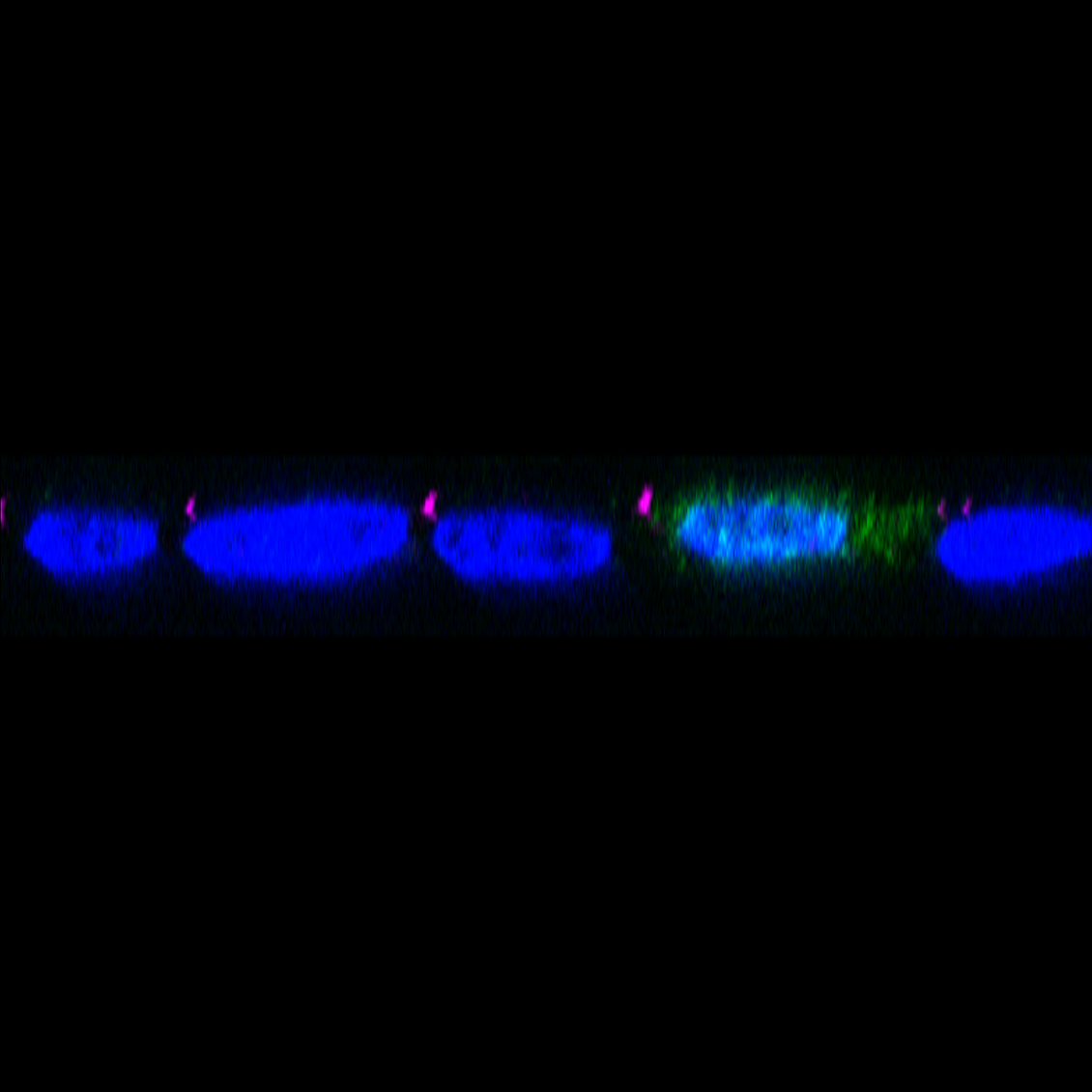

Supplement: Supplementary file 6 — Source Data Fig. 5 [file 44319_2023_18_MOESM6_ESM.zip › Figure_4/4A/4A xz images/6h_left_merge.tif]

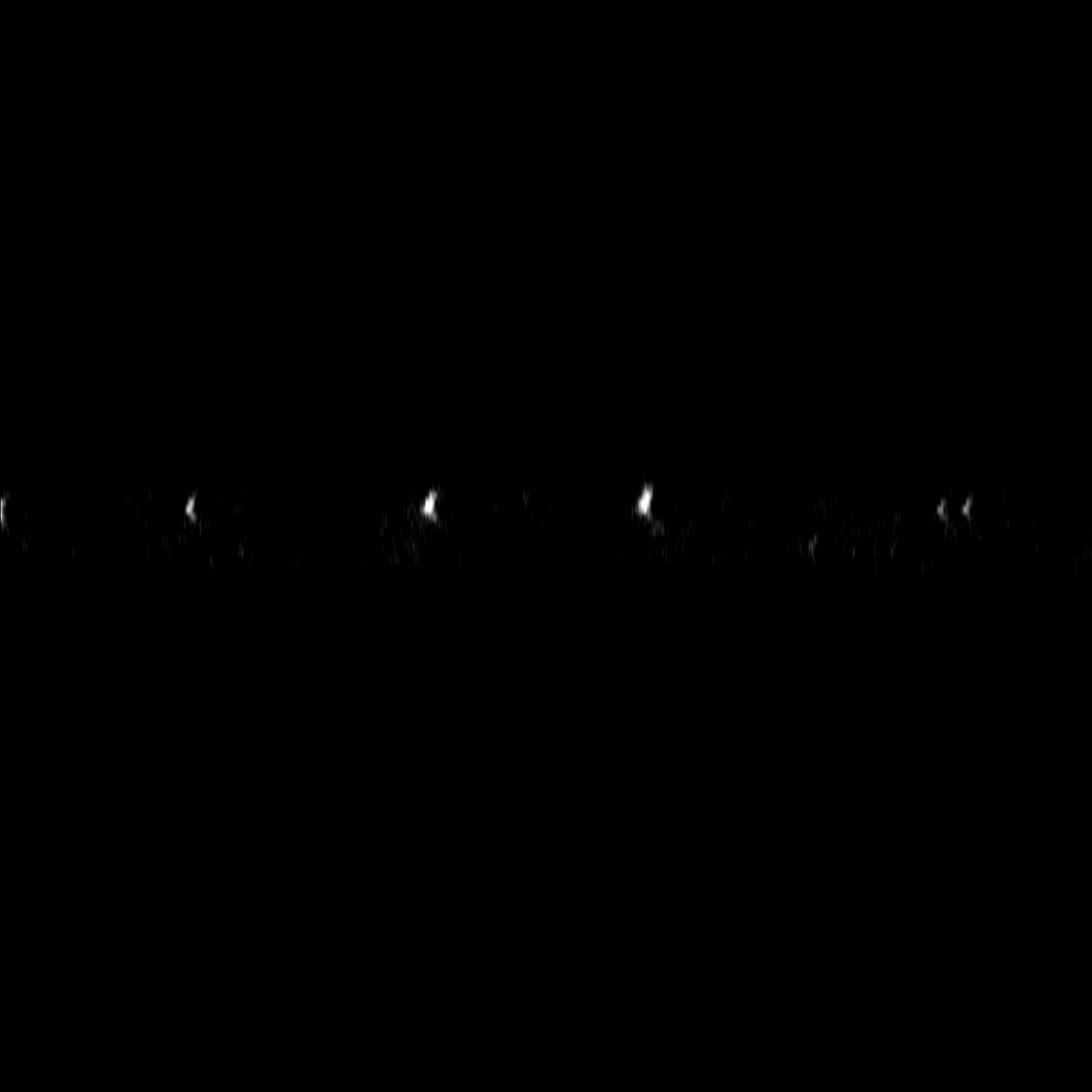

Supplement: Supplementary file 6 — Source Data Fig. 5 [file 44319_2023_18_MOESM6_ESM.zip › Figure_4/4A/4A xz images/6h_left_ZO1.tif]

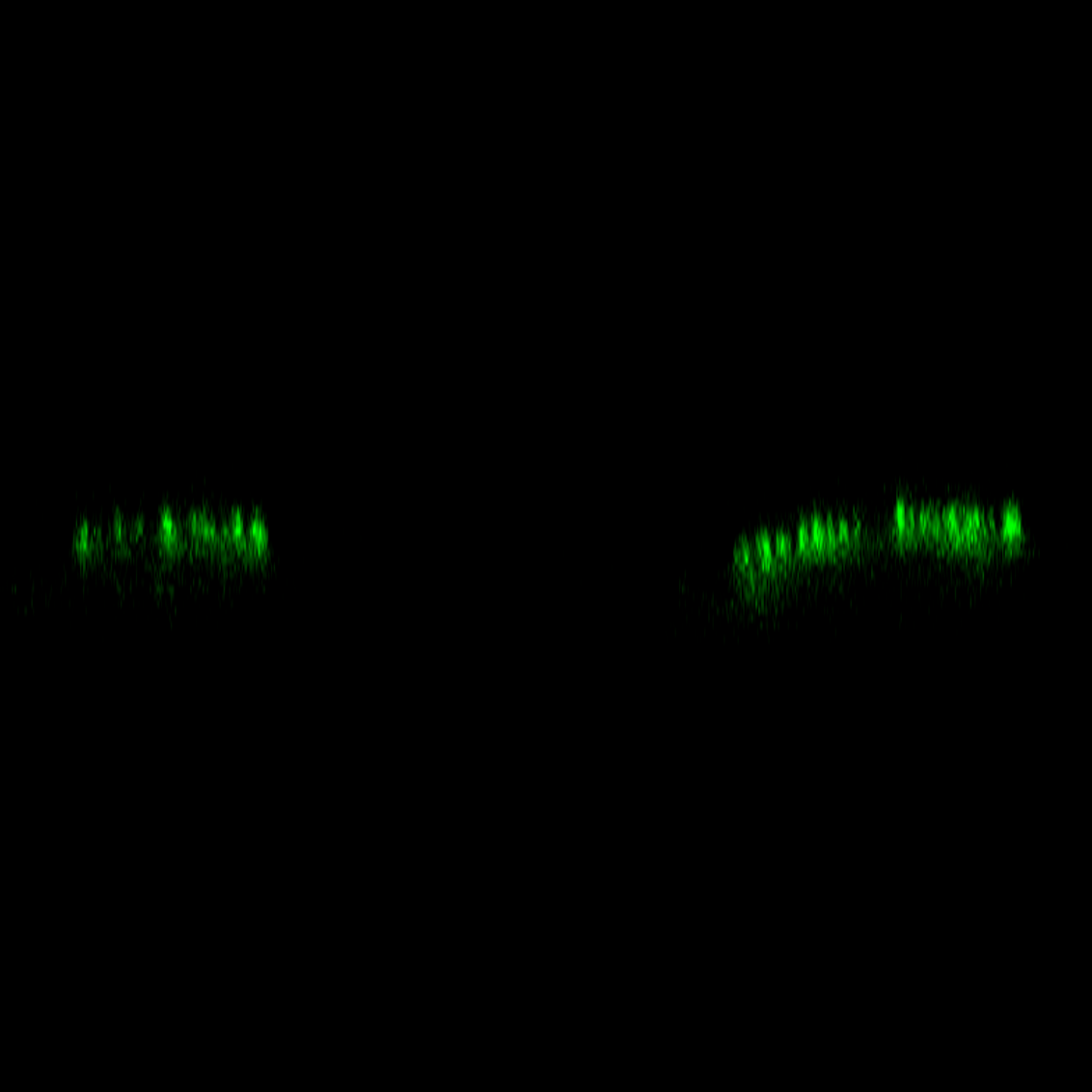

Supplement: Supplementary file 6 — Source Data Fig. 5 [file 44319_2023_18_MOESM6_ESM.zip › Figure_4/4A/4A xz images/12h_right_FLAG.tif]

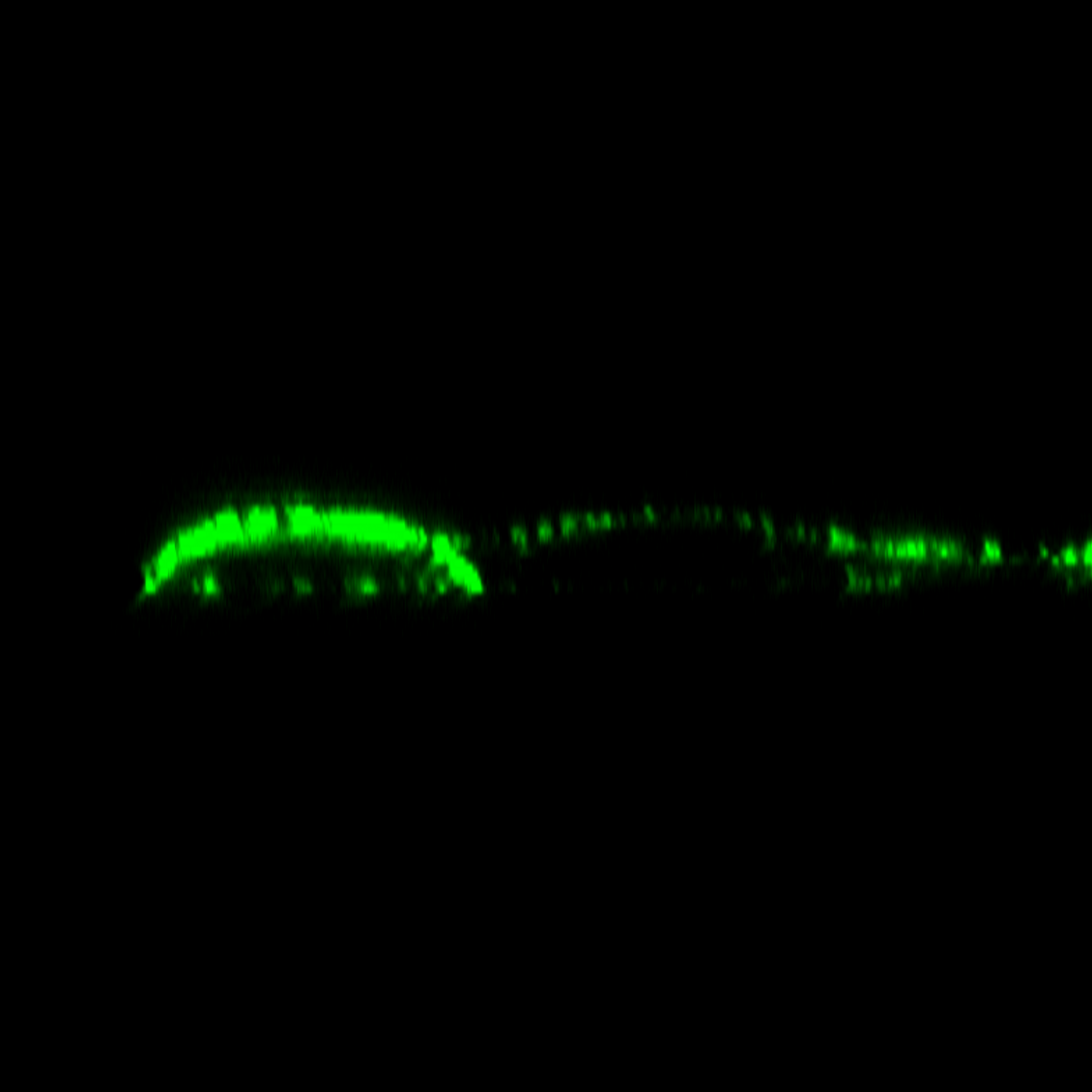

Supplement: Supplementary file 6 — Source Data Fig. 5 [file 44319_2023_18_MOESM6_ESM.zip › Figure_4/4A/4A xz images/6h_middle_FLAG.tif]

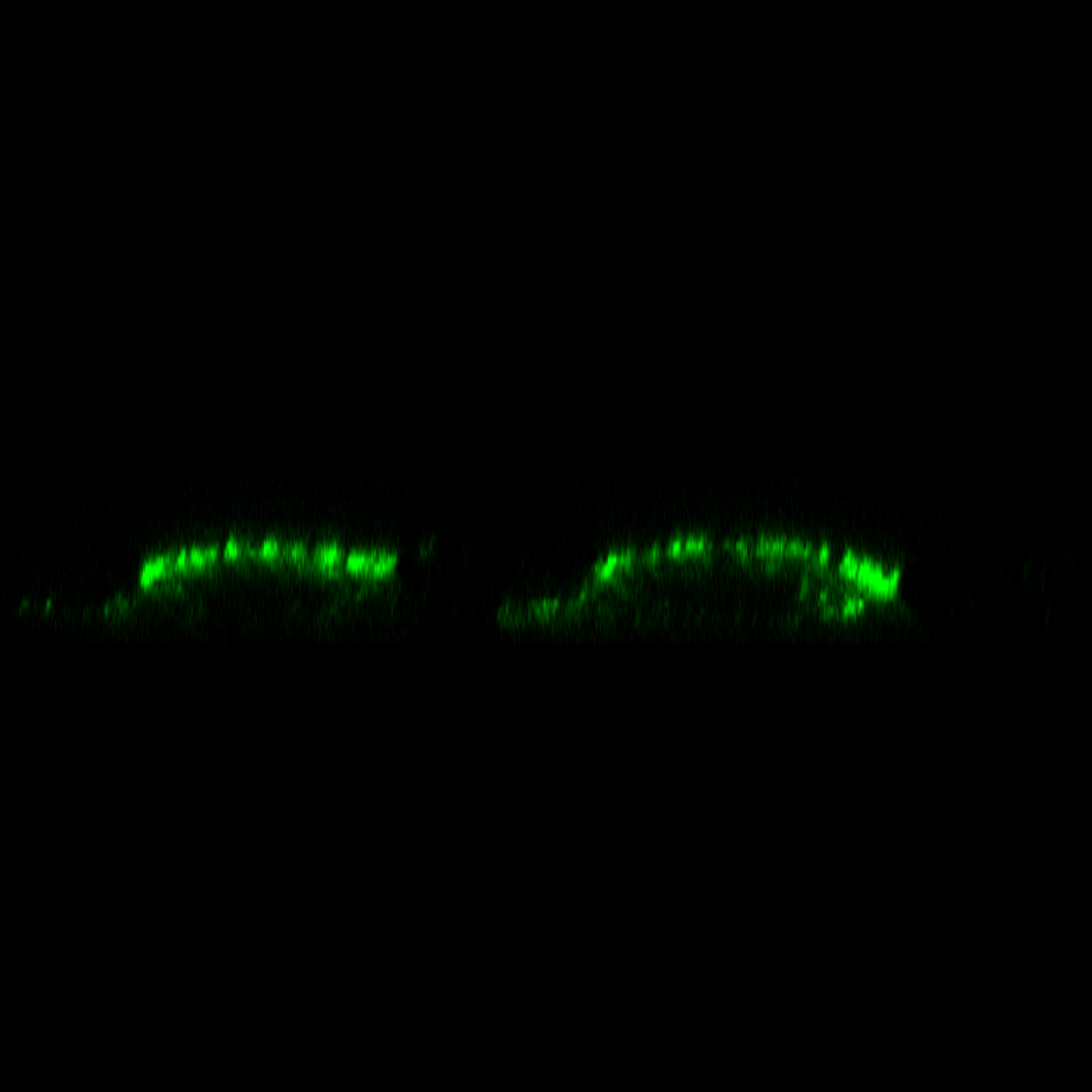

Supplement: Supplementary file 6 — Source Data Fig. 5 [file 44319_2023_18_MOESM6_ESM.zip › Figure_4/4A/4A xz images/12h_middle_FLAG.tif]

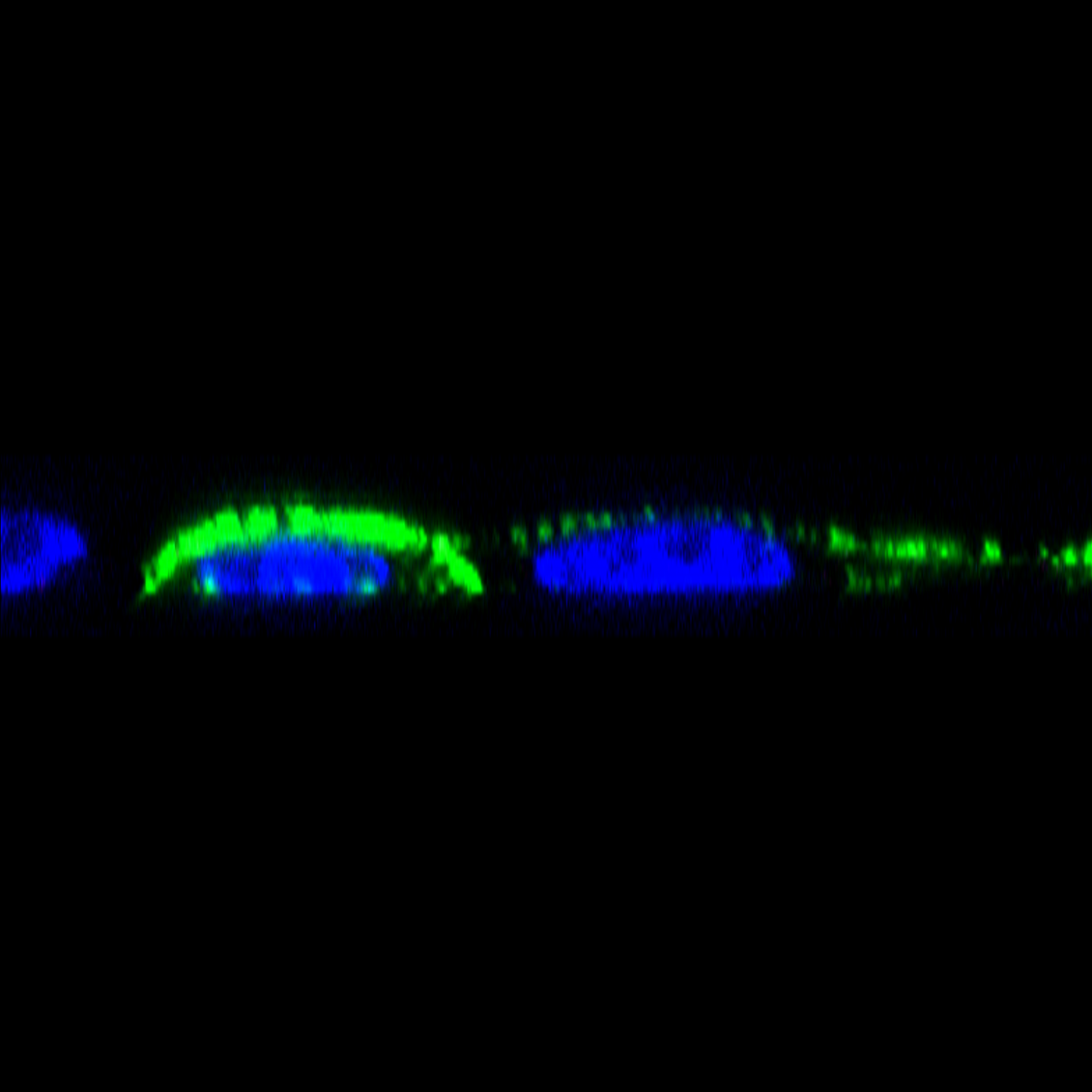

Supplement: Supplementary file 6 — Source Data Fig. 5 [file 44319_2023_18_MOESM6_ESM.zip › Figure_4/4A/4A xz images/6h_middle_merge.tif]

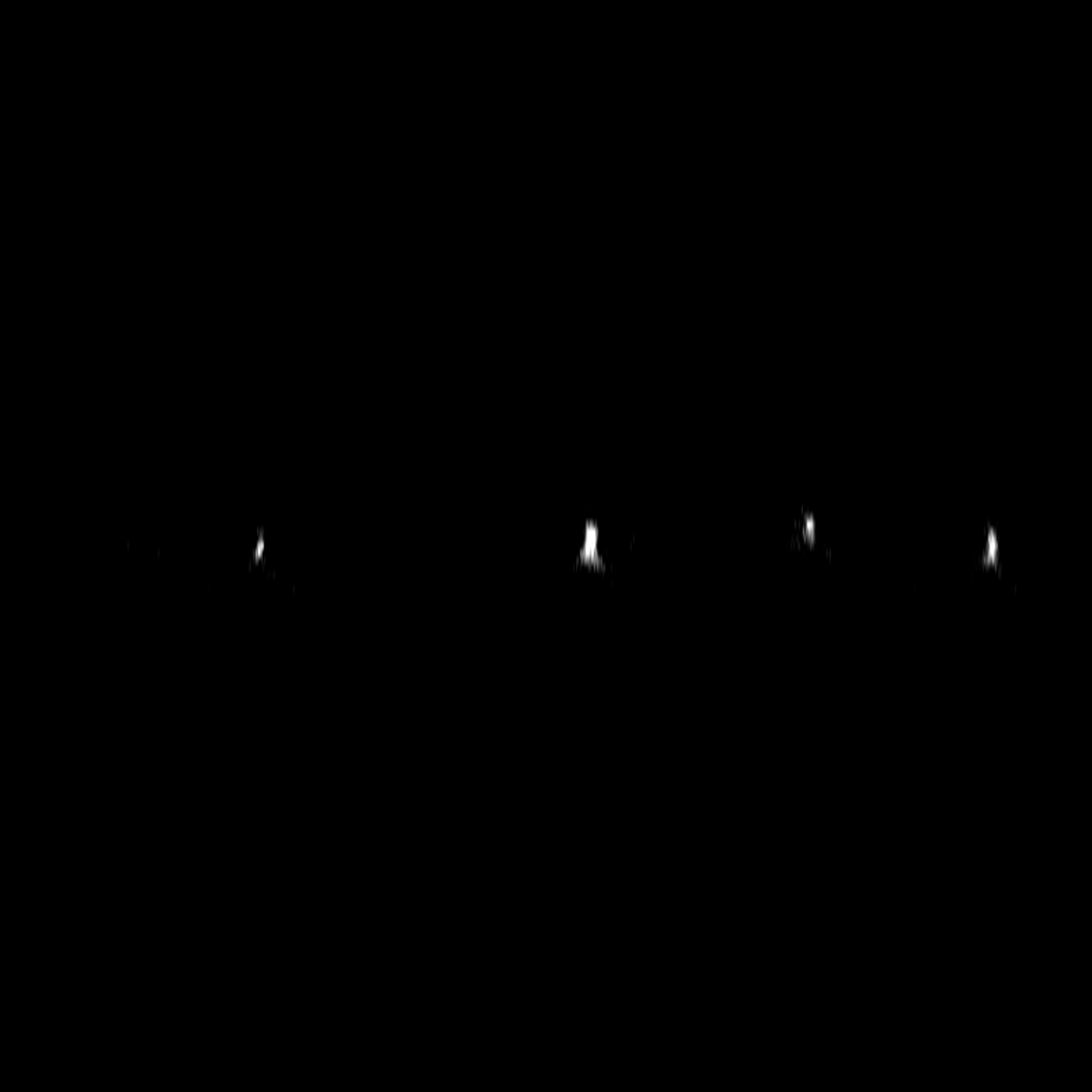

Supplement: Supplementary file 6 — Source Data Fig. 5 [file 44319_2023_18_MOESM6_ESM.zip › Figure_4/4A/4A xz images/6h_right_ZO1.tif]

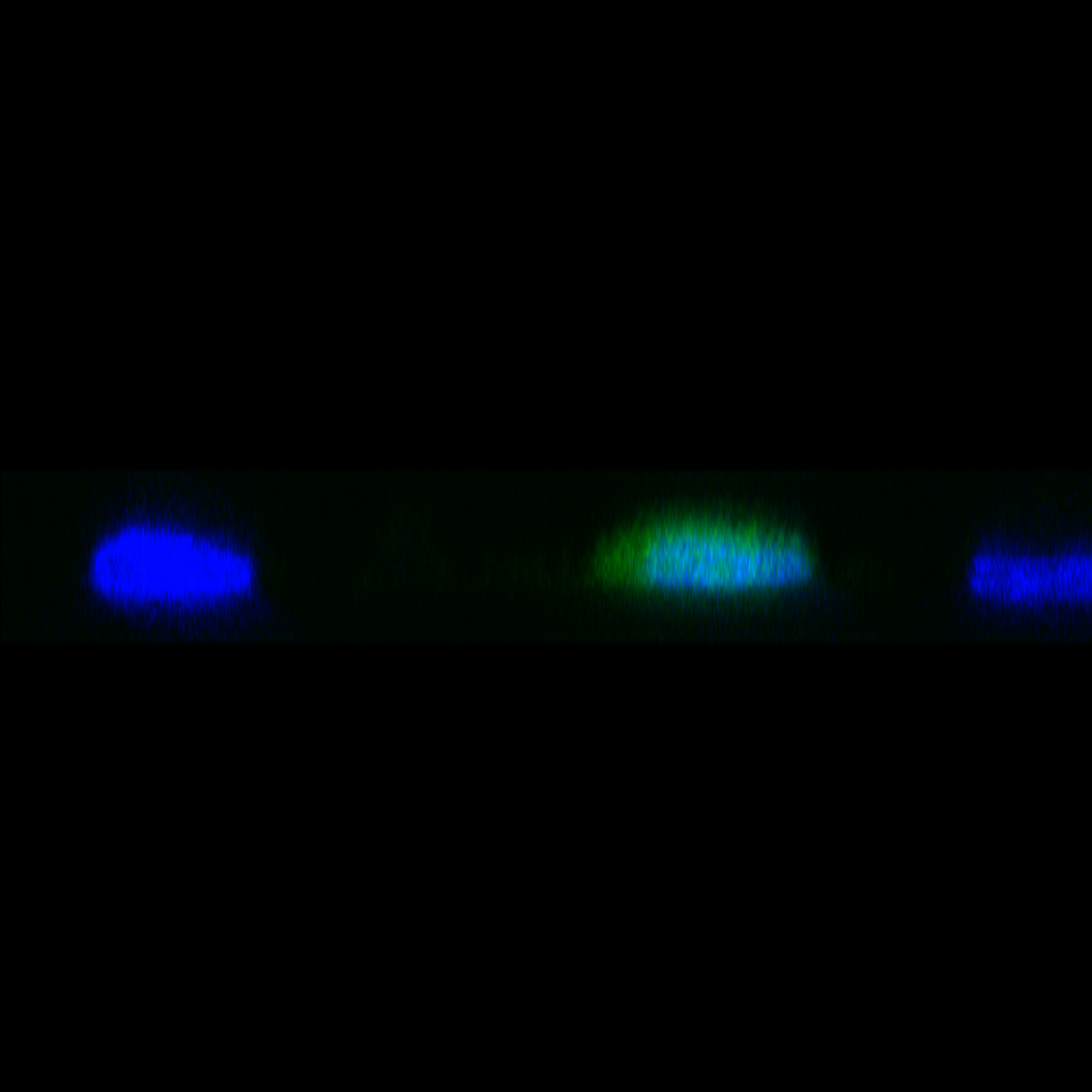

Supplement: Supplementary file 6 — Source Data Fig. 5 [file 44319_2023_18_MOESM6_ESM.zip › Figure_4/4A/4A xz images/0h_left_merge.tif]

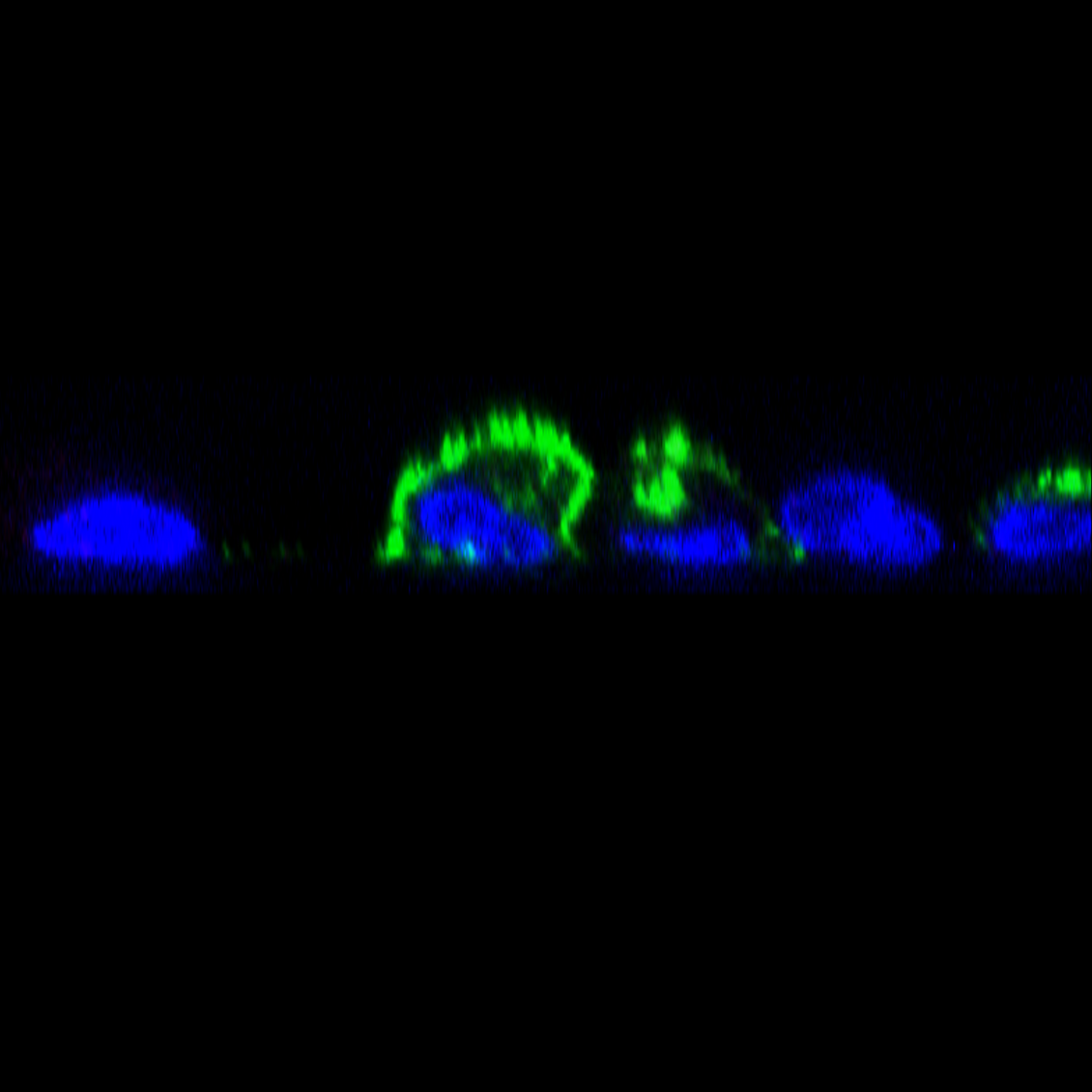

Supplement: Supplementary file 6 — Source Data Fig. 5 [file 44319_2023_18_MOESM6_ESM.zip › Figure_4/4A/4A xz images/0h_middle_merge.tif]

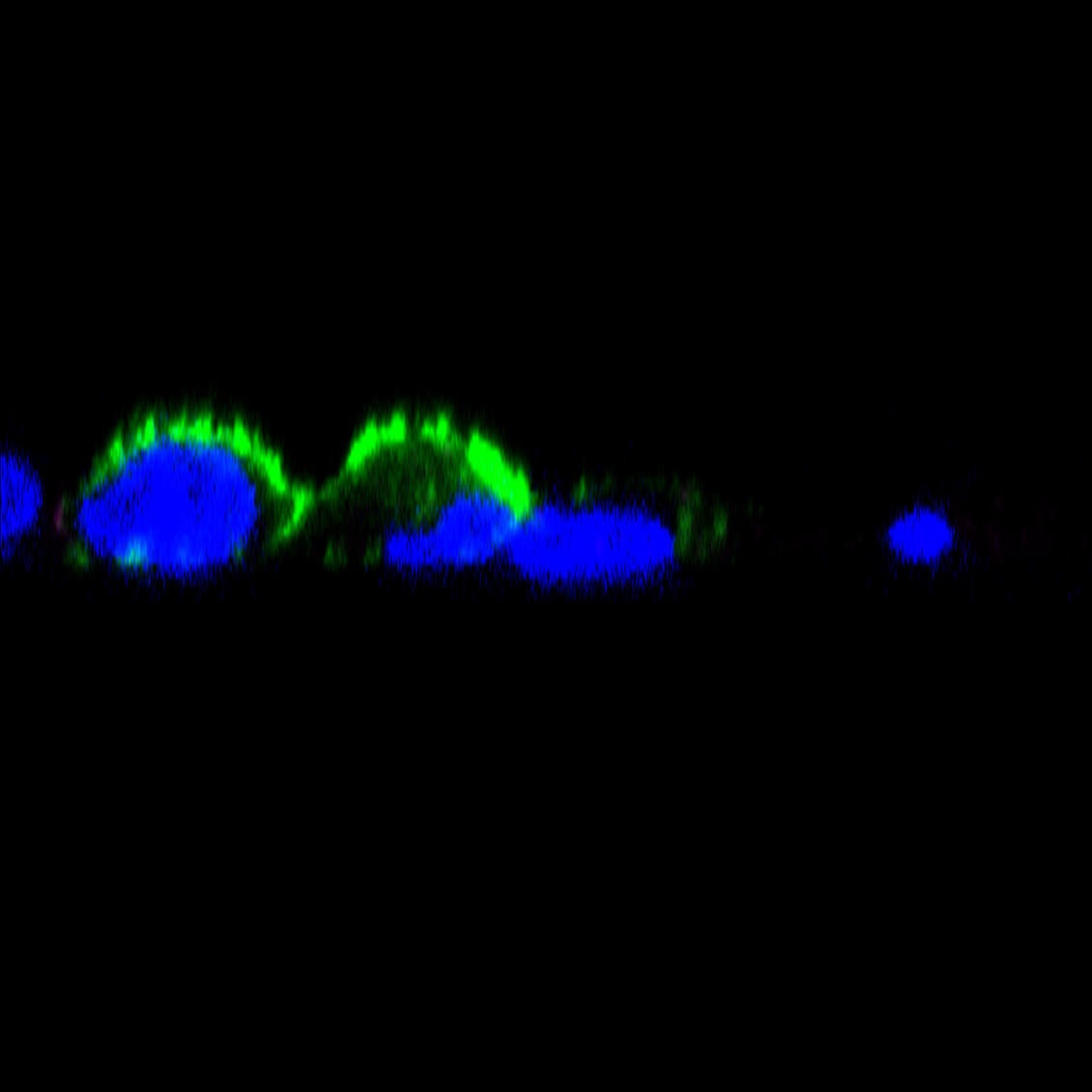

Supplement: Supplementary file 6 — Source Data Fig. 5 [file 44319_2023_18_MOESM6_ESM.zip › Figure_4/4A/4A xz images/0h_right_merge.tif]

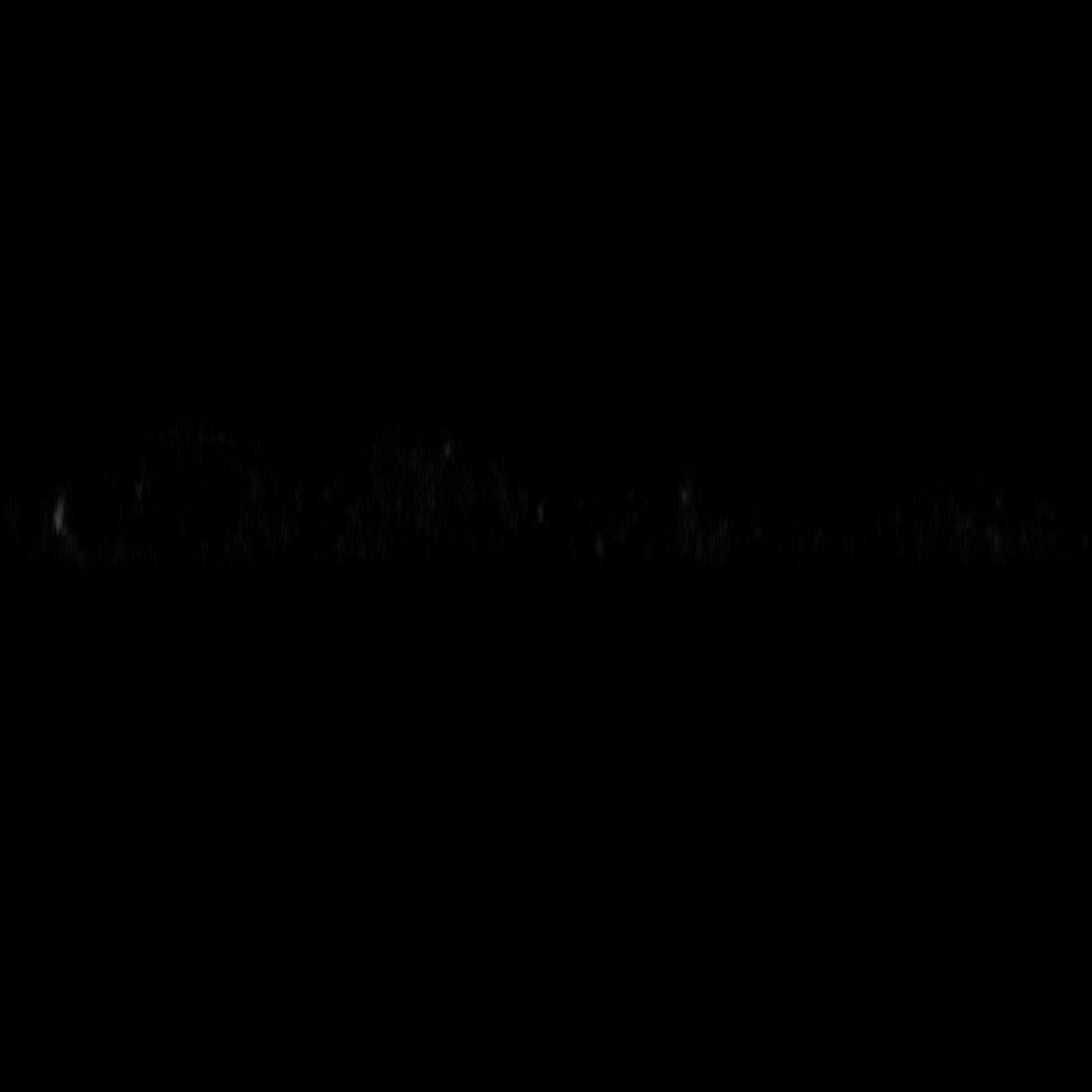

Supplement: Supplementary file 6 — Source Data Fig. 5 [file 44319_2023_18_MOESM6_ESM.zip › Figure_4/4A/4A xz images/0h_right_ZO1.tif]

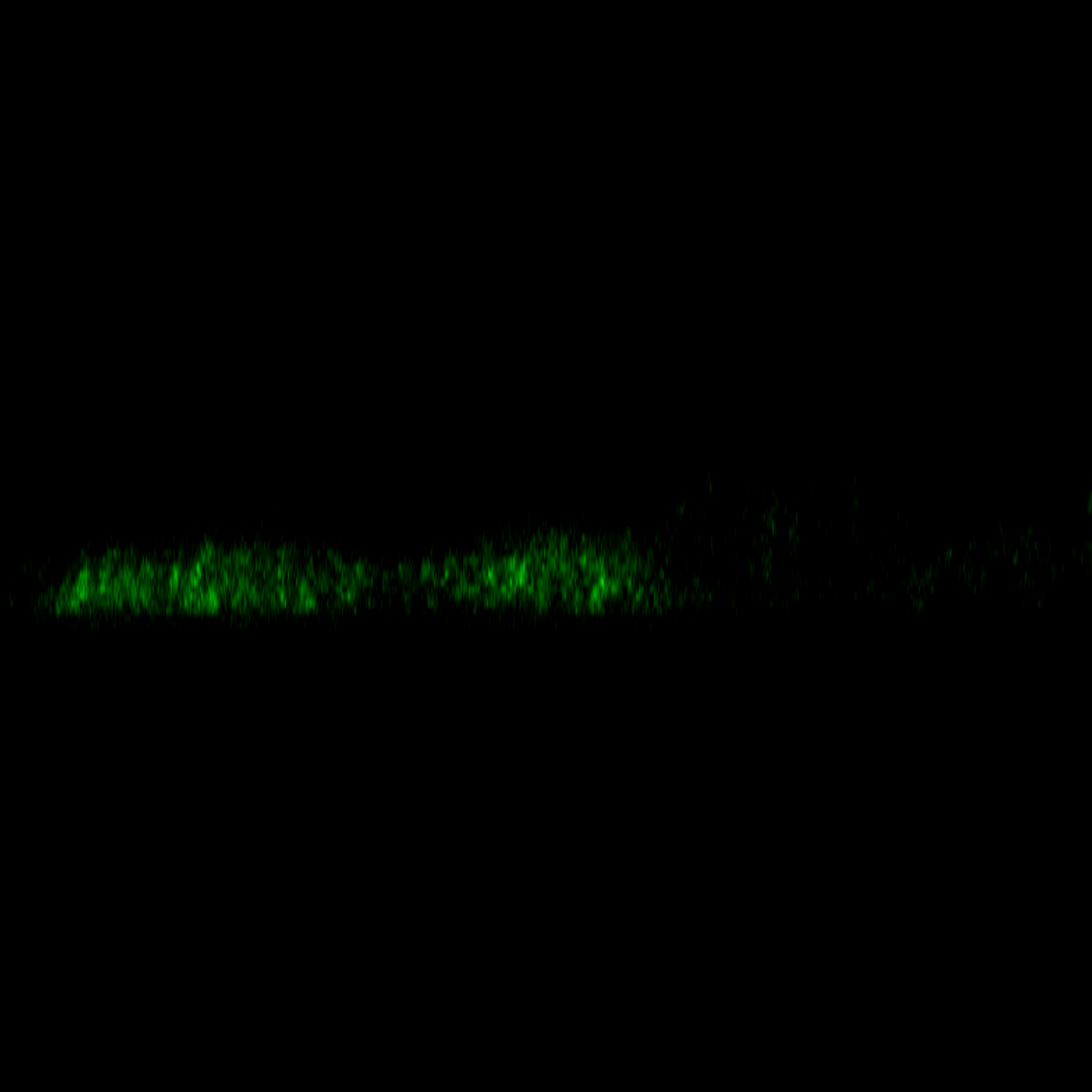

Supplement: Supplementary file 6 — Source Data Fig. 5 [file 44319_2023_18_MOESM6_ESM.zip › Figure_4/4A/4A xz images/12h_left_GFP.tif]

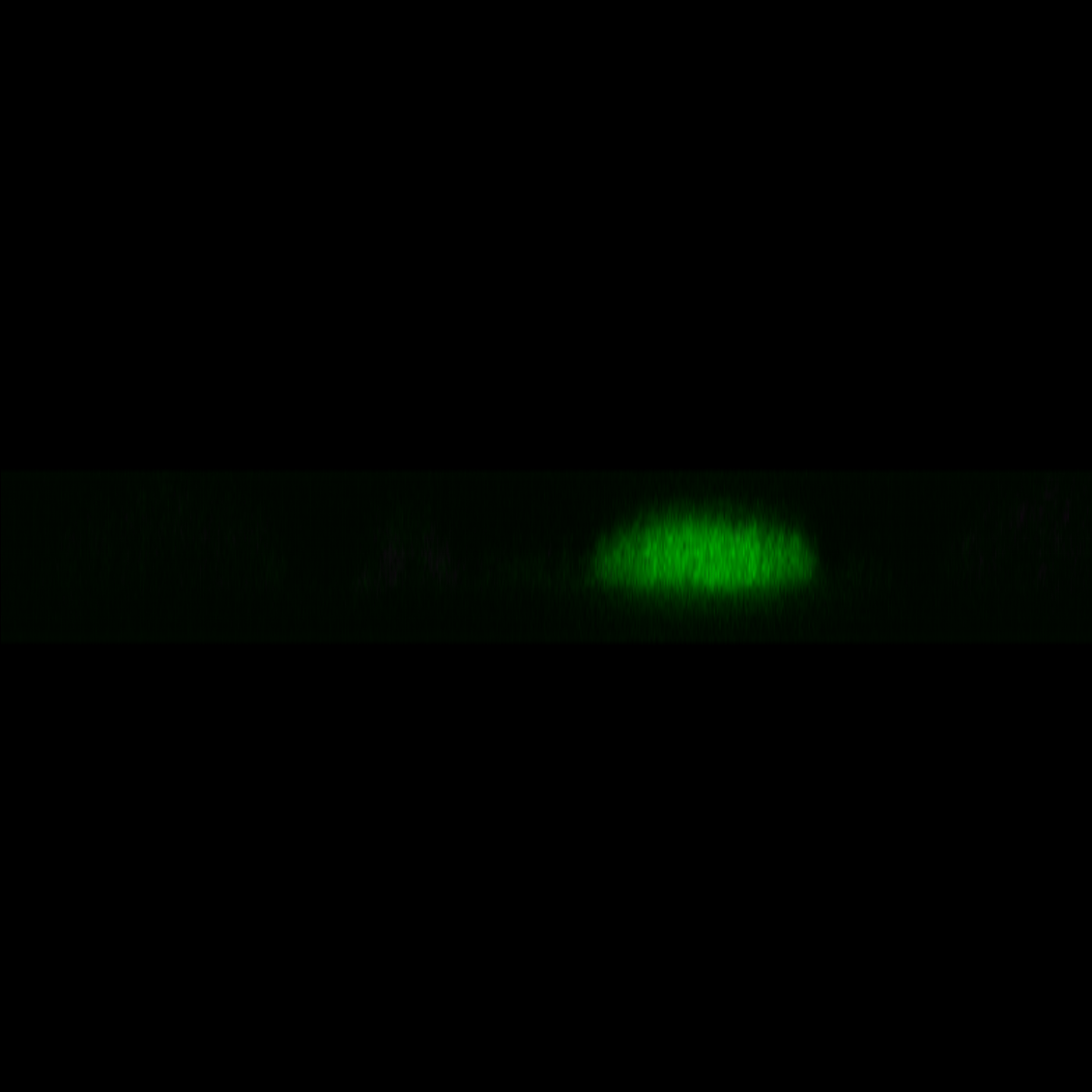

Supplement: Supplementary file 6 — Source Data Fig. 5 [file 44319_2023_18_MOESM6_ESM.zip › Figure_4/4A/4A xz images/0h_left_GFP.tif]

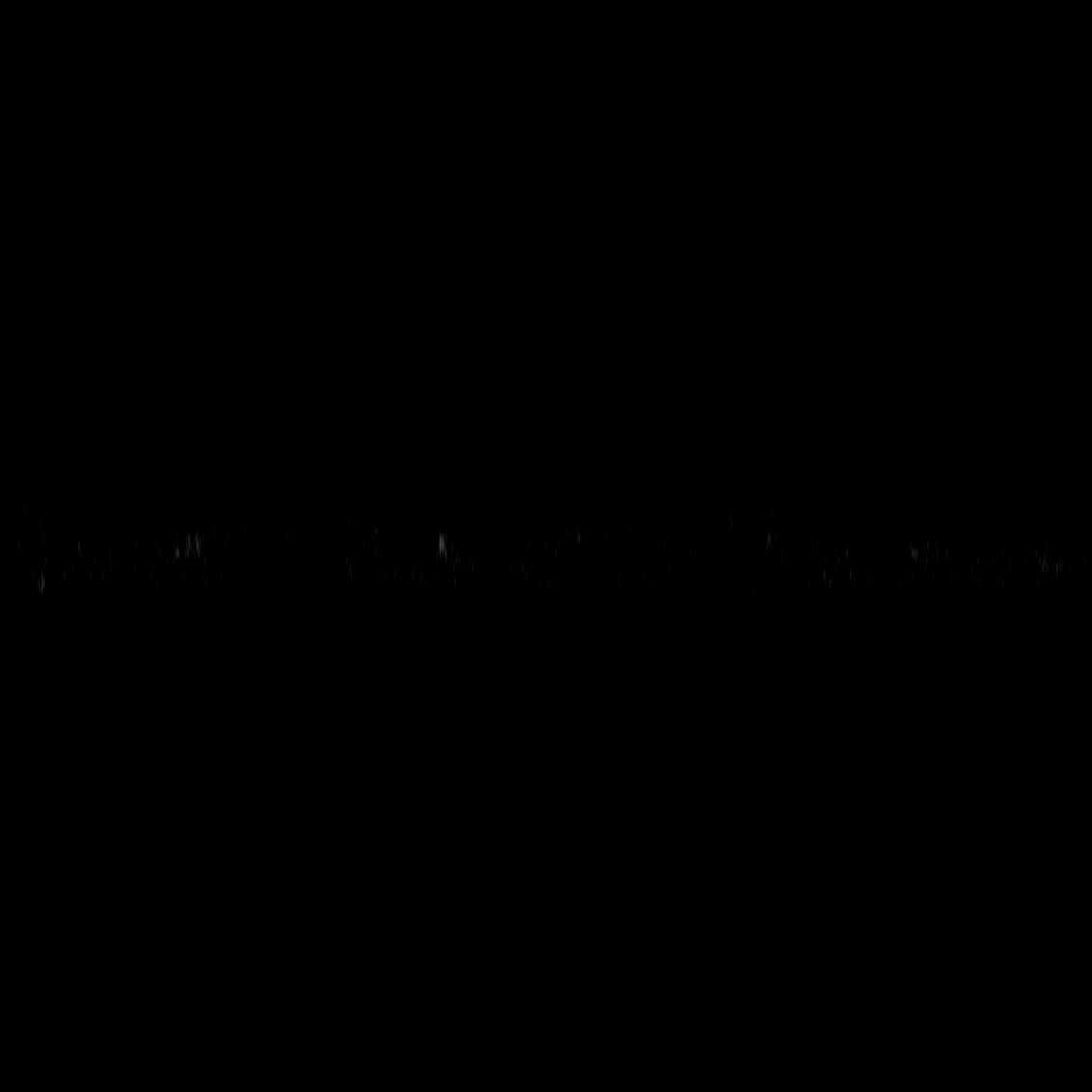

Supplement: Supplementary file 6 — Source Data Fig. 5 [file 44319_2023_18_MOESM6_ESM.zip › Figure_4/4A/4A xz images/6h_middle_ZO1.tif]

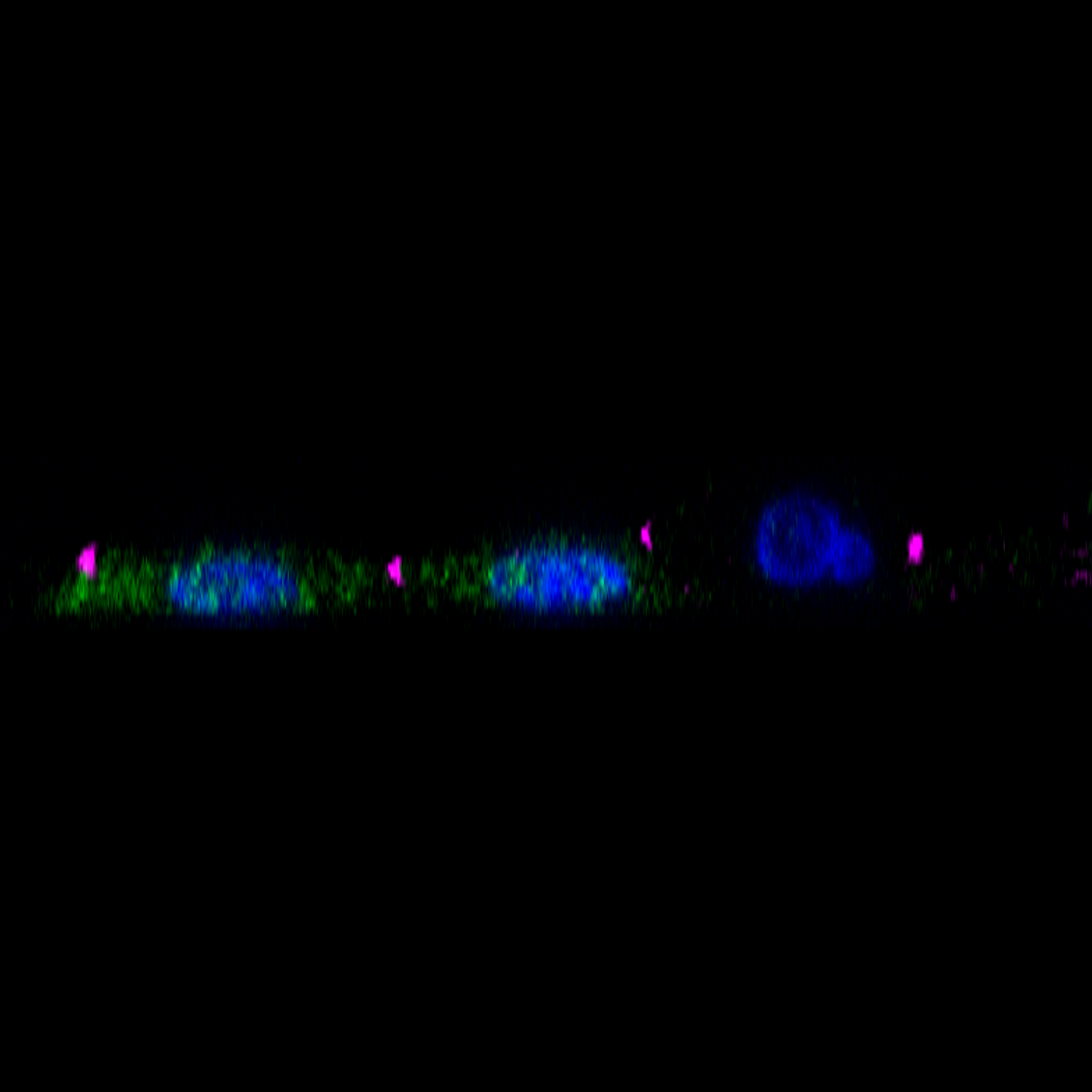

Supplement: Supplementary file 6 — Source Data Fig. 5 [file 44319_2023_18_MOESM6_ESM.zip › Figure_4/4A/4A xz images/12h_left_merge.tif]

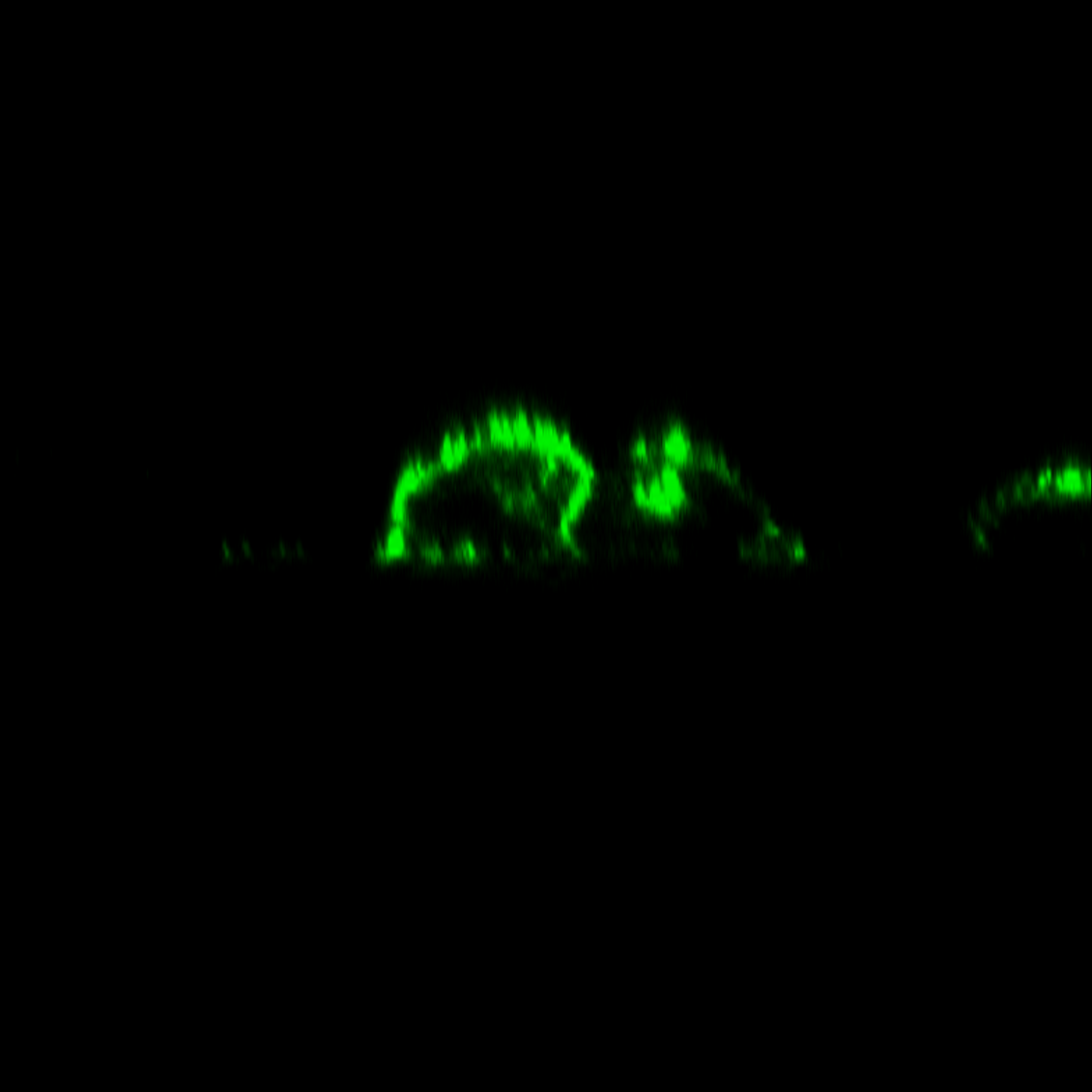

Supplement: Supplementary file 6 — Source Data Fig. 5 [file 44319_2023_18_MOESM6_ESM.zip › Figure_4/4A/4A xz images/0h_middle_FLAG.tif]

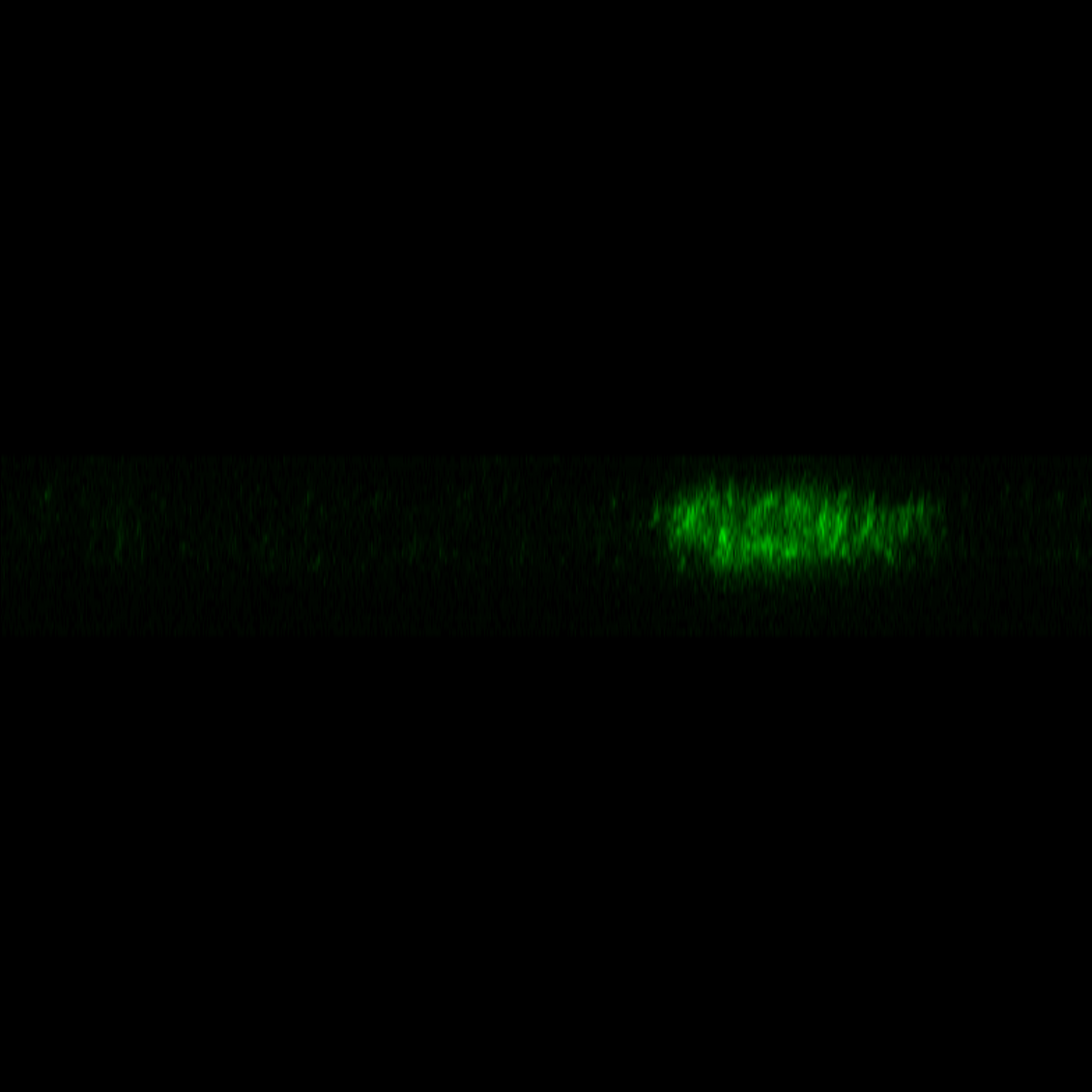

Supplement: Supplementary file 6 — Source Data Fig. 5 [file 44319_2023_18_MOESM6_ESM.zip › Figure_4/4A/4A xz images/6h_left_GFP.tif]

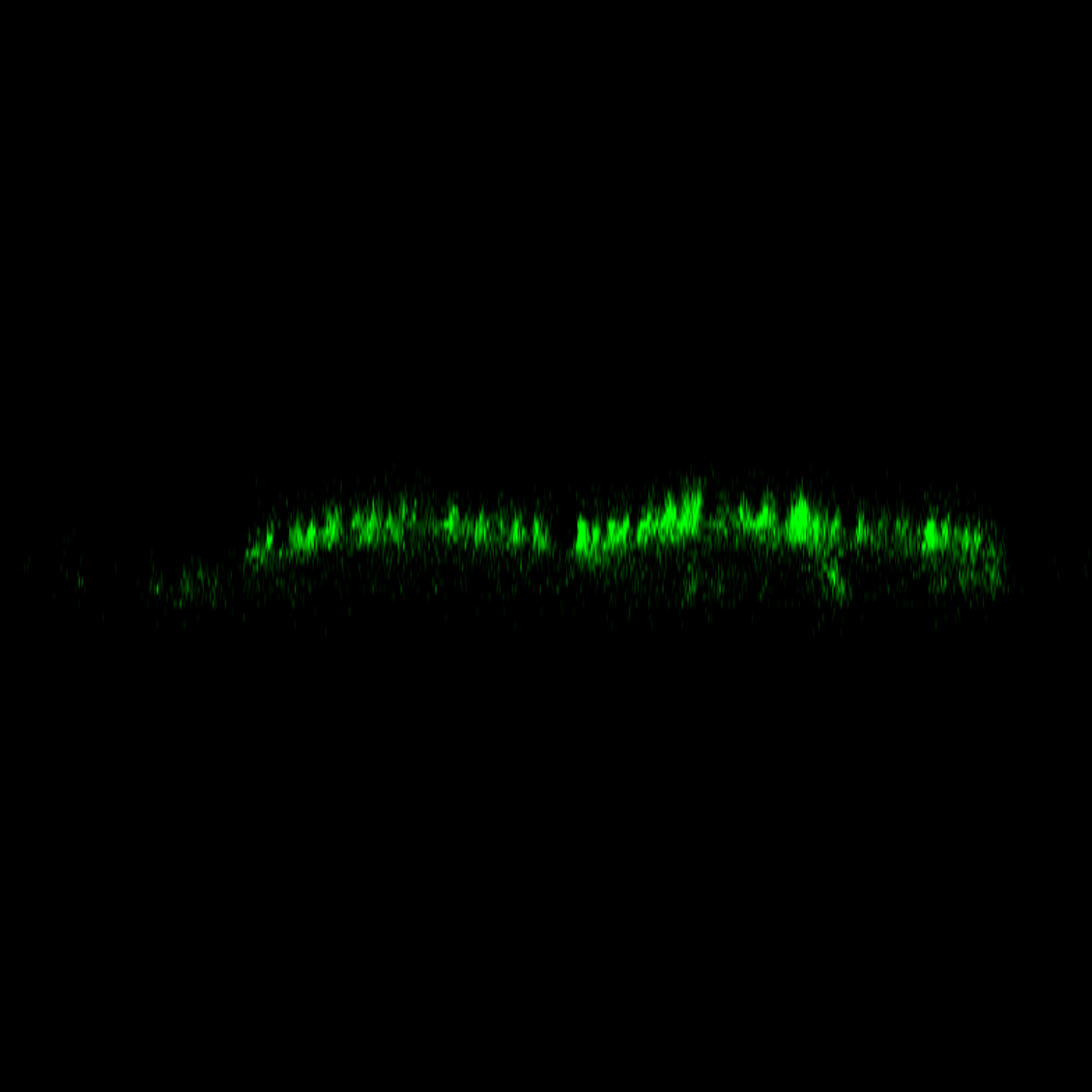

Supplement: Supplementary file 6 — Source Data Fig. 5 [file 44319_2023_18_MOESM6_ESM.zip › Figure_4/4A/4A xz images/6h_right_FLAG.tif]

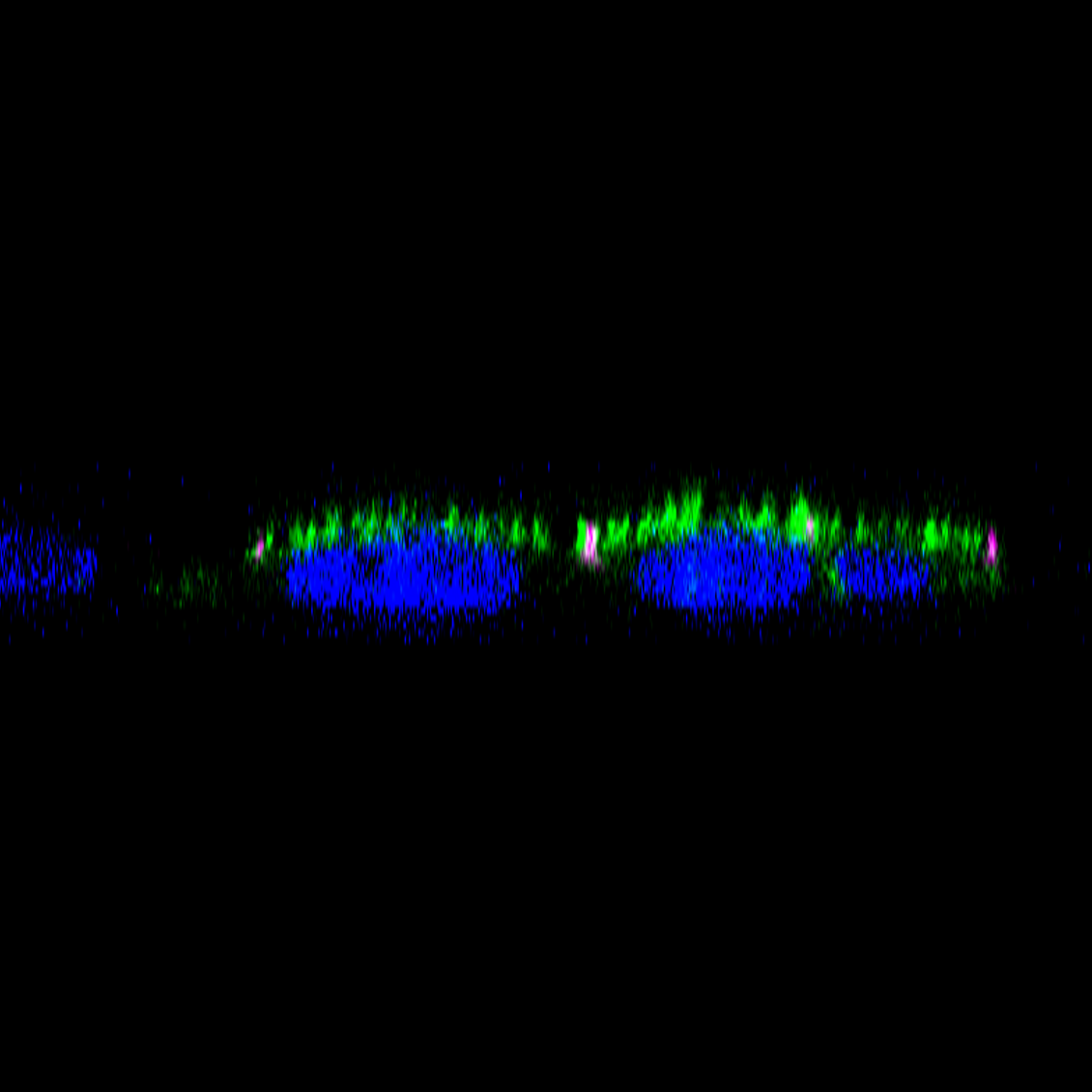

Supplement: Supplementary file 6 — Source Data Fig. 5 [file 44319_2023_18_MOESM6_ESM.zip › Figure_4/4A/4A xz images/6h_right_merge.tif]

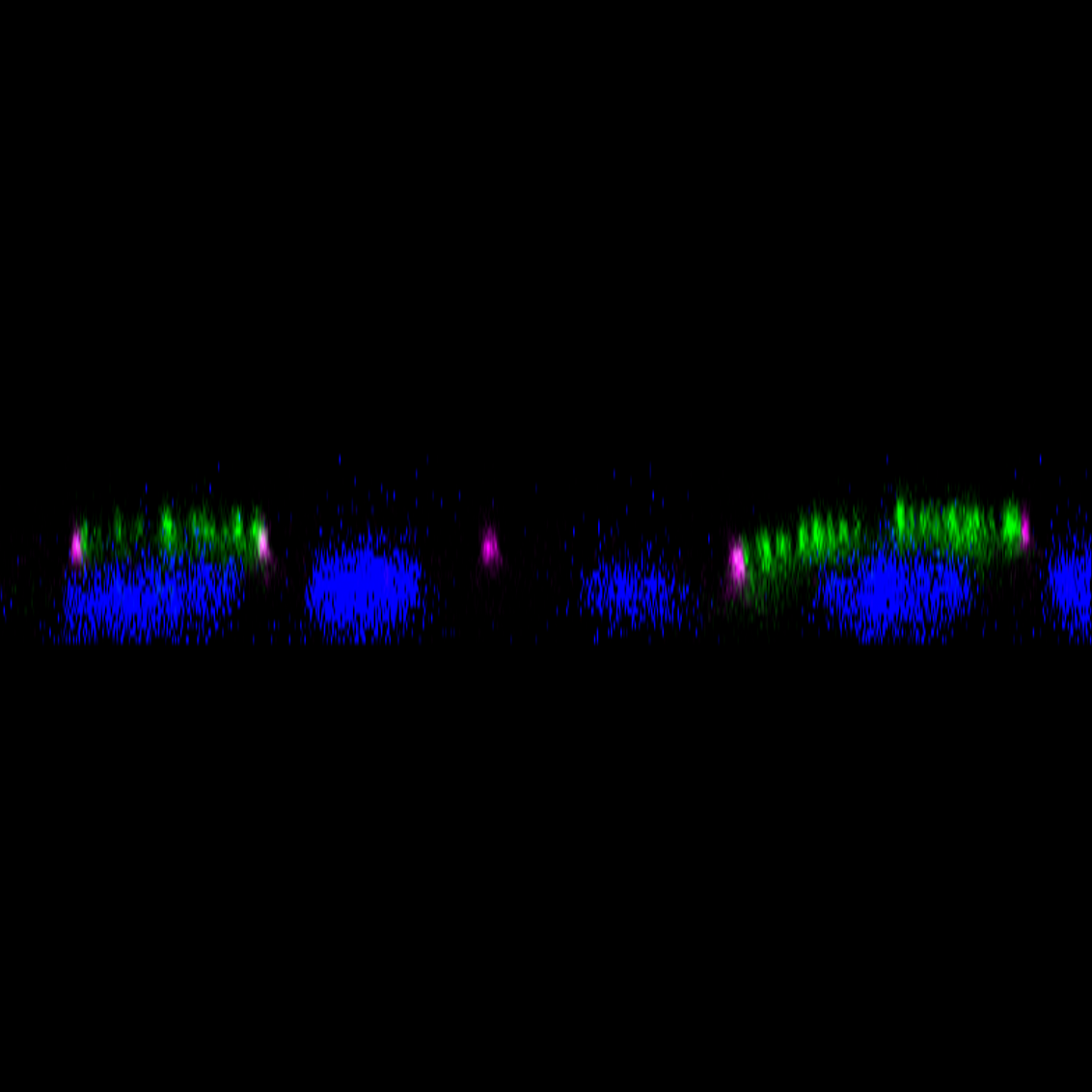

Supplement: Supplementary file 6 — Source Data Fig. 5 [file 44319_2023_18_MOESM6_ESM.zip › Figure_4/4A/4A xz images/12h_right_merge.tif]

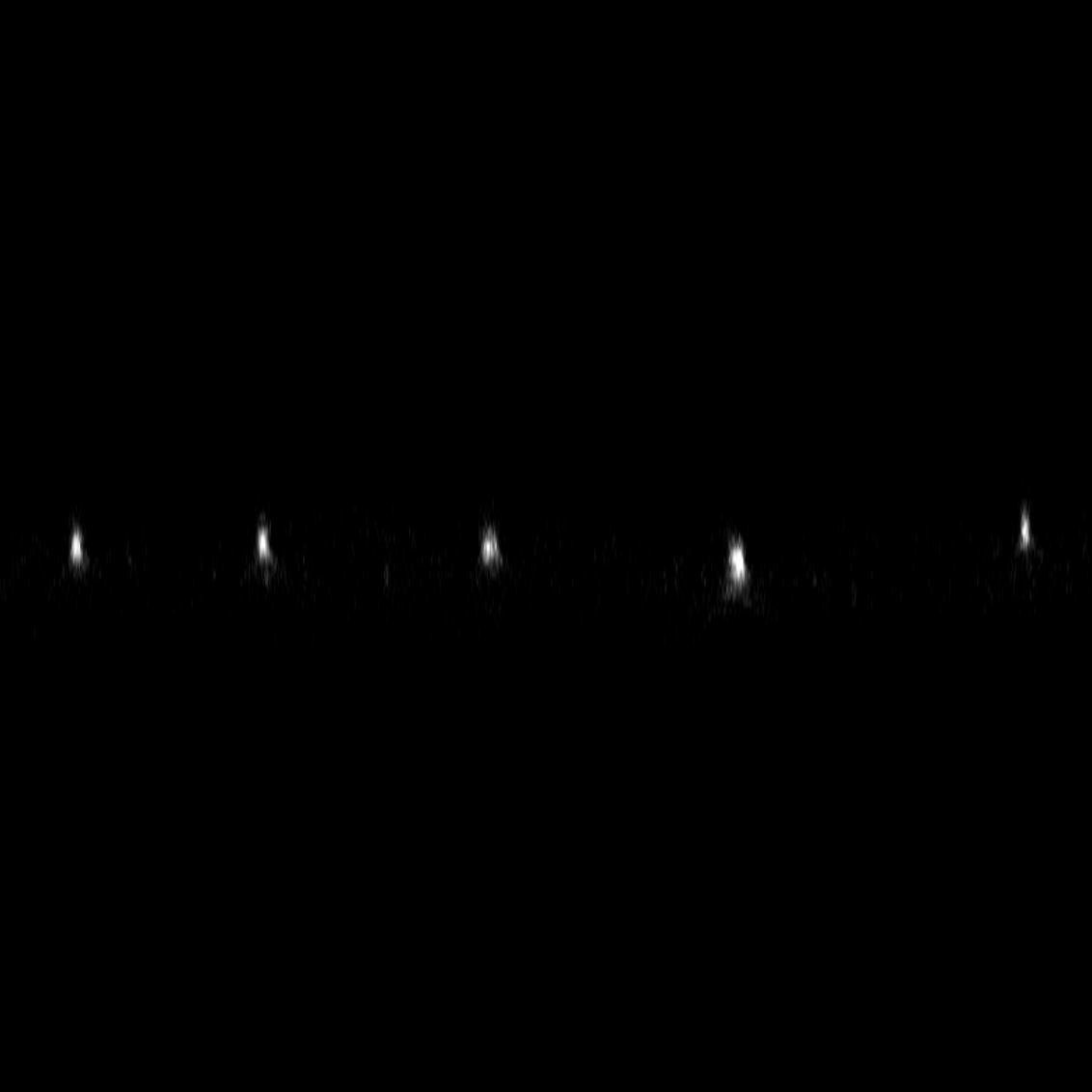

Supplement: Supplementary file 6 — Source Data Fig. 5 [file 44319_2023_18_MOESM6_ESM.zip › Figure_4/4A/4A xz images/12h_right_ZO1.tif]

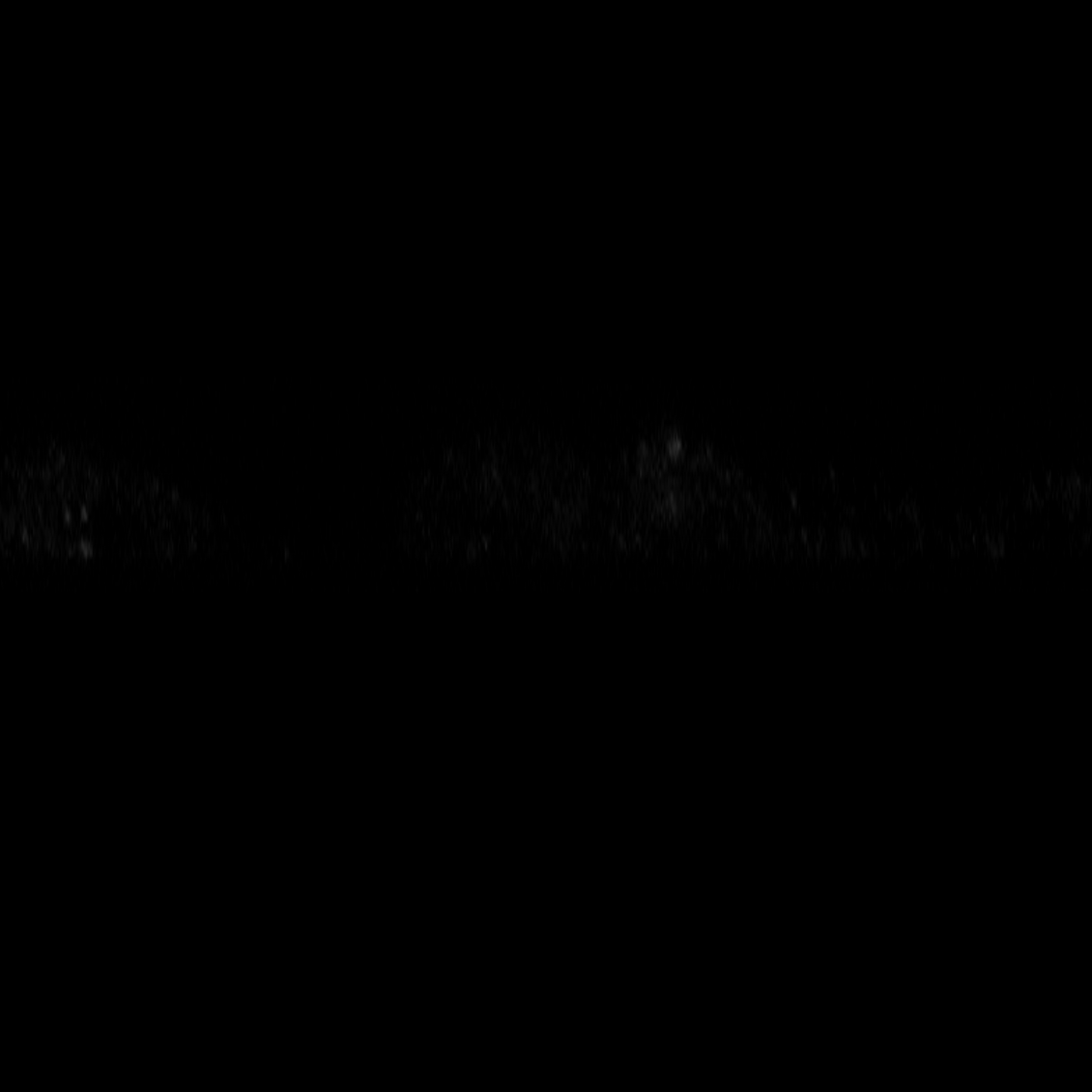

Supplement: Supplementary file 6 — Source Data Fig. 5 [file 44319_2023_18_MOESM6_ESM.zip › Figure_4/4A/4A xz images/0h_middle_ZO1.tif]

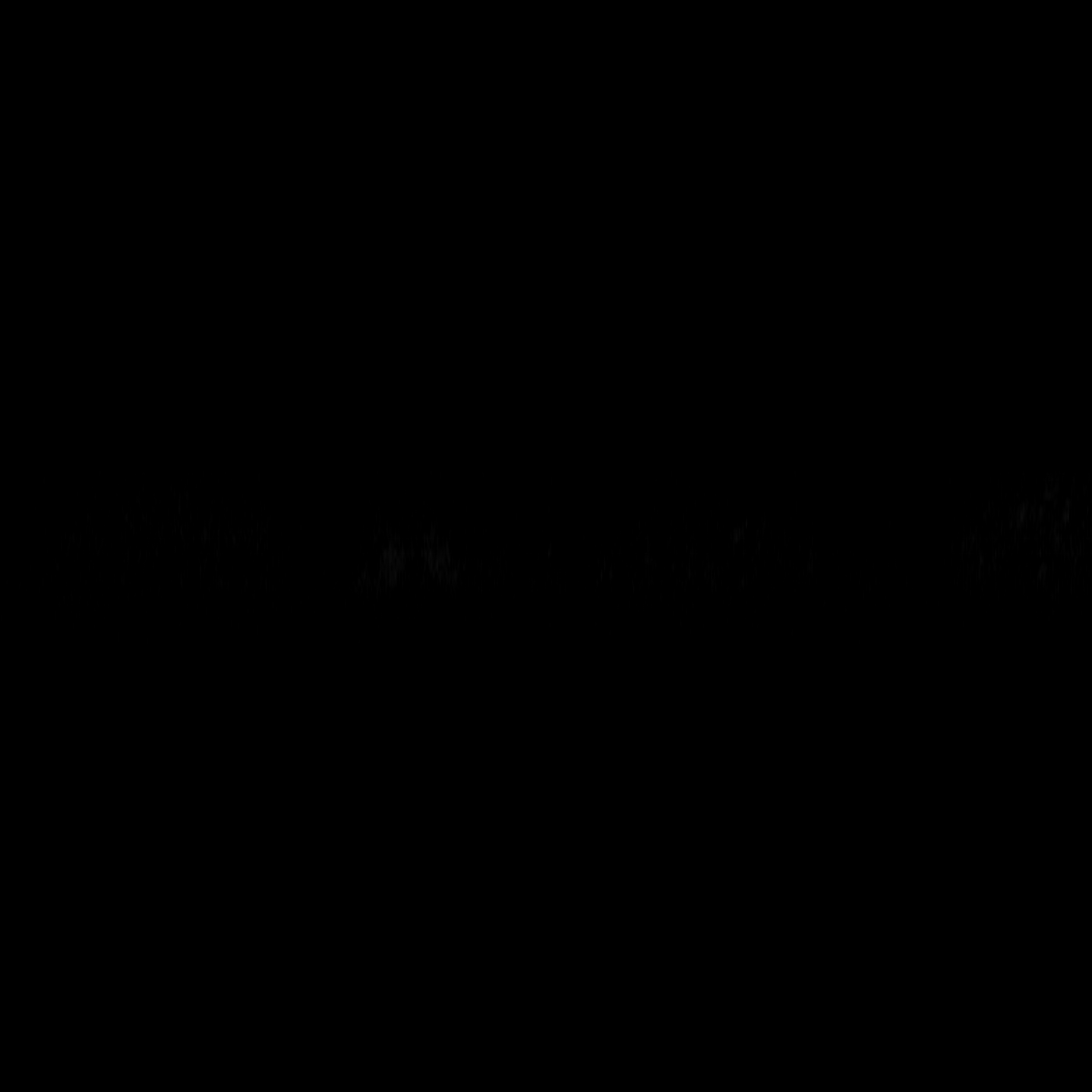

Supplement: Supplementary file 6 — Source Data Fig. 5 [file 44319_2023_18_MOESM6_ESM.zip › Figure_4/4A/4A xz images/0h_left_ZO1.tif]

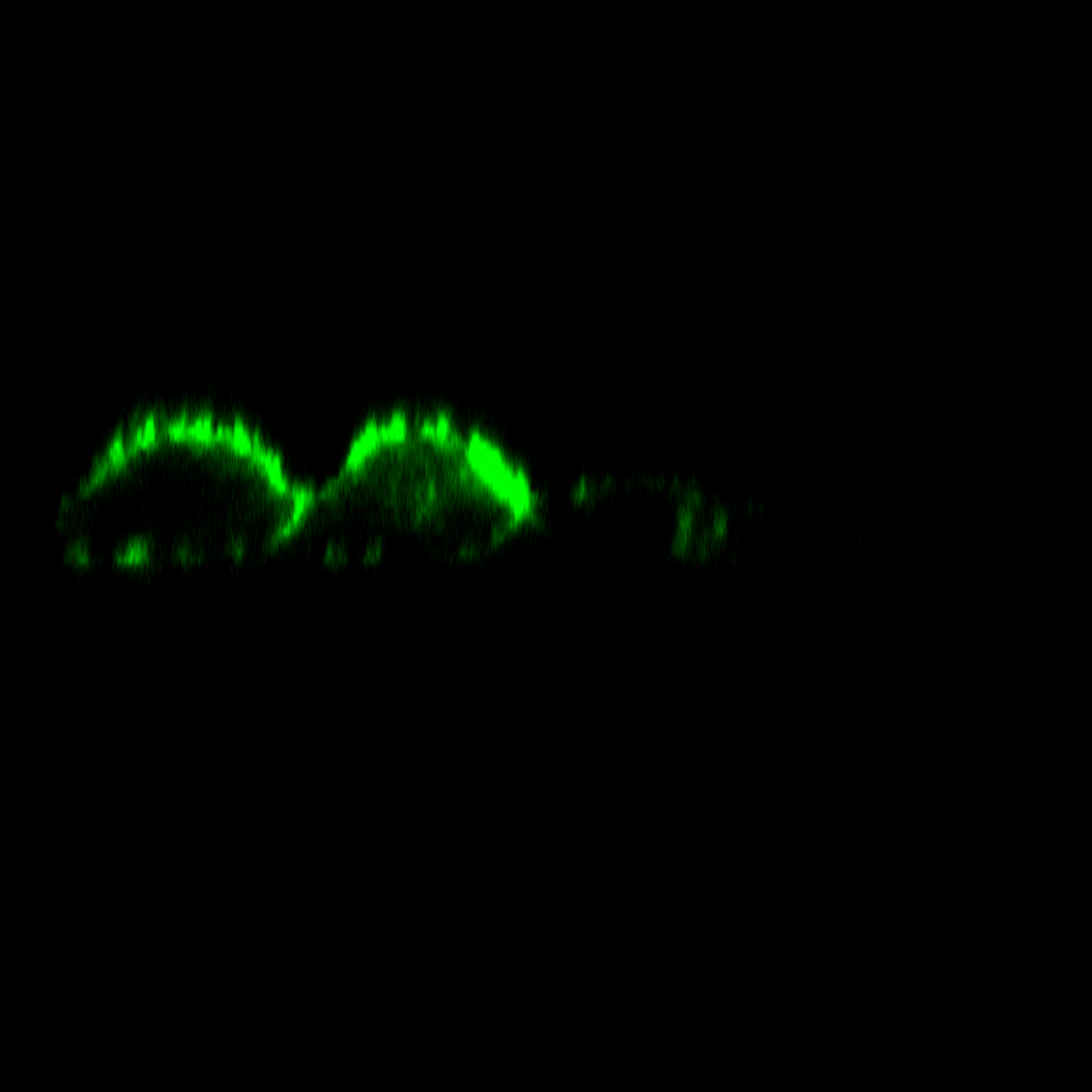

Supplement: Supplementary file 6 — Source Data Fig. 5 [file 44319_2023_18_MOESM6_ESM.zip › Figure_4/4A/4A xz images/0h_right_FLAG.tif]

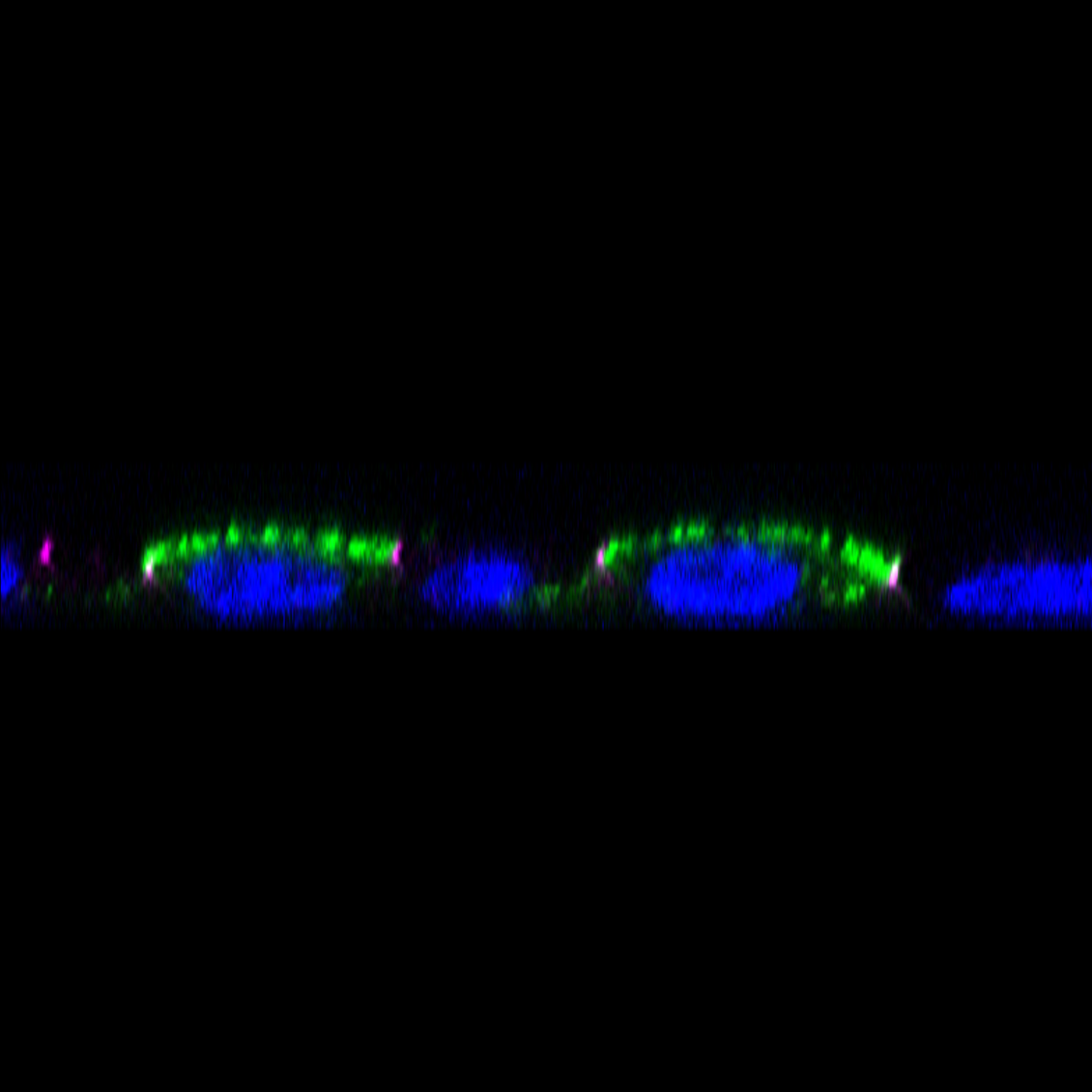

Supplement: Supplementary file 6 — Source Data Fig. 5 [file 44319_2023_18_MOESM6_ESM.zip › Figure_4/4A/4A xz images/12h_middle_merge.tif]

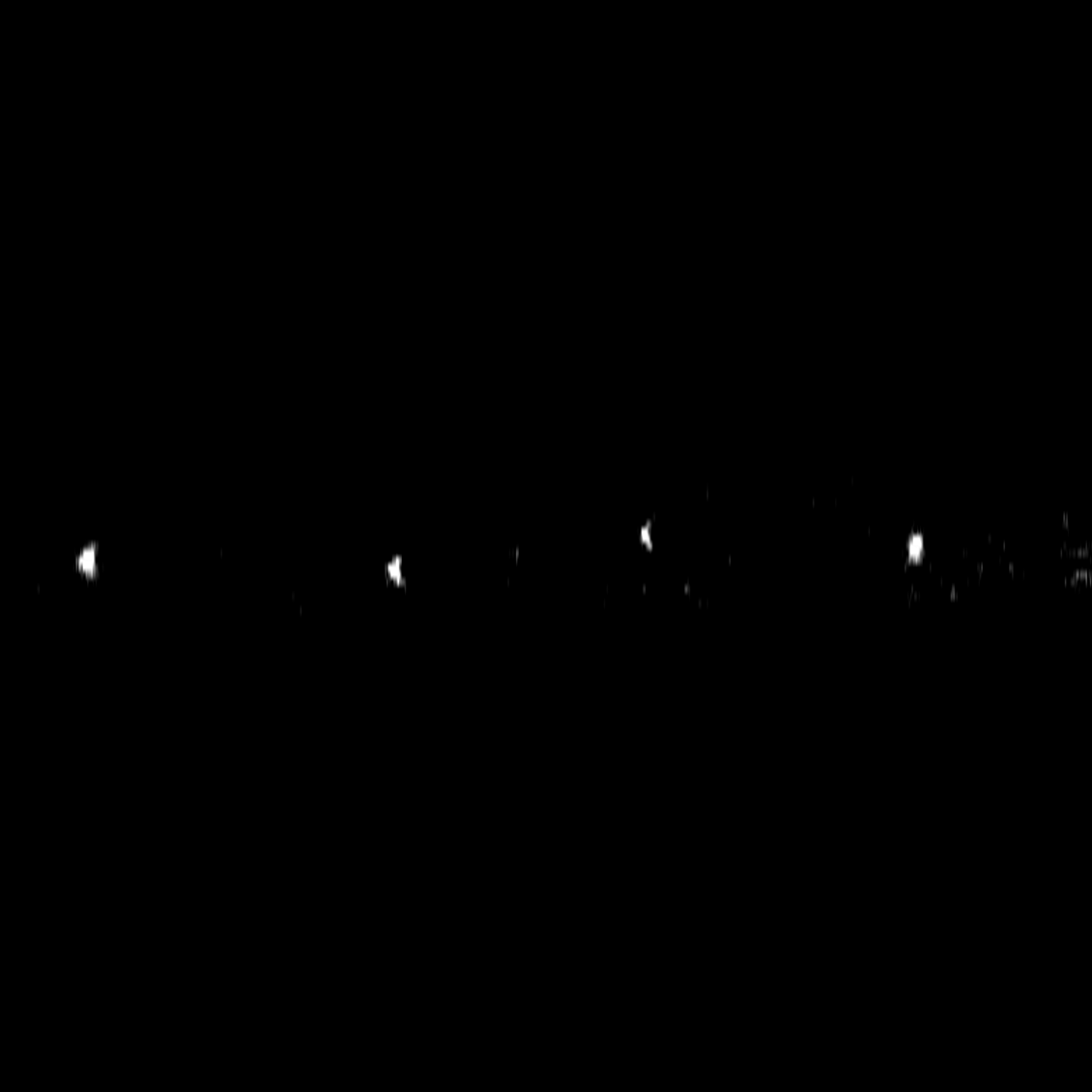

Supplement: Supplementary file 6 — Source Data Fig. 5 [file 44319_2023_18_MOESM6_ESM.zip › Figure_4/4A/4A xz images/12h_left_ZO1.tif]

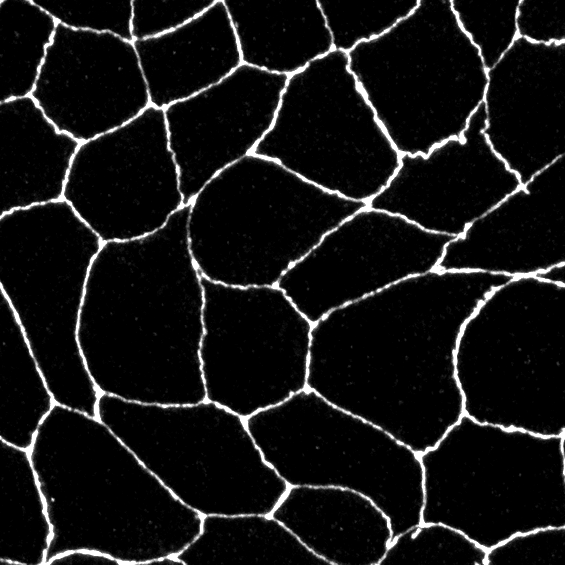

Supplement: Supplementary file 6 — Source Data Fig. 5 [file 44319_2023_18_MOESM6_ESM.zip › Figure_4/4A/4A xy images/12h_middle_ZO1.tif]

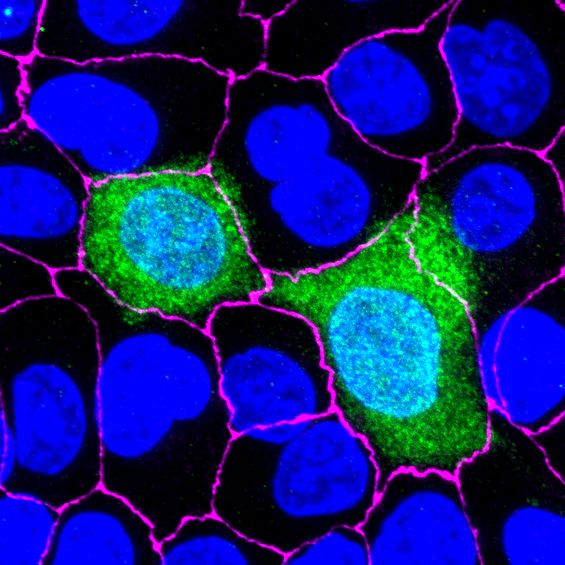

Supplement: Supplementary file 6 — Source Data Fig. 5 [file 44319_2023_18_MOESM6_ESM.zip › Figure_4/4A/4A xy images/6h_left_merge.tif]

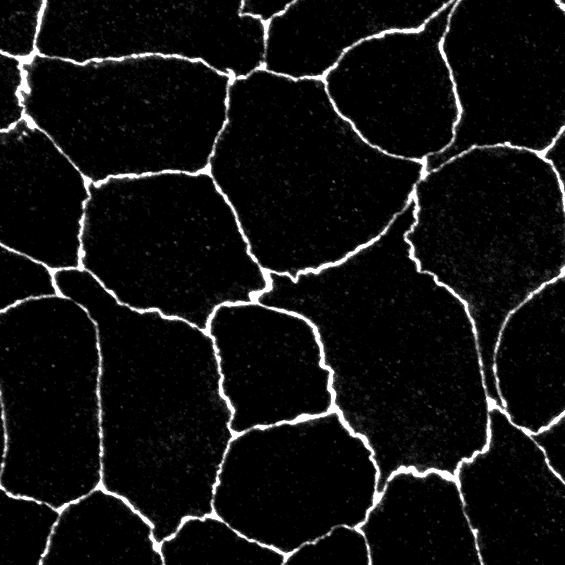

Supplement: Supplementary file 6 — Source Data Fig. 5 [file 44319_2023_18_MOESM6_ESM.zip › Figure_4/4A/4A xy images/6h_left_ZO1.tif]

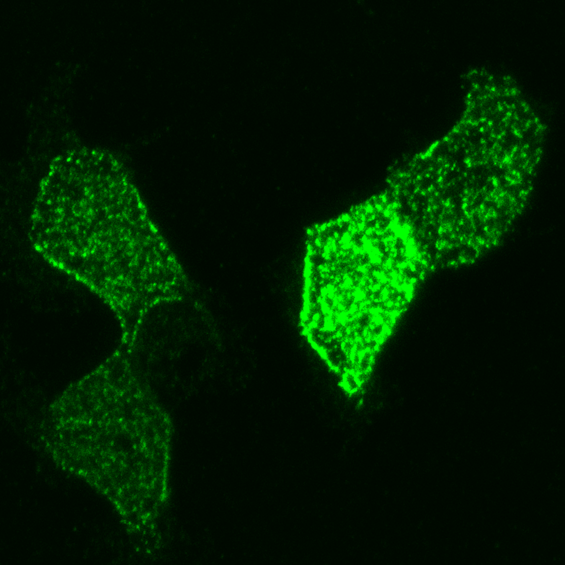

Supplement: Supplementary file 6 — Source Data Fig. 5 [file 44319_2023_18_MOESM6_ESM.zip › Figure_4/4A/4A xy images/12h_right_FLAG.tif]

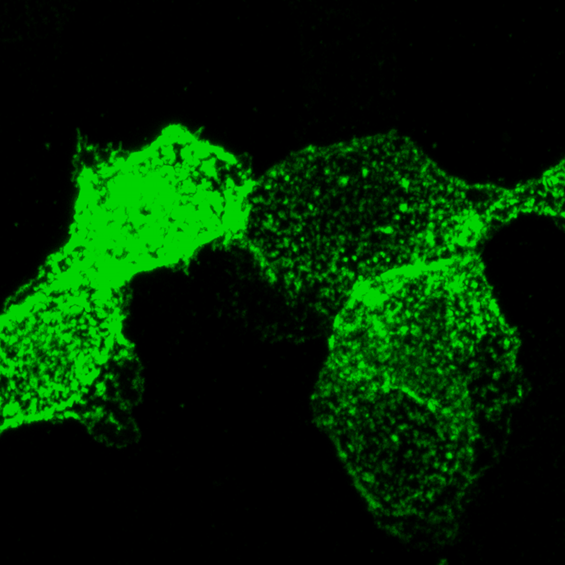

Supplement: Supplementary file 6 — Source Data Fig. 5 [file 44319_2023_18_MOESM6_ESM.zip › Figure_4/4A/4A xy images/6h_middle_FLAG.tif]

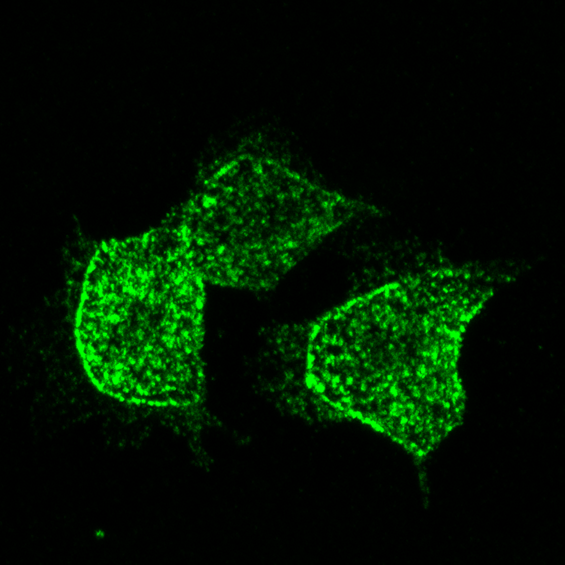

Supplement: Supplementary file 6 — Source Data Fig. 5 [file 44319_2023_18_MOESM6_ESM.zip › Figure_4/4A/4A xy images/12h_middle_FLAG.tif]

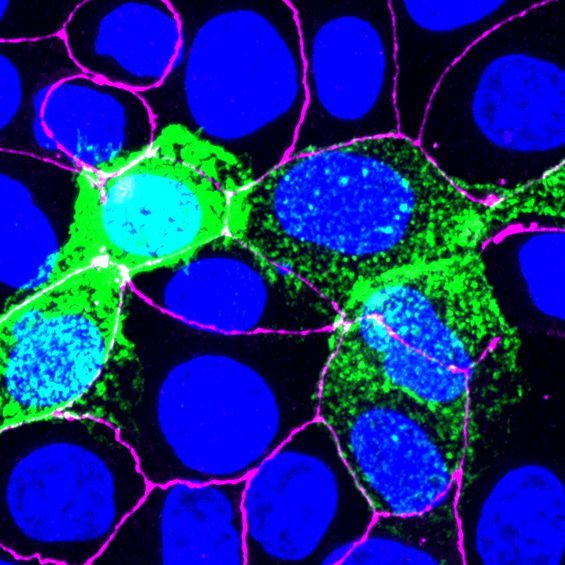

Supplement: Supplementary file 6 — Source Data Fig. 5 [file 44319_2023_18_MOESM6_ESM.zip › Figure_4/4A/4A xy images/6h_middle_merge.tif]

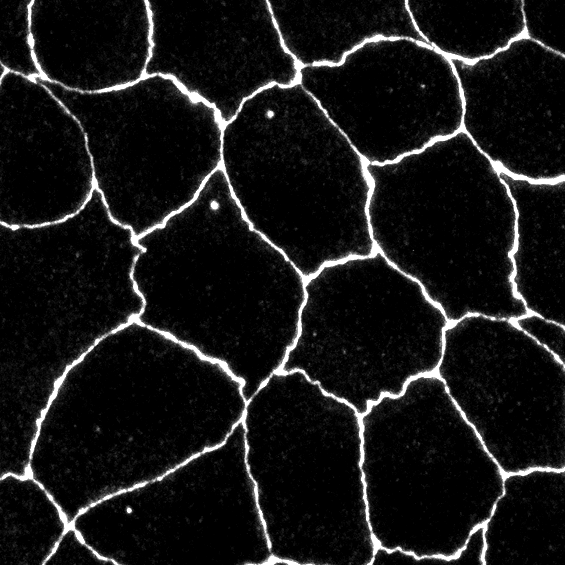

Supplement: Supplementary file 6 — Source Data Fig. 5 [file 44319_2023_18_MOESM6_ESM.zip › Figure_4/4A/4A xy images/6h_right_ZO1.tif]

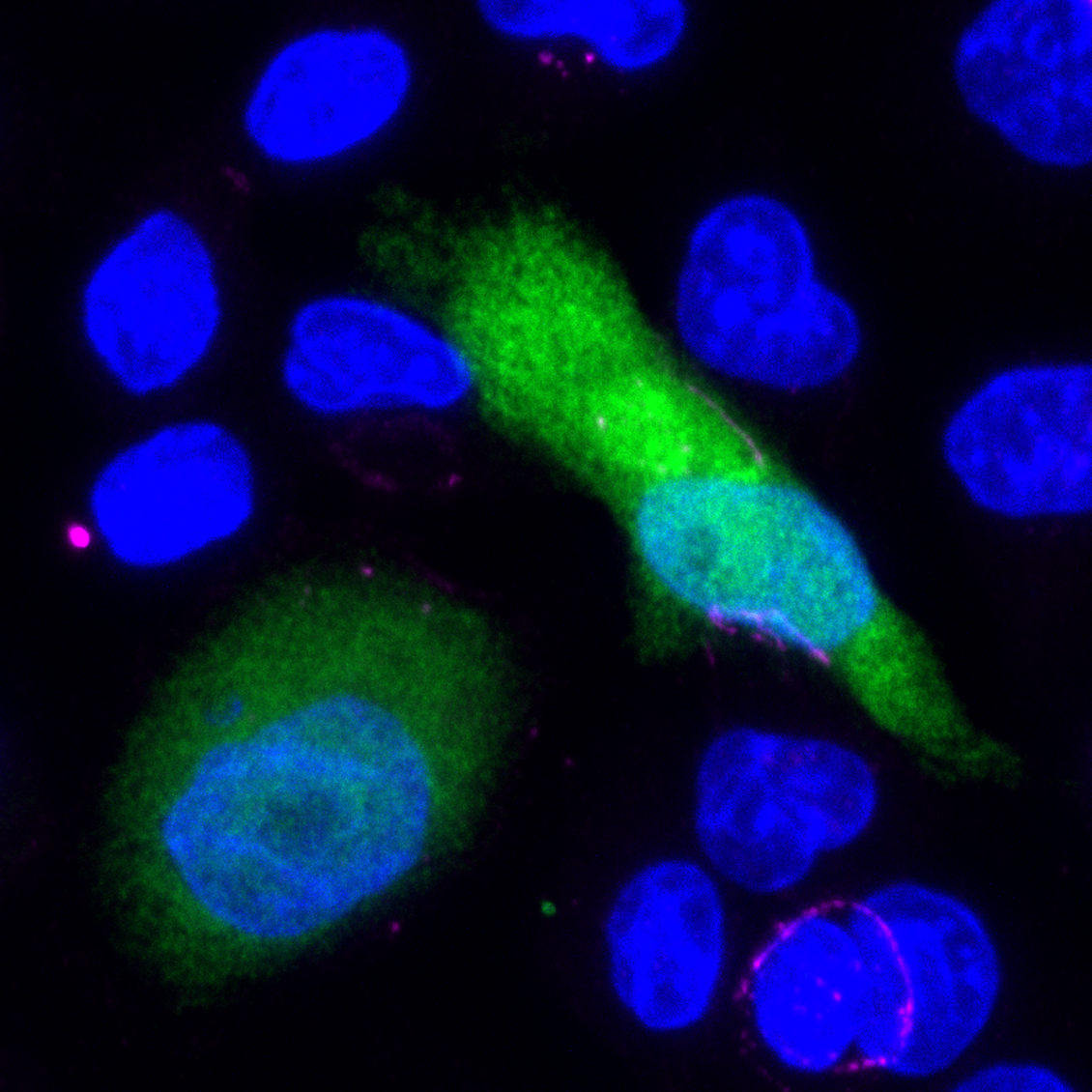

Supplement: Supplementary file 6 — Source Data Fig. 5 [file 44319_2023_18_MOESM6_ESM.zip › Figure_4/4A/4A xy images/0h_left_merge.tif]

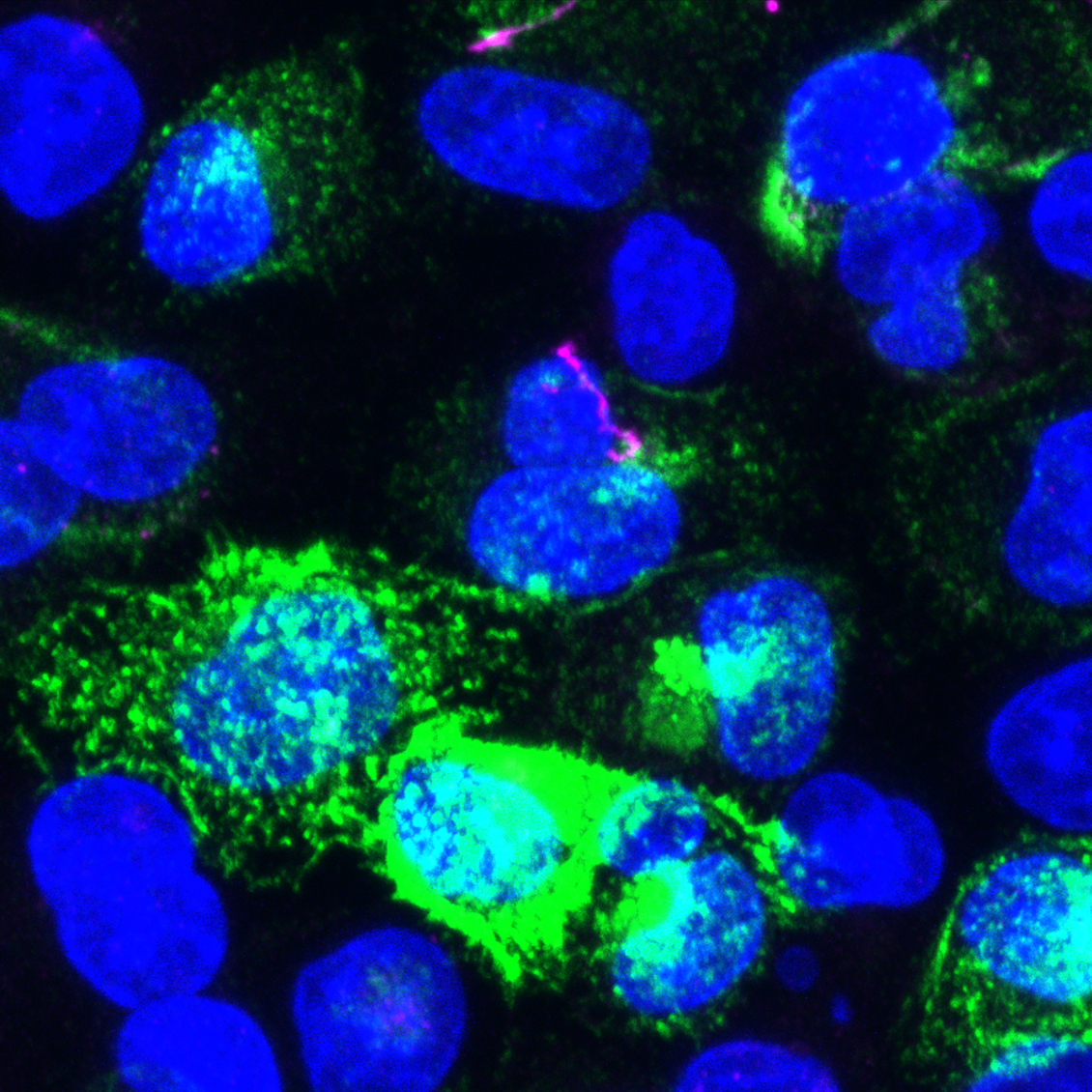

Supplement: Supplementary file 6 — Source Data Fig. 5 [file 44319_2023_18_MOESM6_ESM.zip › Figure_4/4A/4A xy images/0h_middle_merge.tif]

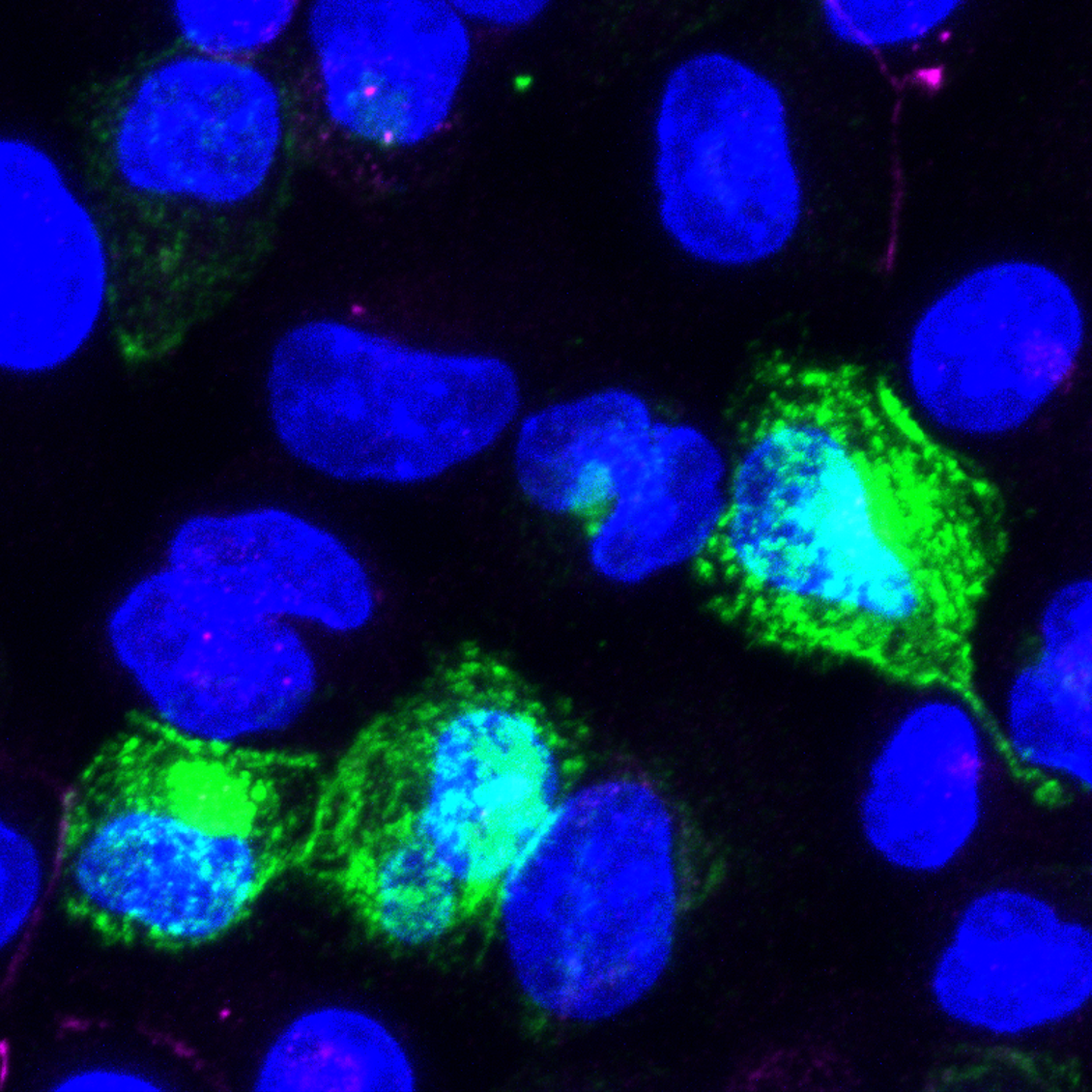

Supplement: Supplementary file 6 — Source Data Fig. 5 [file 44319_2023_18_MOESM6_ESM.zip › Figure_4/4A/4A xy images/0h_right_merge.tif]

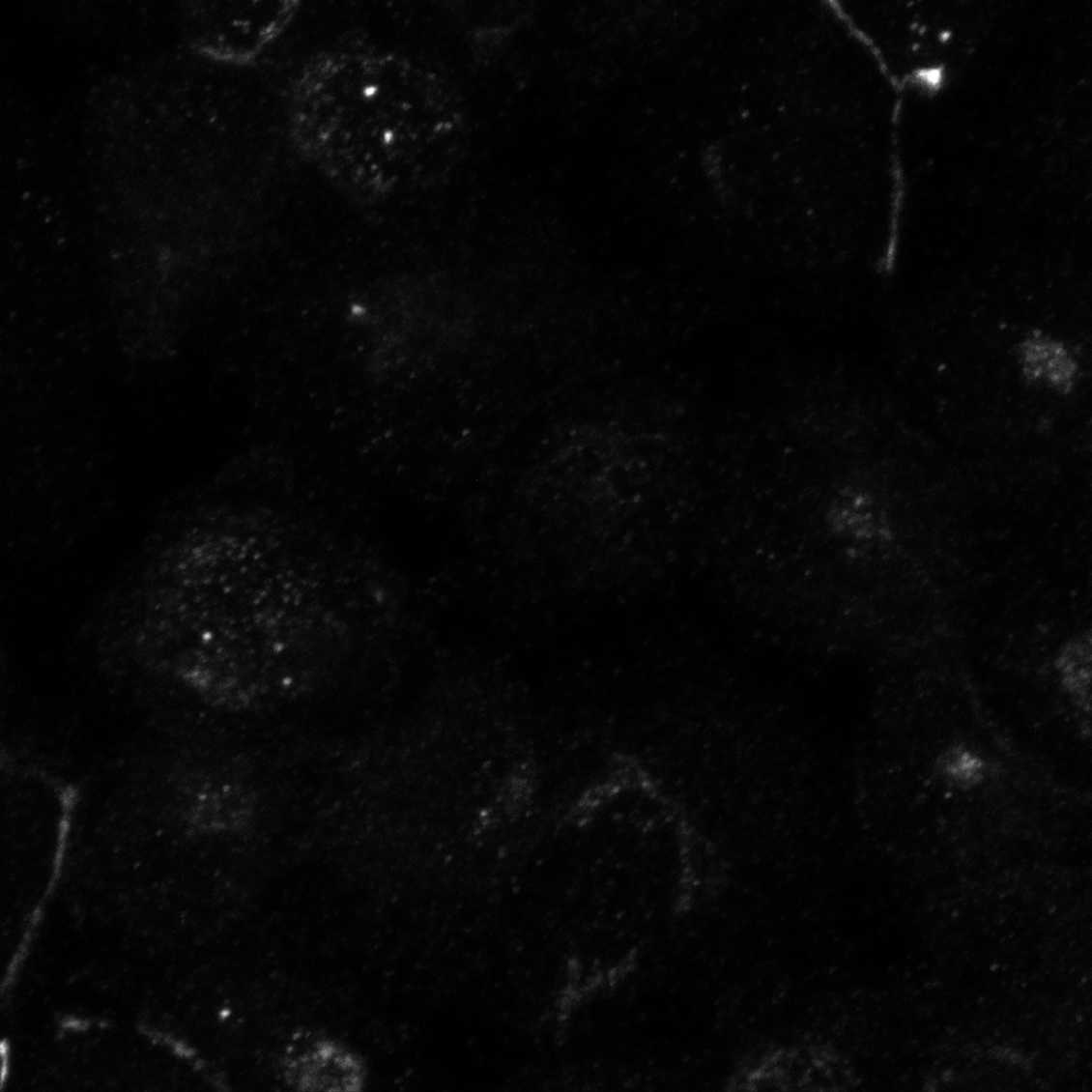

Supplement: Supplementary file 6 — Source Data Fig. 5 [file 44319_2023_18_MOESM6_ESM.zip › Figure_4/4A/4A xy images/0h_right_ZO1.tif]

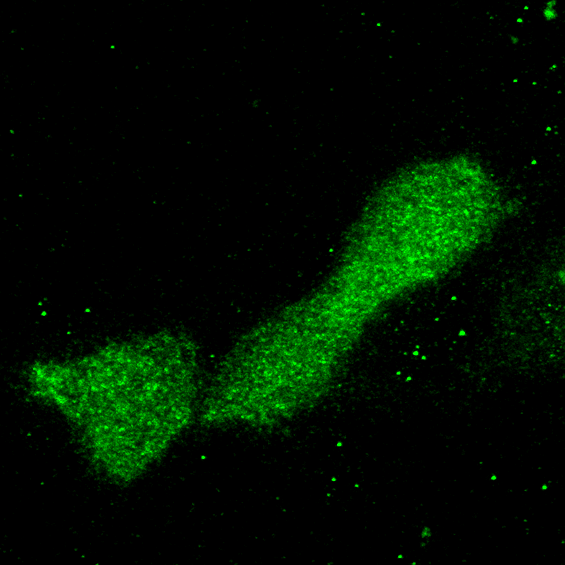

Supplement: Supplementary file 6 — Source Data Fig. 5 [file 44319_2023_18_MOESM6_ESM.zip › Figure_4/4A/4A xy images/12h_left_GFP.tif]

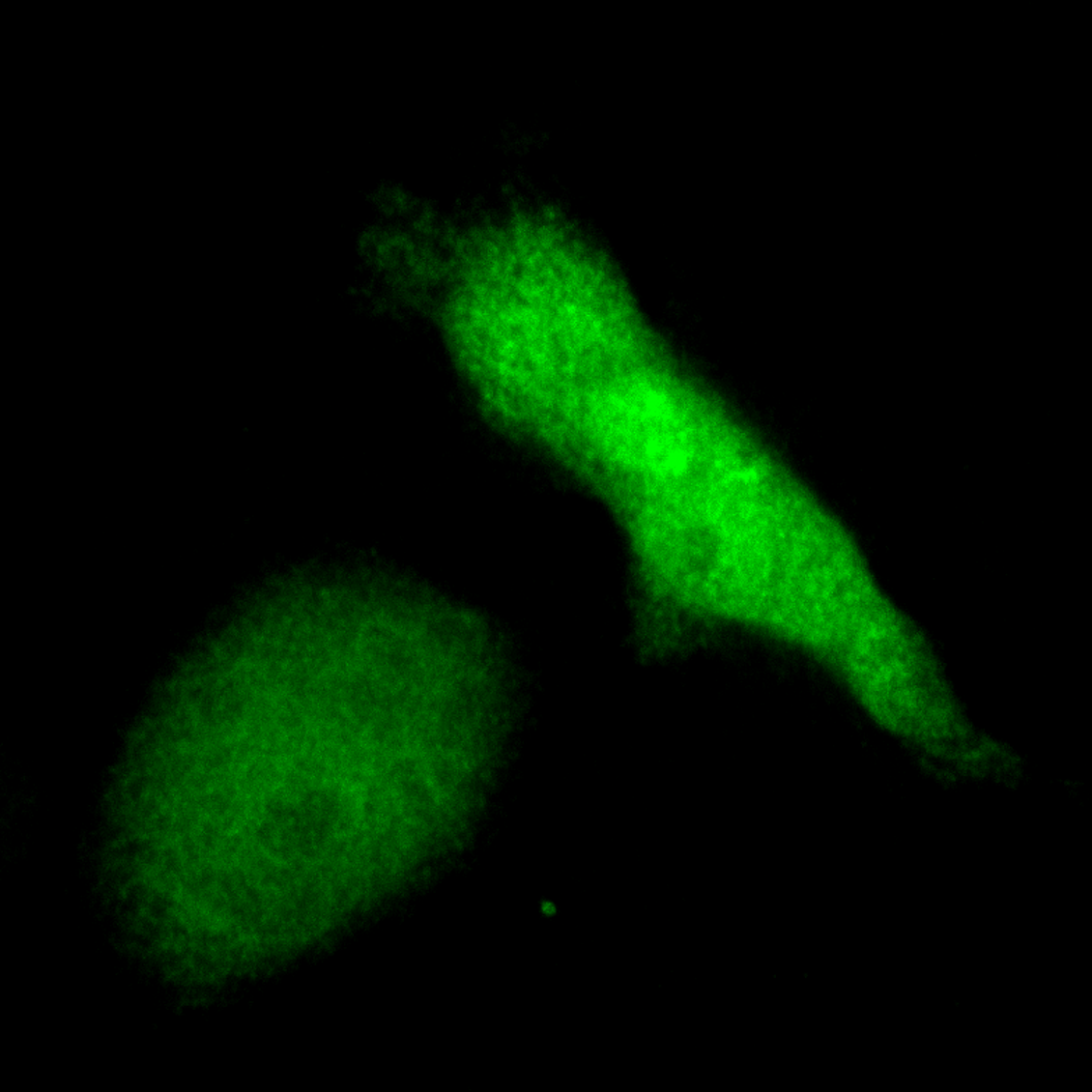

Supplement: Supplementary file 6 — Source Data Fig. 5 [file 44319_2023_18_MOESM6_ESM.zip › Figure_4/4A/4A xy images/0h_left_GFP.tif]

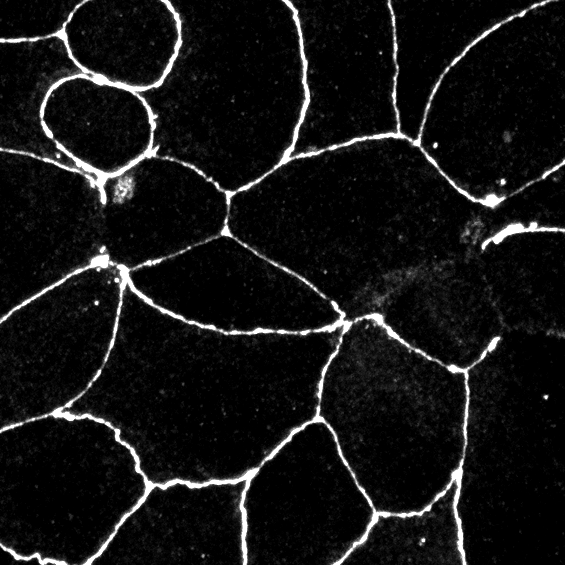

Supplement: Supplementary file 6 — Source Data Fig. 5 [file 44319_2023_18_MOESM6_ESM.zip › Figure_4/4A/4A xy images/6h_middle_ZO1.tif]

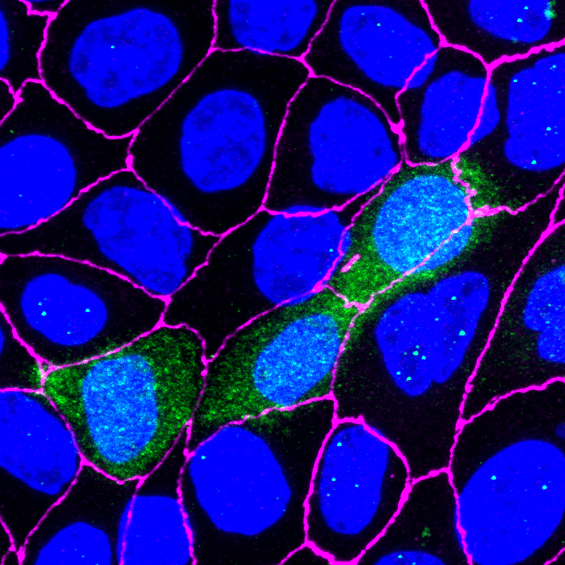

Supplement: Supplementary file 6 — Source Data Fig. 5 [file 44319_2023_18_MOESM6_ESM.zip › Figure_4/4A/4A xy images/12h_left_merge.tif]

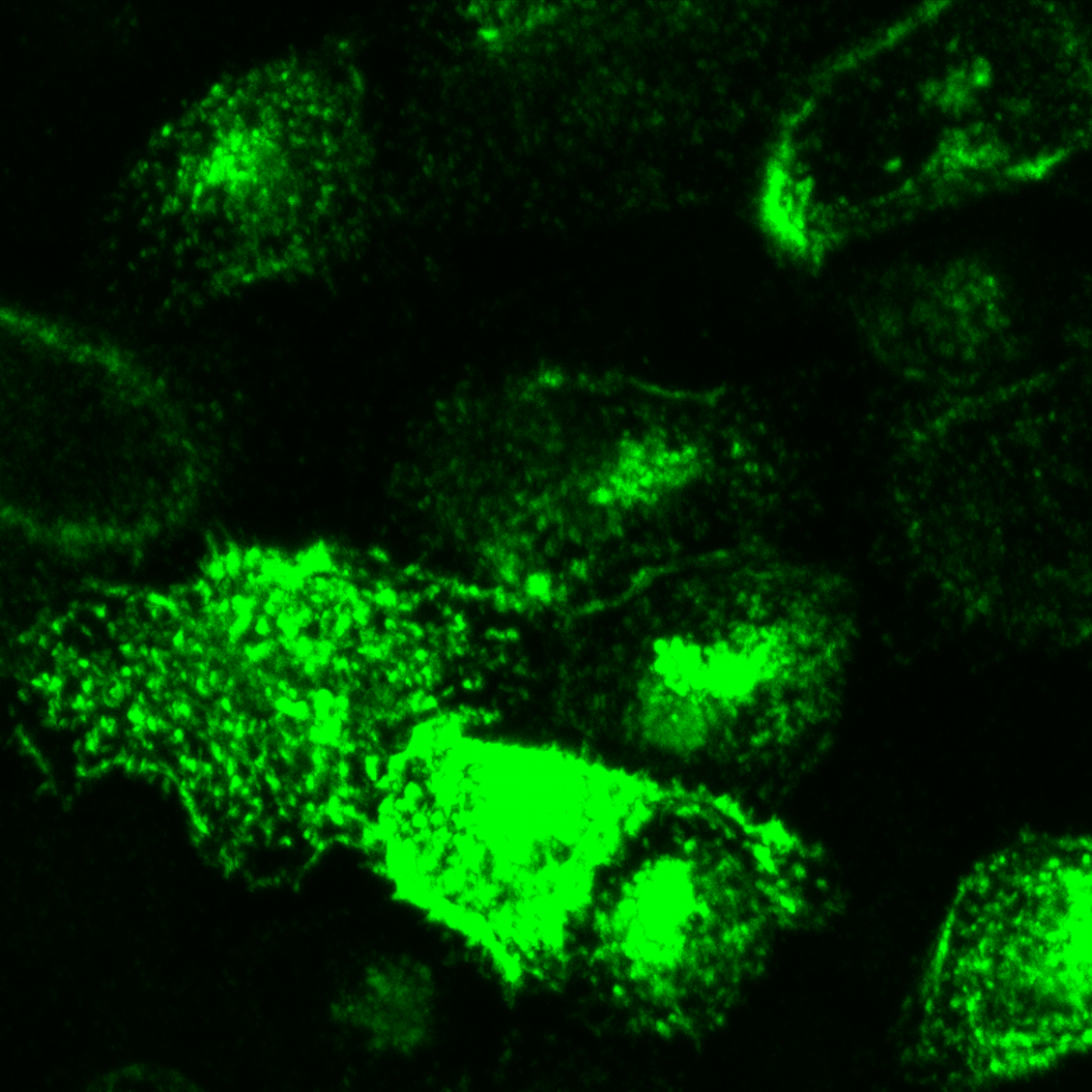

Supplement: Supplementary file 6 — Source Data Fig. 5 [file 44319_2023_18_MOESM6_ESM.zip › Figure_4/4A/4A xy images/0h_middle_FLAG.tif]

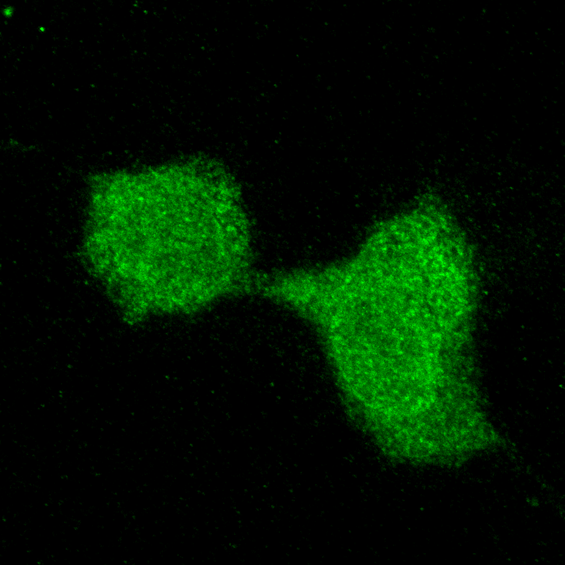

Supplement: Supplementary file 6 — Source Data Fig. 5 [file 44319_2023_18_MOESM6_ESM.zip › Figure_4/4A/4A xy images/6h_left_GFP.tif]

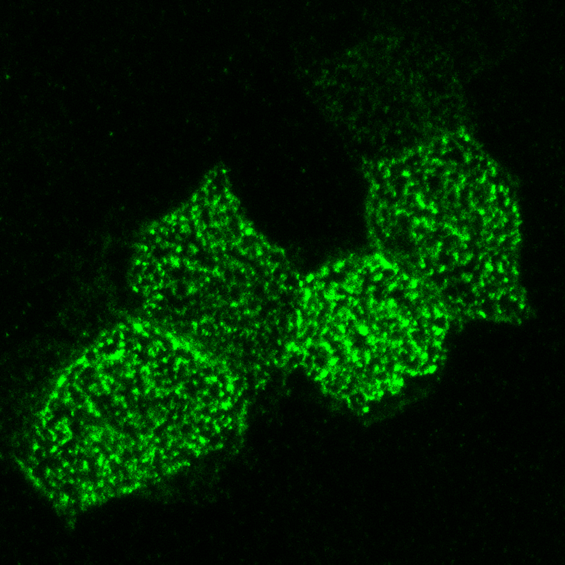

Supplement: Supplementary file 6 — Source Data Fig. 5 [file 44319_2023_18_MOESM6_ESM.zip › Figure_4/4A/4A xy images/6h_right_FLAG.tif]

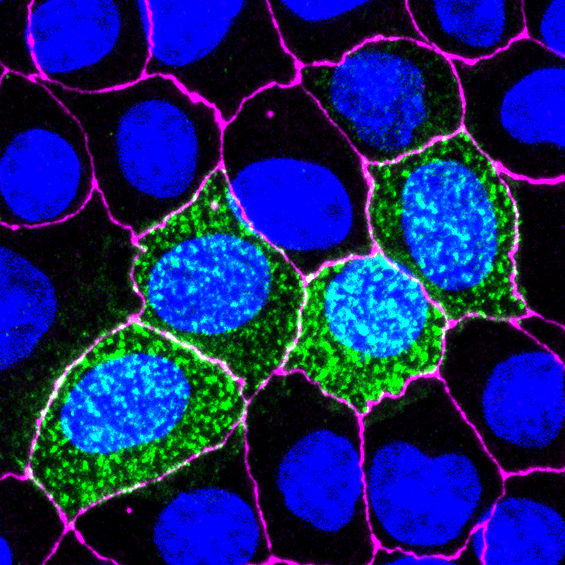

Supplement: Supplementary file 6 — Source Data Fig. 5 [file 44319_2023_18_MOESM6_ESM.zip › Figure_4/4A/4A xy images/6h_right_merge.tif]

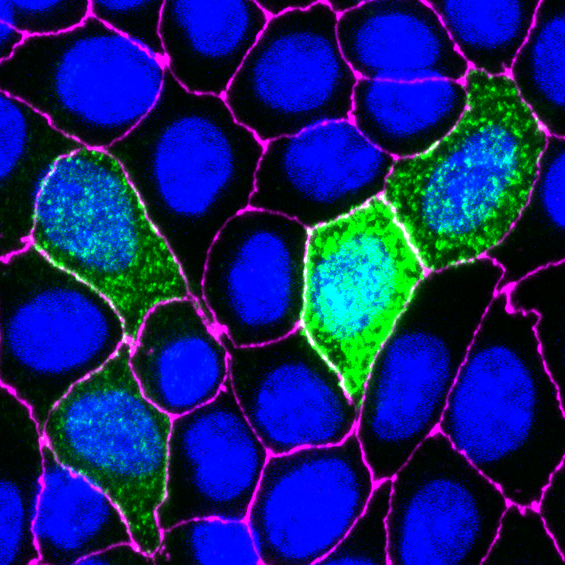

Supplement: Supplementary file 6 — Source Data Fig. 5 [file 44319_2023_18_MOESM6_ESM.zip › Figure_4/4A/4A xy images/12h_right_merge.tif]

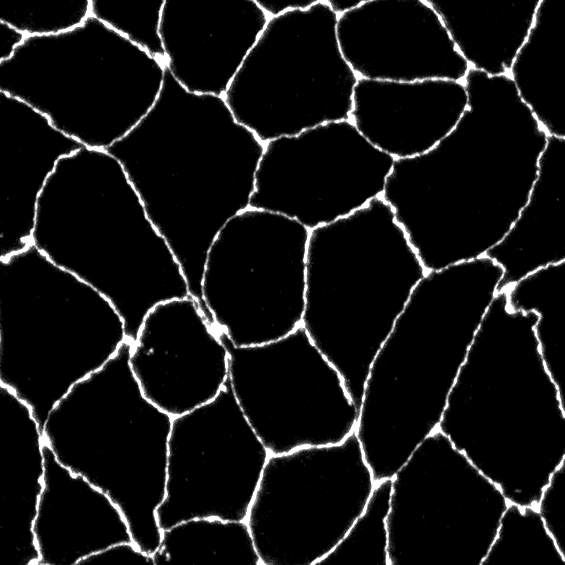

Supplement: Supplementary file 6 — Source Data Fig. 5 [file 44319_2023_18_MOESM6_ESM.zip › Figure_4/4A/4A xy images/12h_right_ZO1.tif]

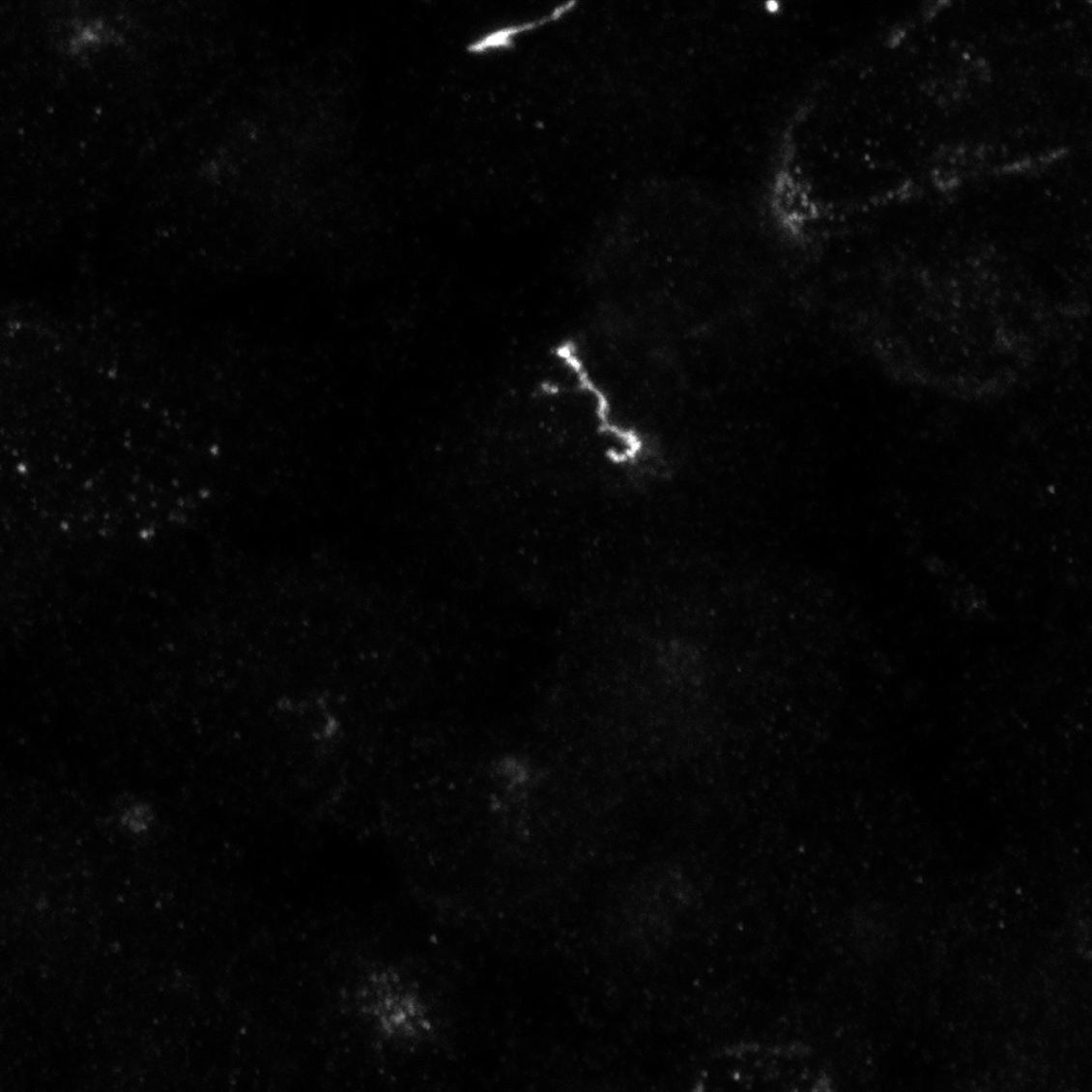

Supplement: Supplementary file 6 — Source Data Fig. 5 [file 44319_2023_18_MOESM6_ESM.zip › Figure_4/4A/4A xy images/0h_middle_ZO1.tif]

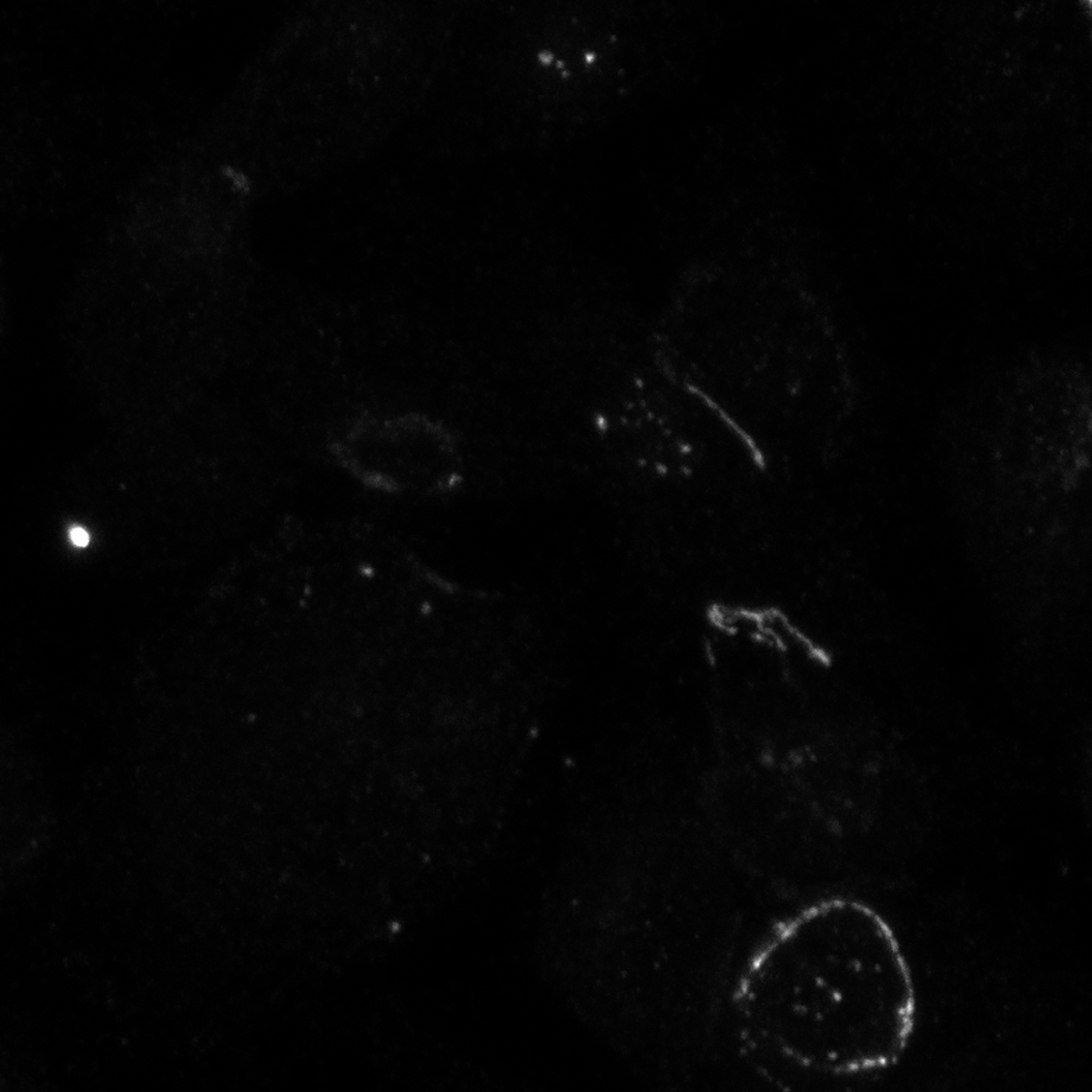

Supplement: Supplementary file 6 — Source Data Fig. 5 [file 44319_2023_18_MOESM6_ESM.zip › Figure_4/4A/4A xy images/0h_left_ZO1.tif]

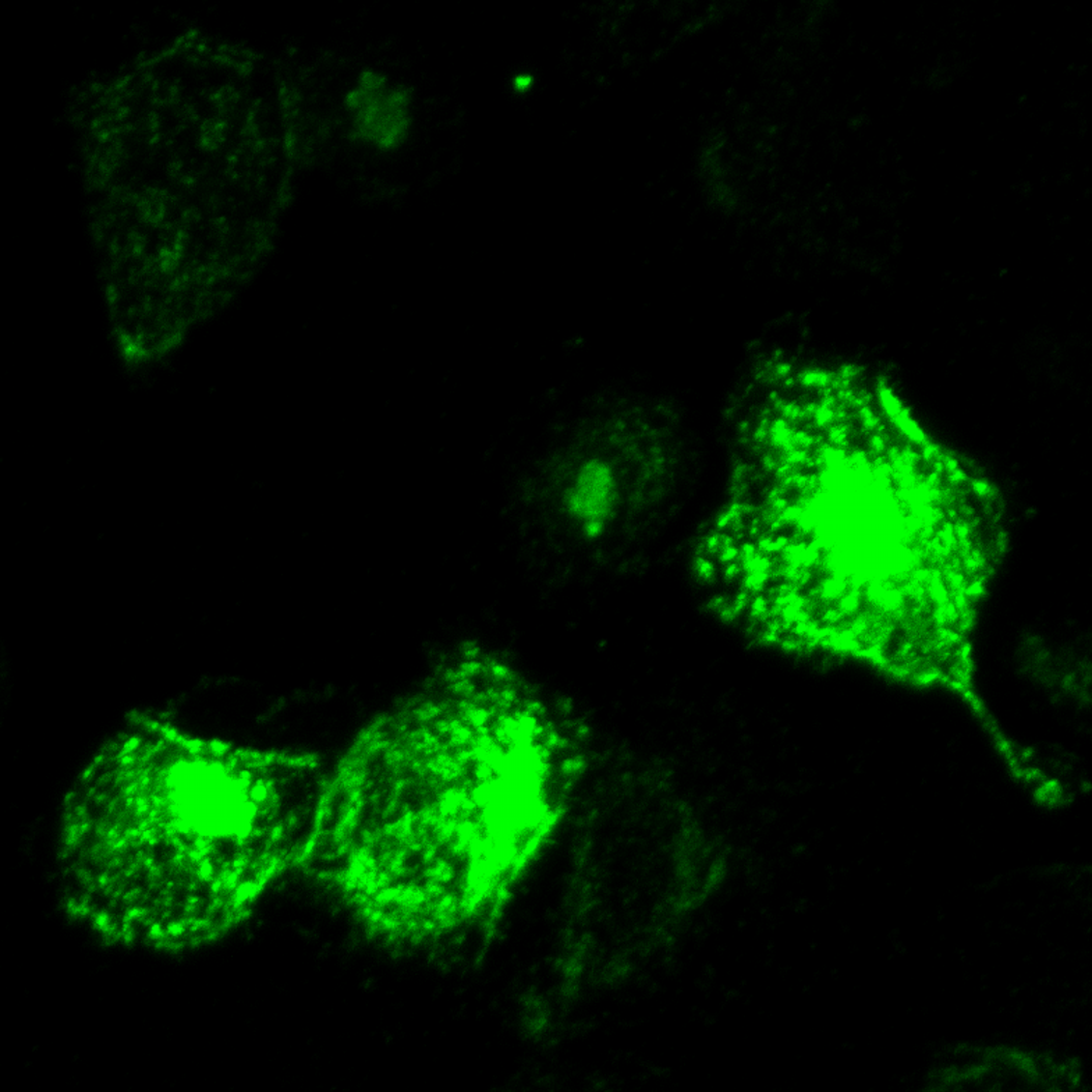

Supplement: Supplementary file 6 — Source Data Fig. 5 [file 44319_2023_18_MOESM6_ESM.zip › Figure_4/4A/4A xy images/0h_right_FLAG.tif]

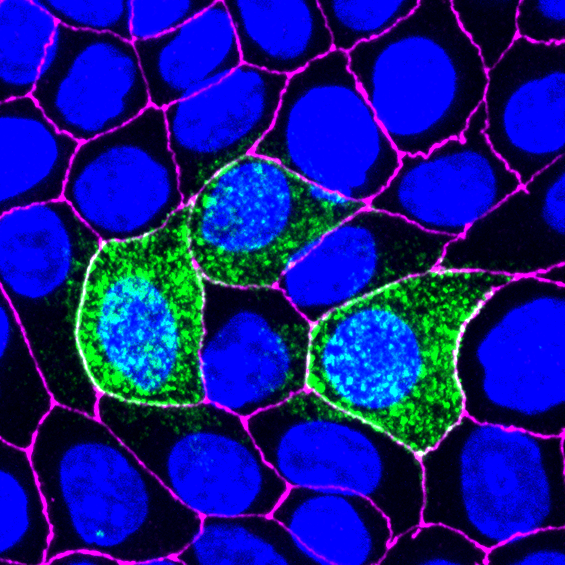

Supplement: Supplementary file 6 — Source Data Fig. 5 [file 44319_2023_18_MOESM6_ESM.zip › Figure_4/4A/4A xy images/12h_middle_merge.tif]

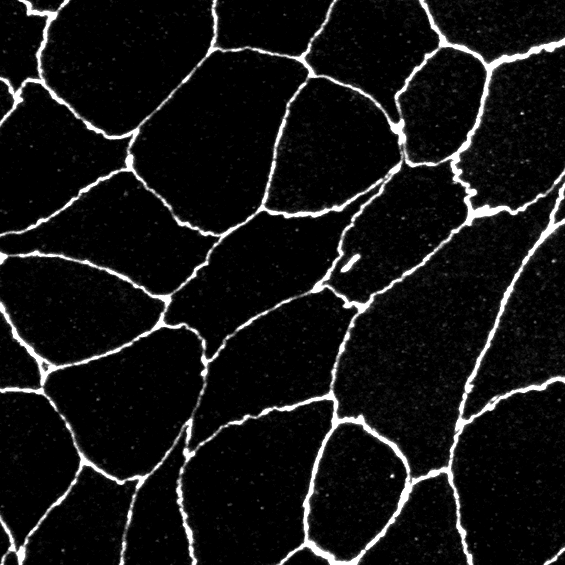

Supplement: Supplementary file 6 — Source Data Fig. 5 [file 44319_2023_18_MOESM6_ESM.zip › Figure_4/4A/4A xy images/12h_left_ZO1.tif]

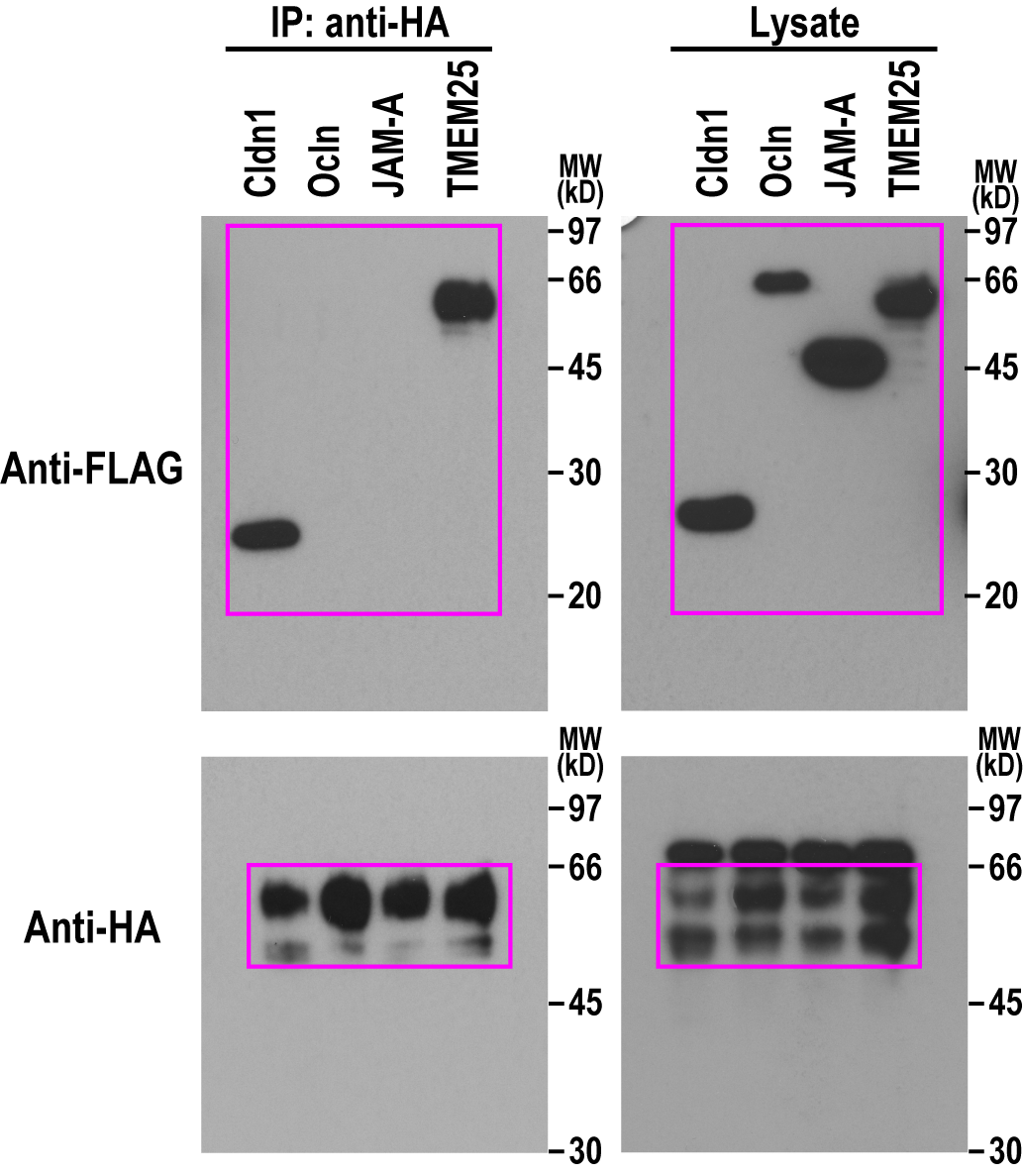

Supplement: Supplementary file 7 — Source Data Fig. 6 [file 44319_2023_18_MOESM7_ESM.zip › Figure_5/5A/5A.tif]

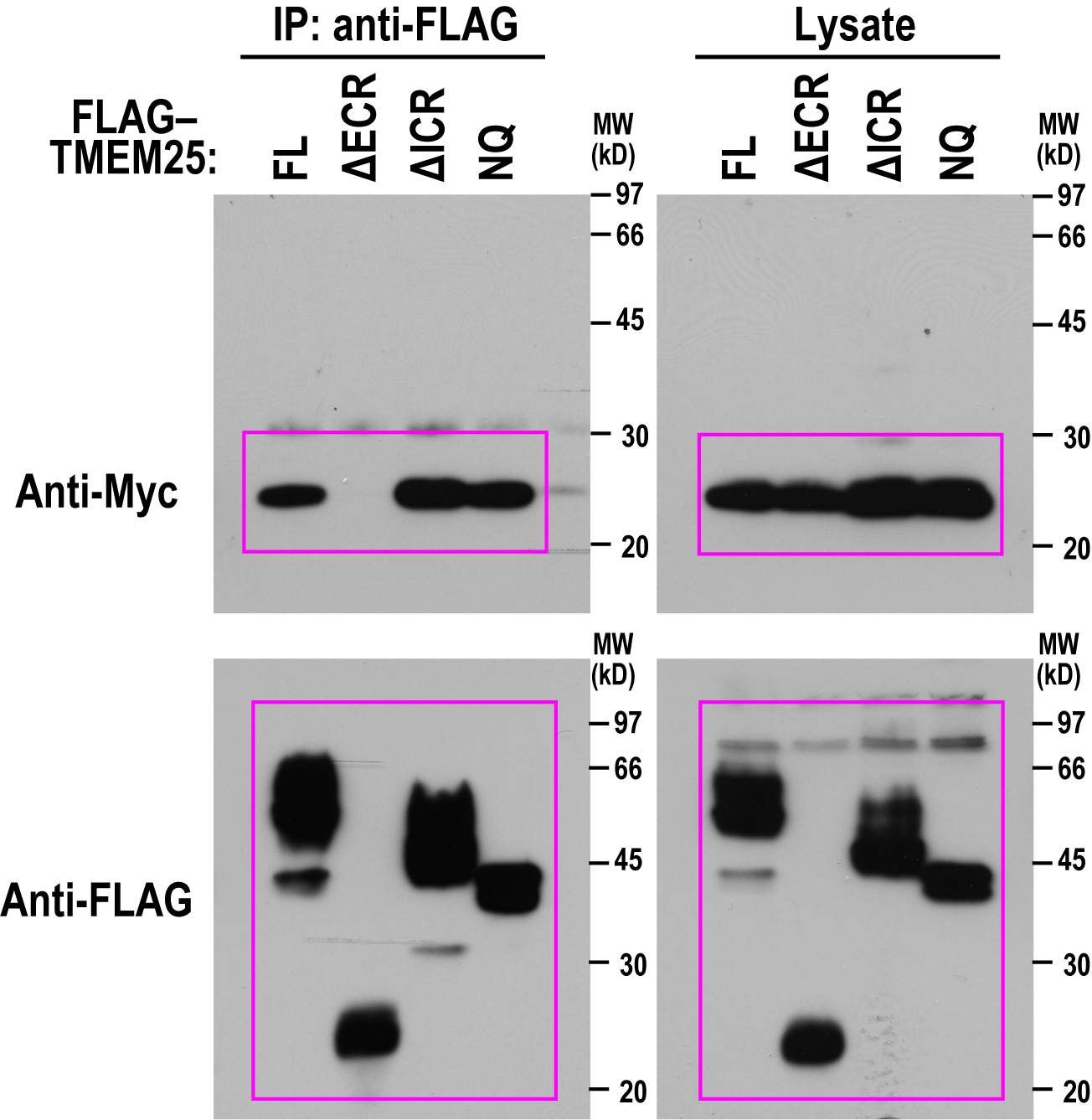

Supplement: Supplementary file 7 — Source Data Fig. 6 [file 44319_2023_18_MOESM7_ESM.zip › Figure_5/5C/5C.tif]

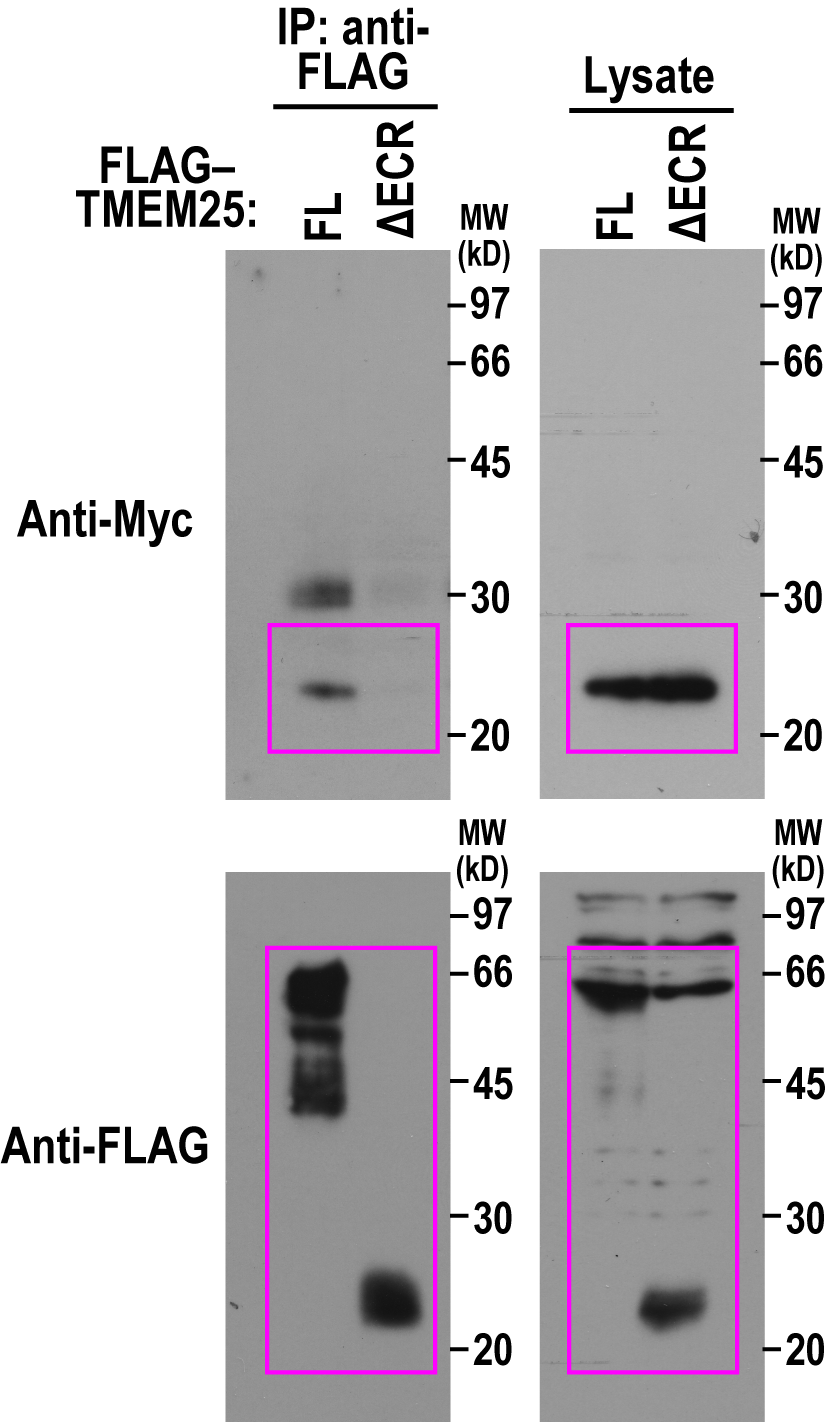

Supplement: Supplementary file 7 — Source Data Fig. 6 [file 44319_2023_18_MOESM7_ESM.zip › Figure_5/5E/5E.tif]

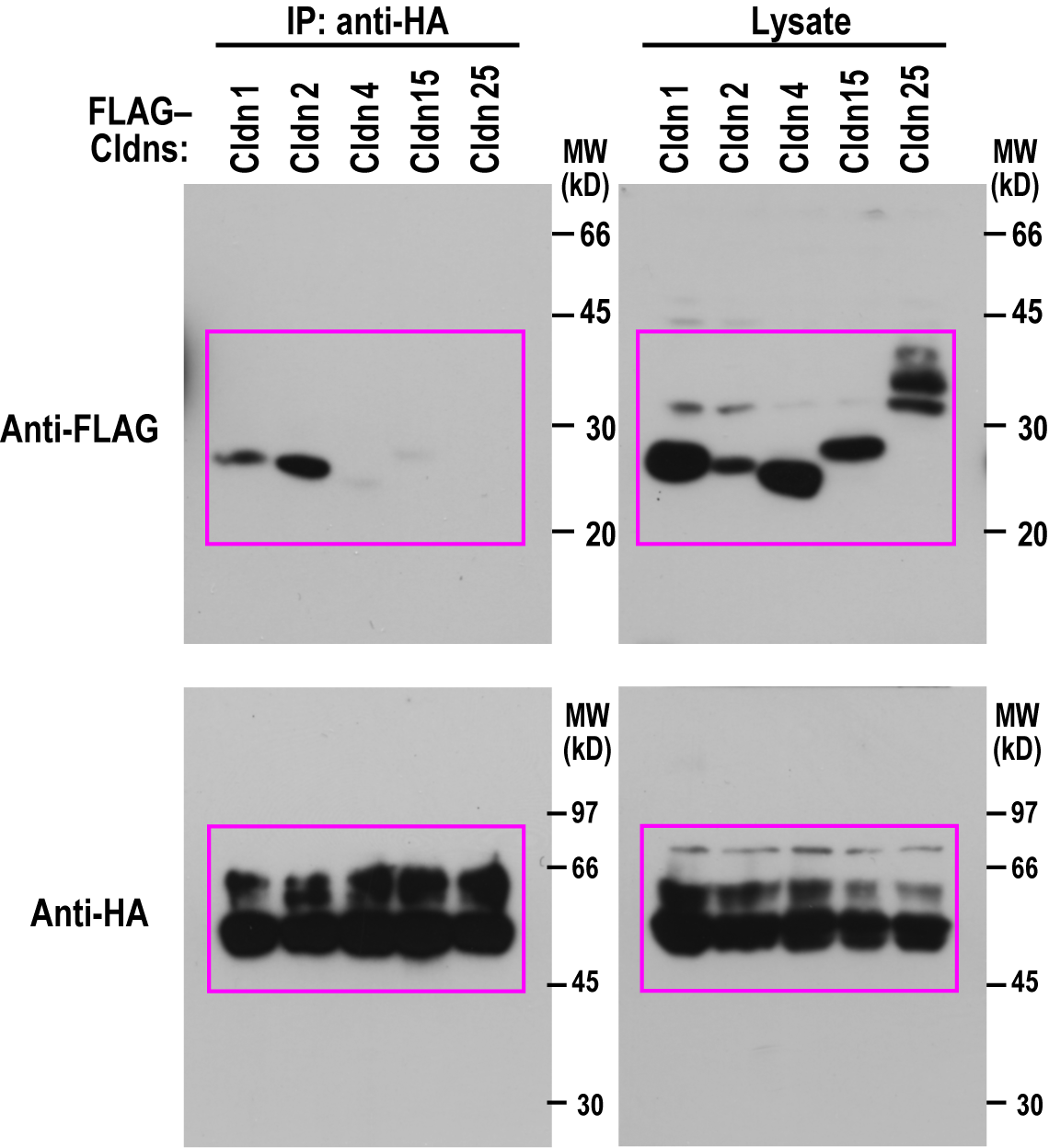

Supplement: Supplementary file 7 — Source Data Fig. 6 [file 44319_2023_18_MOESM7_ESM.zip › Figure_5/5B/5B.tif]

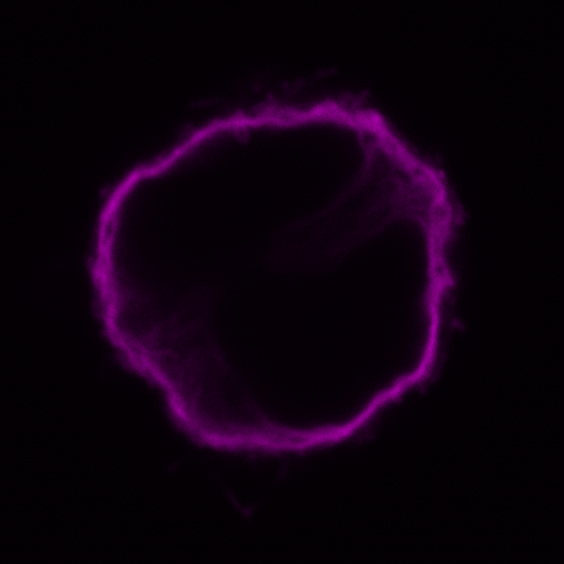

Supplement: Supplementary file 7 — Source Data Fig. 6 [file 44319_2023_18_MOESM7_ESM.zip › Figure_5/5D/Image Data/Right_HA.tif]

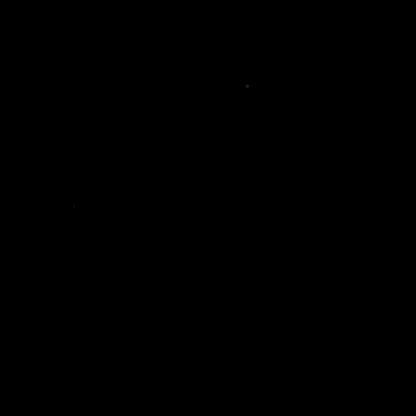

Supplement: Supplementary file 7 — Source Data Fig. 6 [file 44319_2023_18_MOESM7_ESM.zip › Figure_5/5D/Image Data/Right PLA signal.tif]

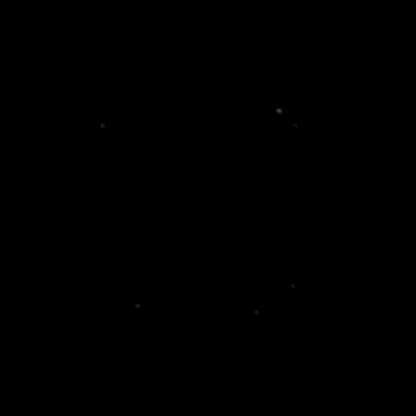

Supplement: Supplementary file 7 — Source Data Fig. 6 [file 44319_2023_18_MOESM7_ESM.zip › Figure_5/5D/Image Data/Left PLA signal.tif]

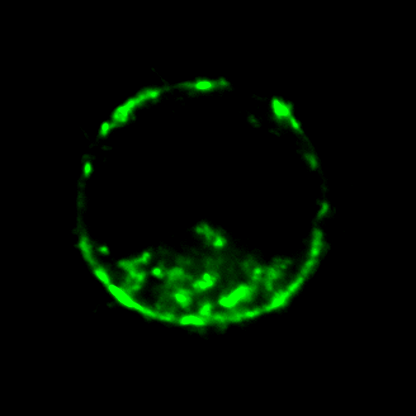

Supplement: Supplementary file 7 — Source Data Fig. 6 [file 44319_2023_18_MOESM7_ESM.zip › Figure_5/5D/Image Data/Left FLAG.tif]

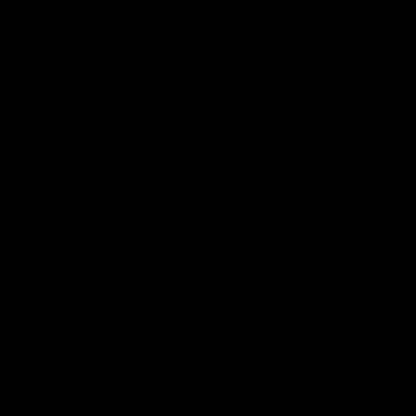

Supplement: Supplementary file 7 — Source Data Fig. 6 [file 44319_2023_18_MOESM7_ESM.zip › Figure_5/5D/Image Data/Right FLAG.tif]

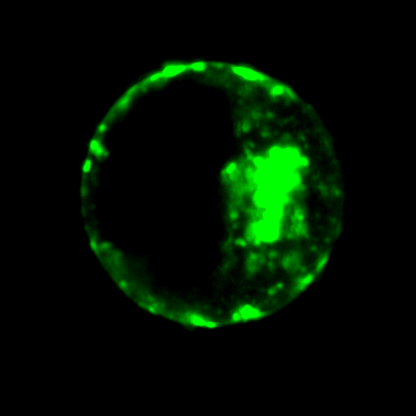

Supplement: Supplementary file 7 — Source Data Fig. 6 [file 44319_2023_18_MOESM7_ESM.zip › Figure_5/5D/Image Data/Middle FLAG.tif]

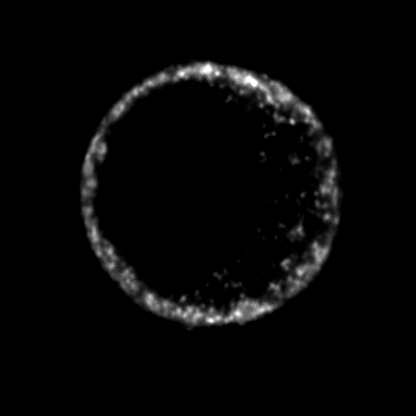

Supplement: Supplementary file 7 — Source Data Fig. 6 [file 44319_2023_18_MOESM7_ESM.zip › Figure_5/5D/Image Data/Middle PLA signal.tif]
