# Supplementary figures and images for: TMEM25 is a Par3-binding protein that attenuates claudin assembly during tight junction development (part 3 of 3)
Source: EMBO Rep. 2023 Dec 18;25(1):13. doi: 10.1038/s44319-023-00018-0 (PMC10897455; doi:10.1038/s44319-023-00018-0)

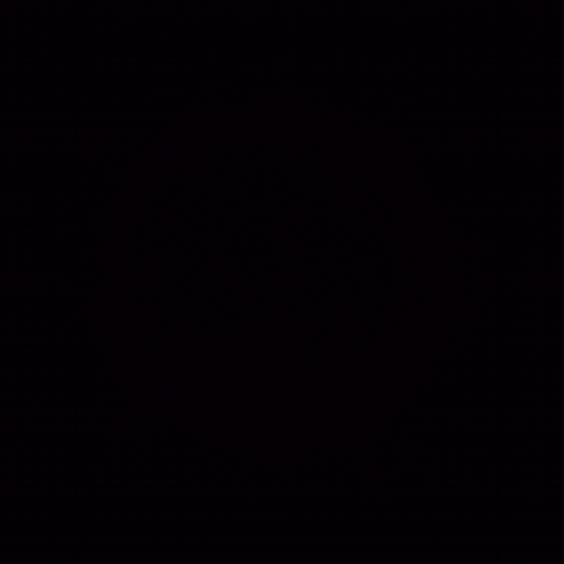

Supplement: Supplementary file 7 — Source Data Fig. 6 [file 44319_2023_18_MOESM7_ESM.zip › Figure_5/5D/Image Data/Left_HA.tif]

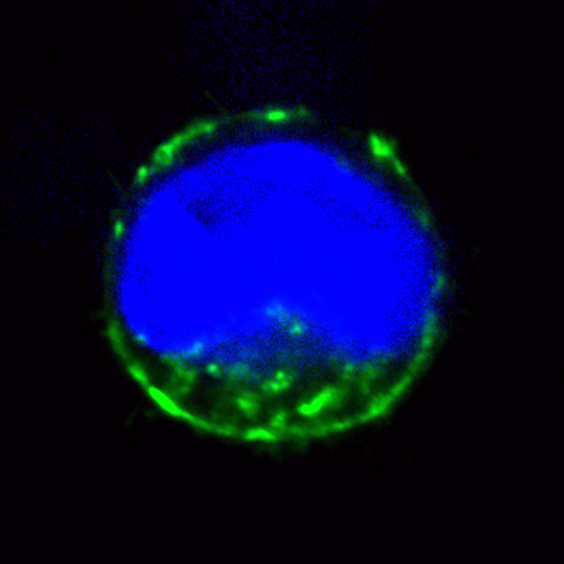

Supplement: Supplementary file 7 — Source Data Fig. 6 [file 44319_2023_18_MOESM7_ESM.zip › Figure_5/5D/Image Data/Left_Merge.tif]

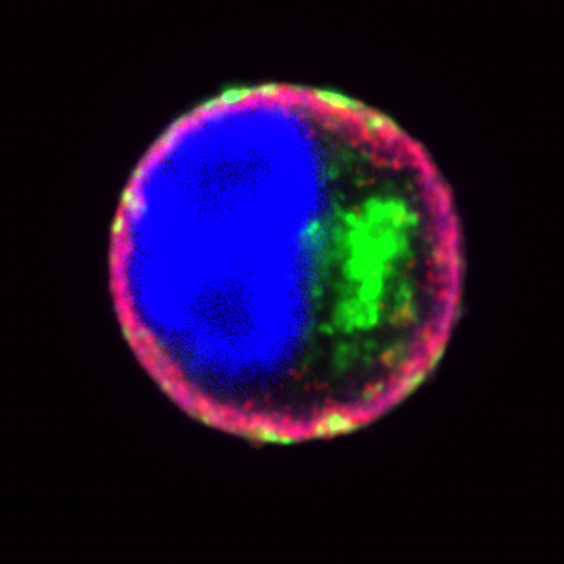

Supplement: Supplementary file 7 — Source Data Fig. 6 [file 44319_2023_18_MOESM7_ESM.zip › Figure_5/5D/Image Data/Middle_Merge.tif]

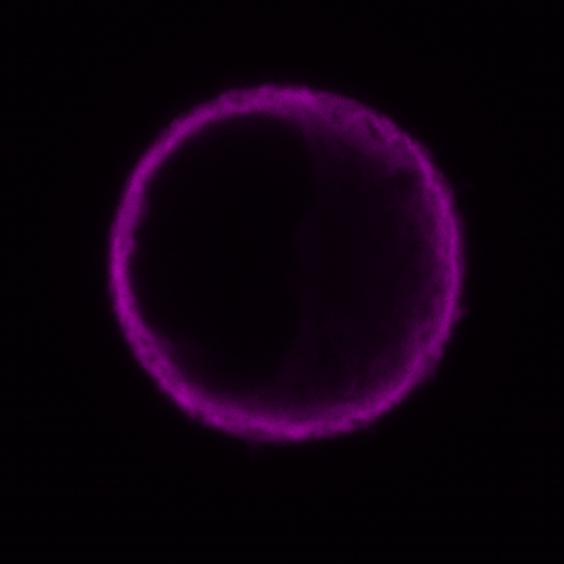

Supplement: Supplementary file 7 — Source Data Fig. 6 [file 44319_2023_18_MOESM7_ESM.zip › Figure_5/5D/Image Data/Middle_HA.tif]

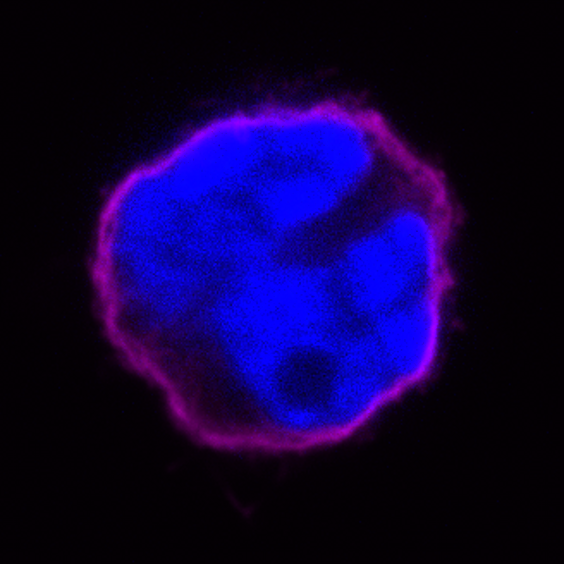

Supplement: Supplementary file 7 — Source Data Fig. 6 [file 44319_2023_18_MOESM7_ESM.zip › Figure_5/5D/Image Data/Right_Merge.tif]

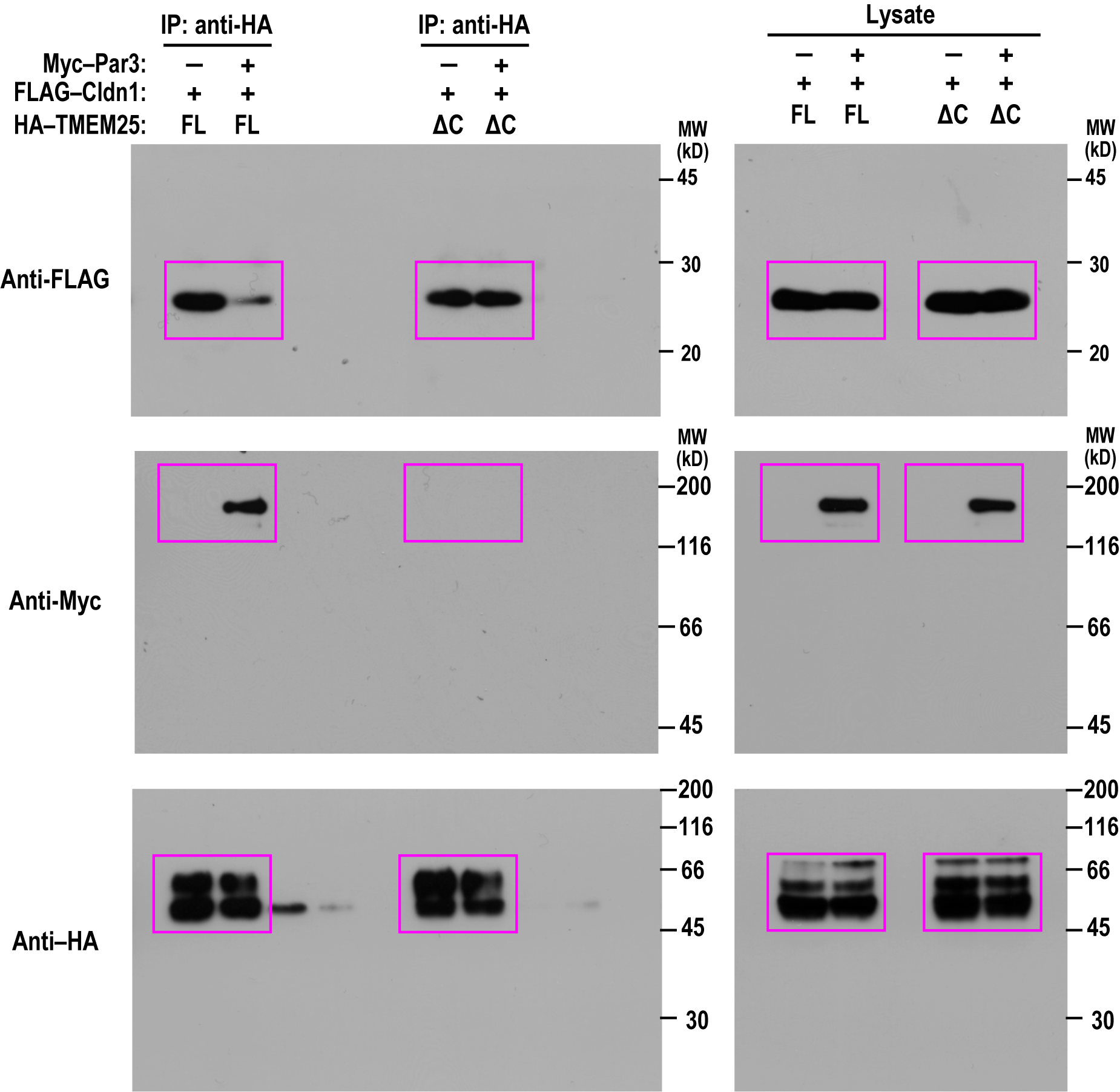

Supplement: Supplementary file 8 — Figure EV2 [file 44319_2023_18_MOESM8_ESM.zip › Figure_6/6F/6F.tif]

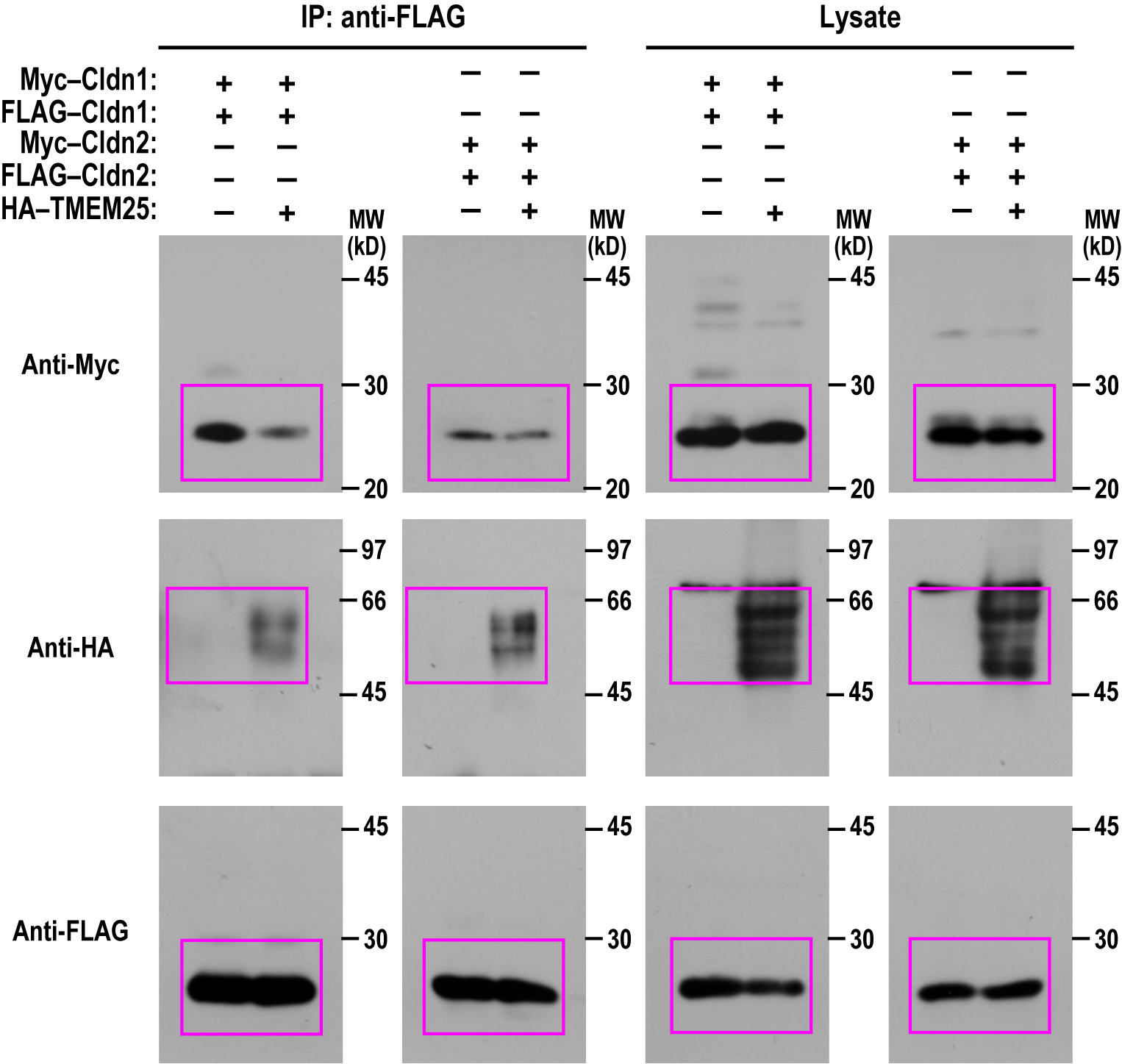

Supplement: Supplementary file 8 — Figure EV2 [file 44319_2023_18_MOESM8_ESM.zip › Figure_6/6A/6A.tif]

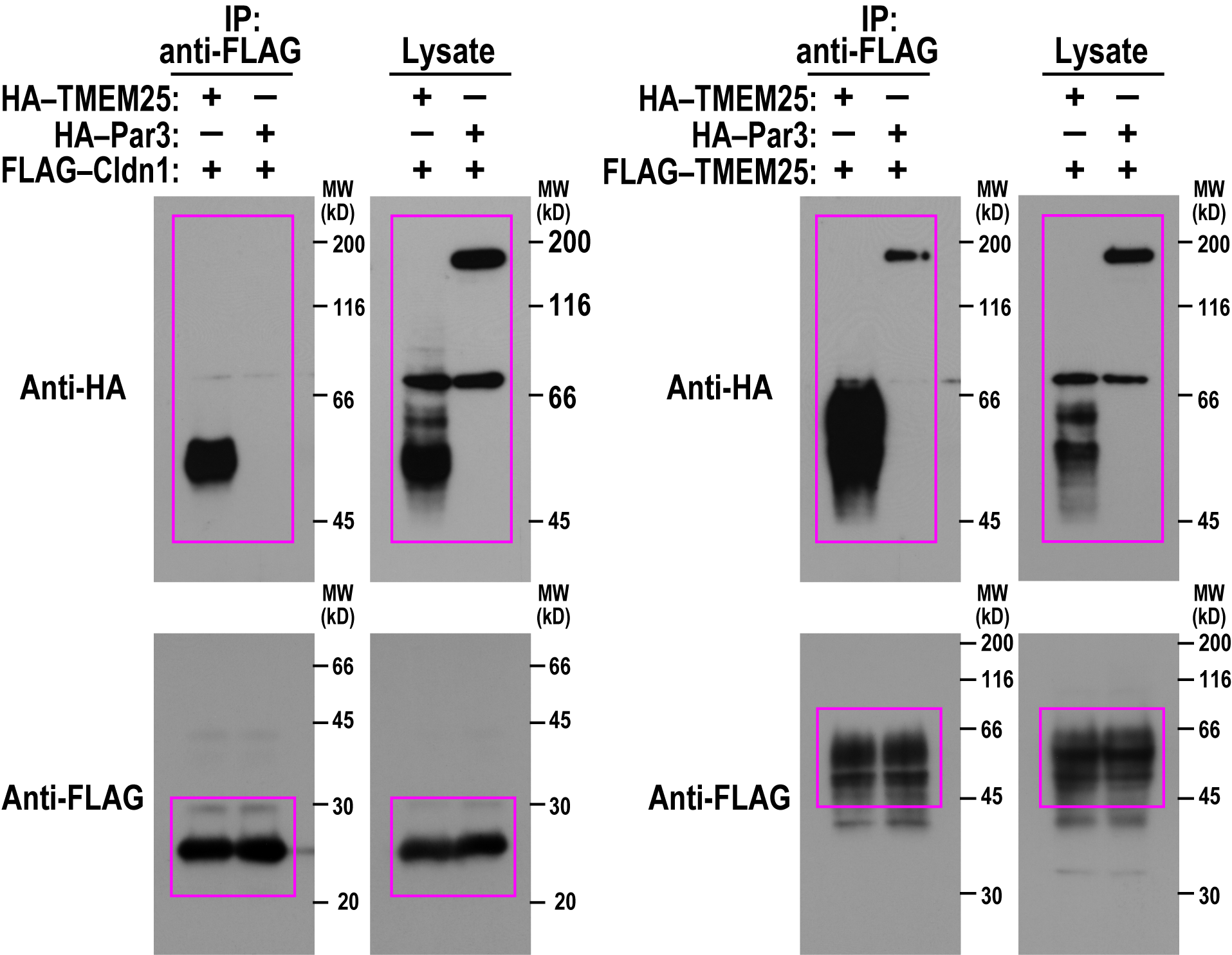

Supplement: Supplementary file 8 — Figure EV2 [file 44319_2023_18_MOESM8_ESM.zip › Figure_6/6G/6G.tif]

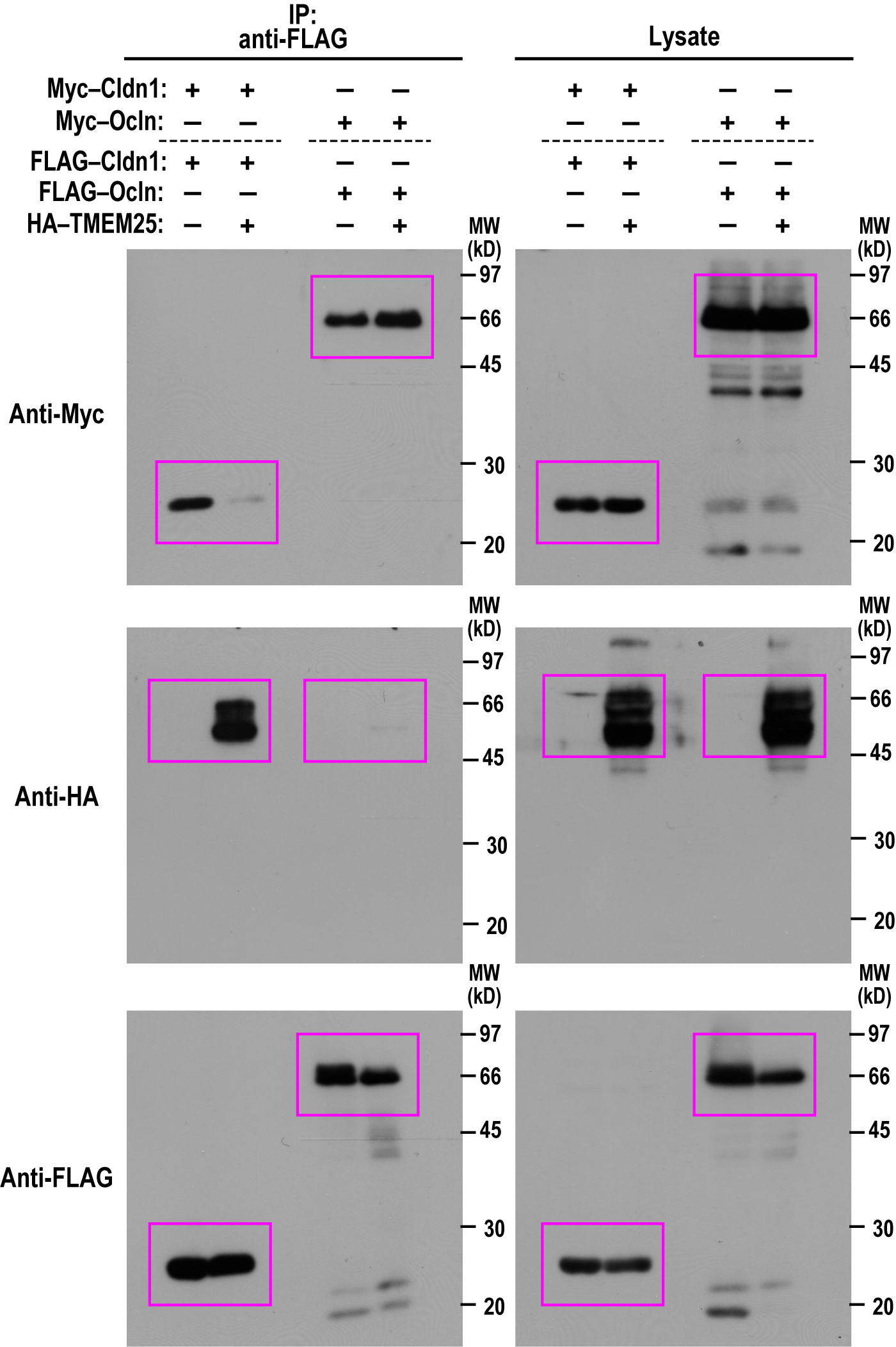

Supplement: Supplementary file 8 — Figure EV2 [file 44319_2023_18_MOESM8_ESM.zip › Figure_6/6B/6B.tif]

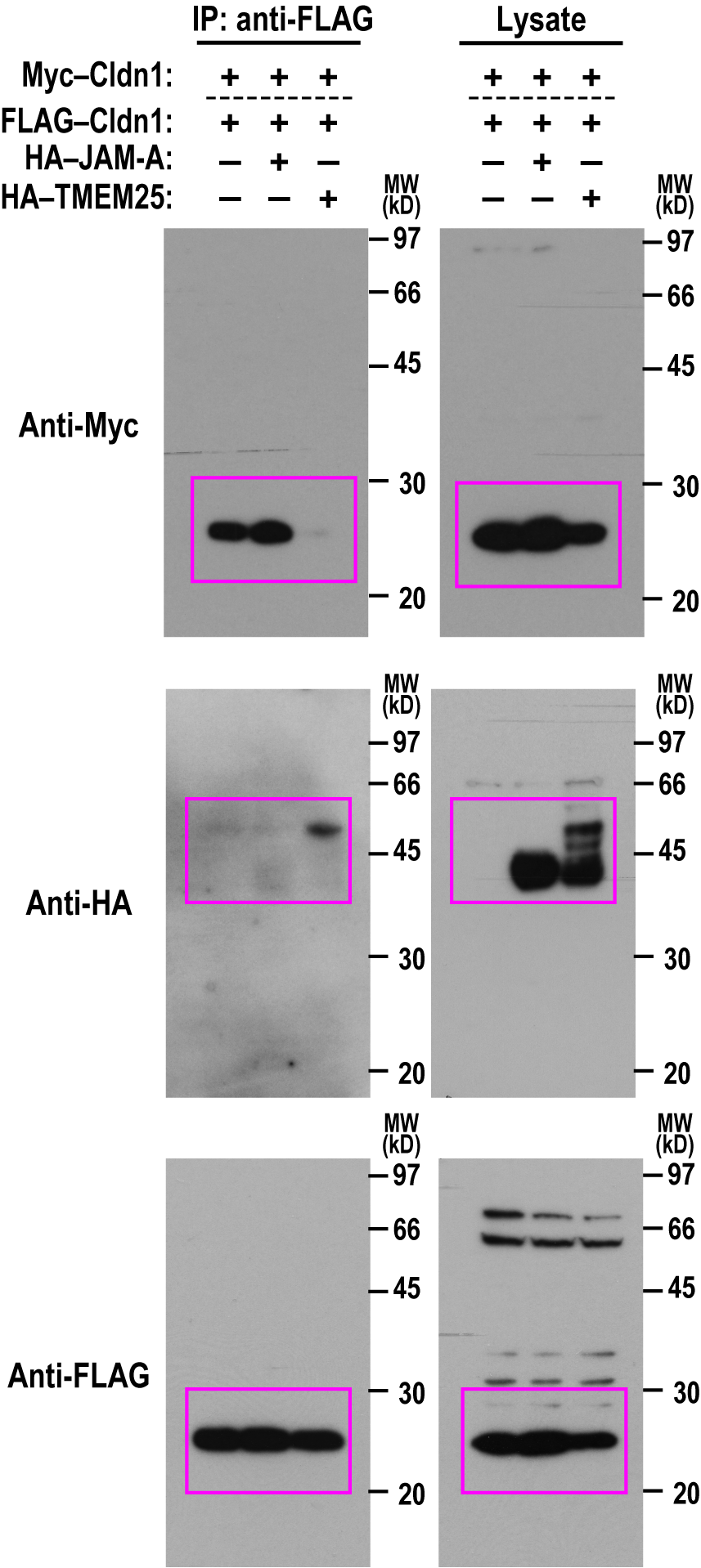

Supplement: Supplementary file 8 — Figure EV2 [file 44319_2023_18_MOESM8_ESM.zip › Figure_6/6D/6D.tif]

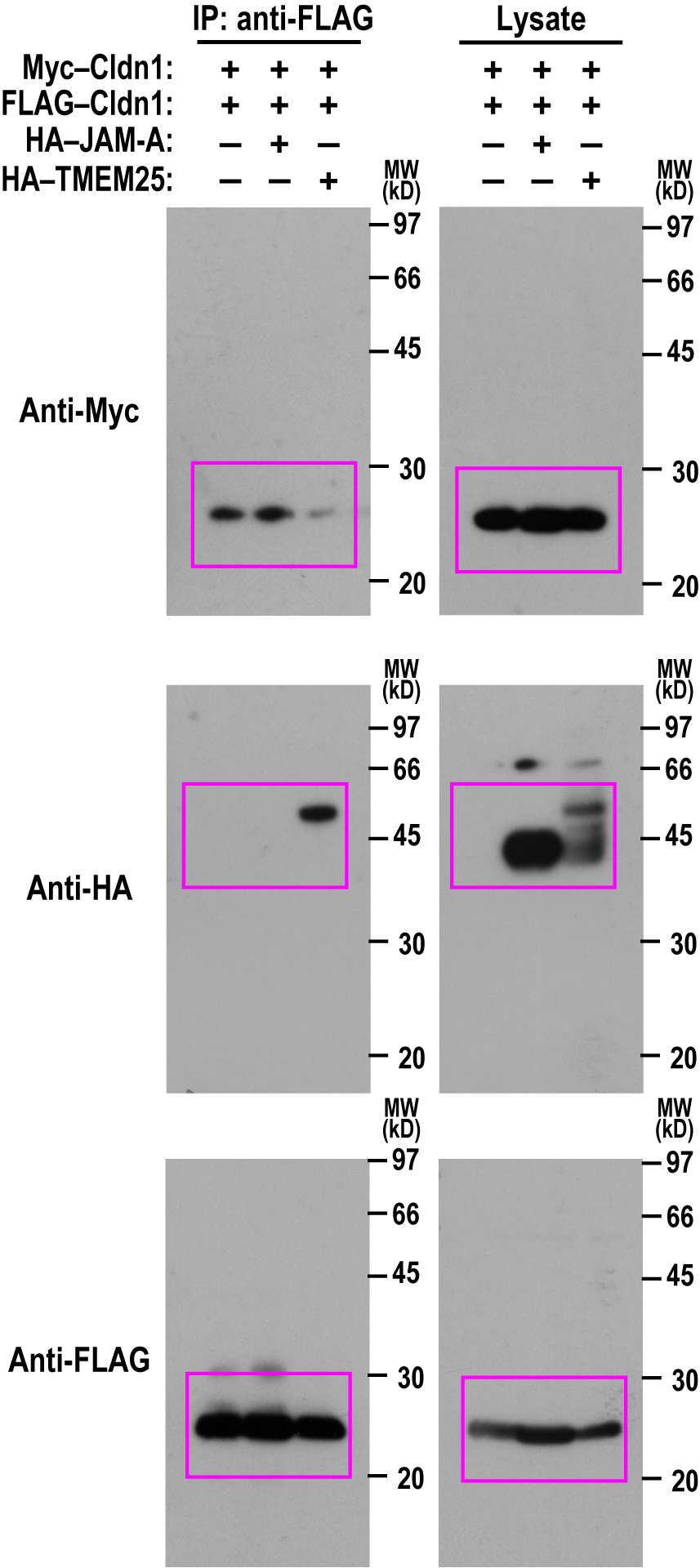

Supplement: Supplementary file 8 — Figure EV2 [file 44319_2023_18_MOESM8_ESM.zip › Figure_6/6C/6C.tif]

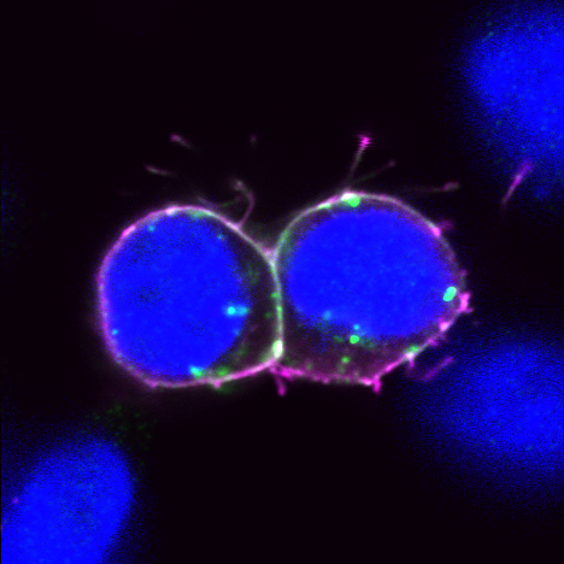

Supplement: Supplementary file 8 — Figure EV2 [file 44319_2023_18_MOESM8_ESM.zip › Figure_6/6E/Image data/Column4_merge.tif]

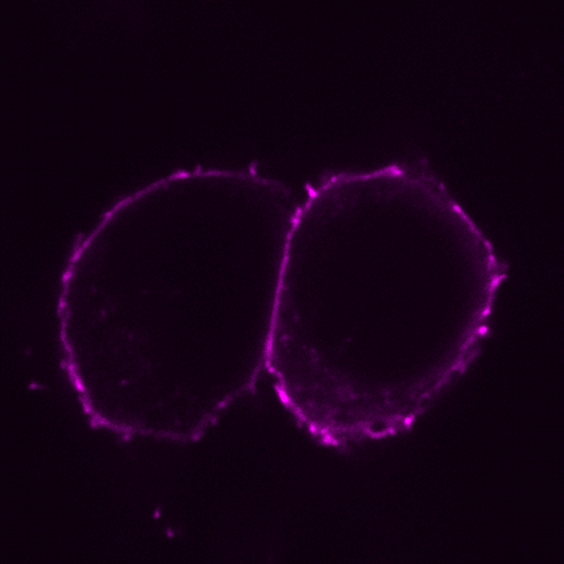

Supplement: Supplementary file 8 — Figure EV2 [file 44319_2023_18_MOESM8_ESM.zip › Figure_6/6E/Image data/Column1_HA.tif]

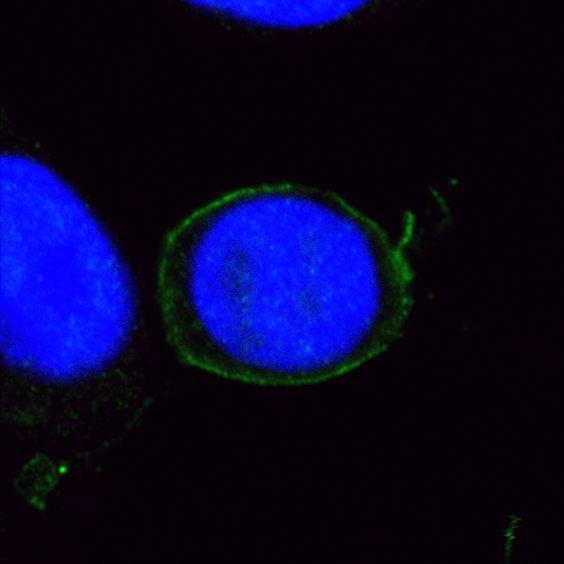

Supplement: Supplementary file 8 — Figure EV2 [file 44319_2023_18_MOESM8_ESM.zip › Figure_6/6E/Image data/Column2_merge.tif]

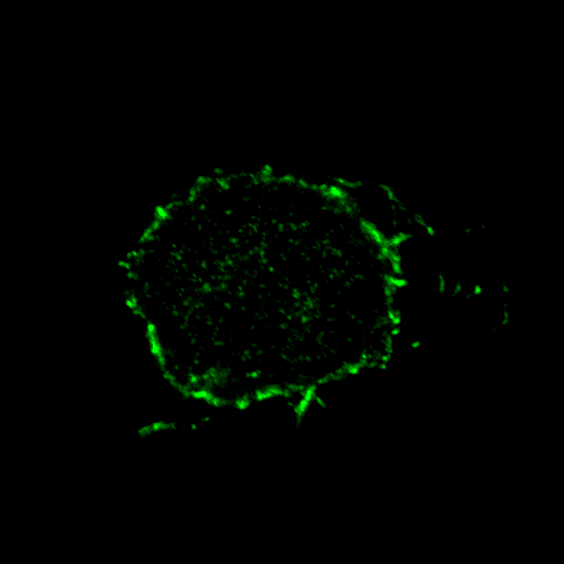

Supplement: Supplementary file 8 — Figure EV2 [file 44319_2023_18_MOESM8_ESM.zip › Figure_6/6E/Image data/column5_FLAG.tif]

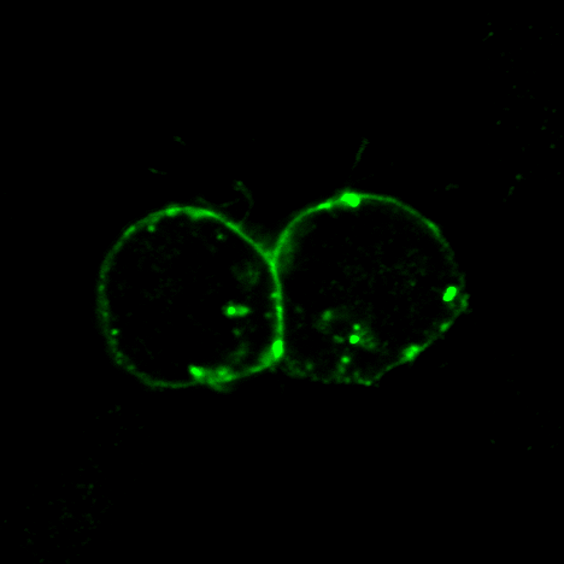

Supplement: Supplementary file 8 — Figure EV2 [file 44319_2023_18_MOESM8_ESM.zip › Figure_6/6E/Image data/column4_FLAG.tif]

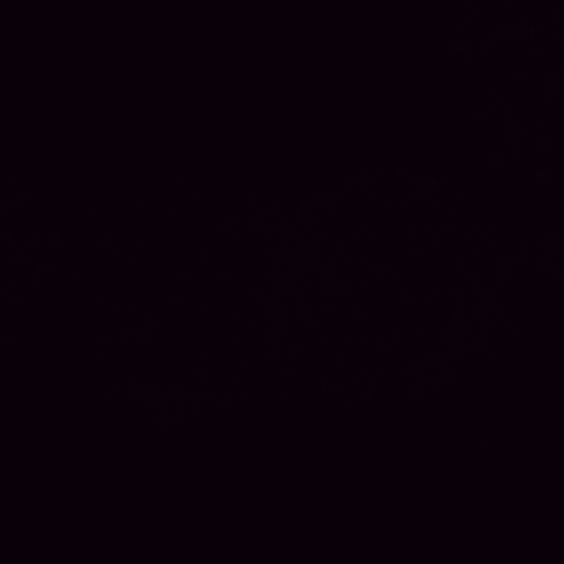

Supplement: Supplementary file 8 — Figure EV2 [file 44319_2023_18_MOESM8_ESM.zip › Figure_6/6E/Image data/Column3_HA.tif]

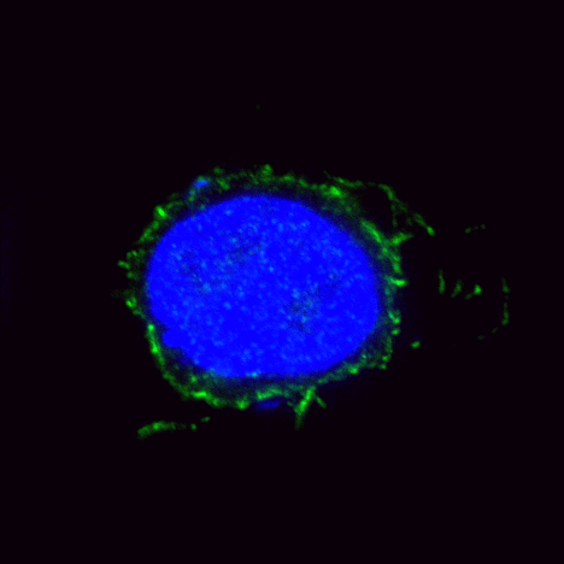

Supplement: Supplementary file 8 — Figure EV2 [file 44319_2023_18_MOESM8_ESM.zip › Figure_6/6E/Image data/Column5_merge.tif]

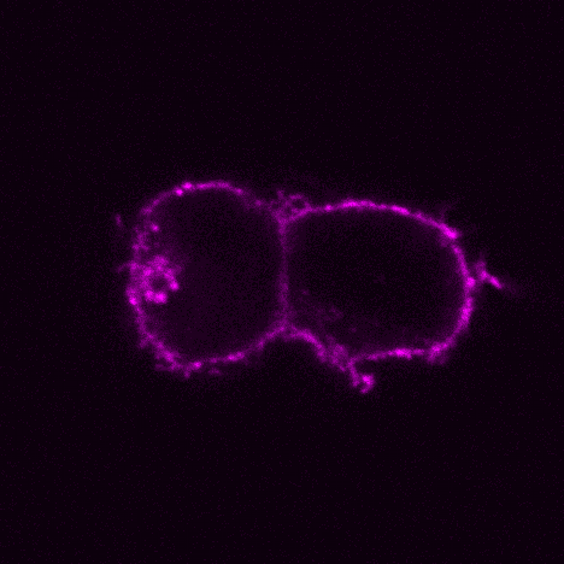

Supplement: Supplementary file 8 — Figure EV2 [file 44319_2023_18_MOESM8_ESM.zip › Figure_6/6E/Image data/Column7_HA.tif]

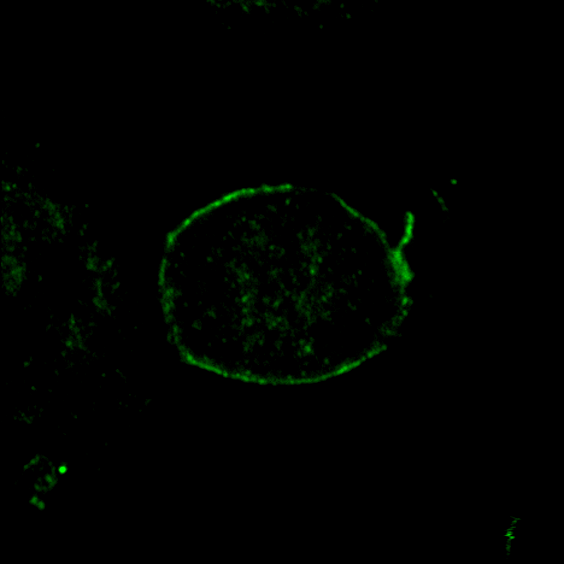

Supplement: Supplementary file 8 — Figure EV2 [file 44319_2023_18_MOESM8_ESM.zip › Figure_6/6E/Image data/column2_FLAG.tif]

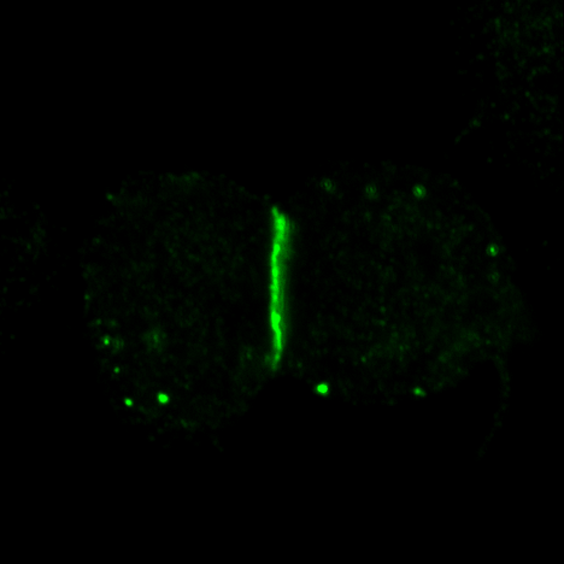

Supplement: Supplementary file 8 — Figure EV2 [file 44319_2023_18_MOESM8_ESM.zip › Figure_6/6E/Image data/column3_FLAG.tif]

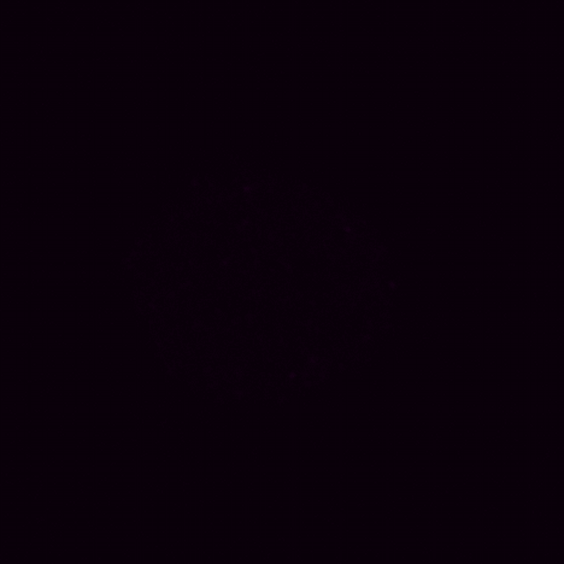

Supplement: Supplementary file 8 — Figure EV2 [file 44319_2023_18_MOESM8_ESM.zip › Figure_6/6E/Image data/Column5_HA.tif]

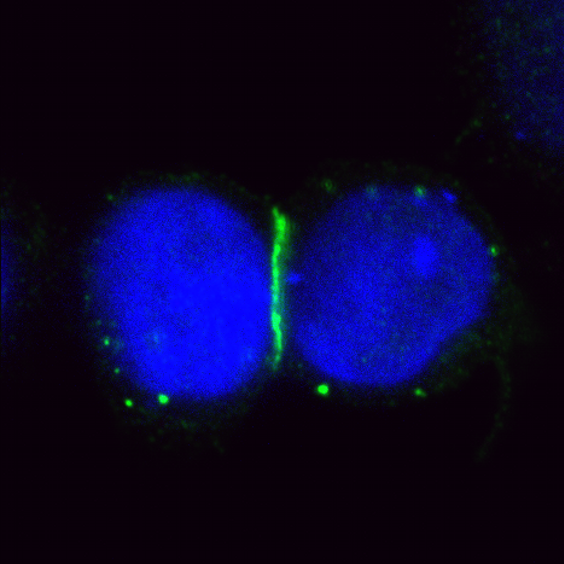

Supplement: Supplementary file 8 — Figure EV2 [file 44319_2023_18_MOESM8_ESM.zip › Figure_6/6E/Image data/Column3_merge.tif]

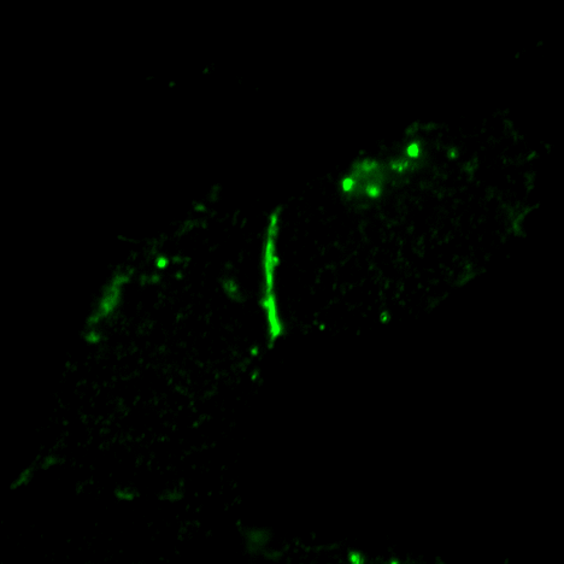

Supplement: Supplementary file 8 — Figure EV2 [file 44319_2023_18_MOESM8_ESM.zip › Figure_6/6E/Image data/column6_FLAG.tif]

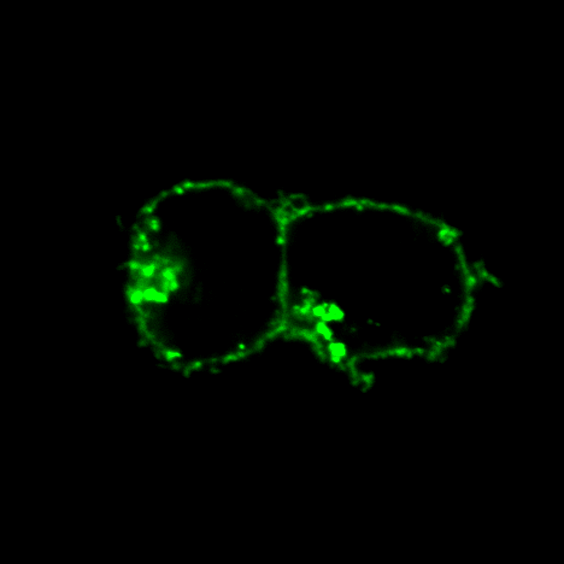

Supplement: Supplementary file 8 — Figure EV2 [file 44319_2023_18_MOESM8_ESM.zip › Figure_6/6E/Image data/column7_FLAG.tif]

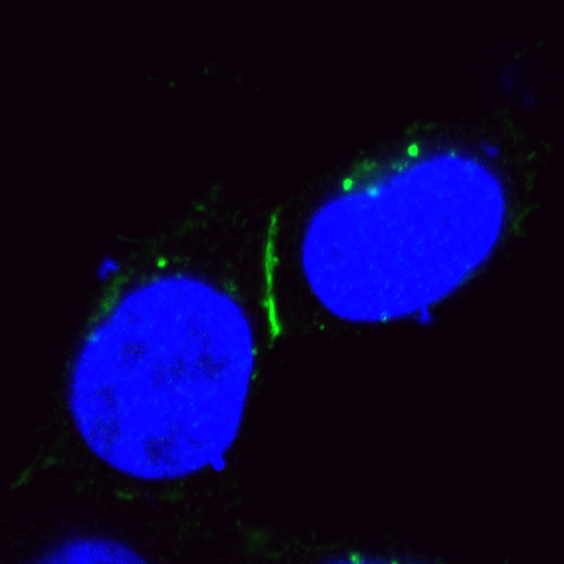

Supplement: Supplementary file 8 — Figure EV2 [file 44319_2023_18_MOESM8_ESM.zip › Figure_6/6E/Image data/Column6_merge.tif]

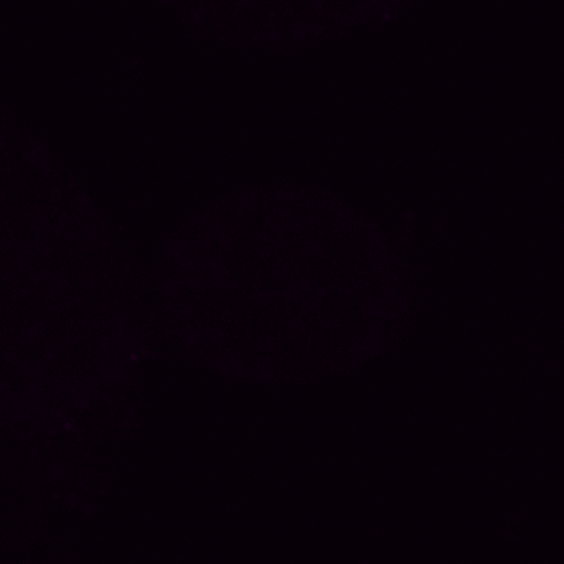

Supplement: Supplementary file 8 — Figure EV2 [file 44319_2023_18_MOESM8_ESM.zip › Figure_6/6E/Image data/Column2_HA.tif]

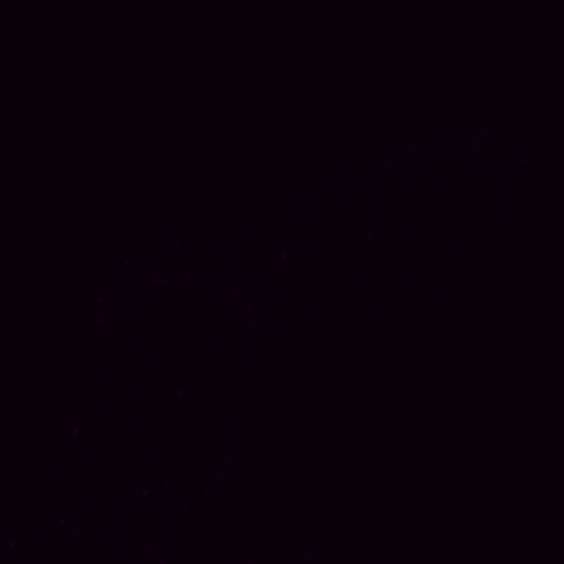

Supplement: Supplementary file 8 — Figure EV2 [file 44319_2023_18_MOESM8_ESM.zip › Figure_6/6E/Image data/Column6_HA.tif]

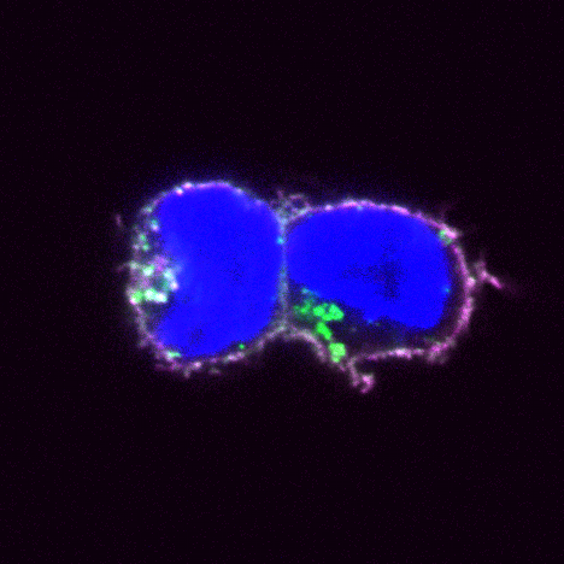

Supplement: Supplementary file 8 — Figure EV2 [file 44319_2023_18_MOESM8_ESM.zip › Figure_6/6E/Image data/Column7_merge.tif]

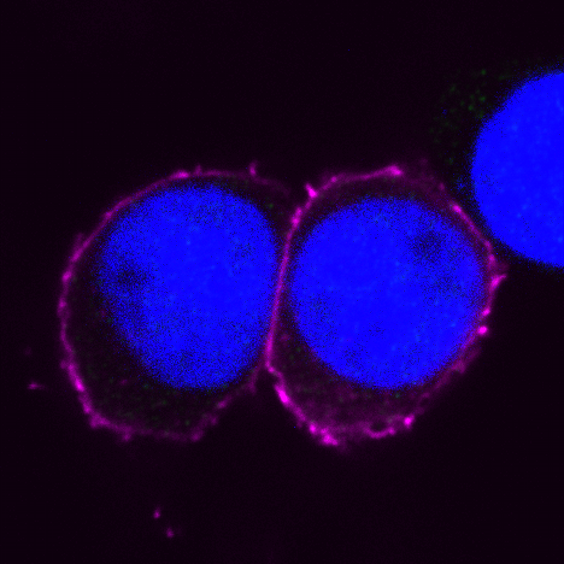

Supplement: Supplementary file 8 — Figure EV2 [file 44319_2023_18_MOESM8_ESM.zip › Figure_6/6E/Image data/Column1_merge.tif]

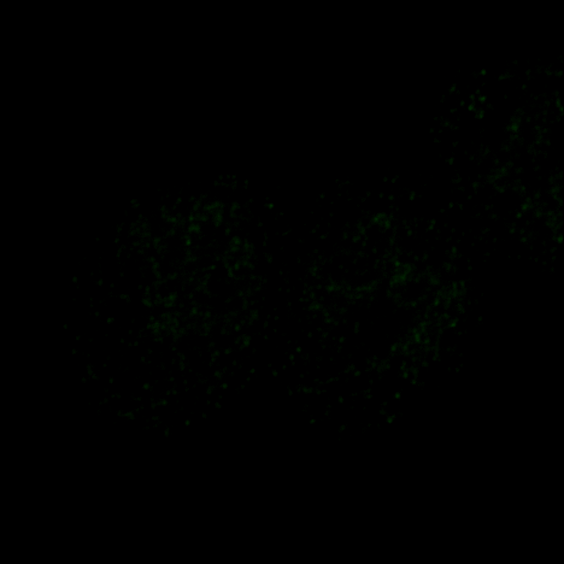

Supplement: Supplementary file 8 — Figure EV2 [file 44319_2023_18_MOESM8_ESM.zip › Figure_6/6E/Image data/column1_FLAG.tif]

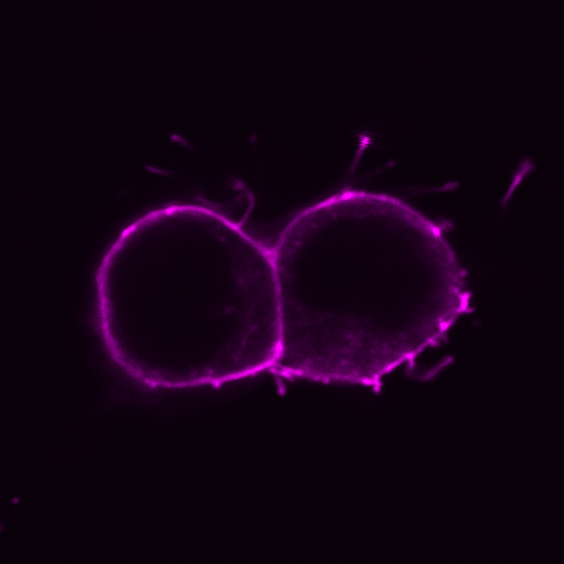

Supplement: Supplementary file 8 — Figure EV2 [file 44319_2023_18_MOESM8_ESM.zip › Figure_6/6E/Image data/Column4_HA.tif]
