# Supplementary material for: TMEM25 is a Par3-binding protein that attenuates claudin assembly during tight junction development
Source: EMBO Rep. 2023 Dec 18;25(1):13. doi: 10.1038/s44319-023-00018-0 (PMC10897455; doi:10.1038/s44319-023-00018-0)
Supplement: Supplementary file 9 — Expanded View Figures [file 44319_2023_18_MOESM9_ESM.pdf]

Expanded View Figures

Figure EV1. Multiple sequence alignment of TMEM25 proteins.

The amino acid sequences of TMEM25-related proteins from various species are aligned using the MAFFT program. Positions of the signal sequence, a C2-type immunoglobulin (Ig) domain, a region of an Ig-like fold, a transmembrane segment, and the PSD-95/DLG/ZO-1 (PDZ)-binding motif are indicated above the sequences. Potential *N*-glycosylation sites (Asn-*X*aa-Ser/Thr, where *X*aa is any amino acid but not proline) are underlined in magenta. Hs, *Homo sapiens* (human); Mm, *Mus musculus* (mouse); Cf, *Canis familiaris* (dog); Xt, *Xenopus tropicalis* (frog); Tr, *Takifugu rubripes* (puffy fish); and Ol, *Oryzias latipes* (Medaka fish).

© The Author(s)

TMEM25

```
                            signal sequence                    C2-type Ig domain
Human(Hs)      1 M-ALPPGPAALRHTLLLLPALLSSGWGELEP--QIDG-QTWAERALRENERHAFTCRVAG
Mouse(Mm)      1 M-ELPLSQATLRHTLLLLPALLSSGQGELAP--QIDG-QTWAERALRENEHHAFTCRVAG
Dog(Cf)        1 M-APPPGPAALPRSLLLLPALLSSGWGELAP--QIDG-QTWAERALRENEQHAFTCRVAG
Frog(Xt)       1 M--------LLRVSLLFFQPLFQRGLG----------------EPLQKDETEGASCEGGG
Fugu(Tr)       1 MGHVCVRSWASSATVMLFHTLTFSWTGAMEPTSKQEGRQQQAAMALQEDVTHQFSCHSDG
Medaka(Ol)     1 MRTASPGSWTSGSAVVFFNTLALSWTGAADSVLKING-QHQAAVTLQENMTLKFNCQTDS
                 *            :::: .*                *      .*:::      .*.  .

Human(Hs)     57 --GPGTPRLAWYLDGQLQEASTSRLLSVGGEA---------FSGGTSTFTVTAHRAQHEL
Mouse(Mm)     57 --GSATPRLAWYLDGQLQEATTSRLLSVGGDA---------FSGGTSTFTVTAQRSQHEL
Dog(Cf)       57 --GSGTPRLAWYLDGQLQEAGTSRLLSVGGEA---------FSGGTSTFTVTAQRAQHEL
Frog(Xt)      37 ------PSLSWYMNGVKQEEGLGREPPFLLPV---------YPGSSSVLTLVETRGE---
Fugu(Tr)      61 RDPRHPLVIRWHLDGNWQKQEPSKHRRLAMTSGRDSDAVHLGYGHNSTFSLRPRKWNREL
Medaka(Ol)    60 WDPRAPPLLTWYLNGVQQKEPSSNRGRLTATSKKDSKVTRPGTNHNSTFSLQARKWDREL
                       : *:::*  *:    ..    .               . .*.:::    : :
                                                     Ig-like region
Human(Hs)    106 NCSLQDPRSGRSANASVILNVQFKPEIAQVGAKYQEAQGPGLLVVLFALVRANPPANVTW
Mouse(Mm)    106 NCSLQDPGSGRPANASVILNVQFKPEIAQVGAKYQEAQGPGLLVVLFALVRANPPANVTW
Dog(Cf)      106 NCSLQDPGSGRSANASVILNVQFKPEIAQVGAKYQDSQGPGLLVVLFALVRANPPANVTW
Frog(Xt)      79 NCS------DAWLKGEELLSVHFPPD-----SPVNSAHTPGISLLLLLVVRTQPASTFTL
Fugu(Tr)     121 VCVASNPRTGERYNATITLSLQFKPEILRVNVNHSETSDPAFALVLFALVRSNPPATISF
Medaka(Ol)   120 VCVALNPSTGQSYNATITLNVQFQPEILRVNAHFTETSDPGLSLVLFALVRSNPSATISF
                  *      :.   :.   *.::* *:          .:  *.: ::*: :**::*.:..:

Human(Hs)    166 IDQDGPVTVNTSDFLVLDAQNYPWLTNHTVQLQLRSLAHNLSVVATNDVGVTSASLPAPG
Mouse(Mm)    166 IDQDGPVTVNASDFLVLDAQNYPWLTNHTVQLQLRSLAHNLSVVATNDVGVTSASLPAPG
Dog(Cf)      166 IDQDGPVTVNTSDFLVLDAQNYPWLTNHTVQLQLRSLAHNLSVVATNDVGVTSASLPAPG
Frog(Xt)     128 RDHDGRKTLNSSRLLLLDTRNLD--TNGSLRVKVS----------TEERGVSHTSVSALG
Fugu(Tr)     181 VDQSGQLVADTTDFLLLDSQTNPQLANNTLRIMLSSLSGTLSLNVTNTAGTVQSNLTLAE
Medaka(Ol)   180 VDQLGQPVANTSDLLTLDSQRYPWLNNHTLRVRLSSLSGNISLNASNSVGAVQSNLTLAE
                  *: *  . ::: :* **::      * :::: :           ::  *.  :.:.
                                    transmembrane
Human(Hs)    226 LLATRVEVPLLGIVVAAGLALGTLVGFSTLVACLVCR-KEKKTKGPSRHPSLISSDSNNL
Mouse(Mm)    226 LLATRIEVPLLGIVVAGGLALGTLVGFSTLVACLVCR-KEKKTKGPSRRPSLISSDSNNL
Dog(Cf)      226 LLATRVEVPLLGIIMAGGLALGALVGFSTLVACLVCR-KEKKTKGPSRRPSLISSDSNNL
Frog(Xt)     176 LLSSHVEVPLFALVVGGAGVVAGILLVNALVCCLLLKRKRRSYGVRNQLTL---STSNNM
Fugu(Tr)     242 FLQSRVEVPMLGIVTGGAMAFMALLILSLIVLCLMQKNKSKSFDQPVEIVMTKKSDSASM
Medaka(Ol)   240 FLQSRVEVPMLGIVTGGAMAFMALLILSLIVLCLMQKNKSKSFDEPVEIVMTTKSESANL
                 :* :::***::.:: ... ..  :: .. :* **: : * :.      .      * * .:

Human(Hs)    285 K---LNNVRLPRENMSLPSNLQLNDLTPDSRAVKPADRQMAQNNSRPELLDPEPGGL---
Mouse(Mm)    285 K---LNNVRLPRENMSLPSNLQLNDLTPDLRG-KATERPMAQHSSRPELLEAEPGGL---
Dog(Cf)      285 K---LNNVRLPRENMSLPSNLQLNDLTPDSRG-KPADRQMAQNNSRPELLDPEPGGL---
Frog(Xt)     233 K---LNNSCLPREHMSLPSNLQLNDLRPQARG--------PLGSS--EGETQEDASLRCG
Fugu(Tr)     301 RAEGAGTSHLPRDHMSLPSHVQLNDLSTLTKA-AQ---QNPGGGKREEEEEEEDLSLV--
Medaka(Ol)   300 QTGRADKAHIPRENMSLPSNMQLNDLSTLRKA-RQTALQIRVGEK--DEEEEEDLSLA--
                 :     ..  :**::*****::***** .  :.              .  :    *  .*
                                      PDZ-binding motif
Human(Hs)    339 -LTSQGFIRLPVLGYIYRVSSVSSDEIWL 366
Mouse(Mm)    338 -LTSRGFIRLPMLGYIYRVSSVSSDEIWL 365
Dog(Cf)      338 -LTSRGFIRLPMLGYIYRVSSVSSDEIWL 365
Frog(Xt)     280 NLDDTGFDRFPLVGYIYKASSVSSDEIWL 308
Fugu(Tr)     355 -YAARGFARYPMVGYIYKVNSTSSEEIWL 382
Medaka(Ol)   355 -YAARGFARYPMVGYIYKVNSTSSEEIWL 382
                      ** * *::****:..*.**:****
```

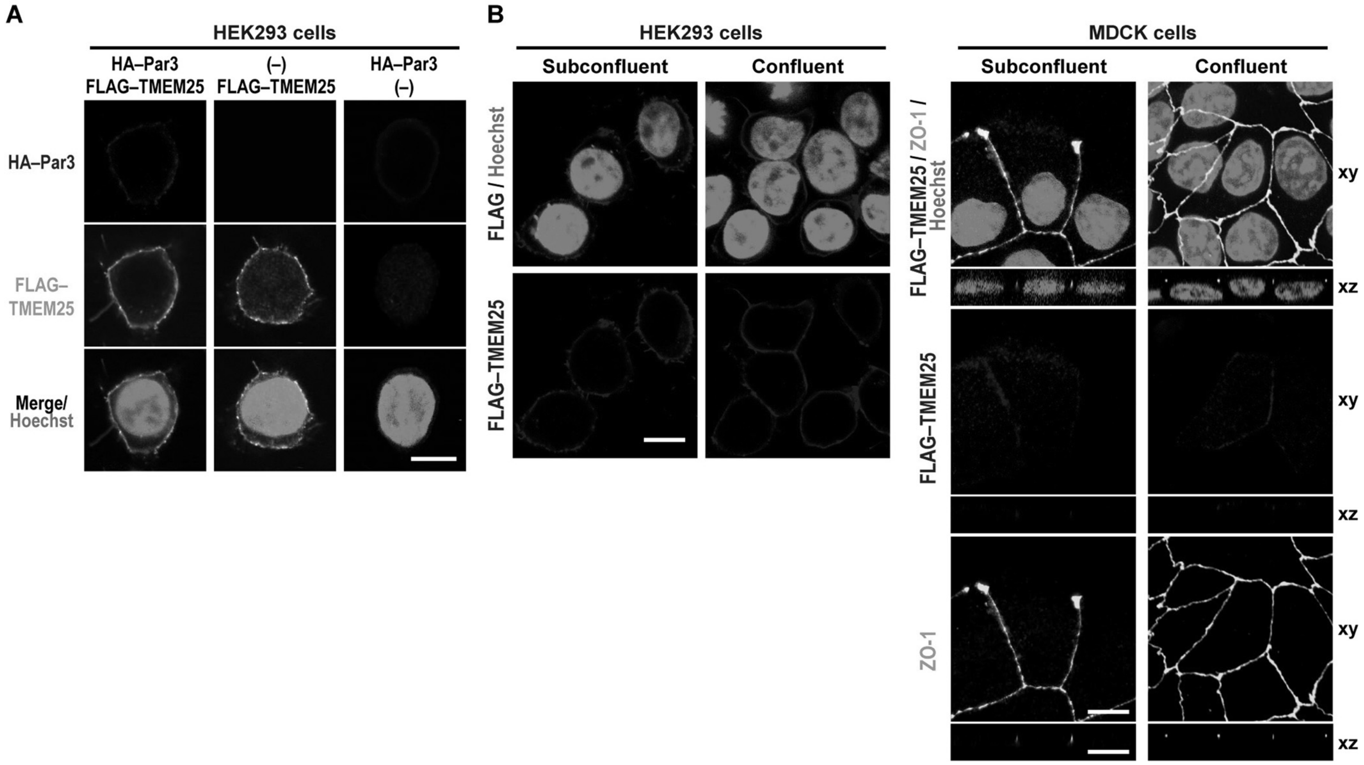

Figure EV2. Plasma membrane localization of TMEM25 expressed in HEK293 cells and MDCK cells.

(**A**) Par3 and TMEM25 expressed in HEK293 cells co-localized at the plasma membrane. HEK293 cells expressing HA–Par3 and/or FLAG–TMEM25 were fixed and stained as indicated. Scale bar, 10 μm. (**B**) Plasma membrane localization of TMEM25 in confluent and sub-confluent cell cultures. HEK293 cells (left) or MDCK cells (right) expressing FLAG–TMEM25 were grown for 48 h in confluent or sub-confluent cultures, and then fixed and stained with the indicated antibodies and Hoechst. Shown are confocal images (left) or stacked images (xy images in right) of the cells. Cross-sectional z-stack analysis is also shown (xz images in right). Scale bar, 10 μm. Source data are available online for this figure

 © The Author(s)

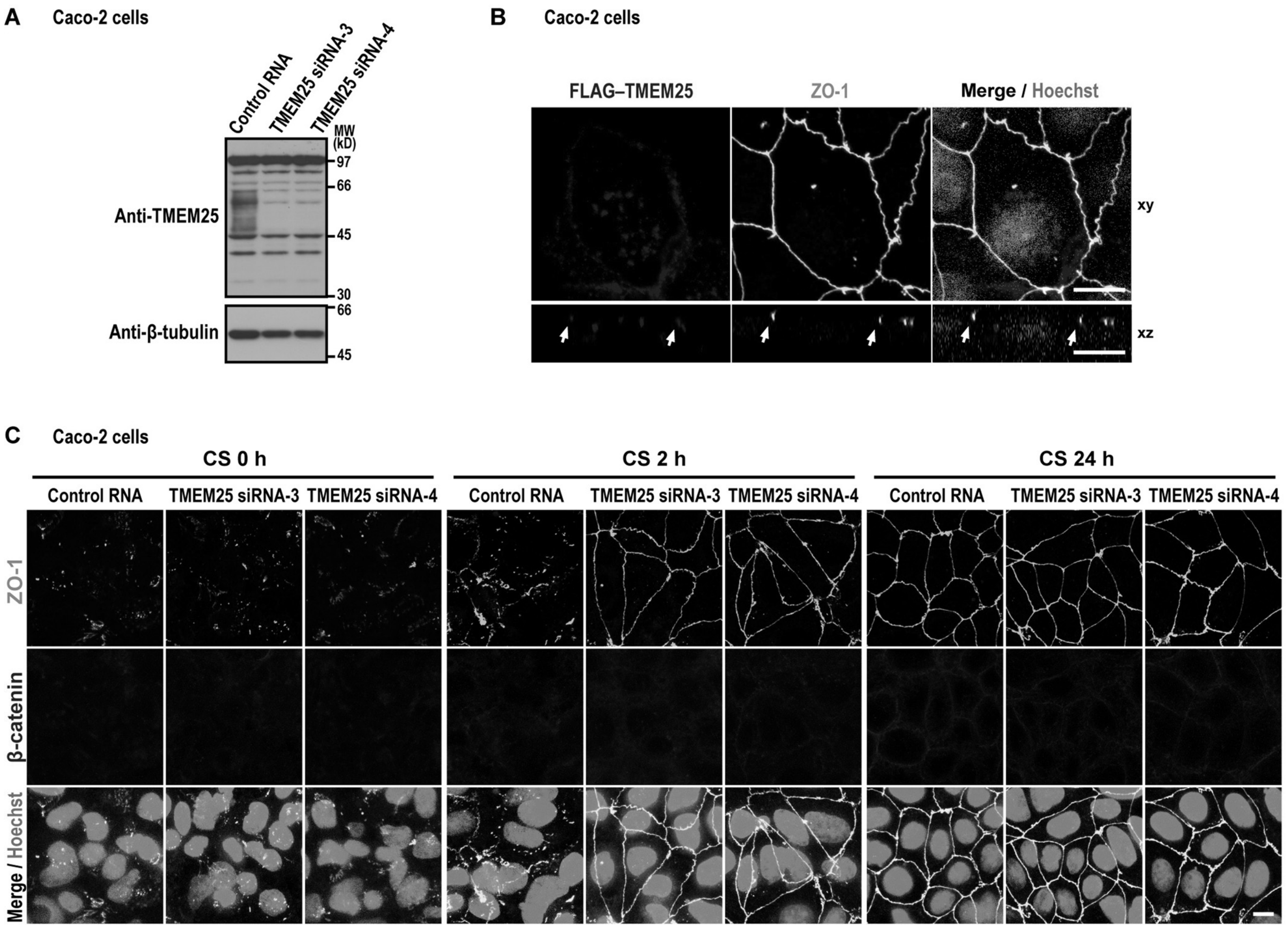

Figure EV3. Effect of TMEM25 depletion on TJ development in Caco-2 cells.

(**A**) The expression of endogenous TMEM25 in Caco-2 cells. Proteins in the lysate of Caco-2 cells transfected with control RNA or TMEM25 siRNA were analyzed by immunoblot with the indicated antibodies; a commercially obtained TMEM25 antibody (from Boster) recognizes endogenous TMEM25, which is effectively knocked down by the TMEM25-specific siRNAs. Positions for marker proteins are indicated in kilodaltons (kD). (**B**) Localization of TMEM25 to the TJ in Caco-2 cells. Caco-2 cells transfected with the Tet-on system plasmid pTetOne-FLAG–TMEM25 were grown on the Transwell chamber for 5 days, and treated with 0.2 μg/ml doxycycline for 48 h. Confocal images of the cells were stacked along the z-axis (xy). Cross-sectional z-stack analysis is also shown (xz). Arrows indicate the positions of TJs. Scale bar, 10 μm. (**C**) TJ development in TMEM25-depleted Caco-2 cells after Ca^{2+} switch. Shown are representative images of TMEM25-depleted Caco-2 cells after Ca^{2+} switch. Cells were fixed 0 h, 2 h, or 24 h after Ca^{2+} switch (CS) and stained with the indicated antibodies and Hoechst. Confocal images of the cells were stacked along the z-axis. Scale bar, 10 μm.

© The Author(s)

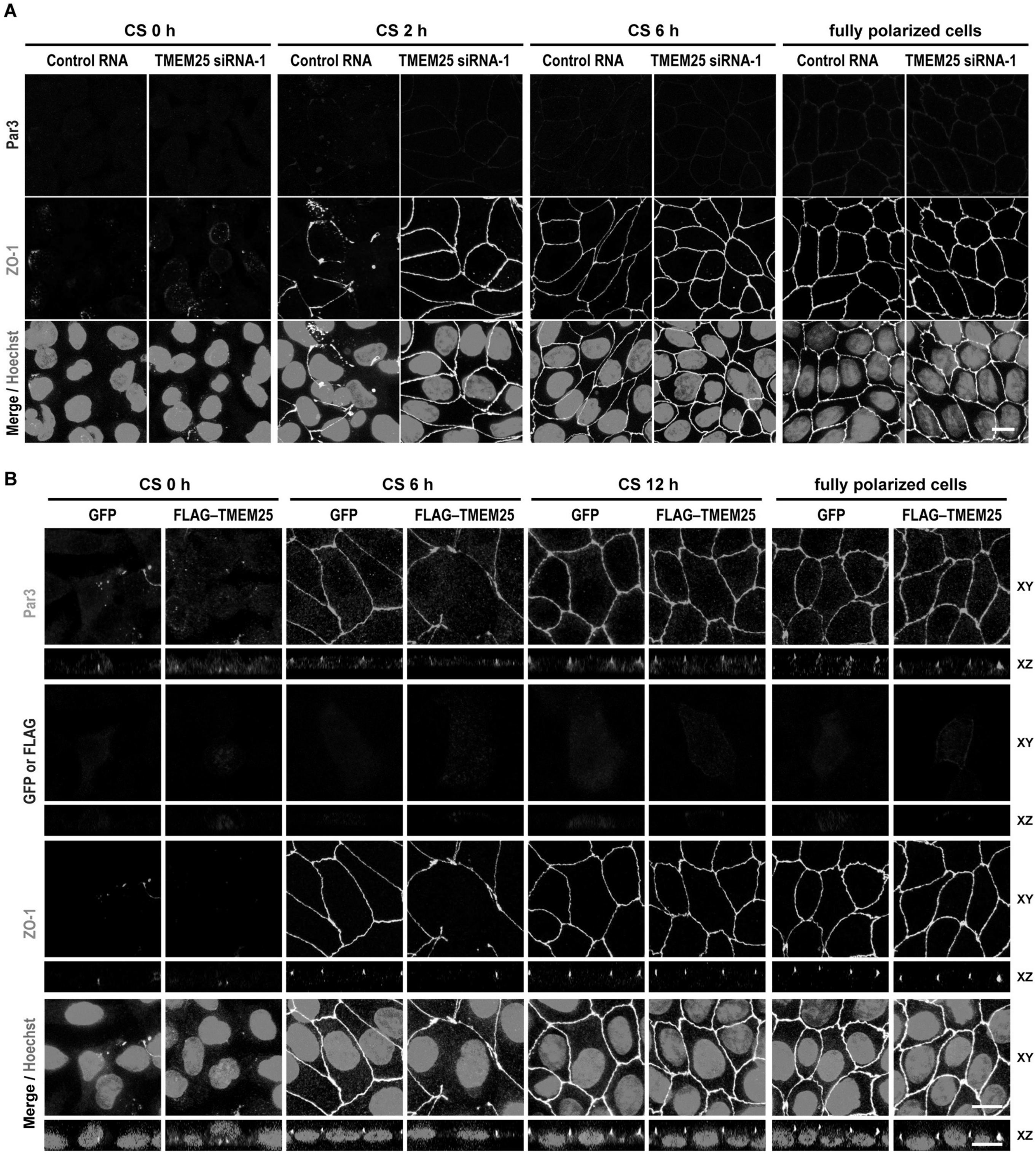

Figure EV4. Localization of Par3 in TMEM25-depleted or TMEM25-expressing MDCK cells after Ca^{2+} switch.

Shown are representative images of TMEM25-depleted MDCK cells (**A**) or the cells expressing GFP alone or FLAG-tagged TMEM25 induced by the Tet-on system in the presence of doxycycline (5 μg/ml) (**B**). Cells were fixed after culture for 72 h (fully polarized cells) or at the indicated time points after Ca^{2+} switch (CS), and stained with the indicated antibodies and Hoechst. Confocal images of the cells were stacked along the z-axis (**A** and xy images in **B**). Cross-sectional z-stack analysis is also shown (xz images in **B**). Scale bar, 10 μm.

 © The Author(s)

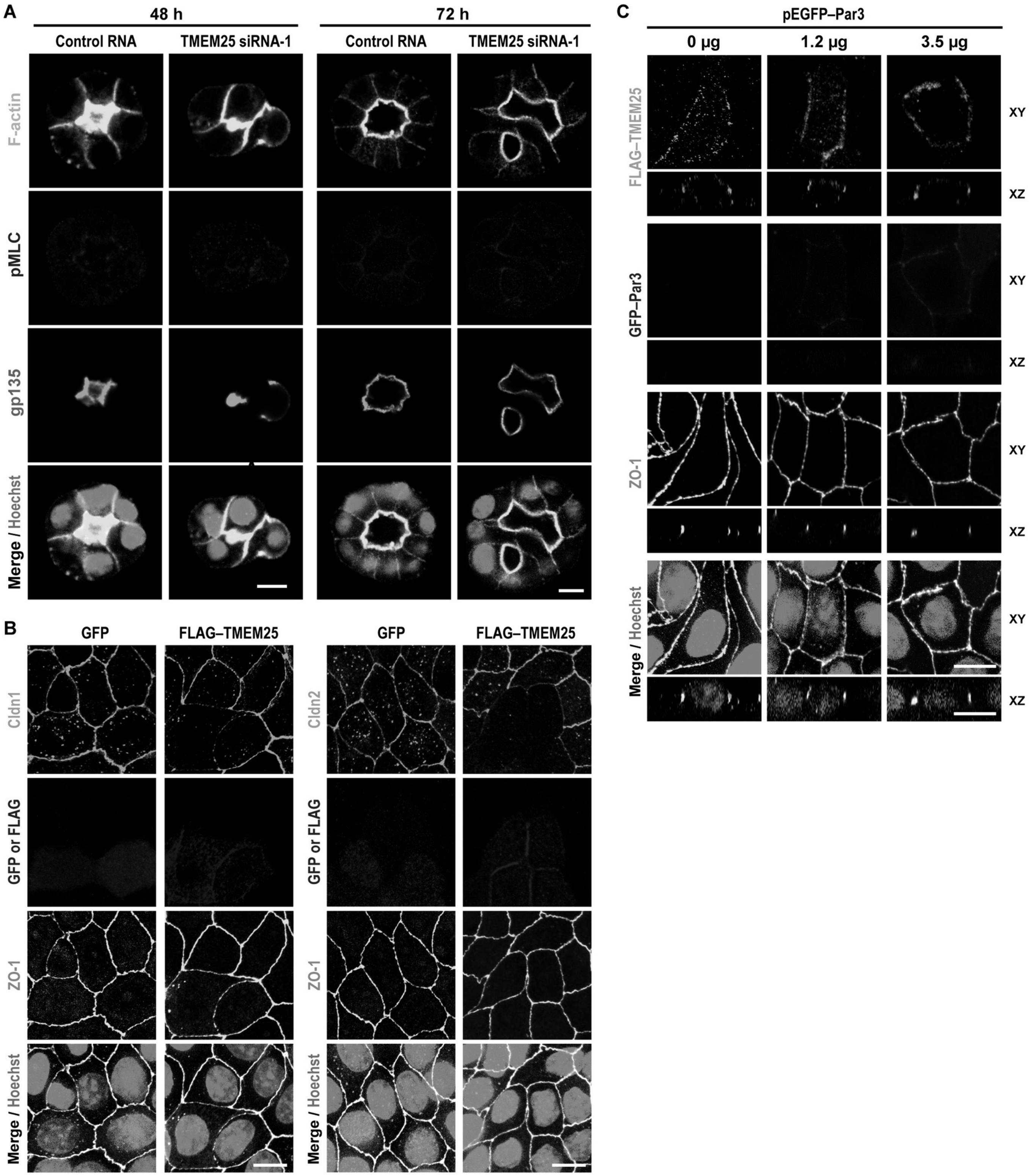
A
48 h
72 h
Control RNA
TMEM25 siRNA-1
Control RNA
TMEM25 siRNA-1
F-actin
pMLC
gp135
Merge / Hoechst
B
GFP
FLAG–TMEM25
GFP
FLAG–TMEM25
Cldn1
Cldn2
GFP or FLAG
ZO-1
Merge / Hoechst
C
pEGFP–Par3
0 µg
1.2 µg
3.5 µg
FLAG–TMEM25
GFP–Par3
ZO-1
Merge / Hoechst
XY
XZ

Figure EV5. Effect of TMEM25 depletion on the actomyosin organization in MDCK cysts.

(**A**) The actomyosin organization in TMEM25-depleted cysts. MDCK cells were transfected with the control RNA or TMEM25 siRNA and grown for 48 h or 72 h in 3D culture and stained with the indicated antibodies, phalloidin (F-actin), and Hoechst. Shown are representative confocal images of cysts. pMLC2, phosphorylated myosin light chain 2. Scale bar, 10 μm. (**B**) TMEM25-mediated suppression of claudin enrichment at the sites of cell–cell contact in MDCK cells. GFP alone or FLAG–TMEM25 was induced by the Tet-on system in the presence of doxycycline (5 μg/ml) in MDCK cells. Cells were then cultured for 48 h, fixed, and stained with the indicated antibodies and Hoechst. Confocal images of the cells were stacked along the z-axis. Scale bar, 10 μm. (**C**) Effect of Par3 overexpression on the localization of TMEM25. MDCK cells (2×10^6 cells) were co-transfected with pEGFP–Par3 (0, 1.2, or 3.5 μg) and the Tet-on system plasmid pTetOne-FLAG–TMEM25 (1.5 μg). After culture for 24 h, FLAG–TMEM25 were induced in the presence of doxycycline (1 μg/ml), and cells were cultured for further 48 h, fixed and stained with the indicated antibodies and Hoechst. The amount of pEGFP–Par3 plasmid used for the transfection is shown. Confocal images of the cells were stacked along the z-axis (xy). Cross-sectional z-stack analysis is also shown (xz). Scale bar, 10 μm.

 © The Author(s)
